# Photoredox catalytic radical fluorosulfonylation of olefins enabled by a bench-stable redox-active fluorosulfonyl radical precursor

**DOI:** 10.1038/s41467-022-31089-7

**Published:** 2022-06-11

**Authors:** Peng Wang, Honghai Zhang, Xingliang Nie, Tianxiao Xu, Saihu Liao

**Affiliations:** 1 Key Laboratory of Molecule Synthesis and Function Discovery (Fujian Province University), State Key Laboratory of Photocatalysis on Energy and Environment, College of Chemistry, Fuzhou University, 350108 Fuzhou, China; 2 Beijing National Laboratory of Molecular Science (BNLMS), 100190 Beijing, China

**Keywords:** Reactive precursors, Photocatalysis, Chemical tools, Synthetic chemistry methodology

## Abstract

Sulfonyl fluorides have attracted considerable and growing research interests from various disciplines, which raises a high demand for novel and effective methods to access this class of compounds. Radical flurosulfonylation is recently emerging as a promising approach for the synthesis of sulfonyl fluorides. However, the scope of applicable substrate and reaction types are severely restricted by limited known radical reagents. Here, we introduce a solid state, redox-active type of fluorosulfonyl radical reagents, 1-fluorosulfonyl 2-aryl benzoimidazolium triflate (FABI) salts, which enable the radical fluorosulfonylation of olefins under photoredox conditions. In comparison with the known radical precursor, gaseous FSO_2_Cl, FABI salts are bench-stable, easy to handle, affording high yields in the radical fluorosulfonylation of olefins with before challenging substrates. The advantage of FABIs is further demonstrated in the development of an alkoxyl-fluorosulfonyl difunctionalization reaction of olefins, which forges a facile access to useful β-alkoxyl sulfonyl fluorides and related compounds, and would thus benefit the related study in the context of chemical biology and drug discovery in the future.

## Abstract (web summary)

Sulfonyl fluorides are compounds with potential application in chemical biology and drug discovery, but their preparation can be challenging. Here, the authors present a type of bench-stable fluorosulfonyl radical reagents that enable radical fluorosulfonylation reactions via photoredox catalysis.

## Introduction

**Fig. 1:**
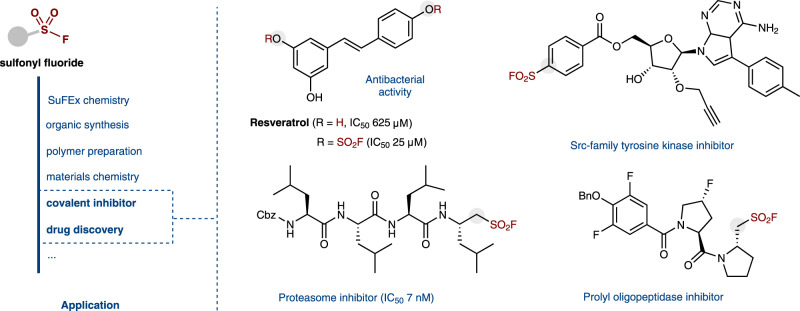
Applications of sulfonyl fluorides. Examples of biologically active molecules containing a SO_2_F moiety.

Since Sharpless and co-workers introduced sulfur (VI) fluoride exchange (SuFEx) reactions as a new generation of click chemistry^1^, the popularity of sulfonyl fluorides has grown dramatically over the recent past^1–4^, with applications in a wide range of fields, including organic synthesis^5–10^, polymer preparation^11–14^, materials science^15–17^, chemical biology etc^18–20^. Particularly, unique and appealing properties were often observed, which has attracted a fast-growing research interest on sulfonyl fluoride being a privileged warhead in chemical biology and drug discovery^1,4,18^, and successful examples keep emerging in the past years^18–27^. Remarkably, enhanced activity was often observed when the SO_2_F moiety was introduced, resembling, to some extent, the common beneficial effect of trifluoromethyl and fluorine groups in pharmaceuticals^18–20,28–33^. For example, Sharpless, and co-workers recently found that fluorosulfonylated Resveratrol showed a potent agent against resistant bacteria, higher than that of the parent compound by over 200-fold (Fig. 1)^33^. Accordingly, novel and efficient synthetic protocols to broaden the scope of available sulfonyl fluorides are desirable^1–4,19–21^.

**Fig. 2:**
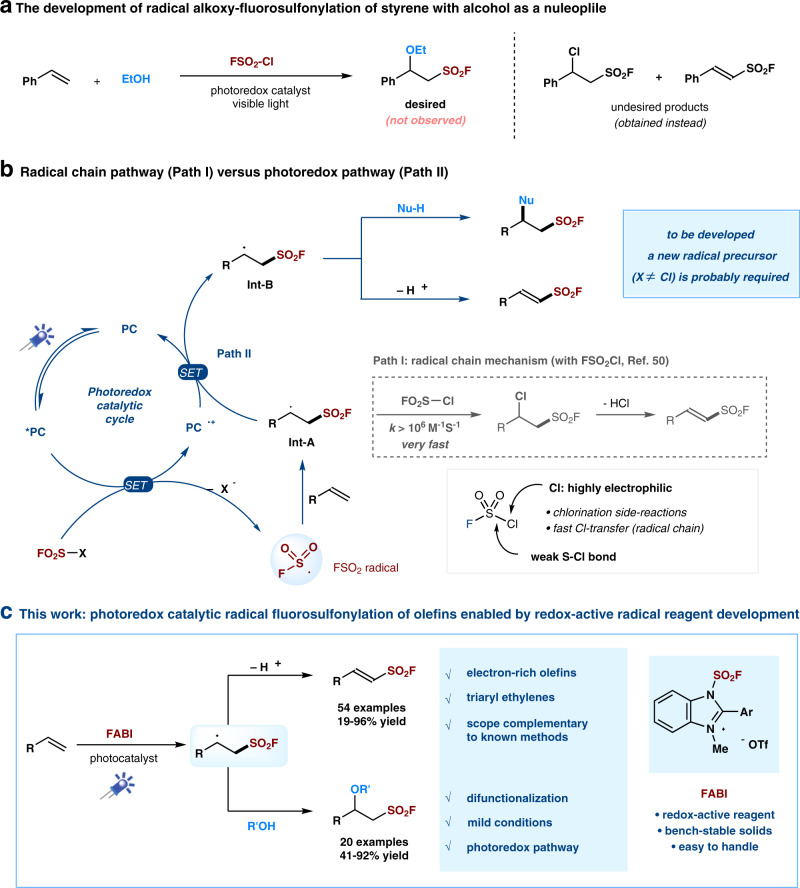
The development of photoredox catalytic radical fluorosulfonylation of olefins. **a** The development of radical alkoxy-fluorosulfonylation of styrene with alcohol as a nuleophile. **b** Radical fluorosulfonylation of alkenes via radical chain pathway (Path I) versus photoredox pathway (Path II). **c** This work: photoredox catalytic radical fluorosulfonylation of olefins enabled by redox-active radical reagent development.

Among the most common methods for the synthesis of sulfonyl fluorides^1–4,34–46^, direct fluorosulfonylation^45–48^ undoubtedly represents a concise and effective approach, and could be particularly useful in the late-stage modifications of drugs and biomolecules^1–4^. Most of the fluorosulfonylating reagents reported so far belong to the FSO_2_ + -type of synthons, including the well-known sulfuryl fluoride gas (SO_2_F_2_)^1^ and other solid reagents (such as FDIT, recently reported by Sharpless, Dong et al., which exhibited high reactivity in the fluorosulfonylation of phneols and amines^47,48^. In contrast, fluorosulfonylation with the corresponding fluorosulfonyl radical (FSO_2_•) remains less investigated^4^, likely due to the instability and challenging preparation^49^. Recently, we used sulfuryl chlorofluoride (FSO_2_Cl) as a radical precursor and we reported the radical fluorosulfonylation of alkenes^50,51^, affording an effective method for the preparation of important alkenyl sulfonyl fluorides^50–56^. However, when we applied this reagent to the development of other transformations, e.g., the alkoxy-fluorosulfonylation reaction of styrene (Fig. 2a), we failed to obtained any desired product even after extensive optimization. Instead, undesired chloro- and styryl-sulfonyl fluorides were obtained, which were supposed to the products from a radical chain mechanism (Path I, Fig. 2b)^50^. The weak S-Cl bond in FSO_2_Cl with a highly reactive chloride renders a fast chloride atom transfer (*k* estimated >10^6 ^M^−1^s^−1^)^50,57^ from FSO_2_Cl to the radical intermediate **Int-A**; this rapid radical chain propagation (Path I) makes it difficult to trap this radical with other reagents or by single electron transfer (SET) oxidation to establish a photoredox reaction pathway (Path II). Given this challenging issue and also other limitations with FSO_2_Cl, such as: chlorination side-reactions and low/no yields with electron-rich substrates (as the chloride in FSO_2_Cl is highly electrophilic due to the electron-withdrawing effect of the FSO_2_-group)^50^, inconvenience in storage and handling due to the gaseous (b.p. 7 °C) and moisture-sensitive nature, the development of a new and convenient FSO_2_ radical precursor (X ≠ Cl) is highly desirable. Here, we report our efforts toward this goal, and the introduction of a solid-state, bench-stable type of reagents, 1-fluorosulfonyl 2-aryl benzoimidazolium triflate (FABI) salts, which can serve as effective redox-active FSO_2_ radical precursors and enable the development of radical fluorosulfonylation of olefins via a photoredox catalytic pathway. FABI is compatible with many substrates that were not compatible or low yielding when FSO_2_Cl is used, such as electron-rich alkenes and triaryl ethylenes. Moreover, a cascade alkoxy-fluorosulfonyl difunctionalization of olefins with FABI is presented, by trapping the postulated cationic intermediate **Int-B** with alcohols via a photoredox pathway (Fig. 2b, c).

**Table 1:** Reaction optimization for the radical fluorosulfonylation of styrene with FABI salts as a radical precursor^a^.


Entry	Radical Precursor	E_1/2_^red^ (V vs SCE)	Photocatalyst	Yield of 3aa^b^ (%)	*E*:*Z* of 3aa^c^
1	**2a**	−1.03	*fac*-Ir(ppy)_3_	0	–
2	**2b**	−1.08	*fac*-Ir(ppy)_3_	Trace	–
3	**2c**	−1.08	*fac*-Ir(ppy)_3_	Trace	–
4	**2d**	−1.09	*fac*-Ir(ppy)_3_	72	90:10
5	**2e**	−1.07	*fac*-Ir(ppy)_3_	94 (90)^d^	>20:1
6	**2e**	−1.07	4CzIPN	43	85:15
7	**2e**	−1.07	*/*	0	–
8	in dark	−1.07	*fac*-Ir(ppy)_3_	0	–

*LEDs* light-emitting diodes, *fac* facial, *ppy* 2-phenylpyridyl, *4CzIPN* 2,4,5,6-tetra(9H-carbazol-9-yl)isophthalonitrile.

^a^On 0.1 mmol scale.

^b^Determined by ^19^F NMR analysis using 4-fluoriodobenzene as an internal standard.

^c^Determined by ^19^F NMR.

^d^In parenthesis is isolated yield.

## Results

### Reaction optimization

We commenced our study with the screening of suitable FSO_2_ radical precursors in the form of imidazolium salts under photoredox conditions (Table 1). In the beginning, we tried a sample imidazolium salt, **2a**^47,58^, but it was found that **2a** was unable to generate the FSO_2_ radicals under this photoredox condition, delivering no any detectable formation of the desired product (entry 1). This is unexpected, as the excited *fac*-Ir(ppy)^3^ should be reducing enough (−1.73 V vs SCE) to reduce **2a** (−1.03 V vs SCE) via single electron transfer (SET). We guess the extrusion of FSO_2_ radicals after accepting one electron from excited *fac*-Ir(ppy)_3_ requires a good driving force of re-aromatization (for details, see the mechanistic discussion later). Therefore, we tested the imidazolium salt **2b** and **2c**^58^, with a 2-substitued or a fused phenyl group, respectively. Encouragingly, we could observe a trace amount of **3aa** (entry 2 and 3) Then, we combined the effects, and synthesized two 1-fluorosulfonyl 2-aryl benzoimidazolium triflate (FABI) salts: **2d** and **2e**. To our delight, **2d** afforded a substantial improvement in the reaction efficiency (entry 4), and the yield of the desired product **3aa** can be further improved to above 90% by using **2e** as the precursor, together with a high *E*/*Z* ratio (94%, entry 5). In this case, the desired product can be isolated in 90% yield. For more details about the reaction optimizations, please see the Supplementary Table 1 and 2. In addition, control experiments indicated that both the photocatalyst and light are crucial to the reaction (entry 7 and 8).

### Substrate scope

**Fig. 3:**
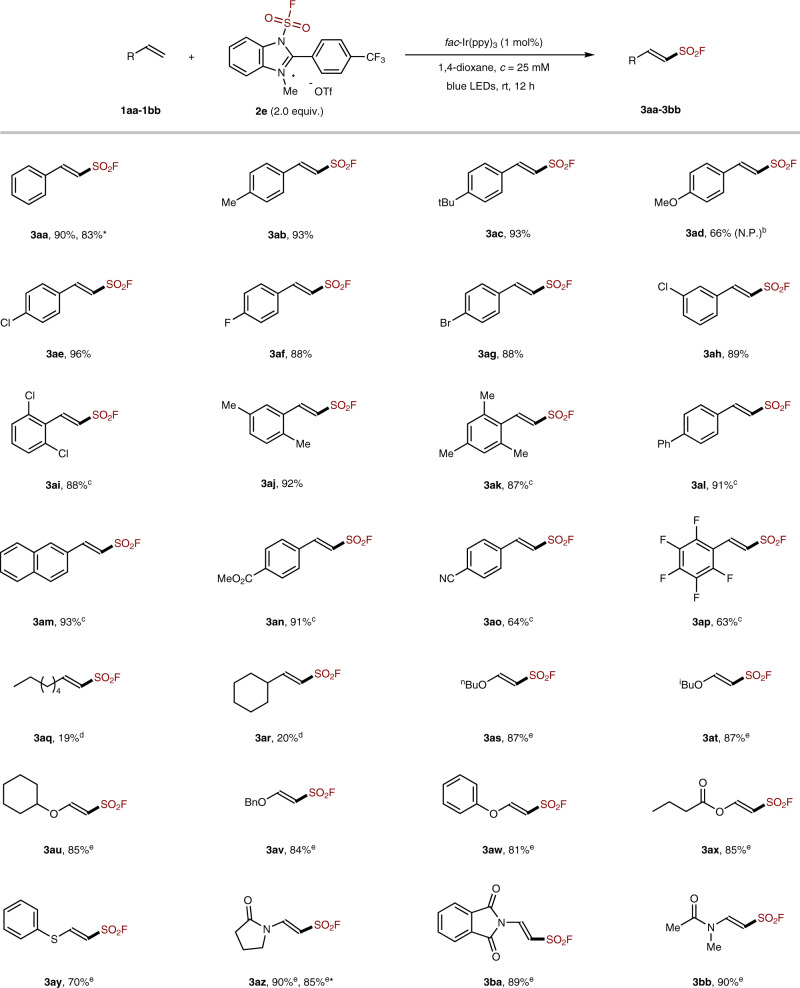
The scope of radical fluorosulfonylation of terminal olefins and electron-rich alkenes. The reactions were performed on 0.1 or 0.2 mmol scales, with 2 equiv. of **2e** in 1,4-dioxane at room temperature for 12 h. ^a^Yields on 1.0 mmol scale. ^b^The yields in parentheses are the reactions with FSO_2_Cl^50^. ^c^With 3.0 eq. of **2e**. ^d^With 1.0 eq. of K_3_PO_4_. ^e^With 1.0 eq. of K_2_CO_3_. *Yields on 1.0 mmol scale.

Having the optimized reaction conditions in hand, we moved on to investigate the reaction scope. As shown in Fig. 3, this protocol could readily accommodate a variety of styrenes (**3aa**-**3ap**) and showed a good tolerance of various functional groups, including halides (F, Cl, Br, **3ae**-**3ai**), ester (**3an**), and nitrile (**3ao**), etc. Notably, 4-methoxystyrene is compatible with the current conditions with FABI **2e**, the desired sulfonyl fluoride **3ad** can be obtained in 66% yield. In sharp contrast, the previous method with FSO_2_Cl as the FSO_2_ radical precursor afforded a messy reaction, and no desired product was obtained^50^. Aliphatic alkenes were less favored by this system (**3aq**-**3ar**), in line with the photoredox mechanism and the higher difficulty in oxidizing simple alkyl radicals than benzylic radicals^59^. Nevertheless, to our great pleasure, this FSO_2_ radical reagent (**2e**) is well compatible with electron-rich olefins, allowing for a facile access to β-*O*- or *N*-substituted vinyl sulfonyl fluorides (**3as**-**3bb**). As shown in Fig. 3, alkyl vinyl ethers (**3as**-**3av**), phenyl vinyl ether (**3aw**), vinyl acetates (**3ax**), vinyl thioether (**3ay**), and *N*-vinyl amides (**3az**-**3bb**), were all well tolerated, which further demonstrated the usefulness and advantages of FABI reagents over FSO_2_Cl.

**Fig. 4:**
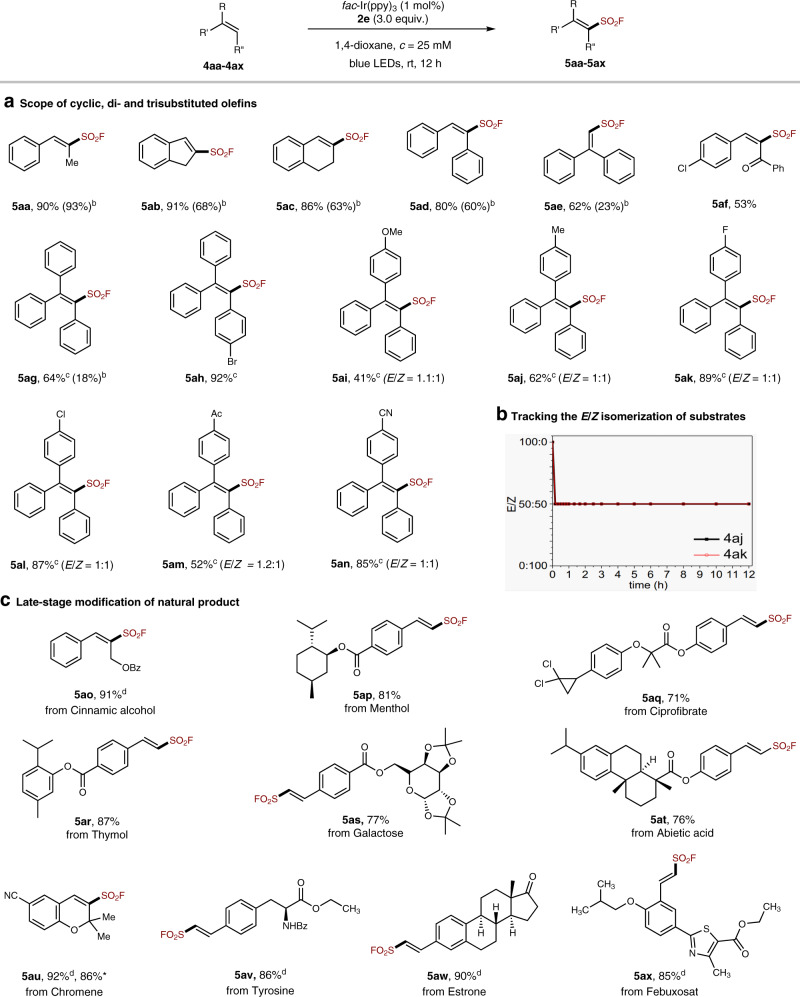
The scope of multi-substituted olefins and late-stage modification of natural products. **a** Scope of cyclic, di- and tri-substituted olefins. **b** Tracking the *E*/*Z* isomerization of substrate **4aj** and **4ak**. **c** Late-stage modification of natural products. ^a^All reactions were performed on 0.1 or 0.2 mmol scales . ^b^In parentheses are yields with FSO_2_Cl. ^c^With 1.0 eq. of K_2_CO_3_. ^d^With 2.0 eq. of **2e**. *Reactions on 1.0 mmol scale.

The direct radical fluorosulfonylation of cyclic, di- and tri-substituted olefins enables the preparation of multi-substituted vinyl sulfonyl fluorides. As shown in Fig. 4, the current protocol with FABI **2e** as the FSO_2_ radical reagent was found very effective for the diaryl and triaryl olefins (**4ad**, **4ae**, and **4ag**-**4an**), delivering the corresponding products in much higher yield than that of reactions with FSO_2_Cl. For example, indene and 1,2-dihydronaphthalene readily underwent the functionalization to give **5ab** and **5ac** in 91% and 86% yield with FABI **2e**, respectively, while the yields were 68% and 63% when using FSO_2_Cl as the radical precursor^50^. In the reactions of stilbene and 1,1-diphenylethylene, **2e** also exhibited good reactivity (Fig. 4, **5ae** and **5af**). The superiority of this reagent was further manifested in the direct fluorosulfonylation of triarylethylenes (**4ag**-**4an**), and the desired sulfonyl fluoride products (**5ag**-**5an**) can be obtained in good to high yields (41–92%). In contrast, the previous method with FSO_2_Cl gave **5ag** in a quite poor yield (18%)^50^. The low *E*/*Z* ratios in some cases probably resulted from the *E*/*Z* isomerization of the starting olefins, suggested by the tracking experiment with **4aj** and **4ak** (Fig. 4, b), in which both starting olefin **4aj & 4ak** was found rapidly isomerized into *Z*/*E* 1:1 ratio in 10 min. Further, more examples of triarylethylenes also afforded the products (**5ay**-**5ba**, in the Supplementary Methods) in ~1:1 Z/E ratios. Moreover, as shown in Fig. 4c, biorelevant molecules, such as cinnamic alcohol, menthol, ciprofibrate, thymol, galactose, abietic acid, chromene, tyrosine, estrone, and febuxosate-derived alkenes, can all be readily modified with this reagent, affording the corresponding sulfonyl fluorides (**5ao**-**5ax**) with a good functional group compatibility and high structural diversity.

**Fig. 5:**
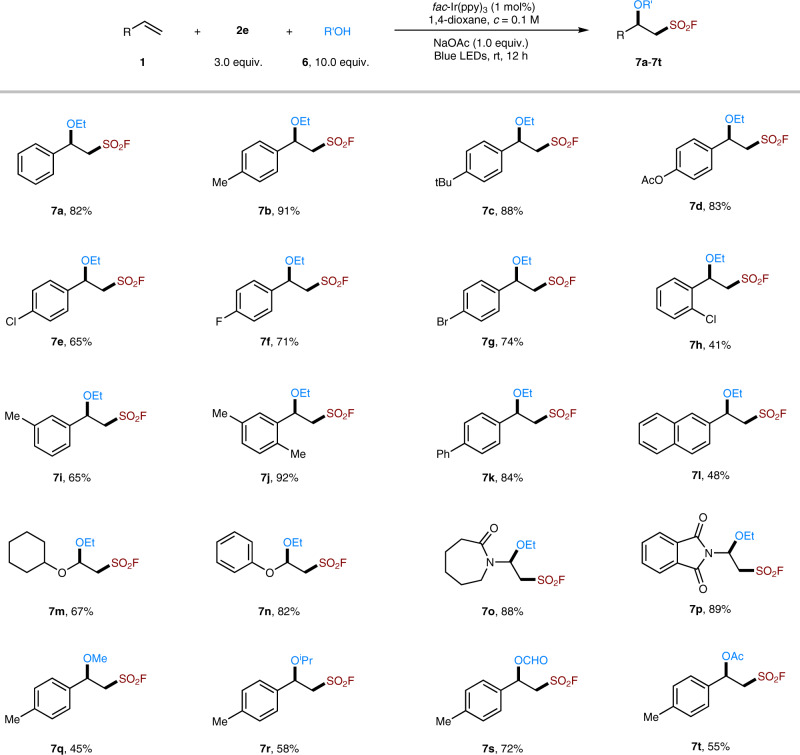
Photoredox alkoxy-fluorosulfonyl difunctionalization of olefins. The reactions were performed with alkene (0.1 mmol), **2e** (0.3 mmol), EtOH (1 mmol), *fac*-Ir(ppy)_3_ (1 mol%), NaOAc (0.1 mmol), and 1,4-dioxane (1 mL) under the irradiation of 6W blue LEDs.

To our delight, this reagent (**2e**) could finally allow the development of the alkoxy-fluorosulfonyl difunctionalization reaction of olefins with alcohols as a nucleophile. As shown in Fig. 5, in the presence of EtOH as nucleophile, this alkoxy-fluorosulfonyl difunctionalization protocol could readily accommodate a variety of styrenes (**7a**-**7l**) and electron-rich olefins (**7m**-**7p**). Reactions with other alcohols including methanol, isopropanol also proceeded well (**7q**, **7r**). Further, formic acid (**7** **s**) and acetic acid (**7t**) were also suitable nucleophiles, affording the corresponding ester product in 72% and 55% yield, respectively. It is worth mentioning that many β-hydoxy or alkoxyl sulfonic acids, sulfonamides, and related compounds were found showing various biological activity^60–62^, while the corresponding β-alkoxyl sulfonyl fluorides could server as precursors to access these molecules via SuFEx reactions^1–4^.

**Fig. 6:**
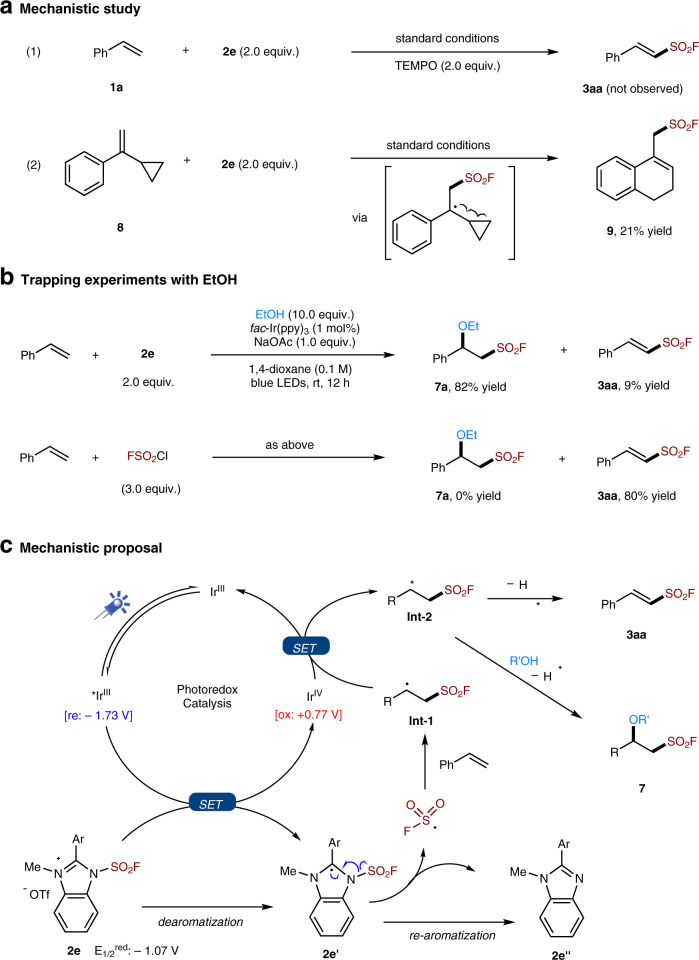
Mechanistic study and proposal. **a** Mechanistic study: (1) the reaction was performed under standard conditions with TEMPO (2.0 equiv.); (2) the reaction was performed under standard conditions with **8** as a radical probe. **b** Trapping experiments was performed with styrene (0.1 mmol), radical precursor (2.0–3.0 equiv.), EtOH (10.0 equiv.), *fac*-Ir(ppy)_3_ (1 mol%), NaOAc (0.1 mmol), and 1,4-dioxane (1 mL) under the irradiation of 6 W blue LEDs. **c** Mechanistic proposal for this photoredox radical fluorosulfonylation with FABI **2e**. TEMPO = 2,2,6,6-tetramethyl-1-piperidinoxyl.

To gain some mechanistic insight into the reaction, the radical scavenger 2,2,6,6-tetramethyl-1-piperidinoxyl (TEMPO, 2.0 equiv.) was added to the reaction mixture of **1a** and **2e** under standard conditions. The reaction was found completely inhibited, and no fluorosulfonylation product **3aa** was observed (Fig. 6 and Eq. 1). To further examine the involvement of FSO_2_ radical in the reaction, a radical-clock experiment was conducted with cyclopropylstyrene (**8**), a well-known radical probe^63,64^, and the cyclization product **9** can be isolated in 21% yield. This is in accord with a redox mechanism, and suggested that fluorosulfonyl radical addition to the double bond, followed by a subsequent radical ring-opening of the three-membered cycle and radical cyclization, should be involved (Fig. 6 and Eq. 2)^50,63,64^. On the other hand, as demonstrated in Fig. 5, carbocationic species can be trapped by alcohols. For comparison, we also performed the reaction with FSO_2_Cl, in which no formation of **7a** was observed under the same reaction conditions (Fig. 6b). In contrast, with FABI **2e** as the radical precursor, the desired ether product **7a** can be isolated in high yield, which further manifested the superiority of the newly developed FABI agents.

According to these results, a possible reaction mechanism for this radical fluorosulfonylation reaction using FABI **2e** as the radical precursor is proposed in Fig. 6c. First, under the irradiation of blue LEDs, the photocatalyst (Ir^III^) is excited (E_1/2_^IV/III*^ = −1.73 V vs SCE)^65^ and then undergoes a single electron transfer (SET) to the redox-active radical precursor **2e** (E_1/2_^red^ = −1.07 V vs SCE). Upon the acceptance of one electron, **2e′** would undergo a homolytic fission of the N-S bond, and give the desired FSO_2_ radicals. Subsequently, the addition of FSO_2_• to styrene furnishes the key radical intermediate **Int-1**. Oxidation of **Int-1** by Ir^IV^ affords the cationic species **Int-2**, which can be deprotonated to give **3aa**, while trapping **Int-2** with alcohols (R’OH) could afford the difunctionalization product **7**. It is worth mentioning that when FSO_2_Cl was used as FSO_2_• precursor, it was found unable to establish this redox cascade difunctionalization reaction as shown in Fig. 6B, probably due to the fast radical chain mechanism (Fig. 2B, path I) preventing the SET oxidation of the radical intermediate **Int-1**^50,51^. Further, considering the other reagents **2a**-**c** have similar redox potentials (*E*_1/2_^red^ = −1.03−1.09 V vs SCE, see the Supplementary Information) as **2e**, the presence of both a benzo-moiety and 2-aryl group in the reagents (FABI **2d** and **2e**) could probably facilitate the extrusion of the desired FSO_2_ radicals by enhancing the driving force of re-aromatization in the step from **2e′** to **2e″**.

## Discussion

In summary, 1-fluorosulfonyl benzoimidazolium triflate (FABI) salts have been demonstrated as an effective redox-active fluorosulfonyl radical precursor, featuring its solid state, bench-stable characters, convenience to handle, and good tolerance of functional groups. This radical fluorosulfonylation method allows for a facile access to various vinyl sulfonyl fluorides from olefinic substrates, with remarkable good compatibility to electron-rich substrates and triaryl ethylenes, in comparison with the methods established with the known FSO_2_ radical precursor. In particular, FABI could allow the radical fluorosulfonylation proceding through a photoredox pathway and thus make it possible to develop the alkoxy-fluorosulfonylation reaction of olefins via trapping the carbocationic intermediates. We expect that the FABI reagents^66^ serving as a convenient and effective radical precursor would bring about the design and development of many radical fluorosulfonylation reactions, and further benefit the related study in the context of chemical biology and drug discovery in the future.

## Methods

### General procedure

The *fac*-Ir(ppy)_3_ (1 mol%) and FABI **2e** (2.0 or 3.0 equivalents) were weighed into an oven-dried Schlenk tube, followed by the addition of anhydrous 1,4-dioxane (4.0 mL, 0.025 M) and olefin substrate (0.1 mmol) under argon. The reaction mixture was allowed to stir at room temperature under irradiation with blue LEDs for 12 h. Purification by column chromatography or preparative thin-layer chromatography on silica gel gave the desired pure product. Full experimental details and characterization of new compounds can be found in the Supplementary Methods and Supplementary Figs. 11–238.

## Supplementary information

Supplementary Information

## Data Availability

The authors declare that all data generated in this study are provided in the article and the Supplementary Information file, and are also available from the corresponding author upon request.
