# Supplementary material for: Photoredox catalytic radical fluorosulfonylation of olefins enabled by a bench-stable redox-active fluorosulfonyl radical precursor
Source: Nat Commun. 2022 Jun 11;13:3370. doi: 10.1038/s41467-022-31089-7 (PMC9188602; doi:10.1038/s41467-022-31089-7)
Supplement: Supplementary file 1 — Supplementary Information [file 41467_2022_31089_MOESM1_ESM.pdf]

Supplementary Information

Photoredox catalytic radical fluorosulfonylation of olefins enabled by a bench-stable redox-active fluorosulfonyl radical precursor

Wang *et al.*

Supplementary Methods

General procedures. Solvents and reagents were bought from Sigma-Aldrich, J&K, Alfa-Aesar, Energy and TCI chemicals, and used directly without further purification. Reactions were monitored by thin layer chromatography (TLC) using silica gel 60 F-254 plates. TLC plates were normally visualized by UV irradiation (254 nm or 365 nm), stained with basic $KMnO_4$. Flash chromatography was performed using silica gel 60 (200-300 mesh).

Instrumentation. Proton nuclear magnetic resonance (^{1}H NMR) spectra, fluorine nuclear magnetic resonance (^{19}F NMR) spectra and carbon nuclear magnetic resonance (^{13}C NMR) spectra were recorded on Bruker Ascend 400 MHz or JEDL ECX-500 MHz. Chemical shifts for protons are reported in parts per million downfield from tetramethylsilane and are referenced to the NMR solvent residual peak ($CDCl_3$: *δ* 7.26, CD_3CN: *δ* 1.94 or DMSO-d_6: *δ* 2.50). Chemical shifts for carbons are reported in parts per million downfield from tetramethylsilane and are referenced to the carbon resonances of the NMR solvent ($CDCl_3$: *δ* 77.0, CD_3CN: *δ* 1.32 and 118.26 or DMSO-d_6: *δ* 39.52). Data are represented as follows: chemical shift, multiplicity (s = singlet, d = doublet, t = triplet, q = quartet, m = multiplet, br = broad), coupling constants in Hertz (Hz), and integration. High resolution mass spectra (HRMS) were recorded on a Thermo Fisher Scientific Exactive Plus by Electrospray Ionisation (ESI) or Atmospheric Pressure Photo-Ionization(APCI). GC-MS measurements were performed on a SHIMADZU GCMS-QP2010 SE. Unless otherwise stated, the alkenes and alkynes were purchased from commercial sources. Cyclic voltammetry data was measured with a CHI 760E potentiostat (Chinstruments).

Abbreviations. LED–light emitting diode; TLC–thin layer chromatography; PE–Petroleum Ethers; THF–tetrahydrofuran; DMF–N,N-dimethylformamide; DMSO–dimethyl sulfoxide

Photoreaction Setup. All manipulations for the radical fluorosulfonylation via photoredox reactions were set up in a 20 mL Schlenk Tubes (unless otherwise noted) under an inert Argon atmosphere using glove-box techniques. The reactions were conducted in photo-reactors (Model:H106062, GEAO CHEMICAL, purchased from http://www.geaochem.com/), which comprise a fan for cooling (approximately room temperature) and six 1W blue LED beads for each place. The average power output of the photo-reactor was recorded at 30 mW/cm^2. One place/hole with three 1W blue LED beads (λ_{max} = 460 nm) on both side, and the distance between the Schlenk tube and LED beads is ca. 1 cm. The emission spectra of the blue LEDs were recorded on an Ocean Optics HR4000CG-UVNIR spectrometer. The spectra was normalised to 1.0 at the maximum (450 nm).

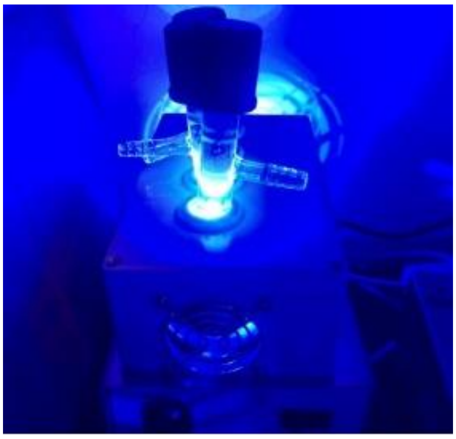

Supplementary Figure 1. Photo-reactor and reaction setup. With blue LEDs.

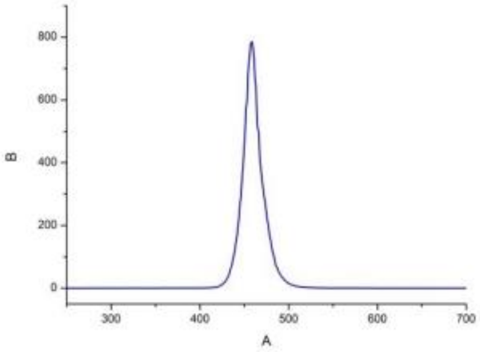

Supplementary Figure 2. The light emission spectrum of photo-reactor. Blue LEDs.

Synthesis of Imidazolium Salt Reagents. Fluorosulfuryl imidazolium salt reagents were synthesized according to the literature with improved procedures.[1-3]

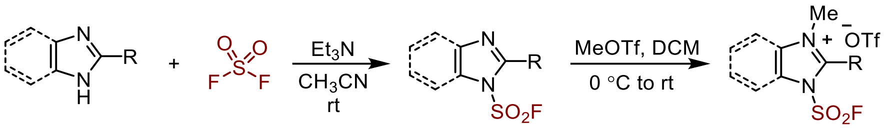

1) To a one-necked 1 L flask equipped with a magnetic stirrer, the corresponding imidazole (100 mmol), NEt_3 (20.8 mL, 150 mmol) and 600 mL CH_3CN were added. The flask was sealed with a rubber septum and the air in the flask was evacuated under gentle vacuum with a water pump until the solution started to degas. Sulfuryl fluoride gas (3 L, approx. 122 mmol) was introduced into the flask using a balloon and a needle, and the reaction mixture was stirred at room temperature overnight, while monitoring by TLC (PE : EA = 2:1). After completion, the reaction mixture was evaporated in vacuo. Then, the reaction mixture was quenched with water and extracted with ethyl acetate (300 mL × 3). The combined organic layers were dried over Na_2SO_4, filtered and concentrated. When R = Me, the crude product can be directly used in the next step without further purification. When R = Ph, the product was purified by flash column chromatography on silica gel with PE/EA as eluent to give the corresponding intermediate.

2) To a one-necked 1L flask equipped with a magnetic stirrer, the product mentioned above and dichloromethane (500 mL) was added, followed by methyl trifluoromethanesulfonate (13.6 mL, 120 mmol, 1.5 equiv.) was added dropwise at 0 ℃ under Ar. The mixture was allowed to warm to room temperature and stirred for 4 hours, while monitoring by TLC (PE/EA = 2:1). After that time, the mixture was concentrated under rotary evaporation to give a white solid (or a viscous liquid) crude product, to which methyl tertbutyl ether (500 mL) was added. With vigorous stirring, a solid precipitate was formed. The precipitate was washed with methyl tertbutyl ether (200 mL × 3) and dried in vacuo to yield the title compound (**2a** - **2c**) as white solid.

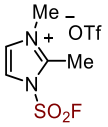

1-(Fluorosulfuryl)-2,3-dimethyl-1H-imidazol-3-ium trifluoromethanesulfonate (**2a**): 26.75 g, 90% yield over two steps. **2a** is a known compound and the characterization data are in accordance with the literature.[1]

^{1}**H NMR** (500 MHz, CD_3CN) δ 7.55 (d, J = 2.5 Hz, 1H), 7.51 (d, J = 2.5 Hz, 1H), 3.73 (s, 3H), 2.55 (s, 3H);

^{19}**F NMR** (471 MHz, CD_3CN) δ 39.7 (s, 1F), -78.1 (s, 3F).

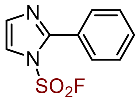

2-Phenyl-1H-imidazole-1-sulfonyl fluoride (S1):

9.28 g, 41% yield, white solid.

^{1}**H NMR** (400 MHz, $CDCl_3$) δ 7.74 (d, J = 7.0 Hz, 2H), 7.63 – 7.45 (m, 4H), 7.28 – 7.22 (m, 1H);

^{19}**F NMR** (376 MHz, $CDCl_3$) δ 61.3;

^{13}**C NMR** (101 MHz, $CDCl_3$) δ 148.9, 130.7, 129.7, 129.5, 128.3, 128.1, 121.4;

HRMS (ESI): m/z calcd. for $C_9H_7FN_2NaO_2S$ $[M+Na]^+$ 249.0104, found 249.0109.

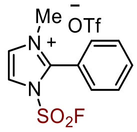

1-(Fluorosulfonyl)-3-methyl-2-phenyl-1H-imidazol-3-ium trifluoromethanesulfonate (2b):

14.88 g, 96% yield, white solid, 98–99 ˚C.

^{1}**H NMR** (400 MHz, CD_3CN) δ 8.28 (s, 1H), 8.08 – 7.95 (m, 2H), 7.98 – 7.80 (m, 4H), 3.90 (s, 3H);

^{19}**F NMR** (376 MHz, CD_3CN) δ 64.6 (s, 1F), -78.1 (s, 3F);

^{13}**C NMR** (101 MHz, CD_3CN) δ 149.3, 135.0, 131.5, 130.5, 126.1, 123.6, 120.2, 38.1;

HRMS (DART): m/z calcd. for $C_{10}H_{10}FN_2O_2S^+$ $[M]^+$ 241.0442, found 241.0448; **HRMS (DART):** m/z calcd for $CO_3F_3S^-$ $[M]^-$ 148.9526, found 148.9529.

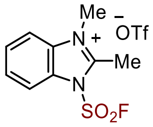

1-(Fluorosulfonyl)-2-methyl-1H-benzo[d]imidazol-3-ium trifluoromethanesulfonate (**2c**):

33.66 g, 89% yield over two steps, white solid, m.p. 173–174 ˚C.

^{1}**H NMR** (400 MHz, DMSO-d_6) δ 7.94 (d, J = 7.6 Hz, 1H), 7.86 – 7.76 (m, 2H), 7.64 – 7.52 (m, 2H), 3.94 (s, 3H), 2.82 (s, 3H);

^{19}**F NMR** (376 MHz, DMSO-d_6) δ 39.7 (s, 1F), -78.1 (s, 3F);

^{13}**C NMR** (101 MHz, DMSO-d_6) δ 151.9, 132.4, 129.5, 125.9, 125.4, 113.7, 112.6, 31.0, 11.6; **HRMS (DART):** m/z calcd. for $C_9H_{10}FN_2O_2S^+$ [M]$^+$ 229.0442, found 229.0449; **HRMS (DART):** m/z calcd for $CO_3F_3S^-$ [M]$^-$ 148.9526, found 148.9527.

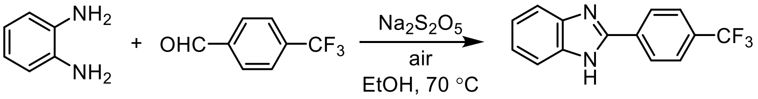

1) According to a modified literature procedure,[4] a mixture of *o*-phenylenediamine (22 g, 200 mmol), $Na_2S_2O_5$ (14.5 g, 150 mmol), and 300 mL ethanol was stirred at 70 ℃ in a 1 L round-bottomed flask under air atmosphere, followed by the 4-(trifluoromethyl)benzaldehyde (13.6 mL, 100 mmol) was added dropwise to a solution. After completion of the reaction, the reaction mixture was cooled, water was added, and the mixture was allowed to stir in 0 ℃ to obtain a precipitate. Then, the obtained crude solid benzimidazole compounds can be directly used in the next step after drying without further purification.

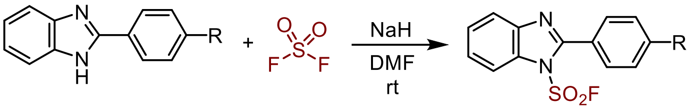

S2, R = H;
S3, R = CF_3

2) To a one-necked 500 mL flask equipped with a magnetic stirrer, the corresponding imidazole (50 mmol), and 200 mL DMF were added. Then 60 percent sodium hydride in mineral oil (2.4 g, 60 mmol) was added to the mixture portionwise at 0 °C. The mixture was allowed to warm to room temperature and stirred for 2 hours. The air in the flask was evacuated under gentle vacuum with a water pump until the solution started to degas. Sulfuryl fluoride gas (1.5 L, approx. 61 mmol) was introduced into the flask using a balloon and a needle, and the reaction mixture was stirred at room temperature overnight, while monitoring by TLC (PE : EA = 10:1). After completion, the reaction mixture was quenched with water and extracted with ethyl acetate (200 mL × 3), then the combined organic layers were dried over Na_2SO_4, filtered and concentrated in vacuo. The product was purified by flash column chromatography on silica gel with PE/EA as eluent to give the corresponding intermediate.

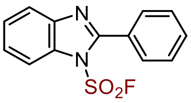

2-Phenyl-1H-benzo[d]imidazole-1-sulfonyl fluoride (S2):

12.01 g, 87% yield, white solid.

^{1}**H NMR** (500 MHz, $CDCl_3$) δ 7.89 – 7.76 (m, 2H), 7.77 – 7.69 (m, 2H), 7.55 – 7.43 (m, 3H), 7.41 – 7.31 (m, 2H);

^{19}**F NMR** (471 MHz, $CDCl_3$) δ 60.8;

^{13}**C NMR** (126 MHz, $CDCl_3$) δ 152.0, 141.6 (d, J = 1.8 Hz), 132.6, 130.7, 129.5, 128.2, 127.9, 126.0, 125.9, 120.7, 113.6;

HRMS (ESI): *m*/*z* calcd. for $C_{13}H_9FN_2NaO_2S$ $[M+Na]^+$ 299.0261, found 299.0266.

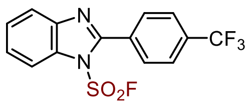

2-(4-(Trifluoromethyl)phenyl)-1H-benzo[d]imidazole-1-sulfonyl fluoride (S3):

14.29 g, 83% yield over two steps, white solid.

^{1}**H NMR** (500 MHz, $CDCl_3$) δ 7.95 – 7.91 (m, 1H), 7.91 – 7.85 (m, 3H), 7.72 – 7.67 (m, 2H), 7.58 – 7.51 (m, 2H);

^{19}**F NMR** (471 MHz, $CDCl_3$) δ 60.7 (s, 1F), -62.9 (s, 3F);

^{13}**C NMR** (126 MHz, $CDCl_3$) δ 150.9, 142.0 (d, J = 1.3 Hz), 133.2 (q, J = 32.8 Hz), 133.1, 132.3 (q, J = 2.5 Hz), 130.5, 127.1, 126.8, 125.5 (q, J = 4.0 Hz), 123.8 (q, J = 273.4 Hz), 121.5, 114.2;

HRMS (ESI): *m*/*z* calcd. for $C_{14}H_8F_4N_2NaO_2S$ $[M+Na]^+$ 367.0135, found 367.0133.

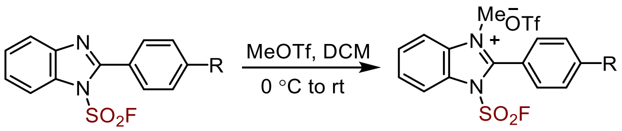

3) To a one-necked 1 L flask equipped with a magnetic stirrer, **S2** or **S3** and dichloromethane (300 mL) was added, followed by methyl trifluoromethanesulfonate (6.8 mL, 60 mmol) added dropwise at 0 °C under Ar. The mixture was allowed to warm to room temperature and stirred for 12 hours, while monitoring by TLC (PE : EA = 10:1). After that time, the mixture was concentrated under rotary evaporation to give a white solid crude product, to which methyl tertbutyl ether (300 mL) was added. and a solid precipitate was formed. The precipitate was washed with methyl tertbutyl ether (200 mL × 3) and dried in vacuo to yield the title compound (**2d**, **2e**) as white solids.

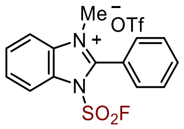

1-(Fluorosulfonyl)-2-phenyl-1H-benzo[d]imidazol-3-ium trifluoromethanesulfonate (**2d**):

18.38 g, 96% yield, white solid, m.p. 163–164 ˚C.

^{1}H NMR (500 MHz, DMSO-d_6) δ 8.12 – 8.08 (m, 1H), 8.00 – 7.95 (m, 2H), 7.91 – 7.88 (m, 1H), 7.85 – 7.77 (m, 3H), 7.73 – 7.65 (m, 2H), 4.05 (s, 3H);

^{19}F NMR (471 MHz, DMSO-d_6) δ 39.7 (s, 1F), -78.1 (s, 3F);

^{13}C NMR (101 MHz, DMSO-d_6) δ 150.5, 133.6, 133.5, 131.1, 130.8, 129.9, 127.2, 126.6, 122.9, 114.6, 113.9, 33.2;

HRMS (DART): *m/z* calcd. for $C_{14}H_{12}FN_2O_2S^+$ [M]$^+$ 291.0598, found 291.0601; **HRMS (DART):** m/z calcd for $CO_3F_3S^-$ [M]$^-$ 148.9526, found 148.9528.

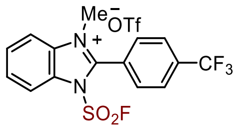

1-(Fluorosulfonyl)-2-(4-(trifluoromethyl)phenyl)-1H-benzo[d]imidazol-3-ium trifluoromethanesulfonate (**2e**):

3.89 g, 95% yield, white solid, m.p. 175–176 ˚C.

^{1}H NMR (500 MHz, CD_3CN) δ 8.19 (d, J = 8.0 Hz, 1H), 8.14 – 8.04 (m, 5H), 8.00 – 7.90 (m, 2H), 3.94 (s, 3H);

^{19}F NMR (471 MHz, CD_3CN) δ 39.7 (s, 1F), -62.0 (s, 3F), -78.1 (s, 3F);

^{13}C NMR (101 MHz, CD_3CN) δ 152.5, 135.9 (q, J = 33.1 Hz), 132.6, 131.9, 130.7, 130.6, 127.6 (q, J = 4.0 Hz), 124.8 (q, J = 1.5 Hz), 124.5 (d, J = 273.4 Hz), 118.4, 116.1, 115.9, 35.6;

HRMS (DART): *m/z* calcd. for $C_{15}H_{11}F_4N_2O_2S^+$ [M]$^+$ 359.0472, found 359.0477; **HRMS (DART):** m/z calcd for $CO_3F_3S^-$ [M]$^-$ 148.9526, found 148.9522.

Cyclic voltammetry studies. Measured for all the imidazonium reagents, and the cyclic voltammograms are shown below.

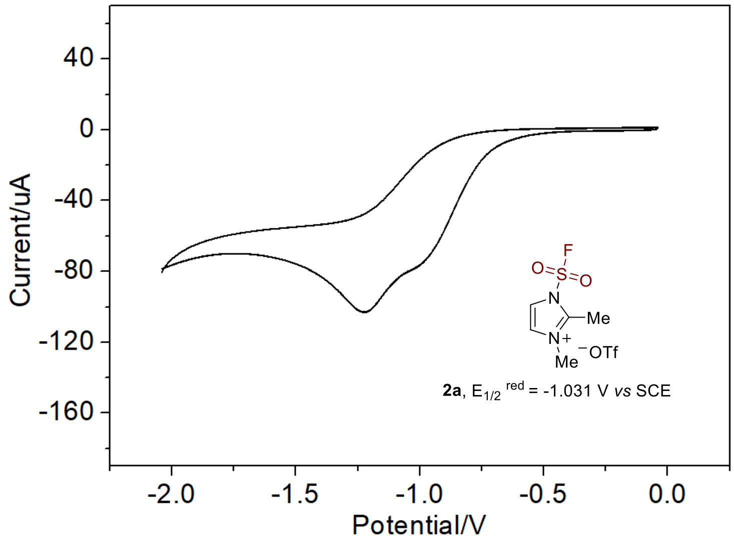

Supplementary Figure 3. Cyclic voltammogram of **2a**.

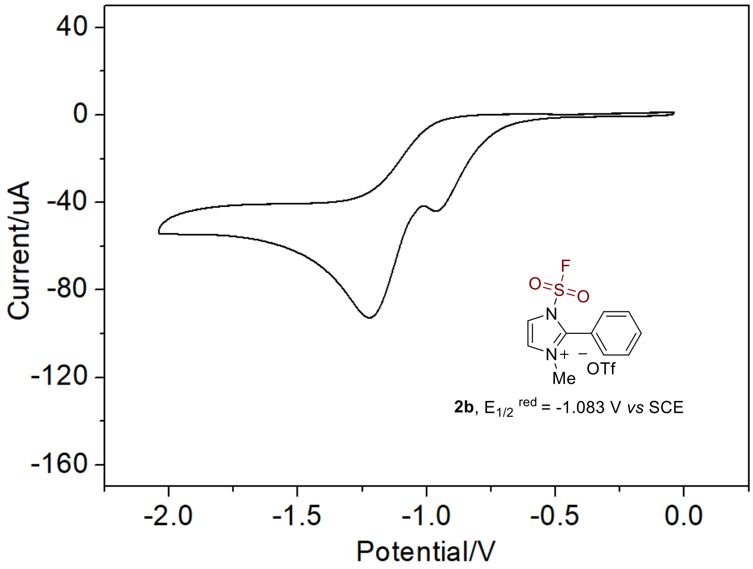

Supplementary Figure 4. Cyclic voltammogram of **2b**.

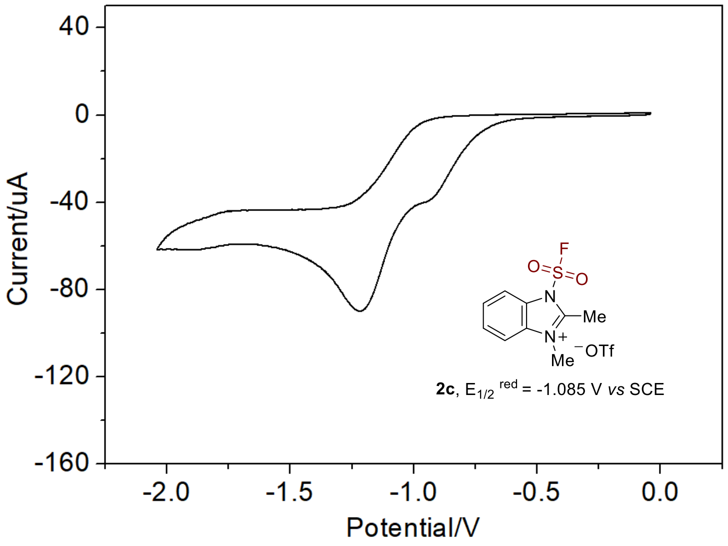

Supplementary Figure 5. Cyclic voltammogram of **2c**.

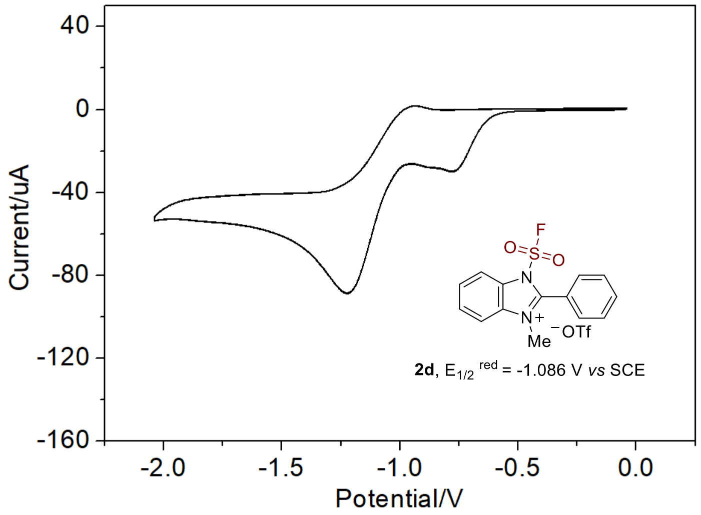

Supplementary Figure 6. Cyclic voltammogram of **2d**.

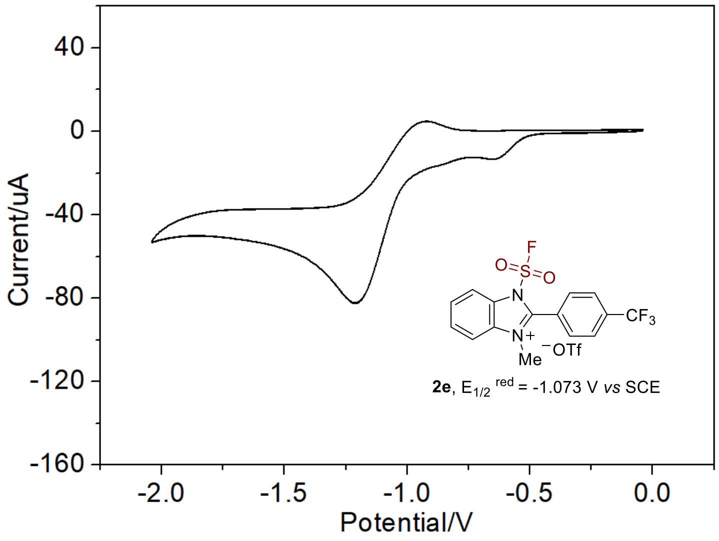

Supplementary Figure 7. Cyclic voltammogram of **2e**.

General procedure for the syntheses of 3 and 5 (Procedure A). The *fac*-Ir(ppy)$_3$ (0.65 mg, 0.001 mmol, 1 mol%) and FABI 2e were weighed into an oven-dried Schlenk tube, followed by the addition of anhydrous 1,4-dioxane (4.0 mL, 0.025 M) and olefin substrate (0.1 mmol) under argon. The reaction mixture was allowed to stir at room temperature under irradiation with blue LEDs for 12 h. Purification by column chromatography or preparative thin layer chromatography on silica gel gave the desired pure product. Photo-induced reactions were conducted in photo-reactors, which comprise a fan for cooling (approximately room temperature) and six 1W blue LED beads for each place (6 W). The average power output of the photo-reactor was ca. 30 mW/cm^2. The emission spectra of the blue LEDs were recorded on an Ocean Optics HR4000CG-UVNIR spectrometer.[5]

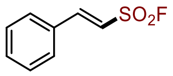

(*E*)-2-Phenylethene-1-sulfonyl fluoride (3aa):
17 mg, 90% yield, white solid, the characterization data are in accordance with the literature.[5]
^{1}H NMR (500 MHz, CDCl$_3$) δ 7.82 (d, J = 15.5 Hz, 1H), 7.59 – 7.50 (m, 3H), 7.50 – 7.42 (m, 2H), 6.88 (dd, J = 15.5, 2.5 Hz, 1H);
^{19}F NMR (471 MHz, CDCl$_3$) δ 62.4.

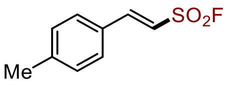

(*E*)-2-(P-tolyl)ethene-1-sulfonyl fluoride (3ab)：

19 mg, 93% yield, white solid, the characterization data are in accordance with the literature.[5]
^{1}H NMR (500 MHz, CDCl$_3$) δ 7.78 (d, J = 16.0 Hz, 1H), 7.47 – 7.42 (m, 2H), 7.29 – 7.26 (m, 2H), 6.80 (dd, J = 15.5 Hz, 2.5 Hz, 1H), 2.42 (s, 3H);
^{19}F NMR (471 MHz, CDCl$_3$) δ 62.7.

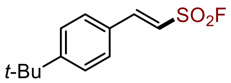

(*E*)-2-(4-(*tert*-Butyl)phenyl)ethene-1-sulfonyl fluoride (3ac):
23 mg, 93% yield, white solid, the characterization data are in accordance with the literature.[5]
^{1}H NMR (500 MHz, CDCl$_3$) δ 7.79 (d, J = 15.5 Hz, 1H), 7.49 (s, 4H), 6.82 (dd, J = 15.5, 2.5 Hz, 1H), 1.34 (s, 9H);
^{19}F NMR (471 MHz, CDCl$_3$) δ 62.7.

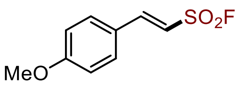

(*E*)-2-(4-Methoxyphenyl)ethene-1-sulfonyl fluoride (3ad)：

14 mg, 66% yield, white solid, m.p. 82–83 ˚C.

^{1}H NMR (500 MHz, $CDCl_3$) δ 7.75 (d, J = 15.5 Hz, 1H), 7.54 – 7.48 (m, 2H), 6.99 – 6.93 (m, 2H), 6.70 (dd, J = 16.0 Hz, 3.0 Hz, 1H), 3.88 (s, 3H);

^{19}F NMR (471 MHz, $CDCl_3$) δ 63.1;

^{13}C NMR (101 MHz, $CDCl_3$) δ 163.2, 148.7 (d, J = 2.5 Hz), 131.1, 123.6, 114.8, 114.6 (d, J = 34.0 Hz), 55.6;

HRMS (EI) *m/z* calcd. for $C_9H_9FO_3S$ $[M]^+$ 216.0256, found 216.0259.

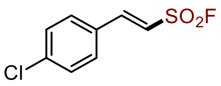

(*E*)-2-(4-Chlorophenyl)ethene-1-sulfonyl fluoride (3ae):

21 mg, 96% yield, white solid, the characterization data are in accordance with the literature.[5]

^{1}H NMR (500 MHz, $CDCl_3$) δ 7.77 (d, J = 15.5 Hz, 1H), 7.53 – 7.48 (m, 2H), 7.48 – 7.42 (m, 2H), 6.85 (dd, J = 15.5, 2.5 Hz, 1H);

^{19}F NMR (471 MHz, $CDCl_3$) δ 62.5.

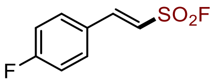

(*E*)-2-(4-Fluorophenyl)ethene-1-sulfonyl fluoride (3af)：

18 mg, 88% yield, white solid, the characterization data are in accordance with the literature.[5]

^{1}H NMR (500 MHz, $CDCl_3$) δ 7.78 (d, J = 15.5 Hz, 1H), 7.60 – 7.55 (m, 2H), 7.21 – 7.14 (m, 2H), 6.81 (dd, J = 15.5 Hz, 2.5 Hz, 1H);

^{19}F NMR (471 MHz, $CDCl_3$) δ 62.5 (s, 1F), -104.9 (s, 1F).

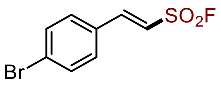

(*E*)-2-(4-Bromophenyl)ethene-1-sulfonyl fluoride (3ag)：

23 mg, 88% yield, white solid, the characterization data are in accordance with the literature.[5]

^{1}H NMR (400 MHz, $CDCl_3$) δ 7.75 (d, J = 15.2 Hz, 1H), 7.62 (d, J = 8.4 Hz, 2H), 7.42 (d, J = 8.4 Hz, 2H), 6.87 (dd, J = 15.2, 2.8 Hz, 1H);

^{19}**F NMR** (376 MHz, CDCl$_3$) δ 62.3.

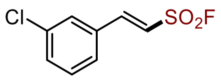

(*E*)-2-(3-Chlorophenyl)ethene-1-sulfonyl fluoride (3ah)：

18 mg, 89% yield, white solid, the characterization data are in accordance with the literature.[5]
^{1}**H NMR** (400 MHz, CDCl$_3$) δ 7.75 (d, J = 15.6 Hz, 1H), 7.58 – 7.53 (m, 1H), 7.52 – 7.46 (m, 1H), 7.46 – 7.39 (m, 2H), 6.89 (dd, J = 15.6, 2.8 Hz, 1H);
^{19}**F NMR** (376 MHz, CDCl$_3$) δ 62.2.

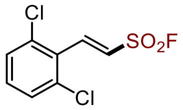

(*E*)-2-(2,6-Dichlorophenyl)ethene-1-sulfonyl fluoride (3ai)：

21 mg, 88% yield, white solid, the characterization data are in accordance with the literature.[5]
^{1}**H NMR** (400 MHz, CDCl$_3$) δ 8.04 (d, J = 15.6 Hz, 1H), 7.44 (d, J = 8.4 Hz, 2H), 7.36 – 7.26 (m, 2H);
^{19}**F NMR** (376 MHz, CDCl$_3$) δ 61.3.

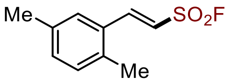

(*E*)-2-(2,5-Dimethylphenyl)ethene-1-sulfonyl fluoride (3aj)：

20 mg, 92% yield, white solid, the characterization data are in accordance with the literature.[5]
^{1}**H NMR** (400 MHz, CDCl$_3$) δ 8.07 (d, J = 15.2 Hz, 1H), 7.34 (s, 1H), 7.22 – 7.15 (m, 2H), 6.79 (dd, J = 15.2, 2.4 Hz, 1H), 2.42 (s, 3H), 2.35 (s, 3H);
^{19}**F NMR** (376 MHz, CDCl$_3$) δ 62.2.

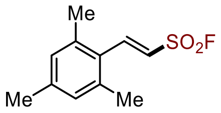

(*E*)-2-Mesitylethene-1-sulfonyl fluoride (3ak)：

20 mg, 87% yield, white solid, the characterization data are in accordance with the literature.[5]
^{1}**H NMR** (500 MHz, CDCl$_3$) δ 8.02 (d, J = 16.0 Hz, 1H), 6.94 (s, 2H), 6.58 (dd, J = 16.0 Hz, 2.5 Hz, 1H), 2.38 (s, 6H), 2.31 (s, 3H);

^{19}F NMR (471 MHz, $CDCl_3$) δ 62.0.

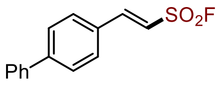

(*E*)-2-([1,1'-Biphenyl]-4-yl)ethene-1-sulfonyl fluoride (3al)：

24 mg, 91% yield, white solid, the characterization data are in accordance with the literature.[5]
^{1}H NMR (400 MHz, $CDCl_3$) δ 7.85 (d, J = 15.6 Hz, 1H), 7.74 – 7.67 (m, 2H), 7.66 – 7.57 (m, 4H), 7.52 – 7.45 (m, 2H), 7.45 – 7.37 (m, 1H), 6.89 (dd, J = 15.6, 2.4 Hz, 1H);
^{19}F NMR (376 MHz, $CDCl_3$) δ 62.6.

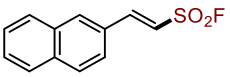

(*E*)-2-(Naphthalen-2-yl)ethene-1-sulfonyl fluoride (3am)：

22 mg, 93% yield, white solid, the characterization data are in accordance with the literature.[5]
^{1}H NMR (400 MHz, $CDCl_3$) δ 8.02 – 7.85 (m, 5H), 7.66 – 7.49 (m, 3H), 6.96 (dd, J = 15.6, 2.4 Hz, 1H);
^{19}F NMR (376 MHz, $CDCl_3$) δ 62.7.

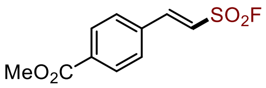

Methyl (*E*)-4-(2-(fluorosulfonyl)vinyl)benzoate (3an)：

22 mg, 91% yield, white solid, the characterization data are in accordance with the literature.[5]
^{1}H NMR (400 MHz, $CDCl_3$) δ 8.13 (d, J = 8.0 Hz, 2H), 7.84 (d, J = 15.6 Hz, 1H), 7.64 (d, J = 8.0 Hz, 2H), 6.97 (dd, J = 15.6, 2.4 Hz, 1H), 3.96 (s, 3H);
^{19}F NMR (376 MHz, $CDCl_3$) δ 62.1.

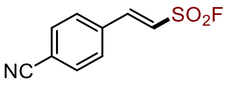

(*E*)-2-(4-Cyanophenyl)ethene-1-sulfonyl fluoride (3ao)：

14 mg, 64% yield, white solid, the characterization data are in accordance with the literature.[5]
^{1}H NMR (400 MHz, $CDCl_3$) δ 7.84 – 7.77 (m, 3H), 7.68 (d, J = 8.0 Hz, 2H), 6.99 (dd, J = 15.6, 2.4 Hz, 1H);
^{19}F NMR (376 MHz, $CDCl_3$) δ 62.1.

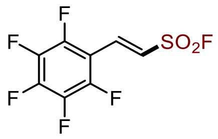

(*E*)-2-(Perfluorophenyl)ethene-1-sulfonyl fluoride (3ap)：

17 mg, 63% yield, colorless oil, the characterization data are in accordance with the literature.[5]

^{1}H NMR (400 MHz, $CDCl_3$) δ 7.81 (d, J = 16.0 Hz, 1H), 7.26 (dd, J = 16.0 Hz, 2.4 Hz, 1H);

^{19}F NMR (471 MHz, $CDCl_3$) δ 61.4 (s, 1F), -136.4 – -137.3 (m, 2F), -144.9 – -145.6 (m, 1F), -158.9 – -159.9 (m, 2F).

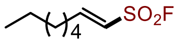

(*E*)-Pent-1-ene-1-sulfonyl fluoride (3aq)：

12 mg, 19% yield, on 0.8 mmol scale, colorless oil, the characterization data are in accordance with the literature.[5]

^{1}H NMR (400 MHz, $CDCl_3$) δ 7.20 (dt, J = 15.2, 6.8 Hz, 1H), 6.37 (d, J = 15.2 Hz, 1H), 2.38 – 2.33 (m, 2H), 1.56 – 1.49 (m, 2H), 1.39 – 1.28 (m, 6H), 0.90 (t, J = 6.4 Hz, 3H);

^{19}F NMR (471 MHz, $CDCl_3$) δ 59.8.

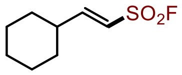

(*E*)-2-Cyclohexylethene-1-sulfonyl fluoride (3ar):

16 mg, 20% yield, on 0.8 mmol scale, colorless oil, the characterization data are in accordance with the literature.[5]

^{1}H NMR (400 MHz, $CDCl_3$) δ 7.13 (dd, J = 15.2, 6.8 Hz, 1H), 6.30 (d, J = 15.2 Hz, 1H), 2.33 – 2.26 (m, 1H), 1.85 – 1.78 (m, 4H), 1.75 – 1.70 (m, 1H), 1.38 – 1.19 (m, 5H);

^{19}F NMR (376 MHz, $CDCl_3$) δ 60.0.

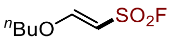

(*E*)-2-(Pentyloxy)ethene-1-sulfonyl fluoride (3as)：

16 mg, 87% yield, colorless oil.

^{1}H NMR (500 MHz, $CDCl_3$) δ 7.69 (dd, J = 13.0 Hz, 3.0 Hz, 1H), 5.72 (dd, J = 12.5 Hz, 4.5 Hz, 1H), 3.98 (t, J = 6.0 Hz, 2H), 1.84 – 1.65 (m, 2H), 1.49 – 1.38 (m, 2H), 0.99 (t, J = 7.0 Hz, 3H);

^{19}F NMR (471 MHz, $CDCl_3$) δ 67.6;

^{13}C NMR (126 MHz, $CDCl_3$) δ 165.3 (d, *J* = 1.3 Hz), 98.0 (d, *J* = 29.0 Hz), 73.1, 30.6, 18.8, 13.5;

HRMS (ESI) *m/z* calcd. for $C_6H_{12}FO_3S$ $[M+H]^+$ 183.0484, found 183.0488.

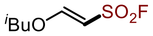

(*E*)-2-Isobutoxyethene-1-sulfonyl fluoride (3at)：

16 mg, 87% yield, colorless oil.

^{1}H NMR (400 MHz, $CDCl_3$) *δ* 7.69 (d, *J* = 14.4 Hz, 1H), 5.72 (dd, *J* = 14.0, 4.0 Hz, 1H), 3.74 (d, *J* = 6.4 Hz, 2H), 2.14 – 1.99 (m, 1H), 0.99 (d, *J* = 6.8 Hz, 6H);

^{19}F NMR (376 MHz, $CDCl_3$) *δ* 67.5;

^{13}C NMR (101 MHz, $CDCl_3$) *δ* 165.4 (d, *J* = 2.1 Hz), 98.0 (d, *J* = 28.3 Hz), 79.4, 28.0, 18.7;

HRMS (ESI) *m/z* calcd. for $C_6H_{12}FO_3S$ $[M+H]^+$ 183.0486, found 183.0489.

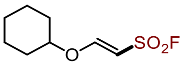

(*E*)-2-(Cyclohexyloxy)ethene-1-sulfonyl fluoride (3au)：

18 mg, 85% yield, colorless oil.

^{1}H NMR (400 MHz, $CDCl_3$) *δ* 7.65 (dd, *J* = 12.0, 2.4 Hz, 1H), 7.58 (dd, *J* = 14.4, 2.8 Hz, 1H), 4.14 – 4.02 (m, 1H), 2.00 – 1.89 (m, 2H), 1.82 – 1.73 (m, 2H), 1.57 – 1.49 (m, 2H), 1.42 – 1.28 (m, 4H);

^{19}F NMR (376 MHz, $CDCl_3$) *δ* 67.6;

^{13}C NMR (101 MHz, $CDCl_3$) *δ* 164.6 (d, *J* = 1.0 Hz), 98.3 (d, *J* = 28.3 Hz), 84.0, 31.6, 24.9, 23.2;

HRMS (ESI) *m/z* calcd. for $C_8H_{14}FO_3S$ $[M+H]^+$ 209.0642, found 208.0649.

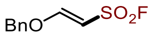

(*E*)-2-(Benzyloxy)ethene-1-sulfonyl fluoride (3av)：

18 mg, 84% yield, colorless oil.

^{1}H NMR (400 MHz, $CDCl_3$) *δ* 7.75 (dd, *J* = 9.6, 1.6 Hz, 1H), 7.55 – 7.37 (m, 3H), 7.41 – 7.30 (m, 2H), 5.84 (dd, *J* = 10.0, 3.2 Hz, 1H), 5.02 (s, 2H);

^{19}F NMR (376 MHz, $CDCl_3$) *δ* 67.5;

^{13}C NMR (101 MHz, $CDCl_3$) *δ* 164.6 (d, *J* = 2.3 Hz), 133.4, 129.4, 129.0, 128.0, 99.3 (d, *J* = 28.3 Hz), 75.0;

HRMS (ESI) *m/z* calcd. for $C_9H_{10}FO_3S$ $[M+H]^+$ 217.0329, found 217.0324.

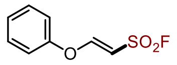

(*E*)-2-Phenoxyethene-1-sulfonyl fluoride (3aw)：

16 mg, 81% yield, colorless oil.

^{1}H NMR (400 MHz, $CDCl_3$) δ 7.91 (dd, *J* = 12.0, 2.4 Hz, 1H), 7.60 – 7.38 (m, 2H), 7.36 – 7.26 (m, 1H), 7.20 – 6.98 (m, 2H), 5.99 (dd, *J* = 12.0, 3.6 Hz, 1H);

^{19}F NMR (376 MHz, $CDCl_3$) δ 67.1;

^{13}C NMR (101 MHz, $CDCl_3$) δ 162.7 (d, *J* = 2.9 Hz), 154.7, 130.4, 126.6, 118.4, 103.0 (d, *J* = 29.3 Hz);

HRMS (ESI) *m/z* calcd. for $C_8H_8FO_3S$ $[M+H]^+$ 203.0173, found 203.0175.

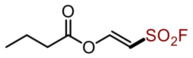

(*E*)-2-(Fluorosulfonyl)vinyl butyrate (3ax)：

17 mg, 85% yield, colorless oil.

^{1}H NMR (400 MHz, $CDCl_3$) δ 8.49 (dd, *J* = 12.4, 2.4 Hz, 1H), 6.31 (dd, *J* = 12.4, 3.2 Hz, 1H), 2.53 (t, *J* = 7.2 Hz, 2H), 1.83 – 1.68 (m, 2H), 1.01 (t, *J* = 7.2 Hz, 3H);

^{19}F NMR (376 MHz, $CDCl_3$) δ 66.1;

^{13}C NMR (101 MHz, $CDCl_3$) δ 168.0, 152.5 (d, *J* = 3.9 Hz), 107.4 (d, *J* = 31.3 Hz), 35.3, 17.8, 13.4;

HRMS (ESI) *m/z* calcd. for $C_6H_9FO_4S$ $[M+H]^+$ 197.0278, found 197.0281.

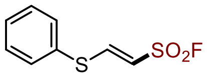

(*E*)-2-(Phenylthio)ethene-1-sulfonyl fluoride (3ay)：

15 mg, 70% yield, colorless oil.

^{1}H NMR (500 MHz, $CDCl_3$) δ 8.02 (d, *J* = 14.5 Hz, 1H), 7.53 – 7.48 (m, 5H), 5.83 (dd, *J* = 14.5 Hz, 2.5 Hz, 1H);

^{19}F NMR (471 MHz, $CDCl_3$) δ 63.0;

^{13}C NMR (101 MHz, $CDCl_3$) δ 154.1 (d, *J* = 1.5 Hz), 134.0, 130.8, 130.4, 127.2, 113.1 (d, *J* = 37.8 Hz);

HRMS (ESI) *m/z* calcd. for $C_8H_8FO_2S_2$ $[M+H]^+$ 218.9944, found 218.9944.

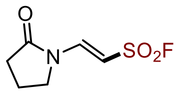

(*E*)-2-(2-Oxopyrrolidin-1-yl)ethene-1-sulfonyl fluoride (3az):

17 mg, 90% yield, colorless oil.

^{1}H NMR (500 MHz, $CDCl_3$) δ 8.15 (d, J = 15.5 Hz, 1H), 5.68 (dd, J = 15.5 Hz, 2.5 Hz, 1H), 3.59 (t, J = 8.0 Hz, 2H), 2.61 (t, J = 9.0 Hz, 2H), 2.29 – 2.19 (m, 2H);

^{19}F NMR (471 MHz, $CDCl_3$) δ 67.1;

^{13}C NMR (126 MHz, $CDCl_3$) δ 174.1, 140.8 (d, J = 2.5 Hz), 99.8 (d, J = 30.2 Hz), 44.9, 30.4, 17.4;

HRMS (ESI) *m/z* calcd. for $C_6H_9FNO_3S$ $[M+H]^+$ 194.0282, found 194.0286.

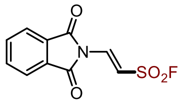

(*E*)-2-(1,3-Dioxoisoindolin-2-yl)ethene-1-sulfonyl fluoride (3ba)：

23 mg, 89% yield, white solid, m.p. 156–157 ˚C.

^{1}H NMR (400 MHz, $CDCl_3$) δ 8.05 (dd, J = 14.0, 1.2 Hz, 1H), 8.04 – 7.99 (m, 2H), 7.95 – 7.85 (m, 2H), 7.58 (dd, J = 14.0, 2.8 Hz, 1H);

^{19}F NMR (376 MHz, $CDCl_3$) δ 64.8;

^{13}C NMR (101 MHz, $CDCl_3$) δ 164.4, 136.0, 133.9 (d, J = 3.0 Hz), 131.0, 125.0, 109.2 (d, J = 30.3 Hz);

HRMS (ESI) *m/z* calcd. for $C_{10}H_7FNO_4S$ $[M+H]^+$ 256.0074, found 256.0081.

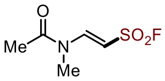

(*E*)-2-(N-methylacetamido)ethene-1-sulfonyl fluoride (3bb)：

16 mg, 90% yield, colorless oil.

^{1}H NMR (400 MHz, $CDCl_3$) δ 8.21 (brs, 1H), 5.72 (dd, J = 13.2, 3.6 Hz, 1H), 3.18 (s, 3H), 2.40 (s, 3H);

^{19}F NMR (376 MHz, $CDCl_3$) δ 66.8;

^{13}C NMR (101 MHz, $CDCl_3$) δ 169.7, 146.2, 98.3 (d, J = 18.2 Hz), 29.8, 21.8;

HRMS (ESI) *m/z* calcd. for $C_5H_9FNO_3S$ $[M+H]^+$ 182.0282, found 182.0289.

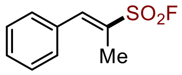

(*E*)-1-Phenylprop-1-ene-2-sulfonyl fluoride (5aa)：

17 mg, 90% yield, colorless oil, the characterization data are in accordance with the literature.[5]

^{1}H NMR (400 MHz, $CDCl_3$) δ 7.78 (s, 1H), 7.52 – 7.41 (m, 5H), 2.39 (s, 3H);

^{19}F NMR (376 MHz, $CDCl_3$) δ 50.5.

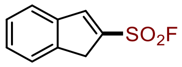

1H-indene-2-sulfonyl fluoride (5ab)：

18 mg, 91 % yield, white solid, the characterization data are in accordance with the literature.[5]

^{1}H NMR (400 MHz, $CDCl_3$) δ 7.94 (s, 1H), 7.67 – 7.56 (m, 2H), 7.51 – 7.42 (m, 2H), 3.92 (s, 2H);

^{19}F NMR (376 MHz, $CDCl_3$) δ 62.8.

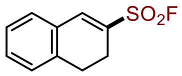

3,4-Dihydronaphthalene-2-sulfonyl fluoride (5ac)：

18 mg, 86% yield, colorless oil, the characterization data are in accordance with the iterature.[5]

^{1}H NMR (400 MHz, $CDCl_3$) δ 7.62 (s, 1H), 7.40 – 7.35 (m, 1H), 7.31 – 7.28 (m, 2H), 7.23 (d, J = 7.6 Hz, 1H), 3.05 (t, J = 8.4 Hz, 2H), 2.79 (t, J = 8.4 Hz, 2H);

^{19}F NMR (376 MHz, $CDCl_3$) δ 54.2.

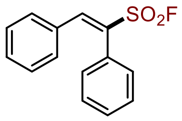

(*E*)-1,2-Diphenylethene-1-sulfonyl fluoride (5ad)：

21 mg, 80% yield, white solid, the characterization data are in accordance with the literature.[5]

^{1}H NMR (400 MHz, $CDCl_3$) δ 7.93 (s, 1H), 7.53 – 7.46 (m, 3H), 7.46 – 7.40 (m, 2H), 7.34 (t, J = 7.6 Hz, 1H), 7.26 – 7.20 (m, 2H), 7.11 (d, J = 8.0 Hz, 2H);

^{19}F NMR (376 MHz, $CDCl_3$) δ 53.1.

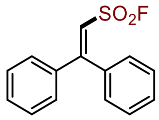

2,2-Diphenylethene-1-sulfonyl fluoride (5ae)：

16 mg, 62% yield, white solid, the characterization data are in accordance with the literature.[5]

^{1}H NMR (500 MHz, $CDCl_3$) δ 7.54 – 7.24 (m, 4H), 7.40 (t, J = 7.6 Hz, 2H), 7.36 – 7.29 (m, 4H), 6.83 (s, 1H);

^{19}F NMR (471 MHz, $CDCl_3$) δ 68.2.

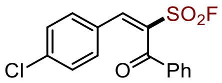

(*E*)-1-(4-Chlorophenyl)-3-oxo-3-phenylprop-1-ene-2-sulfonyl fluoride (5af)：

17 mg, 53% yield, white solid, the characterization data are in accordance with the literature.[5]

^{1}H NMR (400 MHz, $CDCl_3$) δ 8.06 (s, 1H), 7.94 (d, J = 7.6 Hz, 2H), 7.66 – 7.57 (m, 1H), 7.46 (t, J = 8.0 Hz, 2H), 7.34 – 7.14 (m, 4H);

^{19}F NMR (376 MHz, $CDCl_3$) δ 62.9.

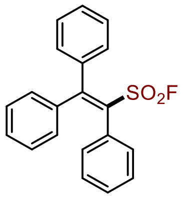

1,2,2-Triphenylethene-1-sulfonyl fluoride (5ag)：

22 mg, 64% yield, white solid, the characterization data are in accordance with the literature.[5]

^{1}H NMR (500 MHz, $CDCl_3$) δ 7.46 – 7.36 (m, 7H), 7.31 – 7.26 (m, 3H), 7.14 – 7.05 (m, 3H), 6.98 – 6.93 (m, 2H);

^{19}F NMR (471 MHz, $CDCl_3$) δ 62.4.

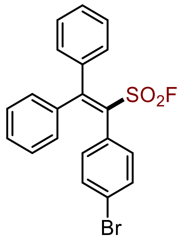

1-(4-Bromophenyl)-2,2-diphenylethene-1-sulfonyl fluoride (5ah)：

38 mg, 92% yield, white solid, m.p. 148–149 ˚C.

^{1}H NMR (400 MHz, $CDCl_3$) δ 7.45 – 7.38 (m, 5H), 7.38 – 7.33 (m, 2H), 7.30 – 7.21 (m, 2H), 7.20 – 7.05 (m, 3H), 6.98 – 6.90 (m, 2H);

^{19}F NMR (376 MHz, $CDCl_3$) δ 62.8;

^{13}C NMR (101 MHz, $CDCl_3$) δ 159.0 (d, J = 2.2 Hz), 139.0 (d, J = 2.2 Hz), 138.6, 133.8, 133.0 (d, J = 21.1 Hz), 131.8, 131.6, 129.7, 129.6, 129.2, 128.6, 128.3, 128.2, 123.9;
HRMS (EI) *m/z* calcd. for $C_{20}H_{14}BrFO_2S$ $[M]^+$ 415.9882, found 415.9886.

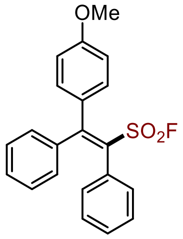

(*Z/E*)-2-(4-Methoxyphenyl)-1,2-diphenylethene-1-sulfonyl fluoride (5ai)：

30 mg, 41% yield on 0.2 mmol scale, *E/Z* = 1:1, white solid, m.p. 121–122 ˚C.
^{1}H NMR (400 MHz, $CDCl_3$) δ 7.47 – 7.41 (m, 2H), 7.41 – 7.35 (m, 2H), 7.35 – 7.29 (m, 2H), 7.29 – 7.27 (m, 2H), 7.16 – 7.01 (m, 2H), 7.00 – 6.91 (m, 2H), 6.90 – 6.83 (m, 1H), 6.62 – 6.55 (m, 1H), 3.85 (s, 1.6H), 3.69 (s, 1.5H);
^{19}F NMR (376 MHz, $CDCl_3$) δ 62.9 (s, 1F), 62.1 (s, 1F);
^{13}C NMR (101 MHz, $CDCl_3$) δ 160.8, 160.2, 158.5 (d, J = 2.0 Hz), 158.0 (d, J = 2.0 Hz), 139.9 (d, J = 2.4 Hz), 139.4, 133.1 (d, J = 21.2 Hz), 132.6, 132.5, 132.3, 130.9, 130.2, 129.4, 129.3, 129.2, 129.1, 129.0, 128.7, 128.4, 128.3, 127.9, 113.8, 113.4, 55.4, 55.3;
HRMS (EI) *m/z* calcd. for $C_{21}H_{17}FO_3S$ $[M]^+$ 368.0882, found 368.0888.

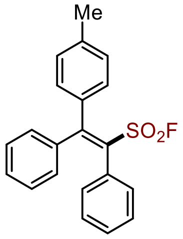

(*Z/E*)-1,2-Diphenyl-2-(p-tolyl)ethene-1-sulfonyl fluoride (5aj)：

22 mg, 62% yield, *E/Z* = 1:1, white solid, m.p. 115–116 ˚C.
^{1}H NMR (400 MHz, $CDCl_3$) δ 7.47 – 7.34 (m, 5H), 7.34 – 7.25 (m, 4H), 7.25 – 7.20 (m, 1H), 7.15 – 7.01 (m, 1H), 6.97 – 6.93 (m, 1H), 6.90 – 6.86 (m, 1H), 6.85 – 6.80 (m, 1H), 2.40 (s, 1.5H, isomer 1), 2.20 (s, 1.5H, isomer 2);
^{19}F NMR (376 MHz, $CDCl_3$) δ 62.6 (s, 1F), 62.3 (s, 1F);
^{13}C NMR (101 MHz, $CDCl_3$) δ 158.7 (d, J = 2.0 Hz), 158.4 (d, J = 2.0 Hz), 139.74, 139.67 (d, J = 2.0 Hz), 139.4, 139.2, 136.4 (d, J = 2.0 Hz), 136.1, 133.7 (d, J = 20.2 Hz), 133.5 (d, J = 20.2 Hz), 132.9, 132.8, 132.4, 130.2, 130.0, 129.4, 129.3, 129.1, 128.90, 128.88, 128.7, 128.6, 128.5, 128.3, 128.0, 21.6, 21.3;

HRMS (EI) *m/z* calcd. for $C_{21}H_{17}FO_2S$ $[M]^+$ 352.0933, found 352.0931.

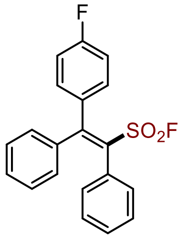

(*Z*/*E*)-2-(4-Fluorophenyl)-1,2-diphenylethene-1-sulfonyl fluoride (5ak)：

32 mg, 89% yield, *E*/*Z* = 1:1, white solid, m.p. 111–112 ˚C.

^{1}H NMR (400 MHz, $CDCl_3$) δ 7.48 – 7.45 (m, 1H), 7.45 – 7.42 (m, 1H), 7.42 – 7.35 (m, 4H), 7.33 – 7.26 (m, 3H), 7.18 – 7.04 (m, 2H), 6.97 – 6.91 (m, 2H), 6.82 – 6.73 (m, 1H);

^{19}F NMR (376 MHz, $CDCl_3$) δ 62.4 (s, 1F), 62.3 (s, 1F), -110.4 – -110.8 (m, 1F), -110.8 – -111.2 (m, 1F);

^{13}C NMR (101 MHz, $CDCl_3$) δ 164.4 (d, *J* = 72.7 Hz), 161.9 (d, *J* = 73.0 Hz), 157.3 (d, *J* = 2.0 Hz), 157.2 (d, *J* = 2.0 Hz), 139.3 (d, *J* = 2.2 Hz), 138.8, 135.4 (d, *J* = 3.2 Hz), 134.9 (d, *J* = 3.4 Hz), 134.7, 134.5, 134.2, 132.6, 132.5, 132.4, 132.2 (d, *J* = 8.6 Hz), 131.0 (d, *J* = 8.6 Hz), 130.0, 129.7 (d, *J* = 15.0 Hz), 129.4 (d, *J* = 26.3 Hz), 128.9, 128.8, 128.60, 128.55, 128.2, 115.7 (d, *J* = 22.0 Hz), 115.3 (d, *J* = 21.9 Hz);

HRMS (EI) *m/z* calcd. for $C_{20}H_{14}F_2O_2S$ $[M]^+$ 356.0683, found 356.0688.

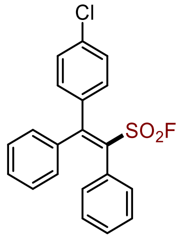

(*Z*/*E*) -2-(4-Chlorophenyl)-1,2-diphenylethene-1-sulfonyl fluoride (5al)：

32 mg, 87% yield, *E*/*Z* = 1:1, white solid, m.p. 104–105 ˚C.

^{1}H NMR (400 MHz, $CDCl_3$) δ 7.47 – 7.42 (m, 2H), 7.42 – 7.34 (m, 4H), 7.34 – 7.26 (m, 4H), 7.14 – 7.08 (m, 1H), 7.08 – 7.03 (m, 1H), 6.97 – 6.91 (m, 1H), 6.91 – 6.83 (m, 1H);

^{19}F NMR (376 MHz, $CDCl_3$) δ 62.4 (s, 1F), 62.3 (s, 1F);

^{13}C NMR (101 MHz, $CDCl_3$) δ 156.9 (d, *J* = 2.0 Hz), 156.8 (d, *J* = 2.0 Hz), 139.0 (d, *J* = 2.6 Hz), 138.5, (d, *J* = 2.6 Hz), 137.3, 135.7, 135.2, 134.8, 134.6, 132.33, 132.31, 132.2, 131.4, 130.2, 129.9, 129.7, 129.6, 129.5, 129.2, 128.83, 128.76, 128.7, 128.6, 128.5, 128.4, 128.2;

HRMS (EI) *m/z* calcd. for $C_{20}H_{14}ClFO_2S$ $[M]^+$ 372.0387, found 372.0381.

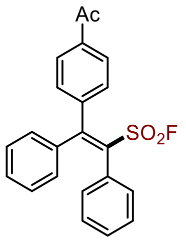

2-(4-Acetylphenyl)-1,2-diphenylethene-1-sulfonyl fluoride (5am)：

20 mg, 52% yield, one configuration was isolated, white solid, m.p. 123–124 ˚C.

^{1}H NMR (400 MHz, $CDCl_3$) δ 8.01 (d, J = 8.0 Hz, 2H), 7.49 (d, J = 8.0 Hz, 2H), 7.44 – 7.38 (m, 2H), 7.35 – 7.27 (m, 3H), 7.19 – 7.04 (m, 3H), 6.99 – 6.90 (m, 2H), 2.64 (s, 3H);

^{19}F NMR (376 MHz, $CDCl_3$) δ 62.4;

^{13}C NMR (101 MHz, $CDCl_3$) δ 197.5, 156.9 (d, J = 2.0 Hz), 143.6, 138.7 (d, J = 2.0 Hz), 137.5, 135.2 (d, J = 21.2 Hz), 132.2, 132.1, 129.8, 129.7, 129.3, 129.0, 128.7, 128.5, 128.3, 26.8;

HRMS (EI) *m/z* calcd. for $C_{22}H_{17}FO_3S$ $[M]^+$ 380.0882, found 380.0885.

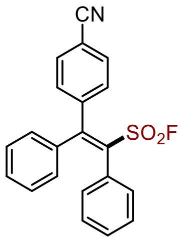

(*Z/E*)-2-(4-Cyanophenyl)-1,2-diphenylethene-1-sulfonyl fluoride (5an)：

31 mg, 85% yield, *E*/*Z* = 1:1, white solid, m.p. 102–103 ˚C.

^{1}H NMR (400 MHz, $CDCl_3$) δ 7.77 – 7.69 (m, 1H), 7.53 – 7.48 (m, 1H), 7.47 – 7.43 (m, 1H), 7.41 – 7.34 (m, 4H), 7.34 – 7.27 (m, 3H), 7.22 – 7.02 (m, 3H), 7.95 – 7.90 (m, 1H);

^{19}F NMR (376 MHz, $CDCl_3$) δ 62.5 (s, 1F), 61.9 (s, 1F);

^{13}C NMR (101 MHz, $CDCl_3$) δ 155.8 (d, J = 2.0 Hz), 143.9 (d, J = 3.0 Hz), 143.5, 138.1 (d, J = 2.0 Hz), 137.7, 136.3 (d, J = 21.8 Hz), 135.7 (d, J = 21.5 Hz), 132.3, 132.1, 132.0, 131.9, 131.7, 131.6, 130.3, 130.0, 129.8, 129.7, 129.6, 129.4, 128.9, 128.80, 128.76, 128.7, 128.4, 118.2 (d, J = 29.5 Hz), 112.9 (d, J = 61.0 Hz);

HRMS (EI) *m/z* calcd. for $C_{21}H_{14}FNO_2S$ $[M]^+$ 363.0729, found 363.0734.

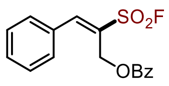

(*E*)-2-(Fluorosulfonyl)-3-phenylallyl benzoate (5ao)：

26 mg, 91% yield, colorless oil.

^{1}H NMR (500 MHz, $CDCl_3$) δ 8.18 (s, 1H), 8.08 (d, J = 8.0 Hz, 2H), 7.65 – 7.59 (m, 1H), 7.56 – 7.44 (m, 7H), 5.37 (s, 2H);

^{19}F NMR (471 MHz, $CDCl_3$) δ 59.8;

^{13}C NMR (126 MHz, $CDCl_3$) δ 165.9, 149.5 (d, J = 1.3 Hz), 133.8, 131.9, 131.3 (d, J = 1.3 Hz), 130.2, 130.0, 129.5, 129.1, 128.8, 58.4 (d, J = 1.3 Hz);

HRMS (EI) *m/z* calcd. for $C_{16}H_{13}FO_4S$ $[M]^+$ 320.0519, found 320.0514.

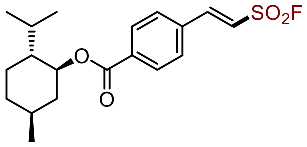

(1*S*,2*R*,5*S*)-2-Isopropyl-5-methylcyclohexyl 4-((E)-2-(fluorosulfonyl)vinyl)benzoate (5ap):

30 mg, 81% yield, colorless oil.

^{1}H NMR (500 MHz, $CDCl_3$) δ 8.13 (d, J = 8.5 Hz, 2H), 7.84 (d, J = 15.5 Hz, 1H), 7.62 (d, J = 8.5 Hz, 2H), 6.96 (d, J = 15.5 Hz, 1H), 4.99 – 4.93 (m, 1H), 2.13 (d, J = 12.5 Hz, 1H), 1.95 – 1.90 (m, 1H), 1.81 – 1.70 (m, 2H), 1.63 – 1.51 (m, 2H), 1.20 – 1.05 (m, 2H), 0.93 (brs, 6H), 0.80 (d, J = 7.0 Hz, 3H);

^{19}F NMR (471 MHz, $CDCl_3$) δ 62.2;

^{13}C NMR (126 MHz, $CDCl_3$) δ 165.0, 147.5 (d, J = 3.0 Hz), 134.7, 134.3, 130.6, 128.9, 120.3 (d, J = 28.9 Hz), 75.7, 47.3, 41.0, 34.3, 31.5, 26.7, 23.7, 22.1, 20.8, 16.6;

HRMS (EI) *m/z* calcd. for $C_{19}H_{25}FO_4S$ $[M]^+$ 368.1458, found 368.1455.

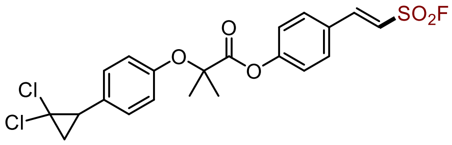

(*E*)-4-(2-(Fluorosulfonyl)vinyl)phenyl 2-(4-(2,2-dichlorocyclopropyl)phenoxy)-2-methylpropanoate (5aq):

34 mg, 71% yield, white solid, m.p. 63–64 ˚C.

^{1}H NMR (400 MHz, $CDCl_3$) δ 7.77 (d, J = 15.6 Hz, 1H), 7.54 (d, J = 7.2 Hz, 2H), 7.17 (d, J = 7.2 Hz, 2H), 7. 07 (d, J = 7.1 Hz, 2H), 6.93 (d, J = 7.0, 2H), 6.83 (d, J = 15.6 Hz, 1H), 2.87 (t, J = 2.8 Hz,1H), 2.07 – 1.89 (m, 1H), 1.83 – 1.80 (m, 1H), 1.77 (s, 6H);

^{19}F NMR (376 MHz, $CDCl_3$) δ 62.4;

^{13}C NMR (101 MHz, $CDCl_3$) δ 172.5, 154.9, 153.7, 147.6 (d, J = 3.0 Hz), 130.4, 130.0, 128.9 (d, J = 19.3 Hz), 122.6, 118.6, 118.4, 118.2, 79.4, 60.9, 34.8, 25.9, 25.5;

HRMS (EI) *m/z* calcd. for $C_{21}H_{19}Cl_2FO_5S$ $[M]^+$ 472.0314, found 472.0319.

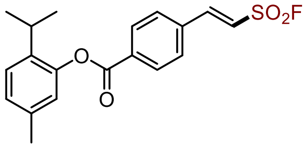

2-Isopropyl-5-methylphenyl (*E*)-4-(2-(fluorosulfonyl)vinyl)benzoate (5ar):

32 mg, 87% yield, white solid, m.p. 104–105 ˚C.

^{1}H NMR (400 MHz, $CDCl_3$) δ 8.31 (d, *J* = 8.0 Hz, 2H), 7.89 (d, *J* = 15.6 Hz, 1H), 7.71 (d, *J* = 8.0 Hz, 2H), 7.30 – 7.23 (m, 1H), 7.10 (d, *J* = 7.6 Hz, 1H), 7.02 (d, *J* = 15.6 Hz, 1H), 6.95 (s, 1H), 3.14 – 2.84 (m, 1H), 2.35 (s, 3H), 1.21 (d, *J* = 6.8 Hz, 6H);

^{19}F NMR (376 MHz, $CDCl_3$) δ 62.1;

^{13}C NMR (101 MHz, $CDCl_3$) δ 164.3, 147.9, 147.2 (d, *J* = 2.8 Hz), 137.1, 136.9, 135.5, 133.0, 131.1, 129.2, 127.6, 126.7, 122.7, 120.8 (d, *J* = 29.0 Hz), 27.4, 23.1, 20.9;

HRMS (EI) *m/z* calcd. for $C_{19}H_{19}FO_4S$ $[M]^+$ 362.0988, found 362.0995.

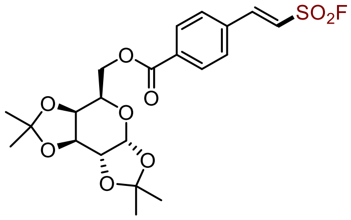

((3a*R*,5*R*,5a*S*,8a*S*,8b*R*)-2,2,7,7-tetramethyltetrahydro-5H-bis([1,3]dioxolo)[4,5-b:4',5'-*d*]pyran-5-yl)methyl 4-((*E*)-2-(fluorosulfonyl)vinyl)benzoate (5as):

36 mg, 77% yield, colorless oil.

^{1}H NMR (400 MHz, $CDCl_3$) δ 8.14 (d, *J* = 7.6 Hz, 2H), 7.84 (d, *J* = 15.6 Hz, 1H), 7.63 (d, *J* = 7.6 Hz, 2H), 6.97 (d, *J* = 15.6 Hz, 1H), 5.58 (s, 1H), 4.67 (d, *J* = 7.6 Hz, 1H), 4.61 – 4.42 (m, 2H), 4.41 – 4.29(m, 2H), 4.23 – 4.15 (m, 1H), 1.52 (s, 3H), 1.48 (s, 3H), 1.36 (s, 3H), 1.34 (s, 3H);

^{19}F NMR (376 MHz, $CDCl_3$) δ 62.1;

^{13}C NMR (101 MHz, $CDCl_3$) δ 165.3, 147.4 (d, *J* = 2.5 Hz), 135.0, 133.5, 130.7, 129.0, 120.4 (d, *J* = 29.0 Hz), 109.9, 108.9, 96.4, 71.2, 70.8, 70.5, 66.2, 64.6, 26.1, 25.0, 24.6;

HRMS (EI) *m/z* calcd. for $C_{21}H_{25}FO_9S$ $[M]^+$ 472.1203, found 472.1207.

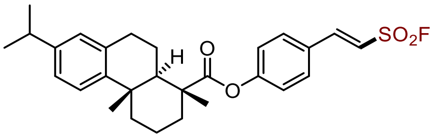

4-((*E*)-2-(Fluorosulfonyl)vinyl)phenyl (1*R*,4a*S*,10a*R*)-7-isopropyl-1,4a-dimethyl-1,2,3,4,4a,9,10,10a-octahydrophenanthrene-1-carboxylate (5at):

37 mg, 76% yield, white solid, 80–81 ˚C.

^{1}H NMR (400 MHz, $CDCl_3$) δ 7.79 (d, *J* = 15.6 Hz, 1H), 7.57 (d, *J* = 8.4 Hz, 2H), 7.20 (d, *J* = 8.4 Hz, 1H), 7.18 – 7.11 (m, 2H), 7.03 (d, *J* = 8.4 Hz, 1H), 6.91 (s, 1H), 6.82 (d, *J* = 15.6 Hz, 1H), 2.97 – 2.94 (m, 2H), 2.89 – 2.74 (m, 1H), 2.54 – 2.24 (m, 2H), 2.10 – 1.89 (m, 2H), 1.90 – 1.74 (m, 2H), 1.65 – 1.57 (m, 2H), 1.40 (s, 3H), 1.27 (s, 3H), 1.22 (d, *J* = 6.8 Hz, 6H);

^{19}F NMR (376 MHz, $CDCl_3$) δ 62.5;

^{13}C NMR (101 MHz, $CDCl_3$) δ 176.7, 154.6, 147.9, 147.8 (d, *J* = 2.8 Hz), 146.7, 146.1, 134.5, 130.4, 127.0, 124.3 (d, *J* = 11.5 Hz), 122.8, 76.8, 48.2, 44.9, 38.0, 37.1, 36.5, 33.6, 30.2, 25.2, 24.1, 22.1, 18.6, 16.7;

HRMS (EI) *m/z* calcd. for $C_{28}H_{33}FO_4S$ $[M]^+$ 484.2084, found 484.2089.

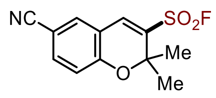

6-Cyano-2,2-dimethyl-2H-chromene-3-sulfonyl fluoride (5au)：

25 mg, 92% yield, white solid, the characterization data are in accordance with the iterature.[5]

^{1}H NMR (500 MHz, $CDCl_3$) δ 7.65 (dd, *J* = 8.5, 2.0 Hz, 1H), 7.60 (brs, 1H), 7.58 (d, *J* = 2.0 Hz, 1H), 7.01 (d, *J* = 8.5 Hz, 1H), 1.746 (s, 3H), 1.745 (s, 3H);

^{19}F NMR (471 MHz, $CDCl_3$) δ 61.7.

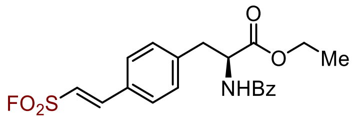

Ethyl (*S*, *E*)-2-benzamido-3-(4-(2-(fluorosulfonyl)vinyl)phenyl)propanoate (5av):

35 mg, 86% yield, white solid, m.p. 156–157 ˚C.

^{1}H NMR (500 MHz, $CDCl_3$) δ 7.79 (d, *J* = 20.0 Hz, 1H), 7.75 – 7.71 (m, 2H), 7.57 – 7.51 (m, 1H), 7.49 – 7.40 (m, 4H), 7.29 – 7.24 (m, 1H), 6.83 (dd, *J* = 15.5, 2.5 Hz, 1H), 6.67 (d, *J* = 7.5 Hz, 1H), 5.14 – 5.02 (m, 1H), 4.31 – 4.17 (m, 2H), 3.39 (dd, *J* = 14.0, 6.5 Hz, 1H), 3.27 (dd, *J* = 14.0, 5.5 Hz, 1H), 1.29 (t, *J* = 7.5 Hz, 3H);

^{19}F NMR (471 MHz, $CDCl_3$) δ 62.5;

^{13}C NMR (126 MHz, $CDCl_3$) δ 171.4, 166.9, 148.5, 141.6, 133.8, 132.1, 130.7, 117.9 (d, *J* = 28.1 Hz), 62.1, 53.5, 38.2, 14.3;

HRMS (EI) *m/z* calcd. for $C_{20}H_{20}FNO_5S$ $[M]^+$ 405.1046, found 405.1041.

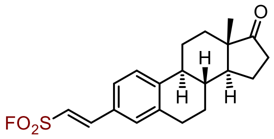

(*E*)-2-(Fluorosulfonyl)vinyl pivalate (5aw)：

33 mg, 90% yield, white solid, the characterization data are in accordance with the literature.[5]

^{1}H NMR (500 MHz, $CDCl_3$) δ 7.76 (d, J = 15.5 Hz, 1H), 7.40 – 7.36 (m, 1H), 7.36 – 7.31 (m, 1H), 7.30 – 7.27 (m, 1H), 6.82 (dd, J = 15.5, 2.5 Hz, 1H), 2.97 – 2.95 (m, 2H), 2.59 – 2.48 (m, 1H), 2.48 – 2.29 (m, 2H), 2.22 – 2.12 (m, 1H), 2.12 – 2.03 (m, 2H), 2.02 – 1.96 (m, 1H), 1.67 – 1.56 (m, 3H), 1.55 – 1.45 (m, 3H), 0.93 (s, 3H);

^{19}F NMR (471 MHz, $CDCl_3$) δ 62.7.

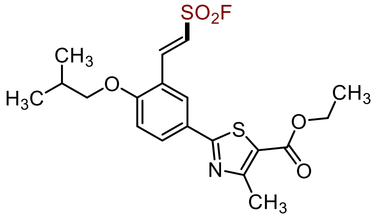

Ethyl (*E*)-2-(3-(2-(fluorosulfonyl)vinyl)-4-isobutoxyphenyl)-4-methylthiazole-5-carboxylate (5ax):

36 mg, 85% yield, white solid, m.p. 132–133 ˚C.

^{1}H NMR (500 MHz, $CDCl_3$) δ 8.10 (d, J = 2.0 Hz, 1H), 8.01 (dd, J = 4.5, 2.5 Hz, 1H), 8.00 (d, J = 16.0 Hz, 1H), 7.24 (dd, J = 15.5, 2.5 Hz, 1H), 7.03 (d, J = 9.0 Hz, 1H), 4.36 (q, J = 7.0 Hz, 2H), 3.93 (d, J = 6.5 Hz, 2H), 2.78 (s, 3H), 2.30 – 2.17 (m, 1H), 1.39 (t, J = 7.0 Hz, 3H), 1.10 (d, J = 7.0 Hz, 6H);

^{19}F NMR (471 MHz, $CDCl_3$) δ 62.2;

^{13}C NMR (126 MHz, $CDCl_3$) δ 168.0, 162.2, 161.2, 160.7, 143.8 (d, J = 2.5 Hz), 132.1, 129.9, 126.2, 121.8, 120.6, 120.1 (d, J = 27.7 Hz), 112.9, 75.8, 61.4, 28.2, 19.4, 17.6, 14.4;

HRMS (EI) *m/z* calcd. for $C_{19}H_{22}FNO_5S_2$ $[M]^+$ 427.0923, found 427.0929.

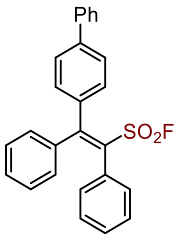

(*Z*/*E*)-2-([1,1'-Biphenyl]-4-yl)-1,2-diphenylethene-1-sulfonyl fluoride (5ay)：

28 mg, 68% yield, *E*/*Z* = 1:1, white solid, 119–120 ˚C.

^{1}H NMR (400 MHz, CDCl$_3$) δ 7.64 (t, J = 8.0 Hz, 2H), 7.49 – 7.40 (m, 7H), 7.40 – 7.33 (m, 2H), 7.34 – 7.26 (m, 4H), 7.14 – 7.07 (m, 2H), 7.03 – 6.98 (m, 2H);
^{19}F NMR (376 MHz, CDCl$_3$) δ 62.6 (s, 1F), 62.4 (s, 1F);
^{13}C NMR (101 MHz, CDCl$_3$) δ 158.0 (d, J = 26.0 Hz), 142.2, 141.6, 140.1, 139.6, 139.4, 138.9, 138.1, 137.8, 132.6 (d, J = 4.0 Hz), 132.3, 130.6, 129.9, 129.4, 129.3, 129.2, 128.9, 128.8, 128.8, 128.5, 128.4, 128.3, 128.0, 127.8, 127.7, 127.1, 126.9, 126.5;
HRMS (EI) *m/z* calcd. for $C_{26}H_{19}FO_2S$ [M]$^+$ 414.1090, found 414.1093.

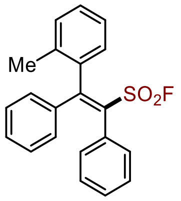

(*Z*)-2-(4-Acetylphenyl)-1,2-diphenylethene-1-sulfonyl fluoride (5az)：

21 mg, 60% yield, white solid, 111–112 ˚C.
^{1}H NMR (500 MHz, CDCl$_3$) δ 7.50 – 7.42 (m, 2H), 7.42 – 7.34 (m, 3H), 7.35 – 7.28 (m, 3H), 7.25 – 7.20 (m, 2H), 7.15 – 7.06 (m, 2H), 7.06 – 7.01 (m, 1H), 7.00 – 6.95 (m, 1H), 2.22 (s, 1.9H, isomer 1), 2.12 (s, 1.1H, isomer 2);
^{19}F NMR (376 MHz, CDCl$_3$) δ 61.8, 61.4;
^{13}C NMR (101 MHz, CDCl$_3$) δ 158.3 (d, J = 2.9 Hz), 157.4 (d, J = 2.4 Hz), 138.9 (d, J = 2.6 Hz), 138.1, 137.5 (d, J = 2.7 Hz), 137.3, 135.3, 135.1 (d, J = 1.8 Hz), 135.0, 134.4, 132.3 (d, J = 9.0 Hz), 132.1, 130.9, 130.6, 130.0, 129.5, 129.4, 129.2, 129.1, 129.0, 128.68, 128.67, 128.61, 128.56, 128.44, 128.37, 128.36, 128.1, 127.9, 20.0, 19.7;
HRMS (EI) *m/z* calcd. for $C_{21}H_{17}FO_2S$ [M]$^+$ 352.0933, found 352.0935.

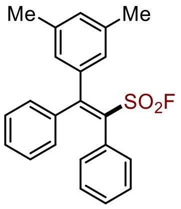

(*Z*/*E*)-2-(3,5-Dimethylphenyl)-1,2-diphenylethene-1-sulfonyl fluoride (5ba)：

23 mg, 63% yield, *E*/*Z* = 1:1, white solid, 124–125 ˚C.
^{1}H NMR (400 MHz, CDCl$_3$) δ 7.49 – 7.41 (m, 2H), 7.41 – 7.36 (m, 3H), 7.30 – 7.26 (m, 3H), 7.15 – 7.04 (m, 2h), 7.01 – 6.92 (m, 2H), 6.55 (s, 1H), 2.34 (s, 3H), 2.07 (s, 3H);
^{19}F NMR (471 MHz, CDCl$_3$) δ 62.5 (s, 1F), 62.2 (s, 1F);

^{13}C NMR (101 MHz, $CDCl_3$) δ 158.7 (d, J = 2.4 Hz), 158.6 (d, J = 2.4 Hz), 139.4 (d, J = 2.7 Hz), 139.07 (d, J = 2.5 Hz), 138.9, 138.7, 137.8, 137.4, 133.9 (d, J = 3.8 Hz), 133.7 (d, J = 3.8 Hz), 132.7 (d, J = 8.1 Hz), 132.3, 132.2, 131.1, 130.6, 129.7, 129.2, 129.1, 129.1, 128.7, 128.6, 128.6, 128.4, 128.2, 128.2, 127.9, 127.6, 126.4, 21.3, 21.0;
HRMS (EI) *m/z* calcd. for $C_{22}H_{19}FO_2S$ $[M]^+$ 366.1090, found 366.1091.

General procedure for the syntheses of 7 (Procedure B). The *fac*-Ir(ppy)$_3$ (0.65 mg, 0.001 mmol, 1 mol%), NaOAc (13.6 mg, 0.1 mmol) and FABI 2e (0.3 mmol) were weighed into an oven-dried Schlenk tube, followed by the addition of anhydrous 1,4-dioxane (1.0 mL, 0.1 M), olefin substrate (0.1 mmol) and ROH (1 mmol) under argon. The reaction mixture was allowed to stir at room temperature under irradiation with blue LEDs for 12 h. Purification by column chromatography or preparative thin layer chromatography on silica gel gave the desired pure product. Photo-induced reactions were conducted in photo-reactors, which comprise a fan for cooling (approximately room temperature) and six 1W blue LED beads for each place (6 W). The average power output of the photo-reactor was ca. 30 mW/cm^2. The emission spectra of the blue LEDs were recorded on an Ocean Optics HR4000CG-UVNIR spectrometer.[5]

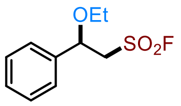

2-Ethoxy-2-phenylethane-1-sulfonyl fluoride (7a):
19 mg, 82% yield, colorless oil.
^{1}H NMR (500 MHz, $CDCl_3$) δ 7.43 – 7.38 (m, 2H), 7.39 – 7.34 (m, 3H), 4.88 (dd, J = 10.0, 5.0 Hz, 1H), 3.80 (dd, J = 15.0, 10.0 Hz, 1H), 3.57 – 3.42 (m, 3H), 1.21 (t, J = 7.0 Hz, 3H);
^{19}F NMR (471 MHz, $CDCl_3$) δ 61.0 (d, J = 6.5 Hz);
^{13}C NMR (126 MHz, $CDCl_3$) δ 138.2 (d, J = 2.7 Hz), 129.3, 129.2, 126.5, 76.2, 65.3, 58.4 (d, J = 13.1 Hz), 15.1;
HRMS (ESI) *m/z* calcd. for $C_{10}H_{13}FNaO_3S$ $[M+Na]^+$ 255.0462, found 255.0466.

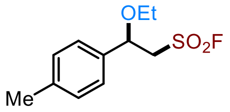

2-Ethoxy-2-(p-tolyl)ethane-1-sulfonyl fluoride (7b):
22 mg, 91% yield, colorless oil.
^{1}H NMR (400 MHz, $CDCl_3$) δ 7.29 – 7.17 (m, 4H), 4.85 (dd, J = 10.0, 3.2 Hz, 1H), 3.80 (dd, J = 14.4, 9.6 Hz, 1H), 3.55 – 3.37 (m, 3H), 2.36 (s, 3H), 1.20 (t, J = 7.2, 3H);

^{19}F NMR (376 MHz, $CDCl_3$) δ 60.9 (d, J = 6.6 Hz);

^{13}C NMR (101 MHz, $CDCl_3$) δ 139.2, 135.2 (d, J = 1.8 Hz), 129.9, 126.5, 76.0, 65.1, 58.5 (d, J = 12.9 Hz), 21.3, 15.1;

HRMS (ESI) *m/z* calcd. for $C_{11}H_{15}FNaO_3S$ $[M+Na]^+$ 269.0618, found 269.0621.

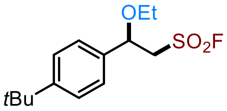

2-(4-(*tert*-Butyl)phenyl)-2-ethoxyethane-1-sulfonyl fluoride (7c):

25 mg, 88% yield, colorless oil.

^{1}H NMR (400 MHz, $CDCl_3$) δ 7.41 (d, J = 8.2 Hz, 2H), 7.27 (d, J = 8.1 Hz, 2H), 4.87 (dd, J = 10.0, 3.2 Hz, 1H), 3.80 (dd, J = 14.8, 10.0 Hz, 1H), 3.61 – 3.31 (m, 3H), 1.32 (s, 9H), 1.21 (t, J = 7.0 Hz, 3H);

^{19}F NMR (376 MHz, $CDCl_3$) δ 60.9 (d, J = 6.6 Hz);

^{13}C NMR (101 MHz, $CDCl_3$) δ 152.2, 135.1 (d, J = 2.7 Hz), 126.2, 126.1, 75.9, 65.2, 58.5 (d, J = 12.7 Hz), 34.8, 31.4, 15.1;

HRMS (ESI) *m/z* calcd. for $C_{14}H_{21}FNaO_3S$ $[M+Na]^+$ 311.1088, found 311.1083.

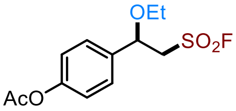

4-(1-Ethoxy-2-(fluorosulfonyl)ethyl)phenyl acetate (7d):

24 mg, 83% yield, colorless oil.

^{1}H NMR (500 MHz, $CDCl_3$) δ 7.38 (d, J = 8.5 Hz, 2H), 7.14 (d, J = 8.5 Hz, 2H), 4.89 (dd, J = 9.9, 3.0 Hz, 1H), 3.79 (dd, J = 14.8, 9.9 Hz, 1H), 3.55 – 3.42 (m, 3H), 2.31 (s, 3H), 1.21 (t, J = 7.0 Hz, 3H);

^{19}F NMR (471 MHz, $CDCl_3$) δ 61.1 (d, J = 7.6 Hz).

^{13}C NMR (126 MHz, $CDCl_3$) δ 169.5, 151.2, 135.7, 127.6, 122.5 , 75.6, 65.4, 58.4 (J = 13.1 Hz), 21.7, 15.0;

HRMS (ESI) *m/z* calcd. for $C_{12}H_{15}FNaO_5S$ $[M+Na]^+$ 313.0516, found 313.0519.

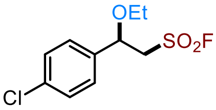

2-(4-Chlorophenyl)-2-ethoxyethane-1-sulfonyl fluoride (7e):

20 mg, 76% yield, colorless oil.

^{1}**H NMR** (400 MHz, $CDCl_3$) δ 7.39 (d, J = 7.6 Hz, 2H), 7.31 (d, J = 7.6 Hz, 2H), 4.87 (d, J = 9.6 Hz, 1H), 3.79 (dd, J = 14.4, 10.0 Hz, 1H), 3.56 – 3.38 (m, 3H), 1.21 (t, J = 7.2, 3H);

^{19}**F NMR** (376 MHz, $CDCl_3$) δ 61.1 (d, J = 8.1 Hz);

^{13}**C NMR** (101 MHz, $CDCl_3$) δ 136.7 (d, J = 1.9 Hz), 135.1, 129.5, 127.9, 75.6, 65.4, 58.2 (d, J = 13.5 Hz), 15.0;

HRMS (ESI) *m/z* calcd. for $C_{10}H_{12}ClFNaO_3S$ $[M+Na]^+$ 289.0072, found 289.0077.

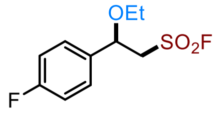

2-Ethoxy-2-(4-fluorophenyl)ethane-1-sulfonyl fluoride (7f):

20 mg, 76% yield, colorless oil.

^{1}**H NMR** (500 MHz, $CDCl_3$) δ 7.37 – 7.32 (m, 2H), 7.13 – 7.07 (m, 2H), 4.87 (dd, J = 9.7, 3.2 Hz, 1H), 3.83 – 3.77 (dd, J =14.8 Hz, 9.6Hz, 1H), 3.55 – 3.48 (m, 1H), 3.47 – 3.41 (m, 2H), 1.21 (t, J = 7.0 Hz, 3H);

^{19}**F NMR** (471 MHz, $CDCl_3$) δ 61.1 (d, J = 7.3 Hz, 1F), -112.1 – -112.3 (m, 1F);

^{13}**C NMR** (126 MHz, $CDCl_3$) δ 163.2 (d, J = 248.2 Hz), 134.0, 128.3 (d, J = 8.4 Hz), 116.3 (d, J = 21.4 Hz), 75.6, 65.3, 58.4 (d, J =13.2 Hz), 15.0;

HRMS (ESI) *m/z* calcd. for $C_{10}H_{12}F_2NaO_3S$ $[M+Na]^+$ 273.0367, found 273.0371.

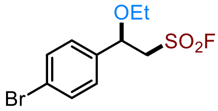

2-(4-Bromophenyl)-2-ethoxyethane-1-sulfonyl fluoride (7g):

20 mg, 76% yield, colorless oil.

^{1}**H NMR** (500 MHz, $CDCl_3$) δ 7.56 – 7.52 (m, 2H), 7.27-7.23 (m, 2H), 4.87 (dd, J = 9.7, 3.2 Hz, 1H), 3.78 (dd, J =14.8 Hz, 9.7 Hz, 1H), 3.54 – 3.48 (m, 1H), 3.48 – 3.42 (m, 2H), 1.21 (t, J = 7.0 Hz, 3H);

^{19}**F NMR** (471 MHz, $CDCl_3$) δ 61.2 (d, J = 7.3 Hz);

^{13}**C NMR** (126 MHz, $CDCl_3$) δ 137.3, 132.5, 128.2, 123.3, 75.6, 65.5, 58.1(d, J = 13.4Hz), 15.0;

HRMS (ESI) *m/z* calcd. for $C_{10}H_{12}BrFNaO_3S$ $[M+Na]^+$ 332.9567, found 332.9572.

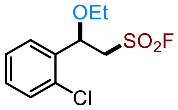

2-(2-Chlorophenyl)-2-ethoxyethane-1-sulfonyl fluoride (7h):

22 mg, 41% yield on 0.2 mmol scale, colorless oil.

^{1}**H NMR** (500 MHz, $CDCl_3$) δ 7.57 (dd, J = 7.7, 1.7 Hz, 1H), 7.43 – 7.30 (m, 3H), 5.33 (dd, J = 9.9, 2.7 Hz, 1H), 3.72 – 3.60 (m, 2H), 3.56 – 3.50 (m, 2H), 1.26 (t, J = 7.0 Hz, 3H);

^{19}**F NMR** (471 MHz, $CDCl_3$) δ 61.4 (d, J = 7.5 Hz).

^{13}**C NMR** (126 MHz, $CDCl_3$) δ 135.7, 134.7, 132.5, 130.2, 127.9, 127.4, 71.8, 66.0, 56.6 (d, J = 13.7 Hz), 15.1;

HRMS (ESI) *m/z* calcd. for $C_{10}H_{12}ClFNaO_3S$ $[M+Na]^+$ 289.0072, found 289.0077.

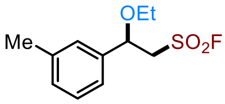

2-Ethoxy-2-(m-tolyl)ethane-1-sulfonyl fluoride (7i):

16 mg, 65% yield, colorless oil.

^{1}**H NMR** (500 MHz, $CDCl_3$) δ 7.29 (t, J = 7.9 Hz, 1H), 7.20 – 7.12 (m, 3H), 4.85 (dd, J = 10.0, 2.9 Hz, 1H), 3.81 (dd, J = 14.8, 10.0 Hz, 1H), 3.56 – 3.40 (m, 3H), 2.37 (s, 3H), 1.21 (t, J = 7.0 Hz, 3H);

^{19}**F NMR** (471 MHz, $CDCl_3$) δ 61.0 (d, J = 7.4 Hz);

^{13}**C NMR** (126 MHz, $CDCl_3$) δ 139.1, 138.2, 130.0, 129.1, 127.1, 123.5, 76.2, 65.2, 58.5 (d, J = 12.7 Hz), 21.6, 15.1;

HRMS (ESI) *m/z* calcd. for $C_{11}H_{15}FNaO_3S$ $[M+Na]^+$ 269.0618, found 269.0615.

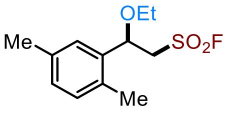

2-(2,5-Dimethylphenyl)-2-ethoxyethane-1-sulfonyl fluoride (7j):

24 mg, 92% yield, colorless oil.

^{1}**H NMR** (400 MHz, $CDCl_3$) δ 7.24 (s, 1H), 7.06 – 7.03 (m, 2H), 5.03 (dd, J = 9.9, 2.5 Hz, 1H), 3.67 (dd, J = 14.9, 10.0 Hz, 1H), 3.44 – 3.32 (m, 2H), 2.26 (s, 3H), 2.25 (s, 3H), 1.15 (t, J = 7.0 Hz, 3H);

^{19}**F NMR** (376 MHz, $CDCl_3$) δ 60.4 (d, J = 6.9 Hz);

^{13}**C NMR** (101 MHz, $CDCl_3$) δ 136.6, 135.9 (d, J = 2.2 Hz), 132.0, 131.1, 129.5, 126.6, 73.0, 65.2, 57.4 (d, J = 12.7 Hz), 21.2, 18.5, 15.1;

HRMS (ESI) *m/z* calcd. for $C_{12}H_{17}FNaO_3S$ $[M+Na]^+$ 283.0775, found 283.0779.

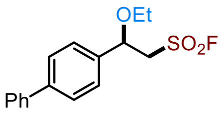

2-([1,1'-Biphenyl]-4-yl)-2-ethoxyethane-1-sulfonyl fluoride (7k):

26 mg, 84% yield, colorless oil.

^{1}HNMR (500MHz, $CDCl_3$) δ 7.64 – 7.61 (m, 2H), 7.60 – 7.57 (m, 2H), 7.48 – 7.42 (m, 4H), 7.39 – 7.36 (m, 1H), 4.96 – 4.90 (dd, J = 9.9, 3.0 Hz, 1H), 3.85 (dd, J = 14.8 Hz, 9.9 Hz, 1H), 3.60 – 3.55 (m, 1H), 3.54 – 3.45 (m, 2H), 1.24 (t, J = 7.0 Hz, 3H);

^{19}F NMR (471 MHz, $CDCl_3$) δ 61.1 (d, J = 7.2 Hz);

^{13}C NMR (126 MHz, CDCl3) δ 142.2, 140.4, 137.1, 129.0, 128.0, 127.8, 127.3, 127.0, 76.0, 65.3, 58.4 (d, J =12.8 Hz), 15.2;

HRMS (ESI) *m/z* calcd. for $C_{16}H_{17}FNaO_3S$ $[M+Na]^+$ 331.0775, found 331.0779.

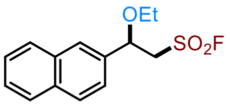

2-Ethoxy-2-(naphthalen-2-yl)ethane-1-sulfonyl fluoride (7l):

24 mg, 48% yield on 0.2 mmol scale, colorless oil.

^{1}H NMR (400 MHz, $CDCl_3$) δ 7.94 – 7.80 (m, 4H), 7.57 – 7.49 (m, 2H), 7.45 (d, J = 8.7 Hz, 1H), 5.06 (dd, J = 9.8, 3.1 Hz, 1H), 3.91 (dd, J = 14.8, 9.8 Hz, 1H), 3.66 – 3.57 (m, 1H), 3.54 – 3.46 (m, 2H), 1.24 (t, J = 8.8 Hz, 3H);

^{19}F NMR (376 MHz, $CDCl_3$) δ 61.0 (d, J = 6.7 Hz);

^{13}C NMR (101 MHz, $CDCl_3$) δ 135.5 (d, J = 2.2 Hz), 133.7, 133.3, 129.5, 128.2, 128.0, 126.9, 126.9, 126.2, 123.4, 76.4, 65.4, 58.4 (d, J =13.2 Hz), 15.1;

HRMS (ESI) *m/z* calcd. for $C_{14}H_{15}FNaO_3S$ $[M+Na]^+$ 305.0618, found 305.0613.

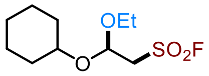

2-Cyclohexyl-2-ethoxyethane-1-sulfonyl fluoride (7m):

17 mg, 67% yield, colorless oil.

^{1}H NMR (500 MHz, $CDCl_3$) δ 5.15 (t, J = 5.4 Hz, 1H), 3.76 – 3.50 (m, 5H), 1.88 (d, J = 15.2 Hz, 2H), 1.79 – 1.72 (m, 2H), 1.57 – 1.49 (m, 1H), 1.48 – 1.28 (m, 5H), 1.24 (t, J = 7.0 Hz, 3H);

^{19}F NMR (471 MHz, $CDCl_3$) δ 60.6;

^{13}C NMR (126 MHz, $CDCl_3$) δ 95.6, 76.6, 62.1, 55.3 (d, J = 14.0 Hz), 33.1, 32.0, 25.5, 24.1, 23.9, 15.2;

HRMS (ESI) *m/z* calcd. for $C_{10}H_{19}FNaO_4S$ $[M+Na]^+$ 277.0880, found 277.0883.

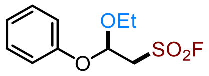

2-Ethoxy-2-phenoxyethane-1-sulfonyl fluoride (7n):

20 mg, 82% yield, colorless oil.

^{1}H NMR (500 MHz, $CDCl_3$) δ 7.37 – 7.30 (m, 2H), 7.14 – 7.03 (m, 3H), 5.72 (dd, J = 5.8, 4.8 Hz, 1H), 3.90 – 3.79 (m, 3H), 3.70 – 3.63 (m, 1H), 1.24 (t, J = 7.1 Hz, 3H);

^{19}F NMR (471 MHz, $CDCl_3$) δ 60.8;

^{13}C NMR (126 MHz, $CDCl_3$) δ 155.9, 130.0, 123.8, 118.1, 97.1 , 64.0, 54.6 (d, J = 15.7 Hz), 15.0;

HRMS (ESI) *m/z* calcd. for $C_{10}H_{13}FNaO_4S$ $[M+Na]^+$ 271.0411, found 271.0416.

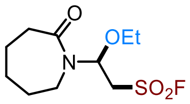

2-Ethoxy-2-(2-oxoazepan-1-yl)ethane-1-sulfonyl fluoride (7o):

24 mg, 88% yield, colorless oil.

^{1}H NMR (500 MHz, $CDCl_3$) δ 6.25 (dd, J = 8.2, 4.5 Hz, 1H), 3.64 (dd, J = 14.4, 8.2Hz, 1H), 3.61 – 3.56 (m, 3H), 3.39 – 3.28 (m, 2H), 2.65 – 2.51 (m, 2H),1.81 – 1.68 (m, 4H), 1.66 – 1.60 (m, 2H), 1.22 (t, J = 7.1 Hz, 3H);

^{19}F NMR (471 MHz, $CDCl_3$) δ 61.2 (d, J = 7.2 Hz);

^{13}C NMR (126 MHz, $CDCl_3$) δ 176.9, 77.8, 65.1, 53.7 (d, J = 15.3 Hz), 41.7, 37.7, 30.1, 29.4, 23.5, 14.8;

HRMS (ESI) *m/z* calcd. for $C_{10}H_{18}FNNaO_4S$ $[M+Na]^+$ 290.0833, found 290.0839.

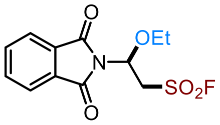

2-(1,3-Dioxoisoindolin-2-yl)-2-ethoxyethane-1-sulfonyl fluoride (7p):

27 mg, 89% yield, colorless oil.

^{1}H NMR (500 MHz, $CDCl_3$) δ 7.94 – 7.91 (m, 2H), 7.83 – 7.79 (m, 2H), 5.94 (t, J = 6.0Hz, 1H), 4.49 – 4.42 (m, 1H), 4.33 – 4.26 (m, 1H), 3.67 – 3.61 (m, 2H), 1.23 (t, J = 7.1 Hz, 3H);

^{19}F NMR (471 MHz, $CDCl_3$) δ 59.5 (d, J = 7.3 Hz);

^{13}C NMR (126 MHz, $CDCl_3$) δ 167.4, 135.0, 131.3, 124.3, 75.0, 65.8, 52.7 (d, J = 17.0 Hz),14.7;

HRMS (ESI) *m/z* calcd. for $C_{12}H_{12}FNNaO_5S$ $[M+Na]^+$ 324.0312, found 324.0315.

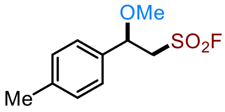

2-Methoxy-2-(p-tolyl)ethane-1-sulfonyl fluoride (7q):

20 mg, 45% yield on 0.2 mmol scale, colorless oil.

^{1}H NMR (500 MHz, $CDCl_3$) δ 7.25 – 7.20 (m, 4H), 4.74 (dd, J = 9.9, 3.1 Hz, 1H), 3.81 (dd, J = 14.8, 9.8 Hz, 1H), 3.56 – 3.49 (m, 1H), 3.29 (s, 3H), 2.37 (s, 3H);

^{19}F NMR (471 MHz, $CDCl_3$) δ 60.9 (d, J = 7.2 Hz);

^{13}C NMR (126 MHz, $CDCl_3$) δ 139.4, 134.3, 130.0, 126.6, 77.9, 58.3 (d, J = 13.3 Hz), 57.1, 21.3;

HRMS (ESI) *m/z* calcd. for $C_{10}H_{13}FNaO_3S$ $[M+Na]^+$ 255.0462, found 255.0458.

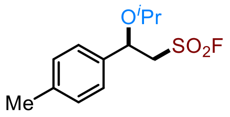

2-Isopropoxy-2-(p-tolyl)ethane-1-sulfonyl fluoride (7r):

15 mg, 58% yield, colorless oil.

^{1}H NMR (400 MHz, $CDCl_3$) δ 7.25 (d, J = 6.3 Hz, 2H), 7.20 (d, J = 7.9 Hz, 2H), 4.96 (dd, J = 9.9, 2.9 Hz, 1H), 3.77 (dd, J = 14.7, 9.9 Hz, 1H), 3.63 – 3.54 (m, 1H), 3.51 – 3.44 (m, 1H), 2.36 (s, 3H), 1.20 (d, J = 6.0 Hz, 3H), 1.07 (d, J = 6.2 Hz, 3H);

^{19}F NMR (376 MHz, $CDCl_3$) δ 61.0 (d, J = 6.8 Hz);

^{13}C NMR (101 MHz, $CDCl_3$) δ 139.0, 136.1 (d, J = 2.2 Hz), 129.8, 126.5, 73.7, 70.7, 58.8 (d, J = 12.3 Hz), 23.3, 21.3, 21.0;

HRMS (ESI) *m/z* calcd. for $C_{12}H_{17}FNaO_3S$ $[M+Na]^+$ 283.0775, found 283.0770.

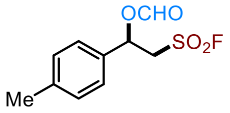

2-(Fluorosulfonyl)-1-(p-tolyl)ethyl formate (7s):

18 mg, 72% yield, colorless oil.

^{1}H NMR (500 MHz, $CDCl_3$) δ 7.28 (d, J = 8.2 Hz, 2H), 7.22 (d, J = 8.1 Hz, 2H), 6.40 (dd, J = 9.7, 3.2 Hz, 1H), 4.07 – 4.01 (m, 1H), 3.75 – 3.69 (m, 1H), 2.36 (s, 3H);

^{19}F NMR (471 MHz, $CDCl_3$) δ 60.1 (d, J = 5.1 Hz);

^{13}C NMR (126 MHz, $CDCl_3$) δ 159.0, 140.1, 132.9 (d, J = 1.8 Hz), 130.1, 126.5, 69.2, 56.0 (d, J = 16.1 Hz), 21.4;

HRMS (ESI) *m/z* calcd. for $C_{10}H_{11}FNaO_4S$ $[M+Na]^+$ 269.0254, found 269.0257.

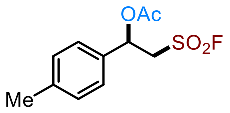

2-(Fluorosulfonyl)-1-(p-tolyl)ethyl acetate (7t):

14 mg, 55% yield, colorless oil.

^{1}H NMR (400 MHz, $CDCl_3$) δ 7.27 (d, J = 8.0 Hz, 2H), 7.21 (d, J = 8.1 Hz, 2H), 6.29 (dd, J = 9.8, 3.3 Hz, 1H), 4.02 – 3.94 (m, 1H), 3.71 – 3.66 (m, 1H), 2.35 (s, 3H), 2.11 (s, 3H);

^{19}F NMR (376 MHz, $CDCl_3$) δ 59.7 (dd, J = 5.5, 3.0 Hz);

^{13}C NMR (101 MHz, $CDCl_3$) δ 169.2, 139.7, 133.5, 130.0, 126.5, 69.4, 56.1 (d, J = 15.4 Hz), 21.3, 20.9;

HRMS (ESI) *m/z* calcd. for $C_{11}H_{13}FNaO_4S$ $[M+Na]^+$ 283.0411, found 283.0415.

Mechanistic studies

TEMPO trapping experiment:

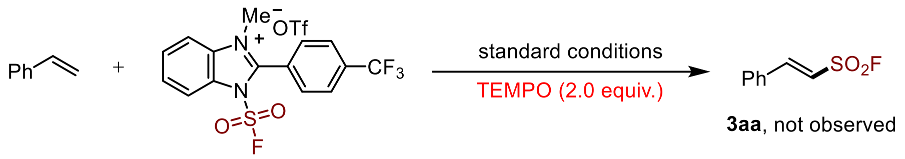

2e, 2.0 equiv.

3aa, not observed

The TEMPO trapping experiment was carried out according to the general procedure A on 0.1 mmol scale with 2.0 equivalents of TEMPO. After 12 hours, the reaction mixture was passed through a short silica gel for GC-MS analysis. Product **3aa** was not observed.

Radical clock experiment:

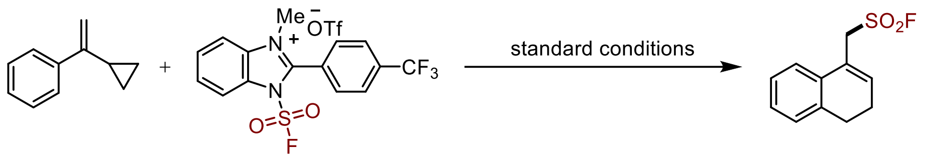

8

2e, 2.0 equiv.

9, 21% yield

Racical clock experiment was carried out with (1-cyclopropylvinyl)benzene **8** according to general procedure A on 0.2 mmol scale and product was isolated through a silica gel flash column as colorless oil (10 mg, 21% yield).

(3,4-Dihydronaphthalen-1-yl)methanesulfonyl fluoride (9)：

^{1}H NMR (400 MHz, $CDCl_3$) δ 7.27 – 7.17 (m, 4H), 6.40 (s, 1H), 4.49 (s, 2H), 2.83 (t, J = 8.4 Hz, 2H), 2.46 – 2.38 (m, 2H);

^{19}F NMR (376 MHz, $CDCl_3$) δ 54.3;

^{13}C NMR (126 MHz, $CDCl_3$) δ 136.7 (d, J = 2.0 Hz), 136.2, 128.24, 128.23, 128.21, 126.9, 123.7, 122.7 (d, J = 1.4 Hz), 54.2 (d, J = 17.4 Hz), 27.5, 23.6;

HRMS (EI) *m/z* calcd. for $C_{11}H_{11}FO_2S$ $[M]^+$ 226.0464, found 226.0468.

Cationic intermediate trapping experiment:

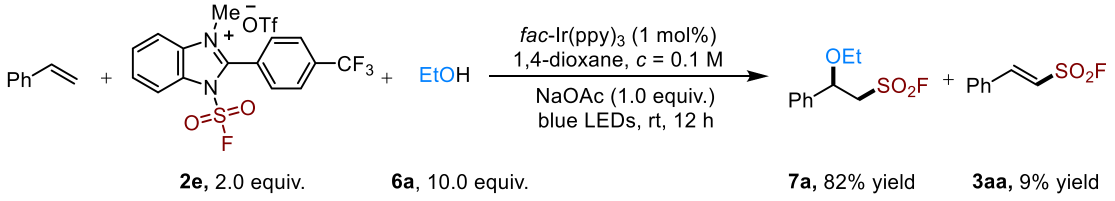

Cationic intermediate trapping experiment using FS-BIT as a radical precursor was carried out according to the general procedure B. The product **7a** was isolated through a silica gel flash column as olorless oil. The product **3aa** was detected by ^{19}F NMR in 9% yield.

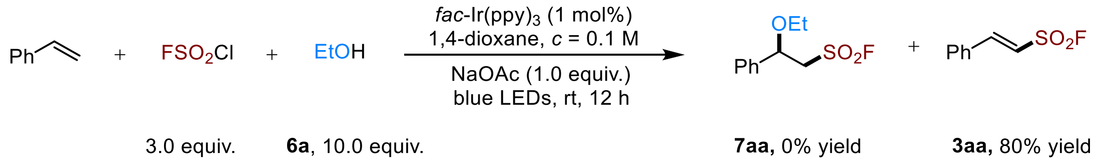

Cationic intermediate trapping experiment using FSO_2Cl as a radical precursor was carried out according to the general procedure B, The *fac*-Ir(ppy)$_3$ (0.64 mg, 1 mol%) and NaOAc (13.6 mg, 0.1 mmol) were weighed into an oven-dried Schlenk tube, followed by the addition of anhydrous 1,4-dioxane (0.8 mL, 0.1 M), styrene (11.5 μL, 0.1 mmol), EtOH (58 μL, 1 mmol), and FSO_2Cl (0.3 mmol, in anhydrous $PhCF_3$, 1.5 mol/L) under argon. The reaction mixture was allowed to stir at room temperature under irradiation with blue LEDs for 12 h. The product **3aa** was isolated through a silica gel flash column in 80% yield. Product **7a** was not observed.

Imidazole product identification:

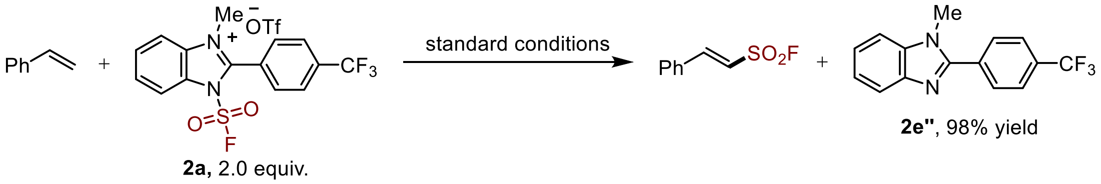

Imidazole product **2e''** was separated from the reaciton with styrene according to the general procedure A as white solid (27 mg, 98% yield).

1-Methyl-2-(4-(trifluoromethyl)phenyl)-1H-benzo[d]imidazole (2e'')：

^{1}H NMR (500 MHz, $CDCl_3$) δ 7.90 (d, J = 8.2 Hz, 2H), 7.87 – 7.82 (m, 1H), 7.78 (d, J = 8.3 Hz, 2H), 7.48 – 7.37 (m, 1H), 7.37 – 7.28 (m, 2H), 3.87 (s, 3H);

^{19}F NMR (376 MHz, $CDCl_3$) δ -62.8;

^{13}C NMR (126 MHz, $CDCl_3$) δ 152.2, 142.9, 136.7, 133.8, 131.7 (q, J = 33.4 Hz), 129.9, 125.8 (q, J = 4.0 Hz), 124.0 (q, J = 273.4 Hz), 123.5, 122.9, 120.2, 109.9, 31.9;

HRMS (EI) *m/z* calcd. for $C_{15}H_{11}F_3N_2$ $[M]^+$ 276.0874, found 276.0878.

Fluorescence quenching studies:

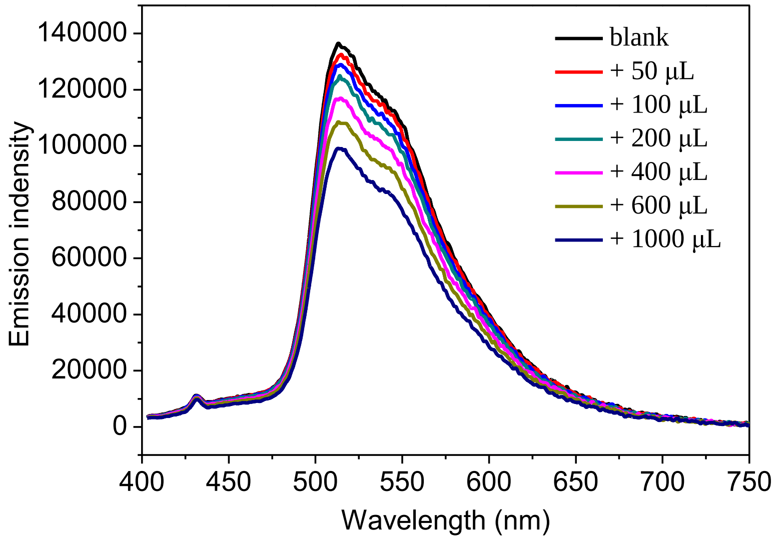

Supplementary Figure 8. Fluorescence titration of *fac*-Ir(ppy)$_3$ (20 μM in 1,4-dioxane) with blank sample (solvent 1,4-dioxane).

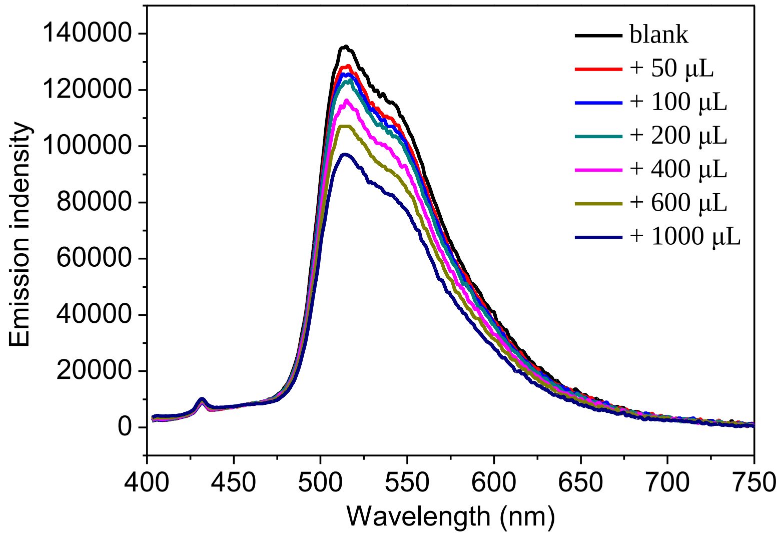

Supplementary Figure 9. Fluorescence titration of *fac*-Ir(ppy)$_3$ (20 μM in 1,4-dioxane) with upon titration with styrene **1a** (6 mM in 1,4-dioxane).

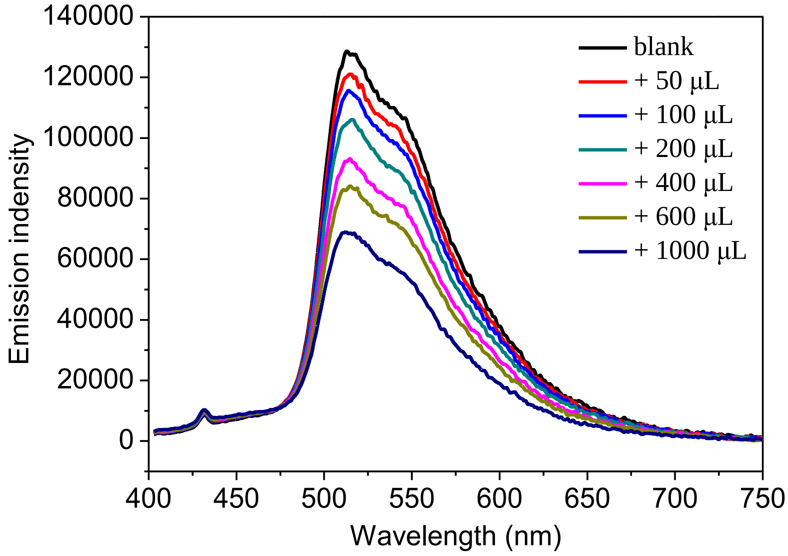

Supplementary Figure 10. Fluorescence titration of *fac*-Ir(ppy)$_3$ (20 µM in 1,4-dioxane) with upon titration with reagent **2e** (6 mM in 1,4-dioxane).

Supplementary Tables

Supplementary Table 1. Reaction condition optimization. [a]

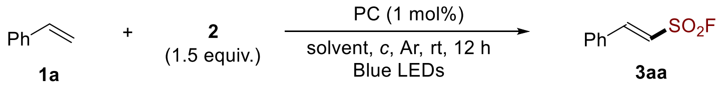

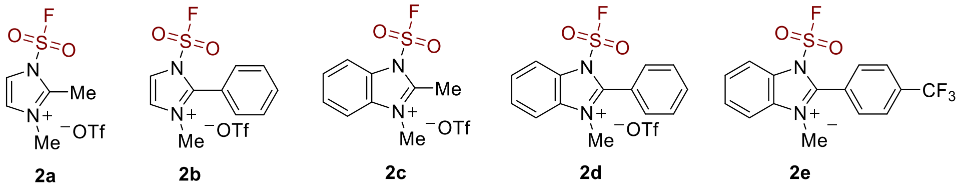

Entry	PC	Solvent	*c* (mM)	2	Yield of 3aa[b]	*E*:*Z* of 3aa[c]
1	*fac*-Ir(ppy)$_3$	1,4-dioxane	50	**2a**	N.P.	-
2	*fac*-Ir(ppy)$_3$	1,4-dioxane	50	**2b**	trace	-
3	*fac*-Ir(ppy)$_3$	1,4-dioxane	50	**2c**	trace	-
4	*fac*-Ir(ppy)$_3$	1,4-dioxane	50	**2d**	32%	94:6
5	*fac*-Ir(ppy)$_3$	1,4-dioxane	50	**2e**	51%	>20:1
6	*fac*-Ir(ppy)$_3$	THF	50	**2e**	40%	93:7
7	*fac*-Ir(ppy)$_3$	Et_2O	50	**2e**	38%	93:7
8	*fac*-Ir(ppy)$_3$	toluene	50	**2e**	31%	87:13
9	*fac*-Ir(ppy)$_3$	acetone	50	**2e**	18%	90:10
10	*fac*-Ir(ppy)$_3$	CH_2Cl_2	50	**2e**	27%	>20:1
11	*fac*-Ir(ppy)$_3$	CH_3CN	50	**2e**	trace	-
12	Ru(bpy)$_3$Cl$_2$•6H$_2$O	1,4-dioxane	50	**2e**	21%	95:5
13	Ir[dF(CF$_3$ppy)$_2$(dtbpy)]PF$_6$	1,4-dioxane	50	**2e**	18%	88:12
14	4CzIPN	1,4-dioxane	50	**2e**	43%	85:15
15	Rodamine 6G	1,4-dioxane	50	**2e**	12%	87:13
16[d]	*fac*-Ir(ppy)$_3$	1,4-dioxane	50	**2e**	30%	91:9
17[e]	*fac*-Ir(ppy)$_3$	1,4-dioxane	50	**2e**	76%	>20:1
18[f]	*fac*-Ir(ppy)$_3$	1,4-dioxane	50	**2e**	69%	>20:1
19[e]	*fac*-Ir(ppy)$_3$	1,4-dioxane	25	**2e**	94%	>20:1

20[e]	*fac*-Ir(ppy)3	1,4-dioxane	100	**2e**	63%	92:8
21[e]	No PC	1,4-dioxane	25	**2e**	N.P.	-
22[e]	*fac*-Ir(ppy)3, In dark	1,4-dioxane	25	**2e**	N.P.	-

[a]Reaction conditions: Styrene (**1a**, 0.1 mmol), **2**, PC (1 mol%), solvent, argon, blue LEDs (λ_{max} = 460 nm), room temperature for 12 h. [b]Yield determined by ^{19}F NMR spectroscopy using 4-fluoriodobenzene as an internal standard. [c]The *Z*/*E* ratio was determined by ^{19}F NMR spectroscopy. [d]**2e** (0.1 mmol). [e]**2e** (0.2 mmol). [f]**2e** (0.3 mmol).

Supplementary Table 2. Further Screening of the Fluorosulfonylation Reagent Type of 2.[a]

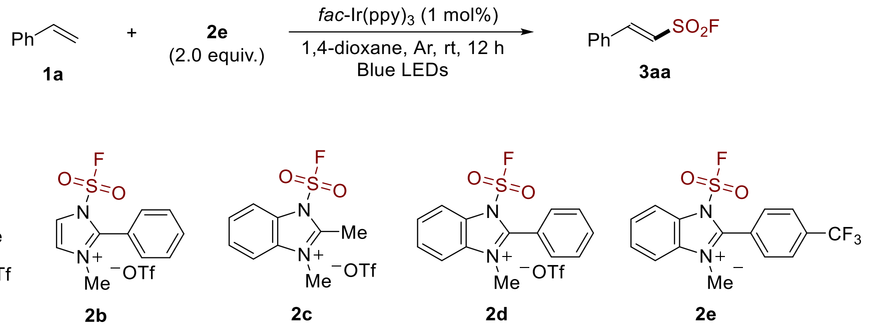

Entry	Fluorosulfonylation Reagent	Yield of 3aa[b]	*E*:*Z* of 3aa[c]
1	**2a**	N.P.	-
2	**2b**	trace	-
3	**2c**	trace	-
4	**2d**	72%	94:6
5	**2e**	94%	>20:1

[a]Reaction conditions: Styrene (**1a**, 0.1 mmol), **2** (0.2 mmol), *fac*-Ir(ppy)3 (1 mol%), 1,4-dioxane (4 mL), argon, blue LEDs (λ_{max} = 460 nm), room temperature for 12 h. [b]Yield determined by ^{19}F NMR spectroscopy using 4-fluoriodobenzene as an internal standard. [c]The Z/E ratio was determined by ^{19}F NMR spectroscopy.

Supplementary Figures

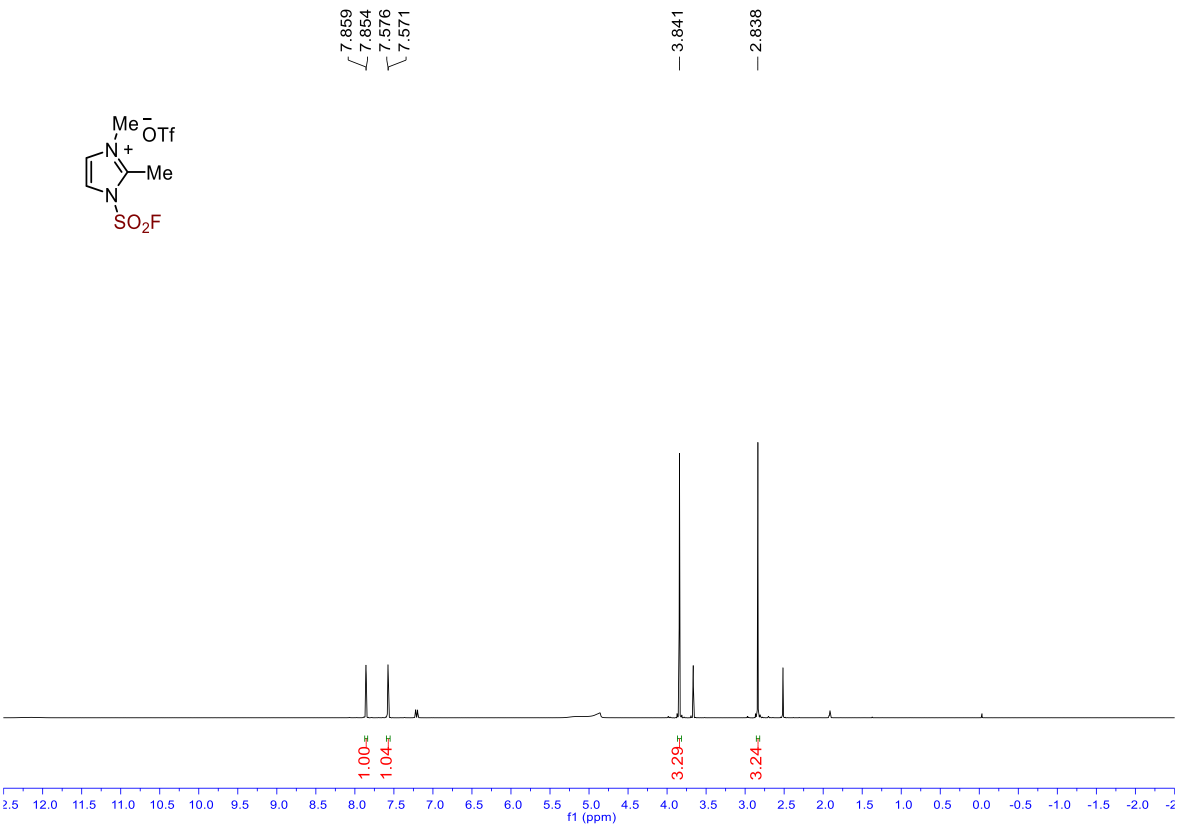

Supplementary Figure 11. ^{1}H NMR spectra of **2a**

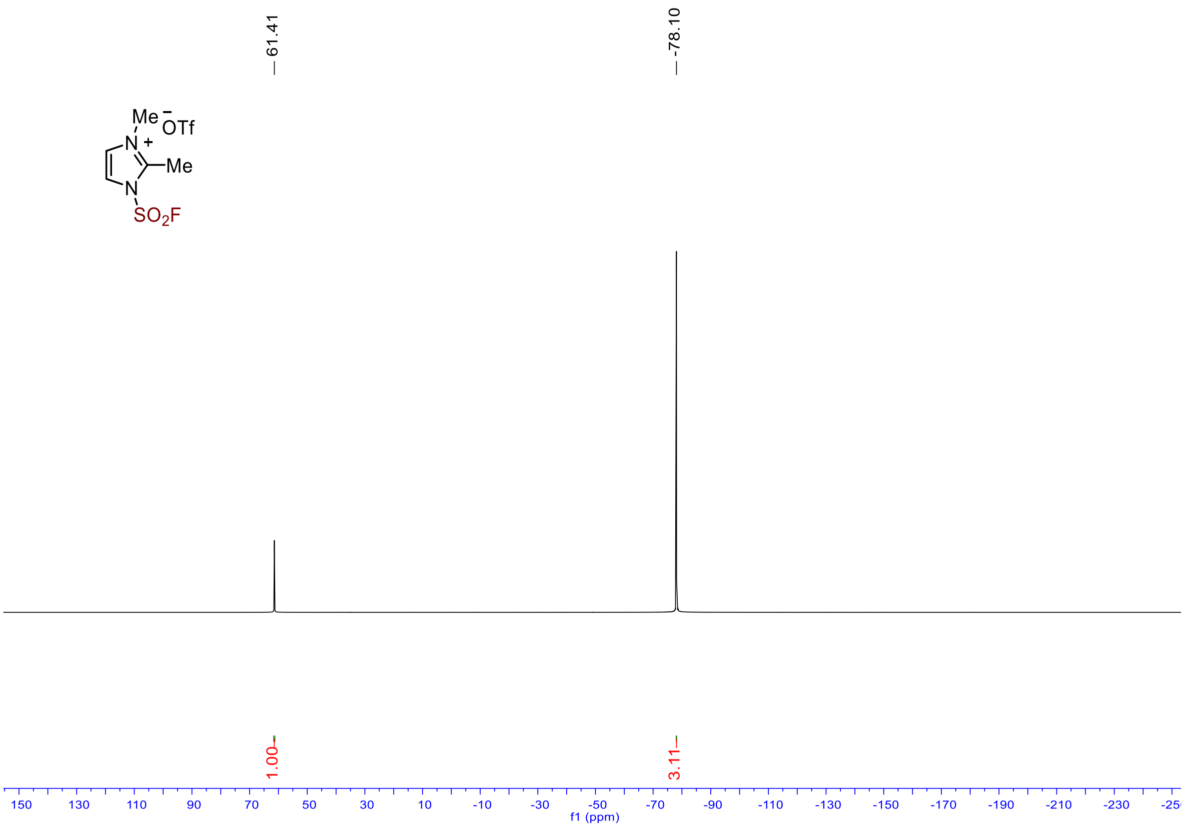

Supplementary Figure 12. ^{19}F NMR spectra of **2a**

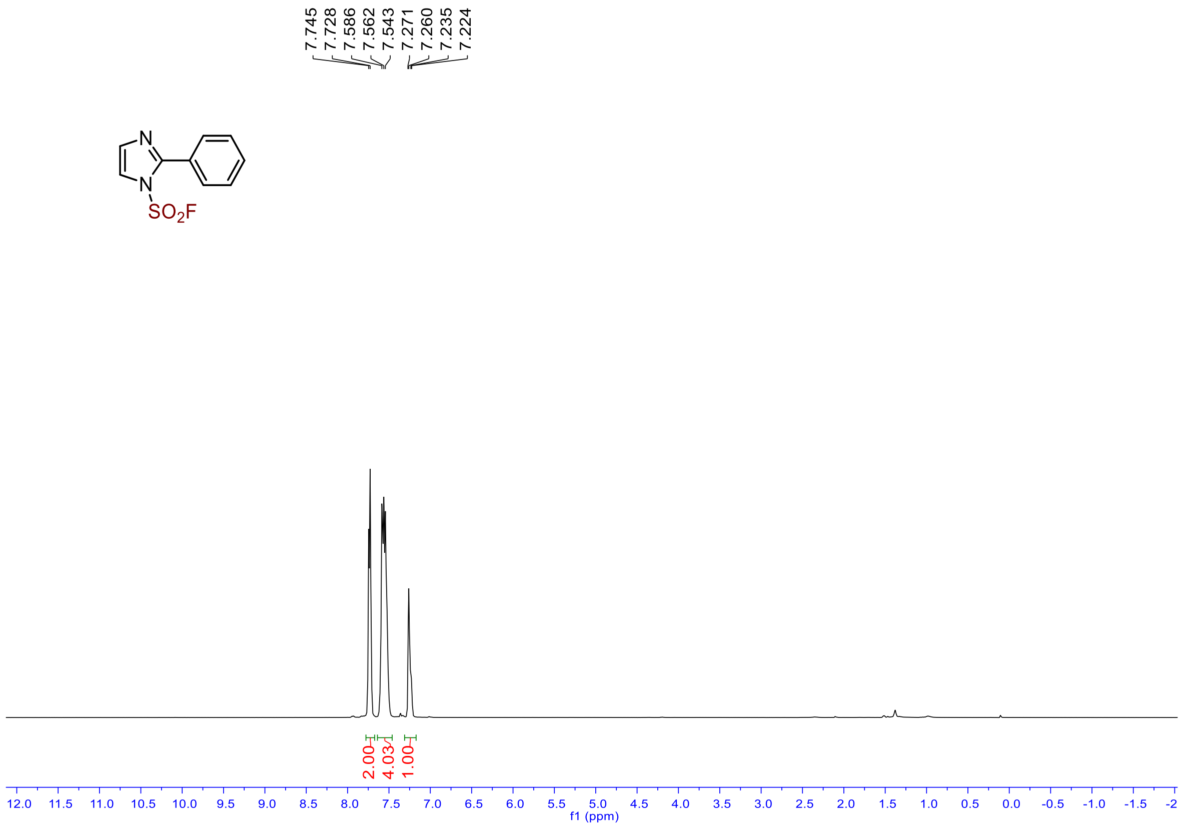

Supplementary Figure 13. ^{1}H NMR spectra of **S1**

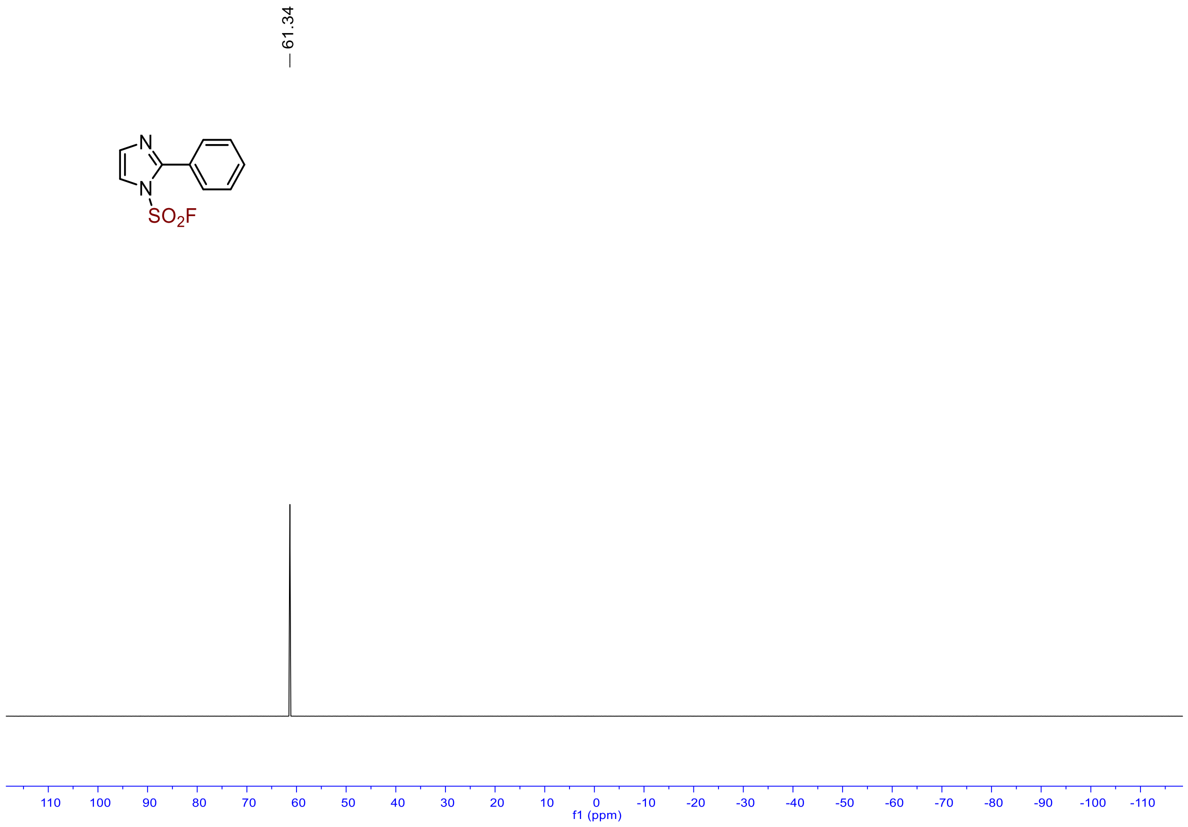

Supplementary Figure 14. ^{19}F NMR spectra of **S1**

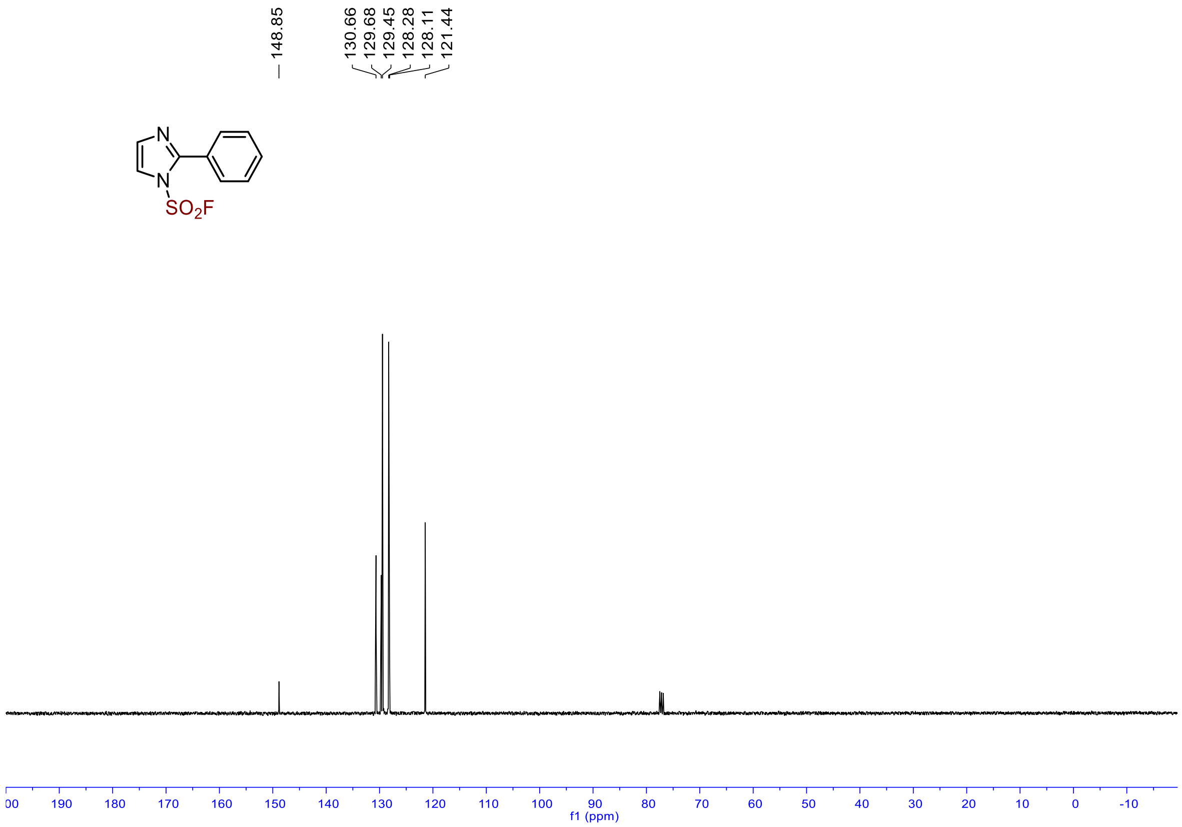

Supplementary Figure 15. ^{13}C NMR spectra of **S1**

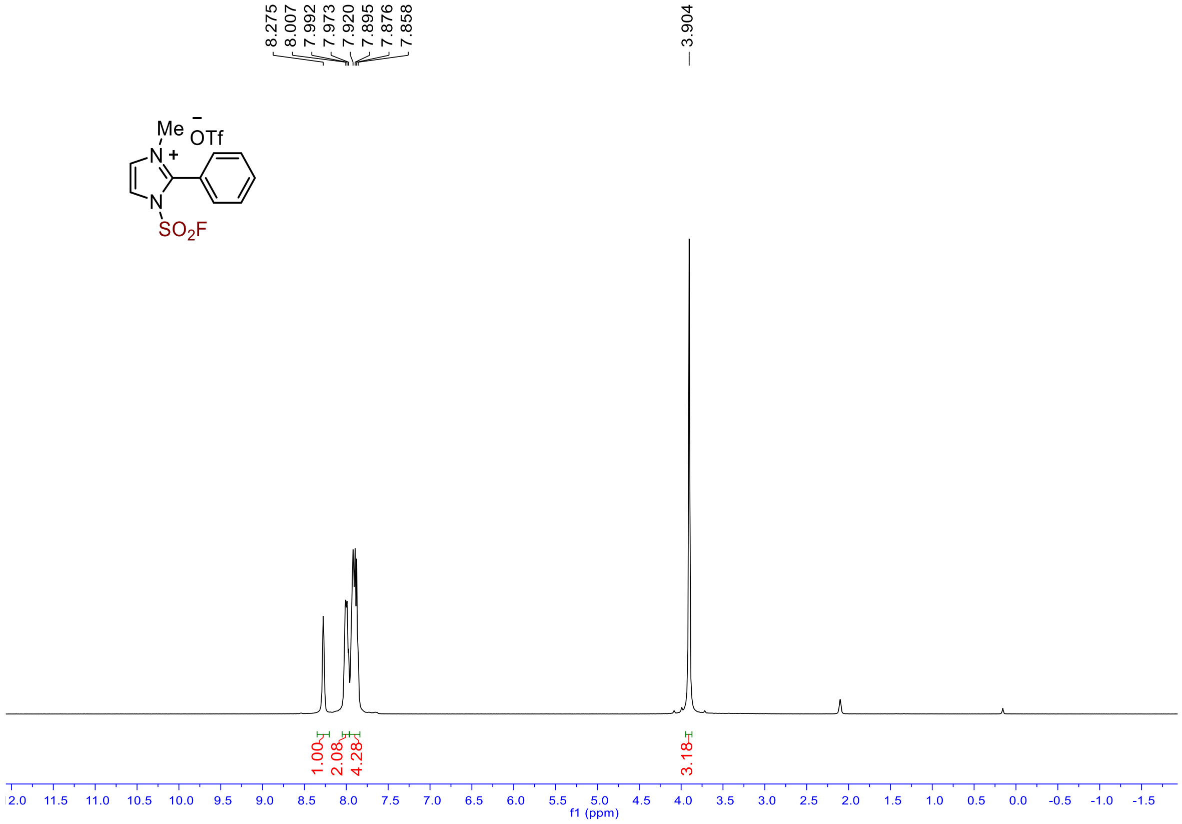

Supplementary Figure 16. ^{1}H NMR spectra of **2b**

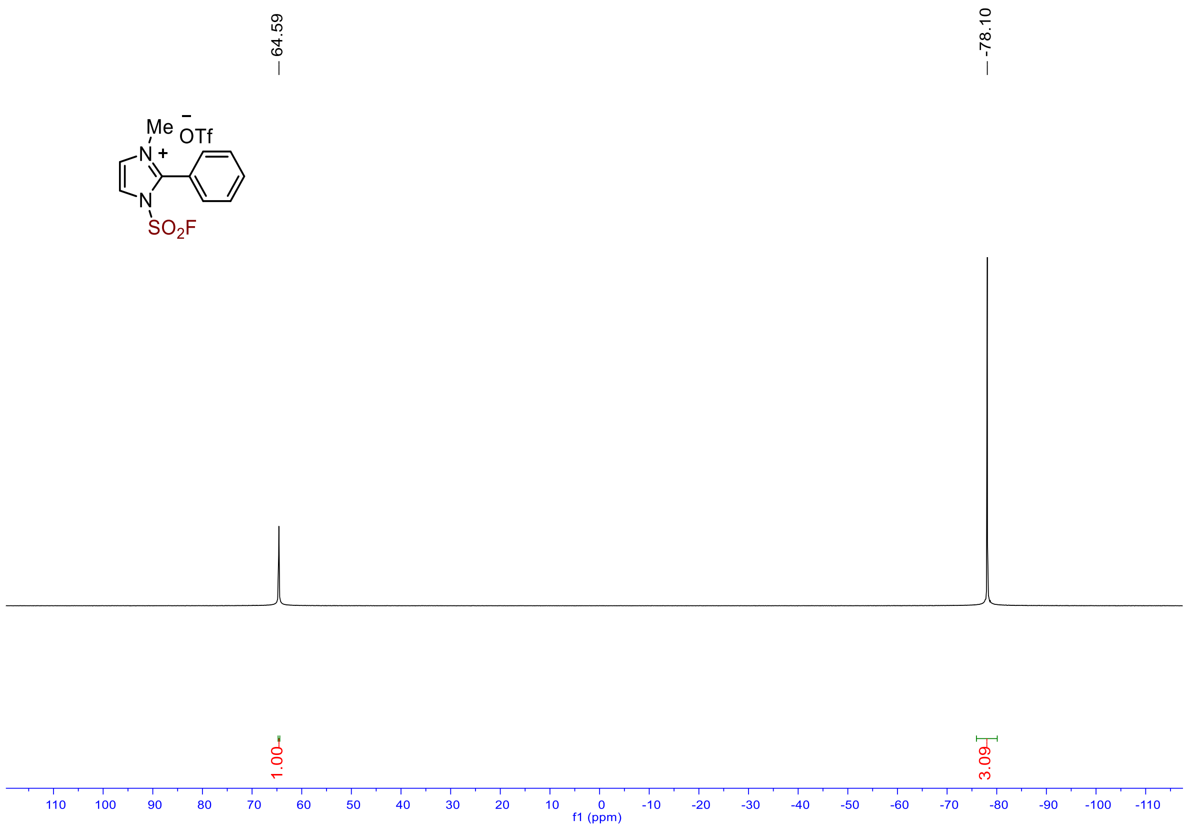

Supplementary Figure 17. ^{19}F NMR spectra of **2b**

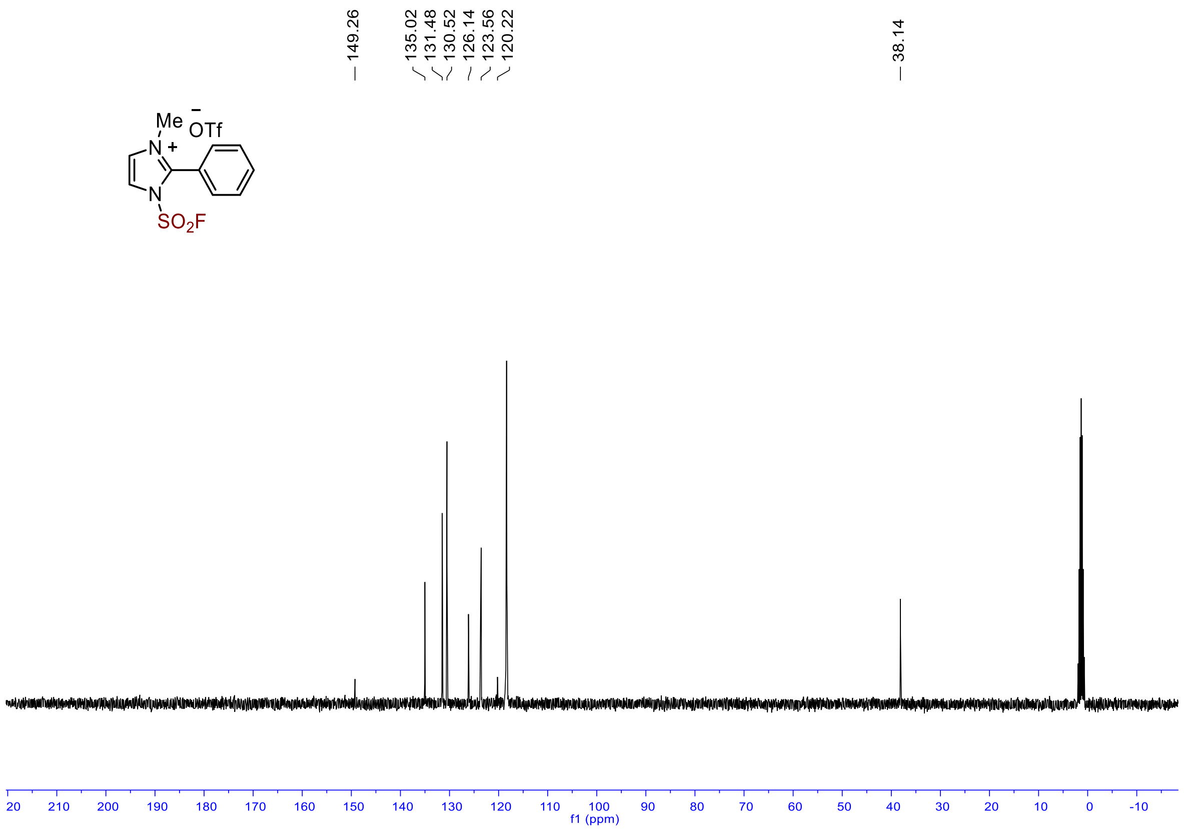

Supplementary Figure 18. ^{13}C NMR spectra of **2b**

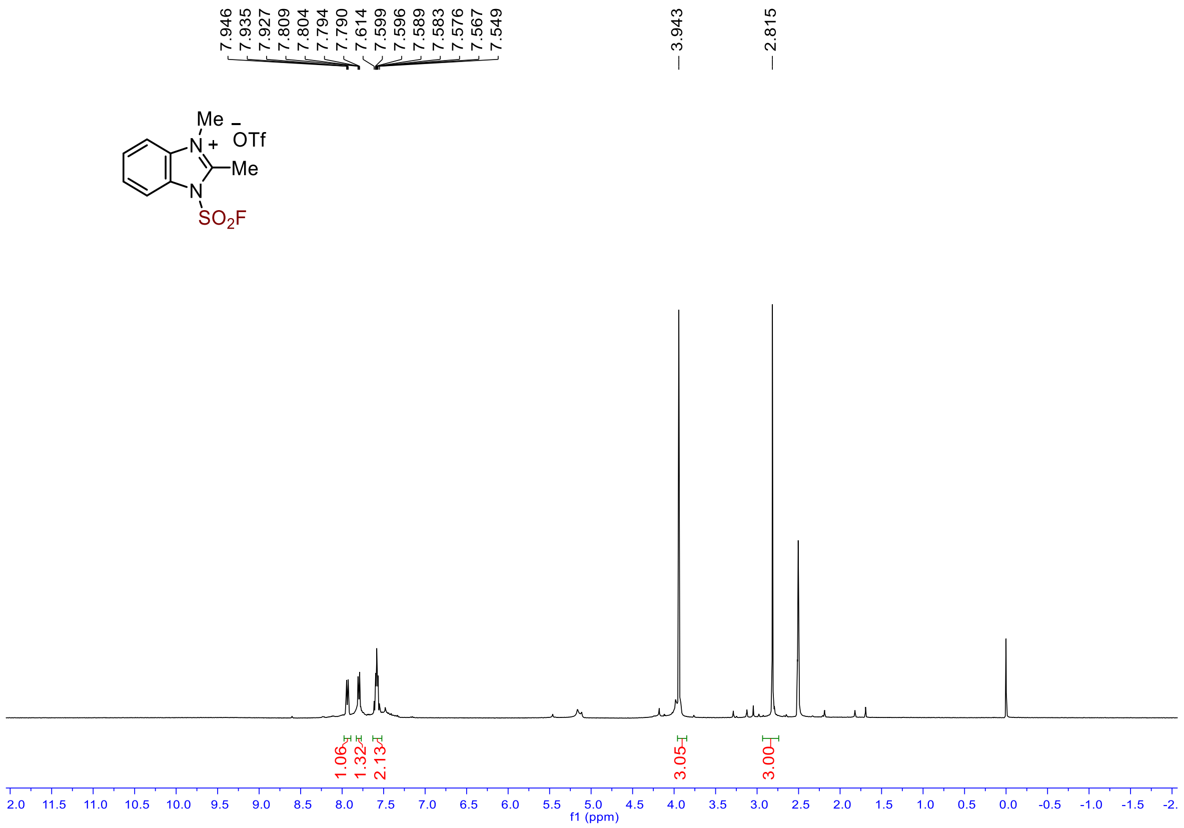

Supplementary Figure 19. ^{1}H NMR spectra of **2c**

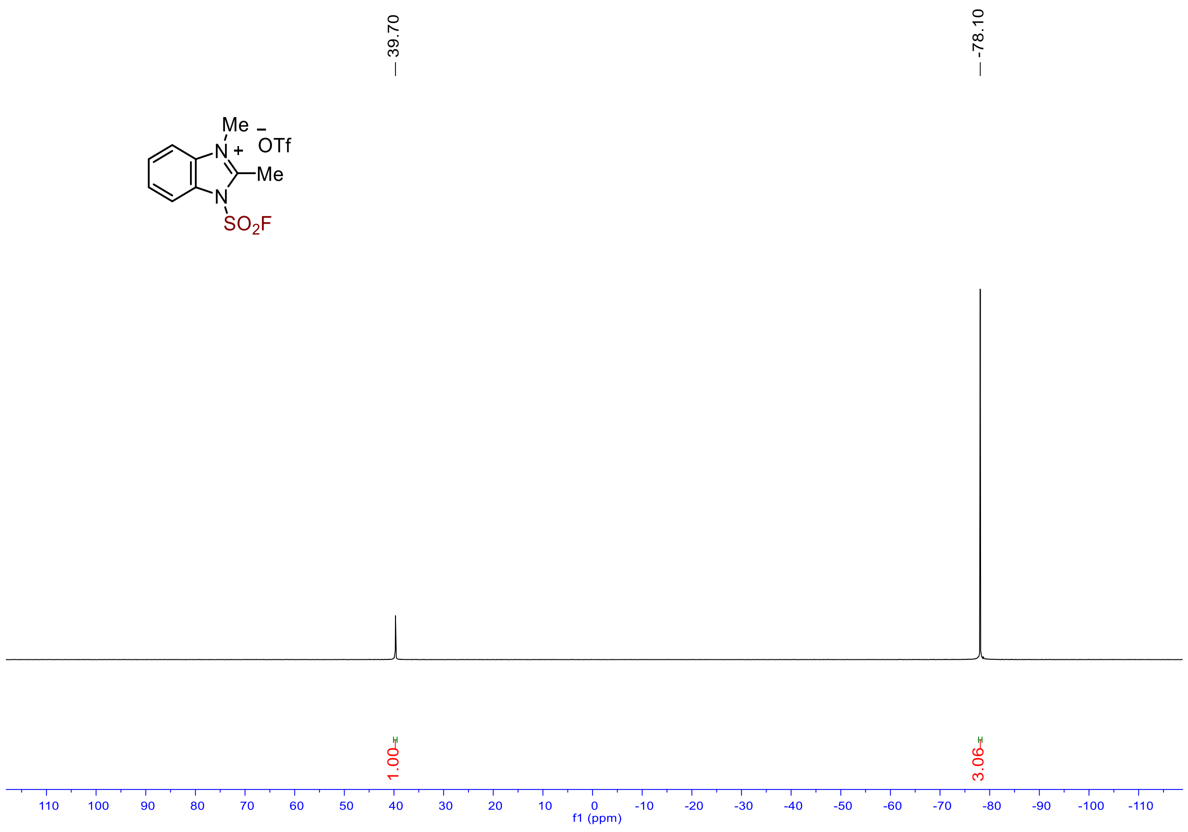

Supplementary Figure 20. ^{19}F NMR spectra of **2c**

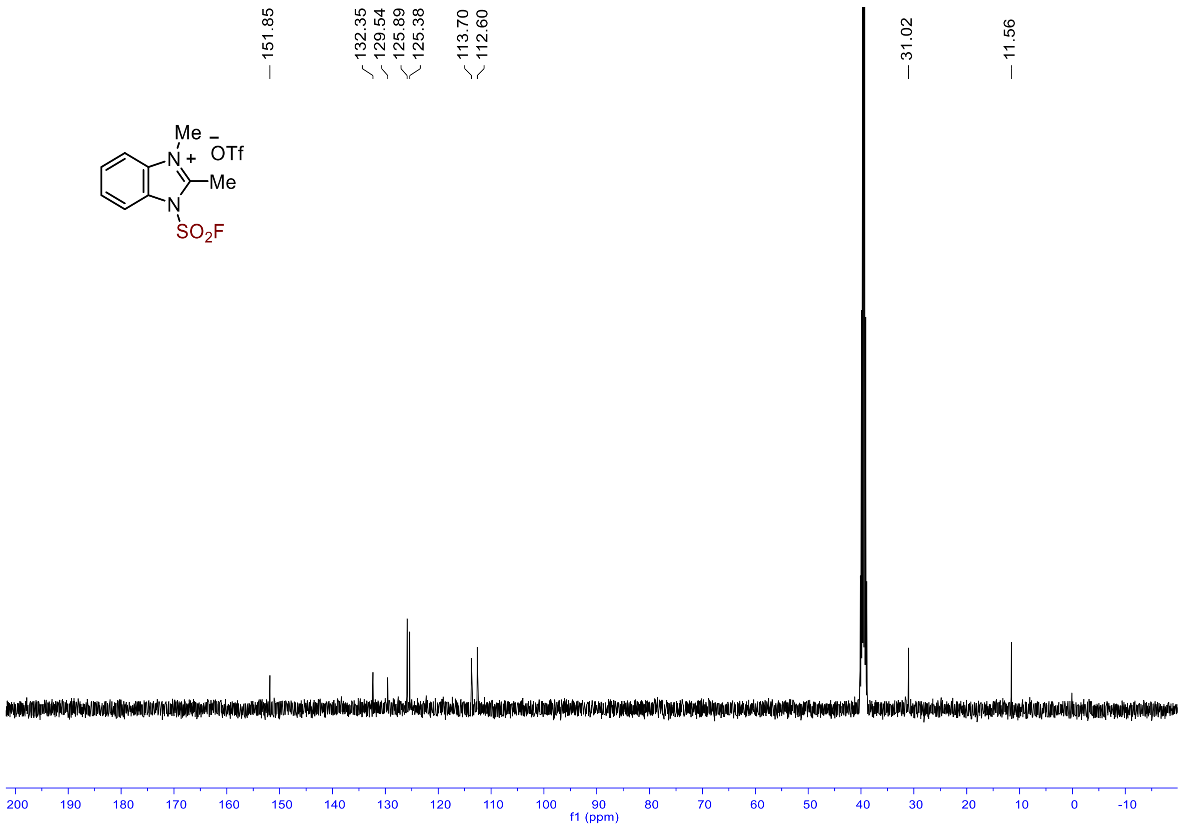

Supplementary Figure 21. ^{13}C NMR spectra of **2c**

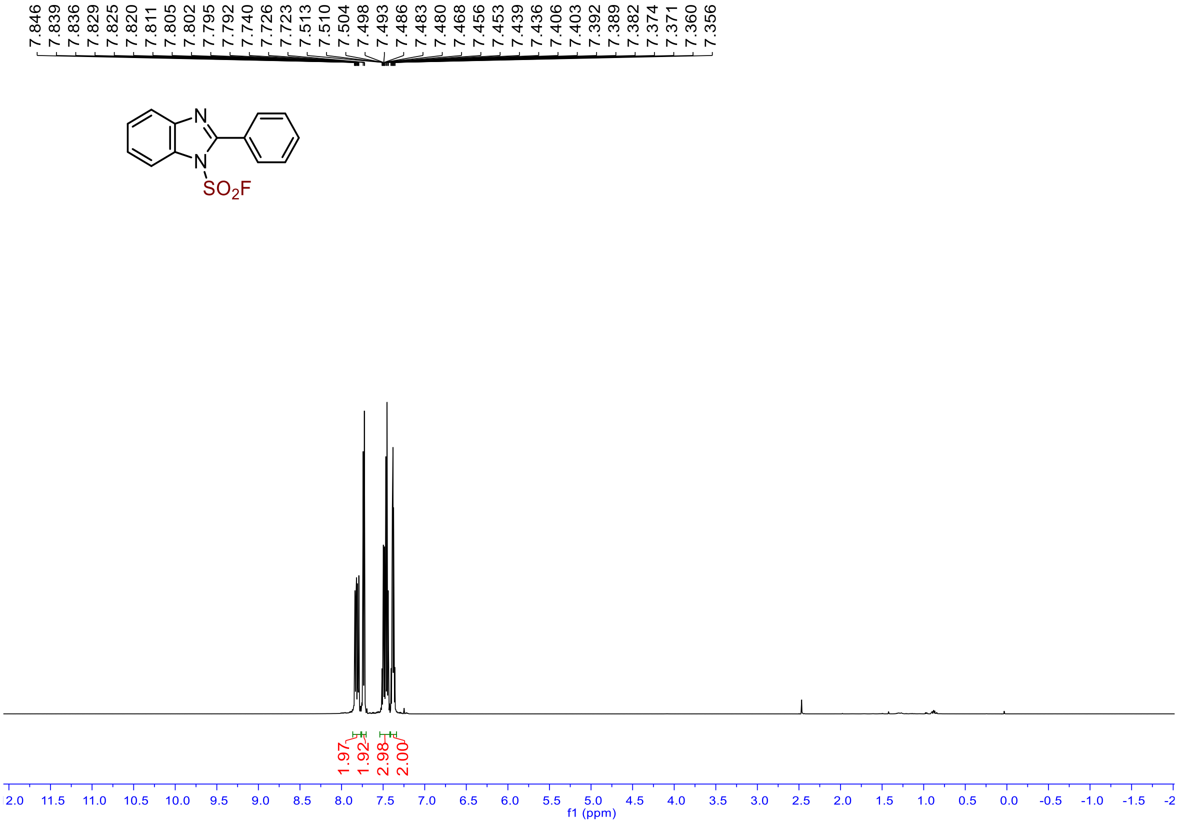

Supplementary Figure 22. ^{1}H NMR spectra of **S2**

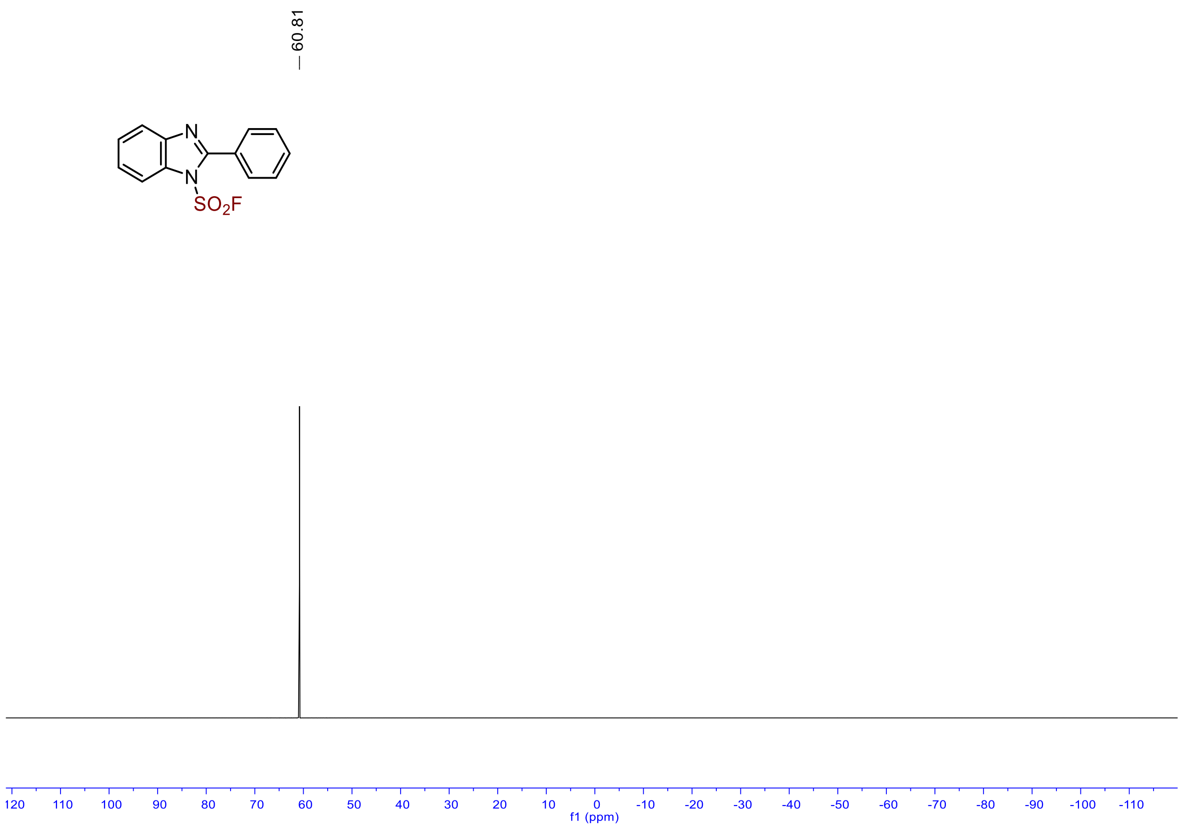

Supplementary Figure 23. ^{19}F NMR spectra of **S2**

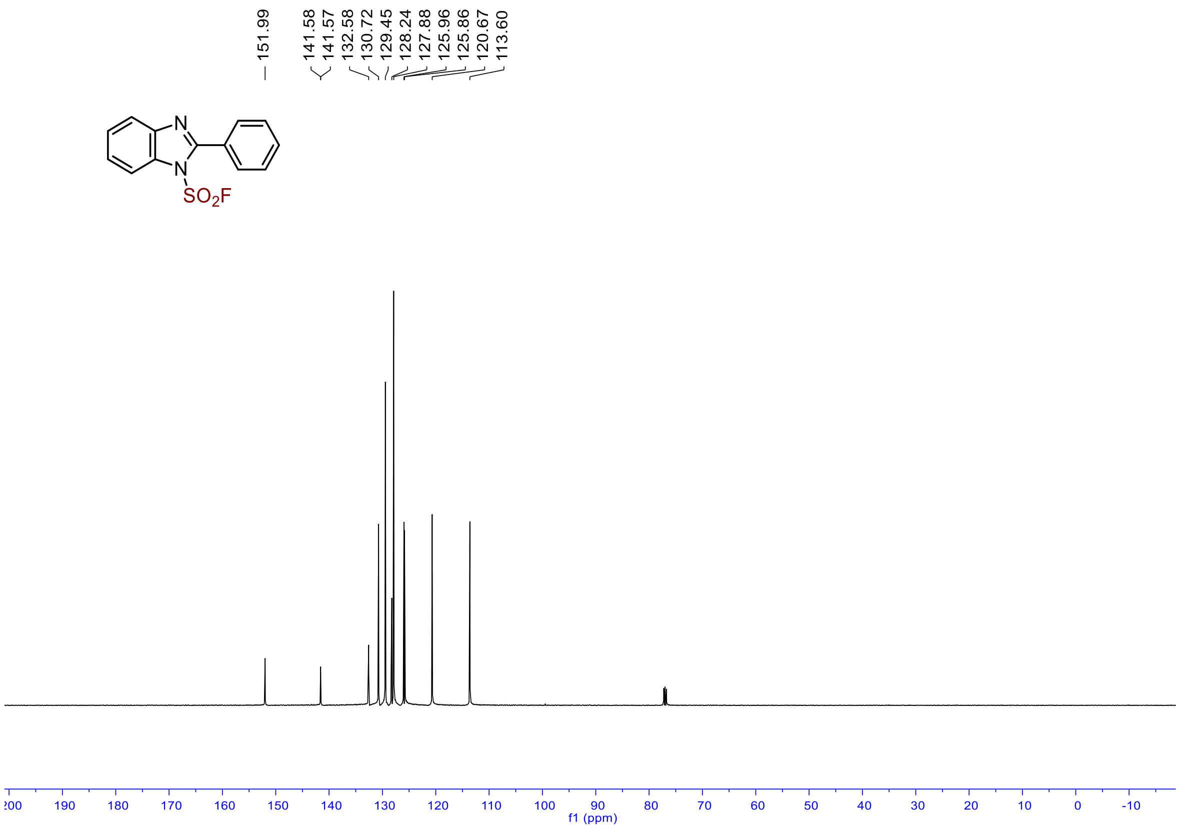

Supplementary Figure 24. ^{13}C NMR spectra of **S2**

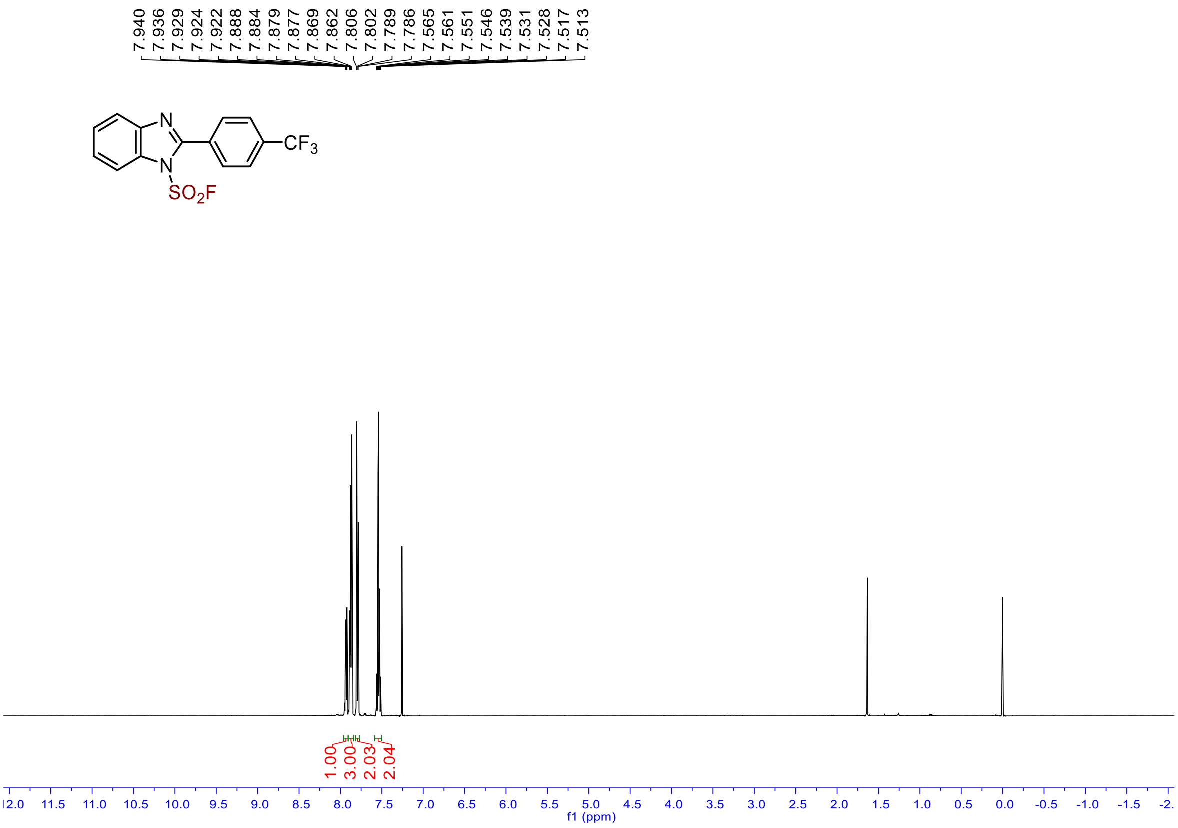

Supplementary Figure 25. ^{1}H NMR spectra of **S3**

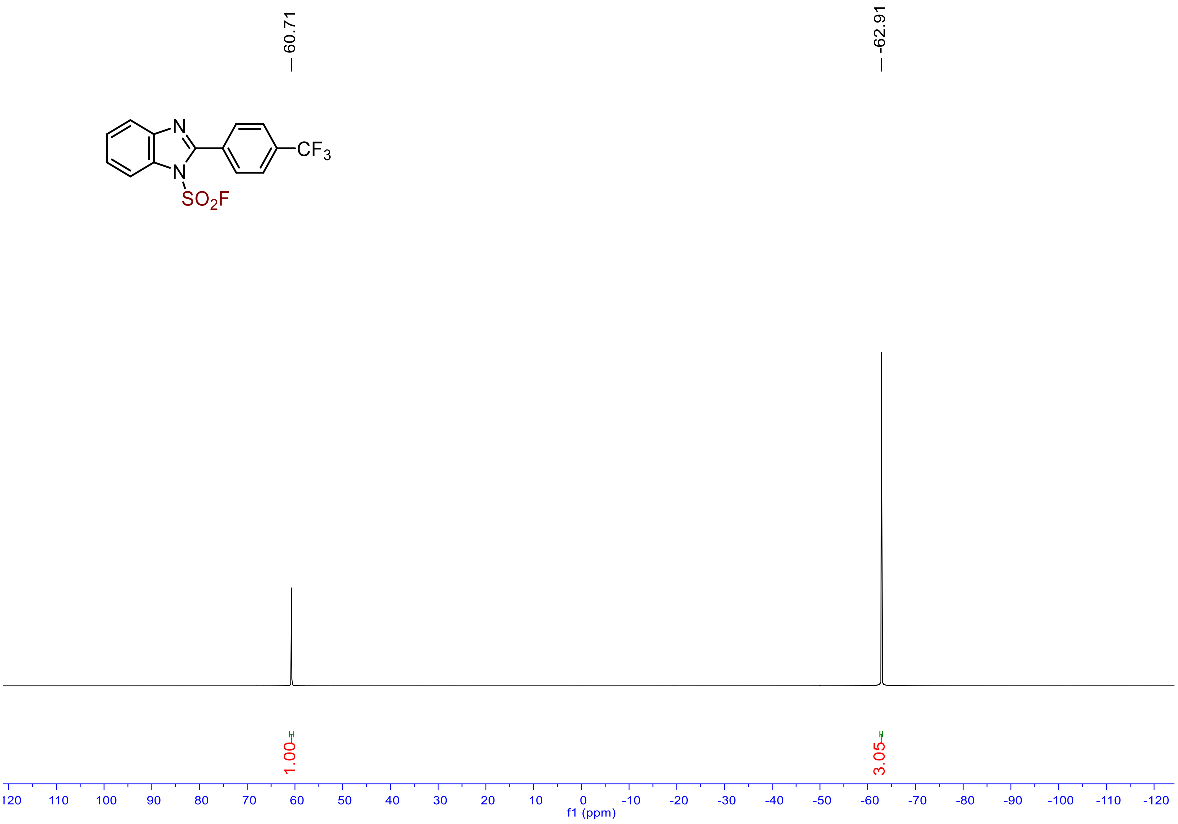

Supplementary Figure 26. ^{19}F NMR spectra of **S3**

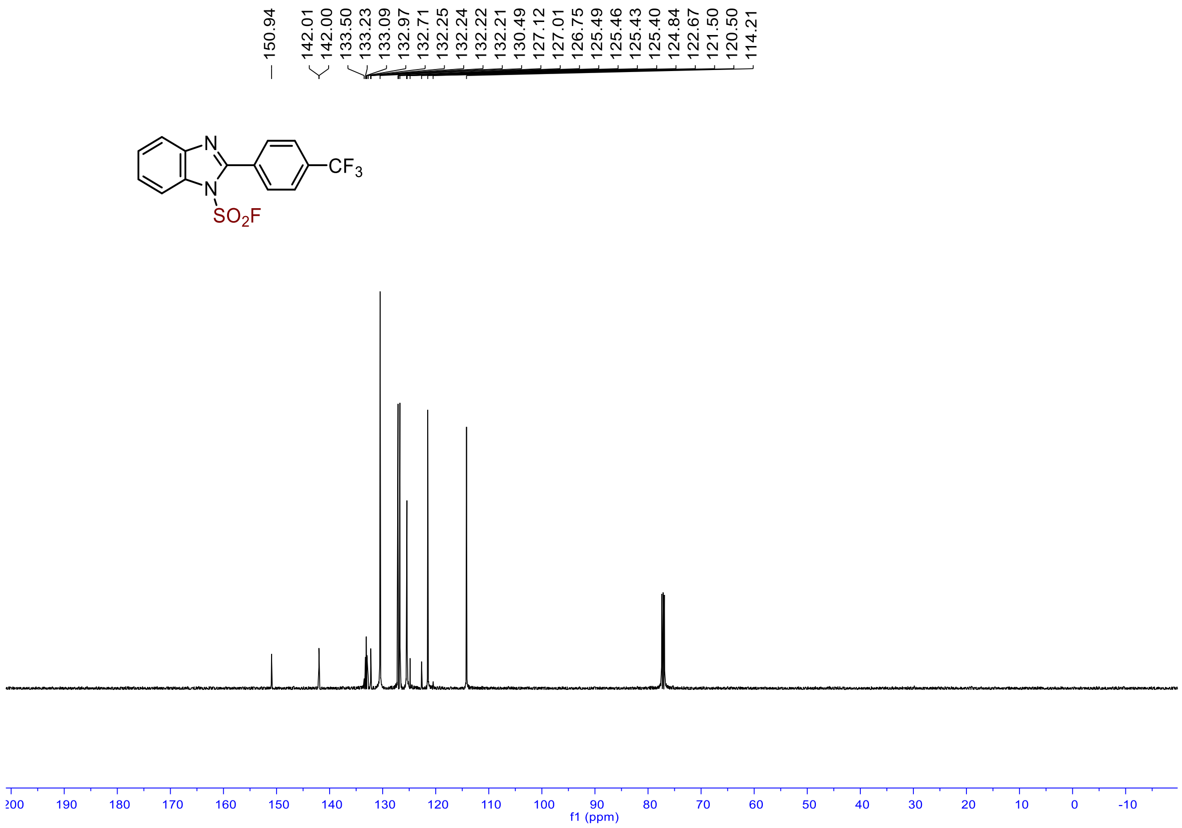

Supplementary Figure 27. ^{13}C NMR spectra of **S3**

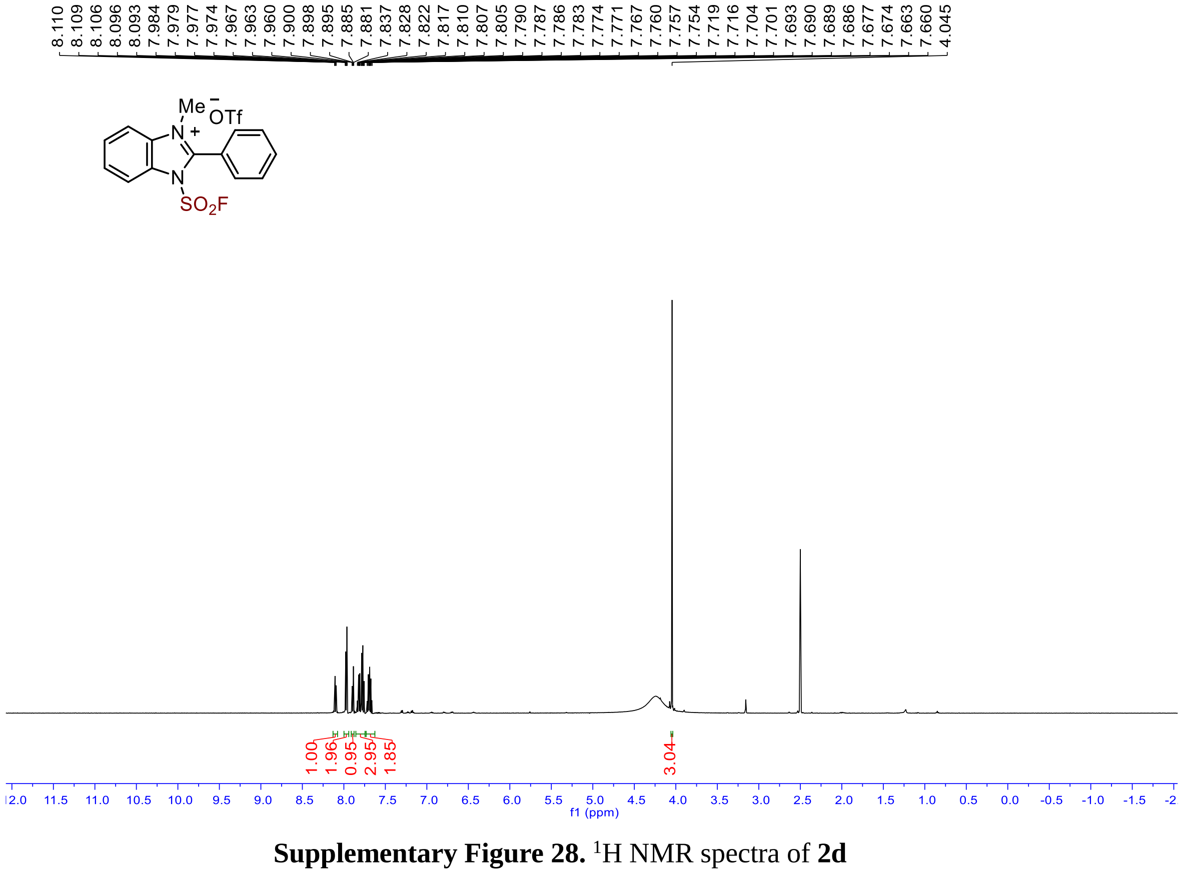

Supplementary Figure 28. ^{1}H NMR spectra of **2d**

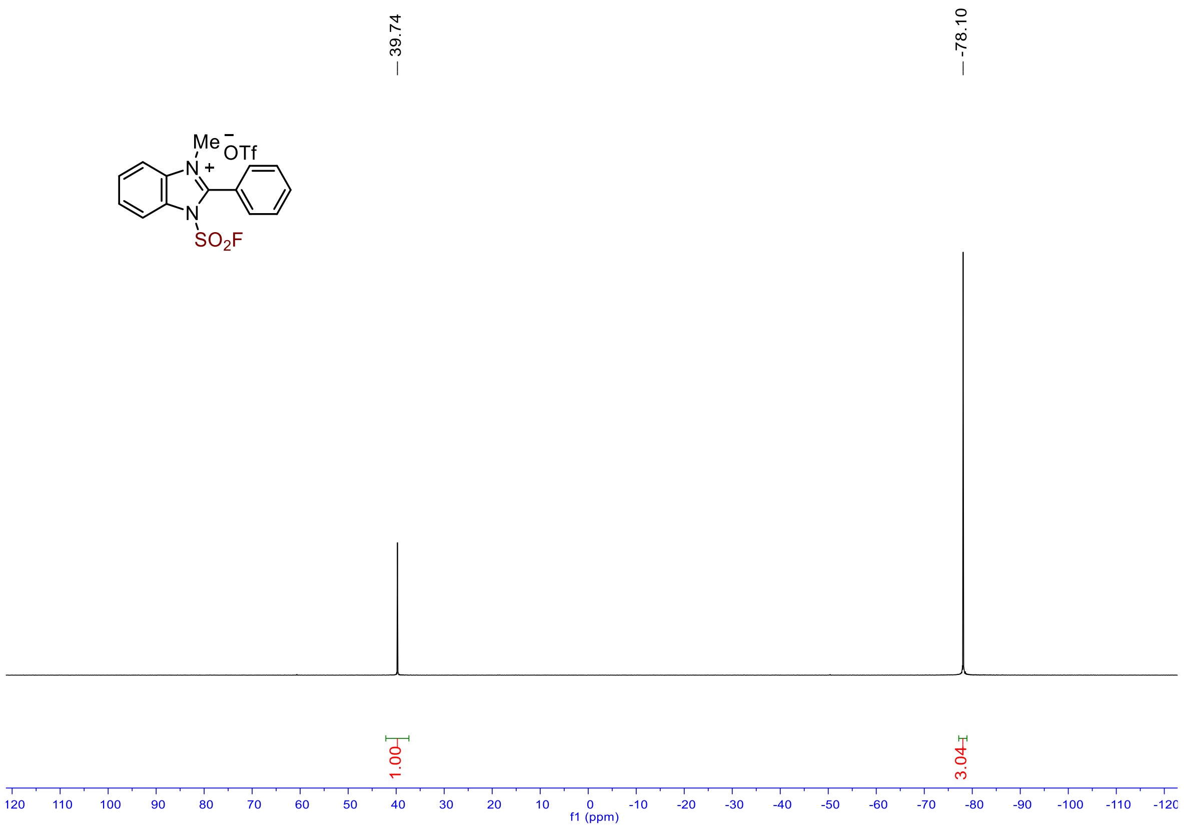

Supplementary Figure 29. ^{19}F NMR spectra of **2d**

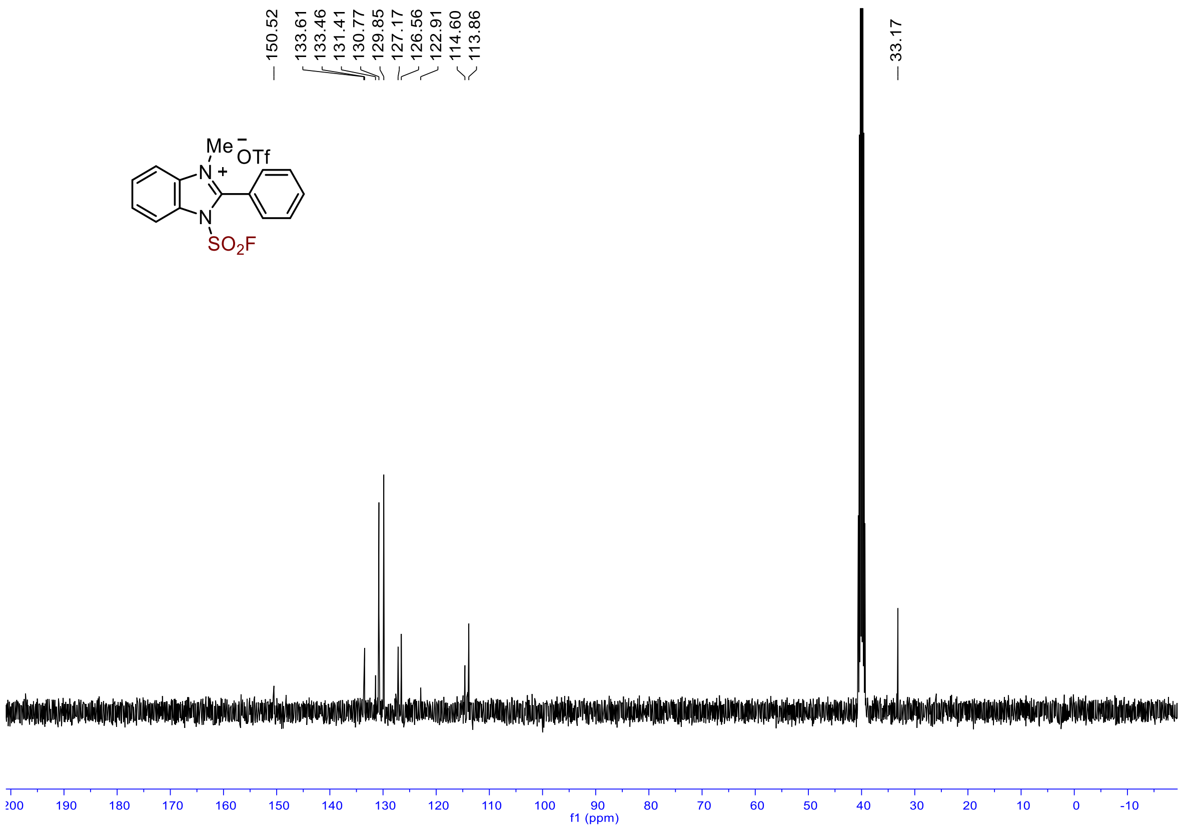

Supplementary Figure 30. ^{13}C NMR spectra of **2d**

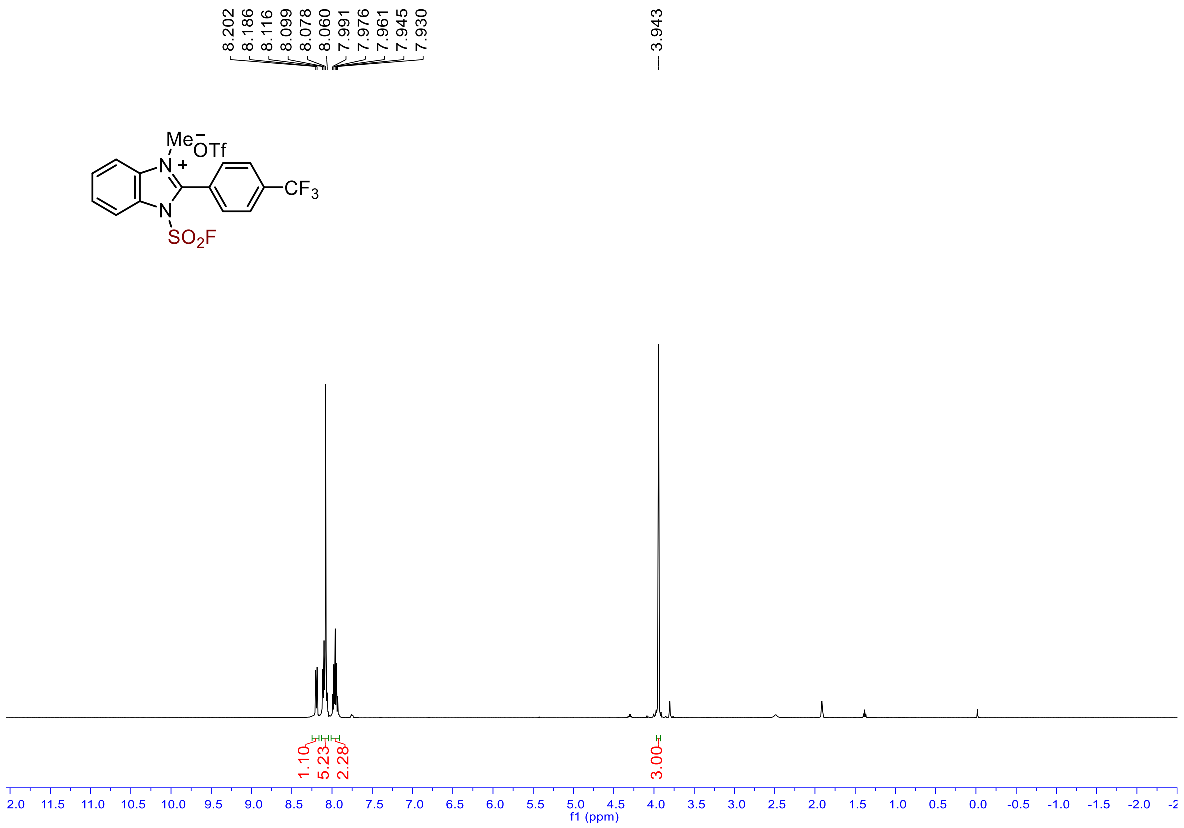

Supplementary Figure 31. ^{1}H NMR spectra of **2e**

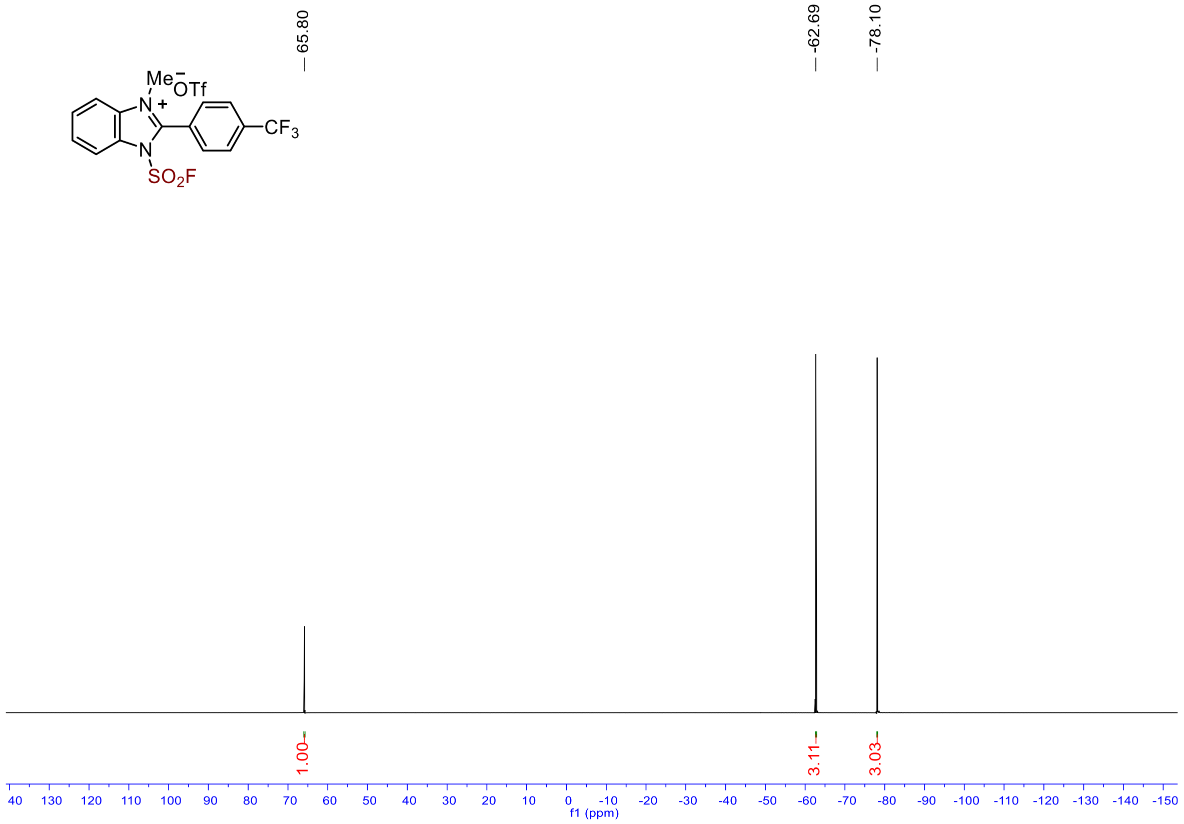

Supplementary Figure 32. ^{19}F NMR spectra of **2e**

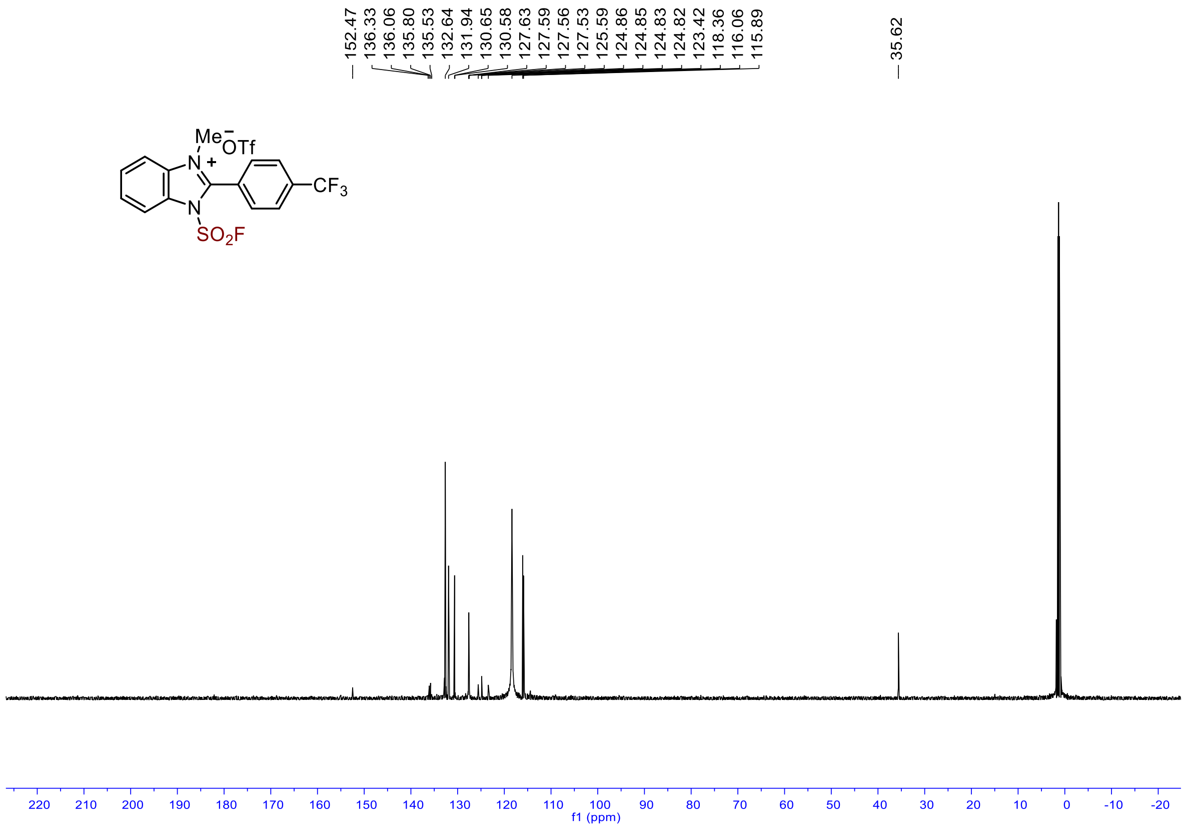

Supplementary Figure 33. ^{13}C NMR spectra of **2e**

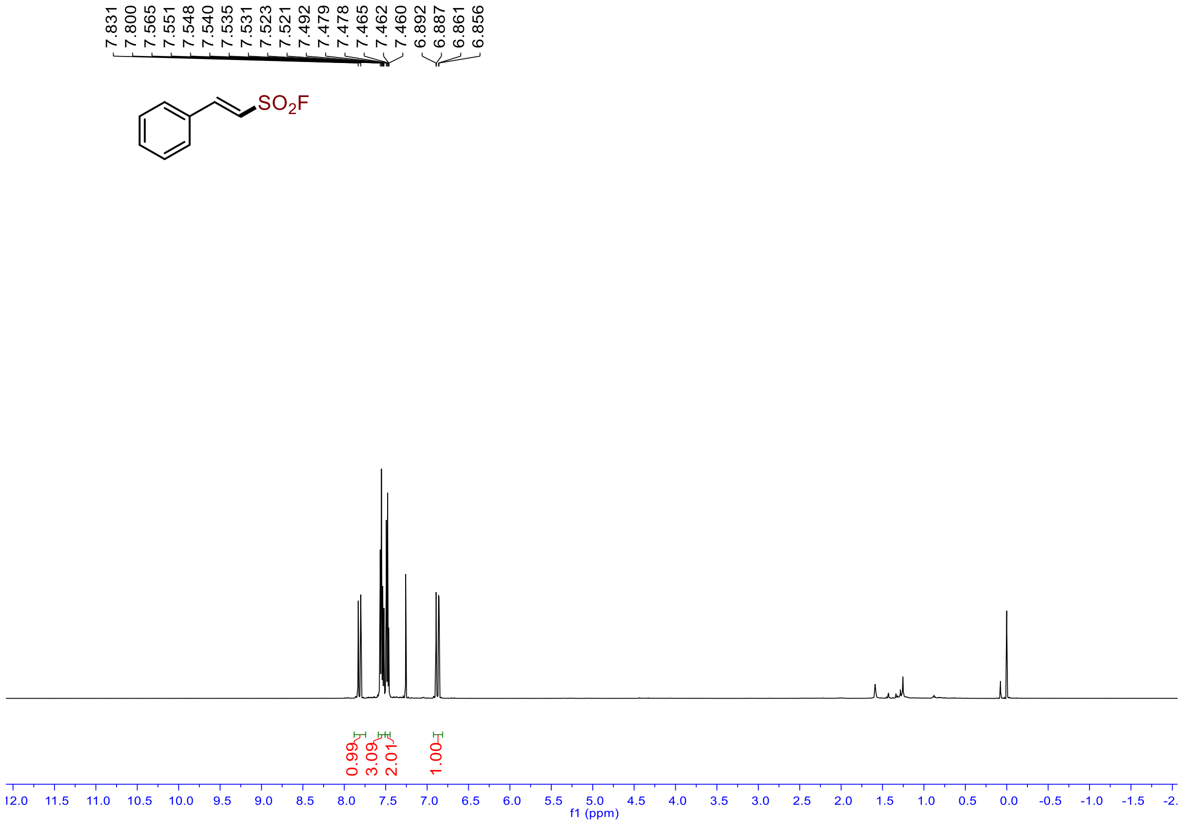

Supplementary Figure 34. ^{1}H NMR spectra of **3aa**

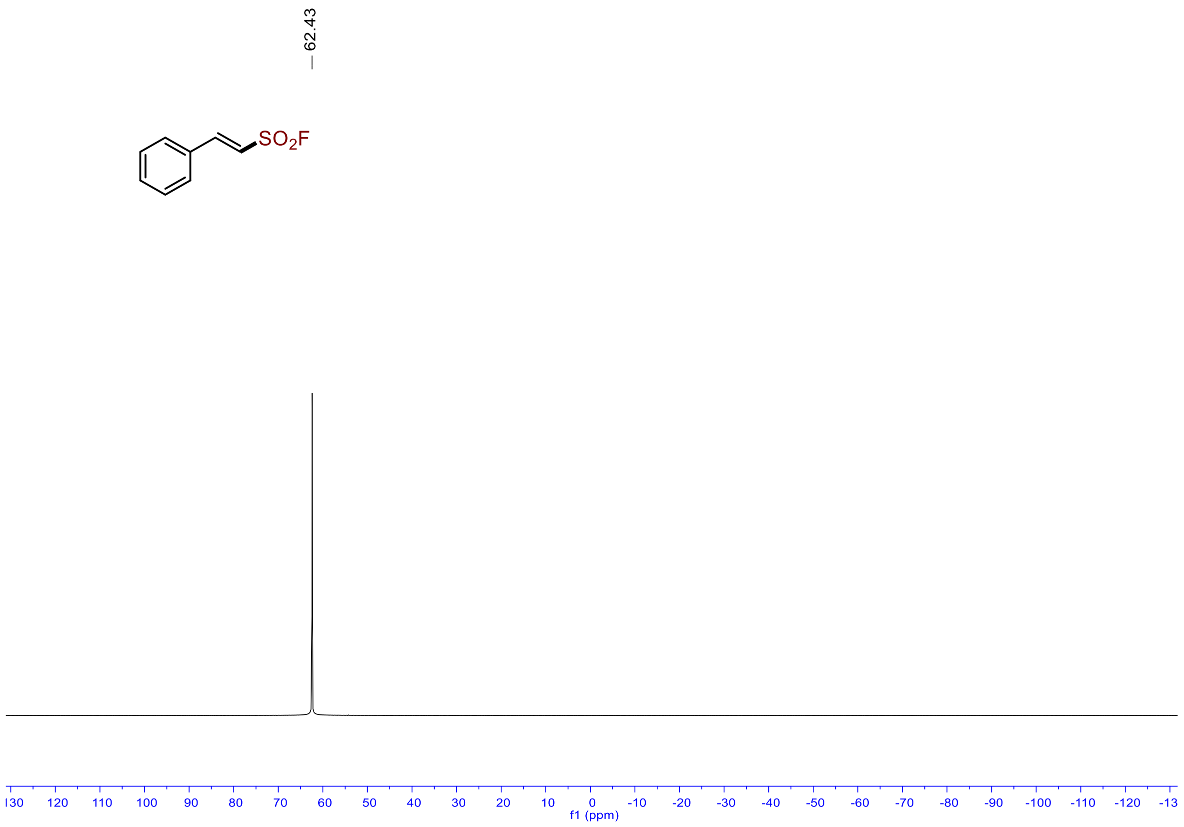

Supplementary Figure 35. ^{19}F NMR spectra of **3aa**

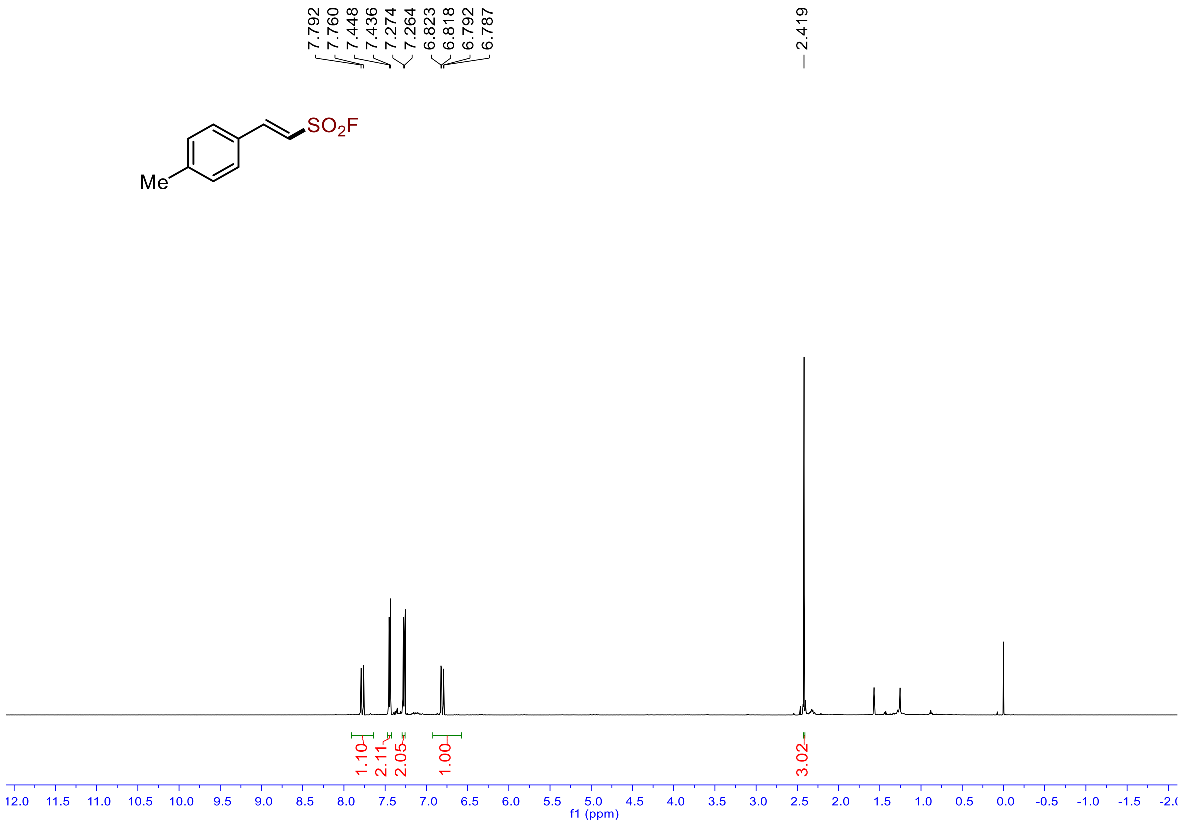

Supplementary Figure 36. ^{1}H NMR spectra of **3ab**

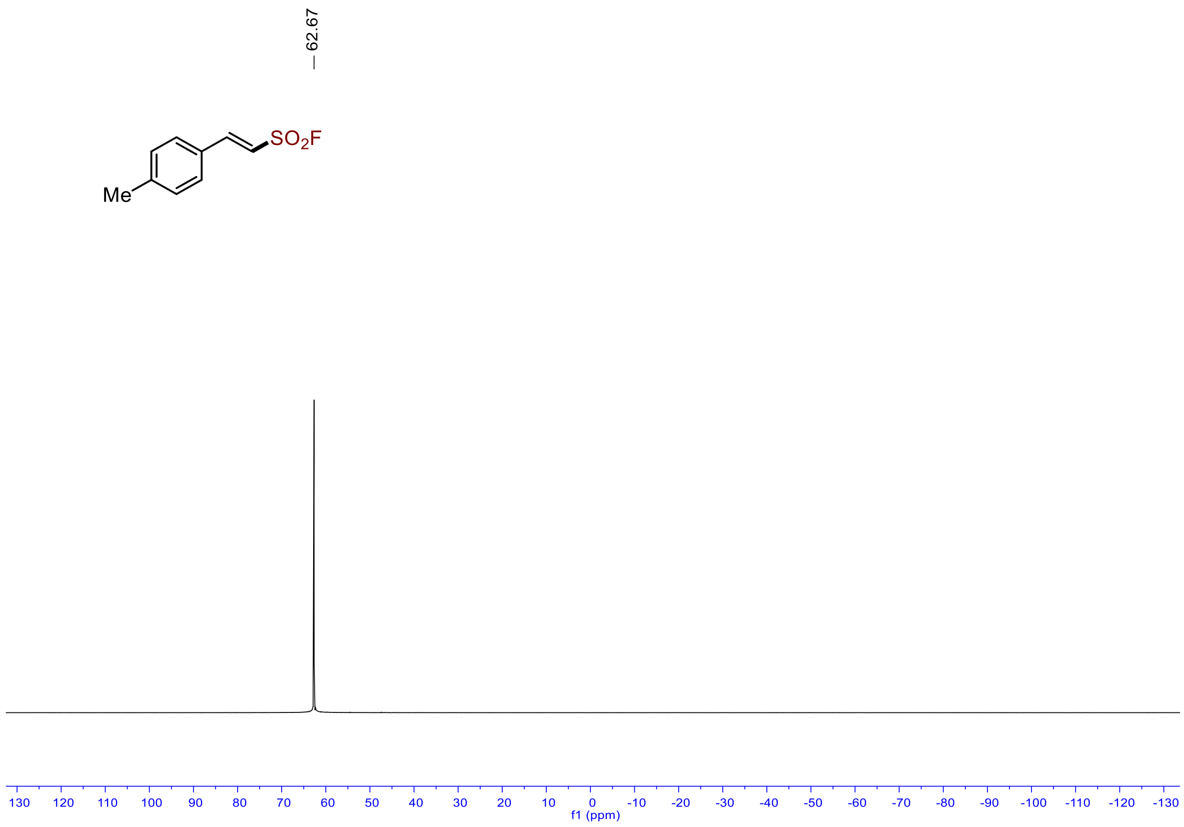

Supplementary Figure 37. ^{13}F NMR spectra of **3ab**

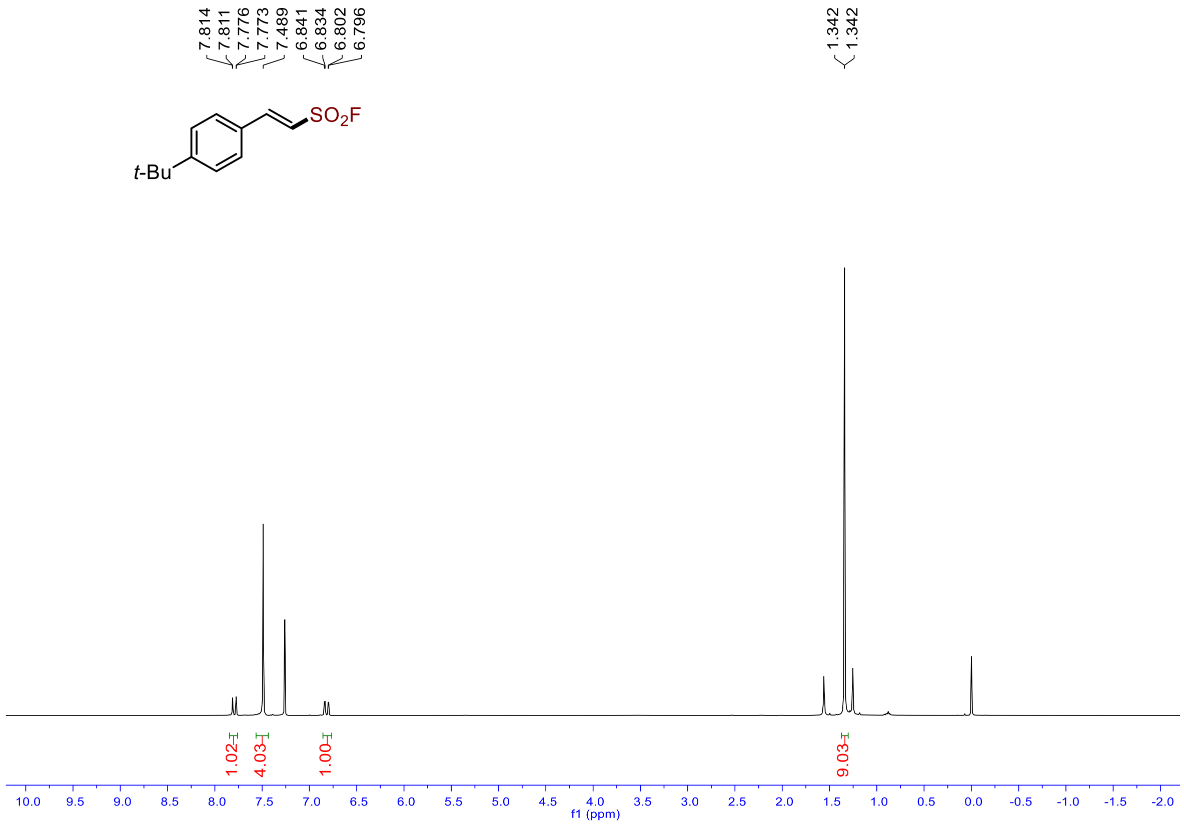

Supplementary Figure 38. ^{1}H NMR spectra of **3ac**

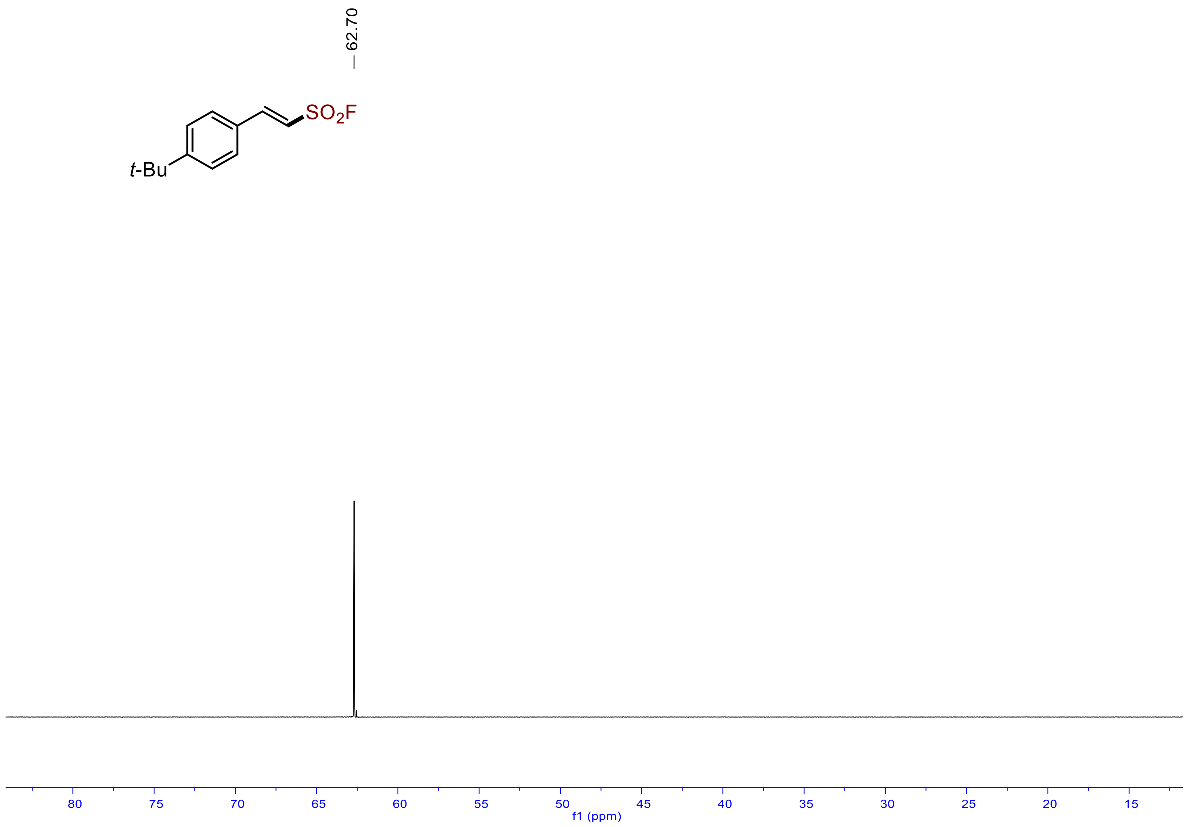

Supplementary Figure 39. ^{19}F NMR spectra of **3ac**

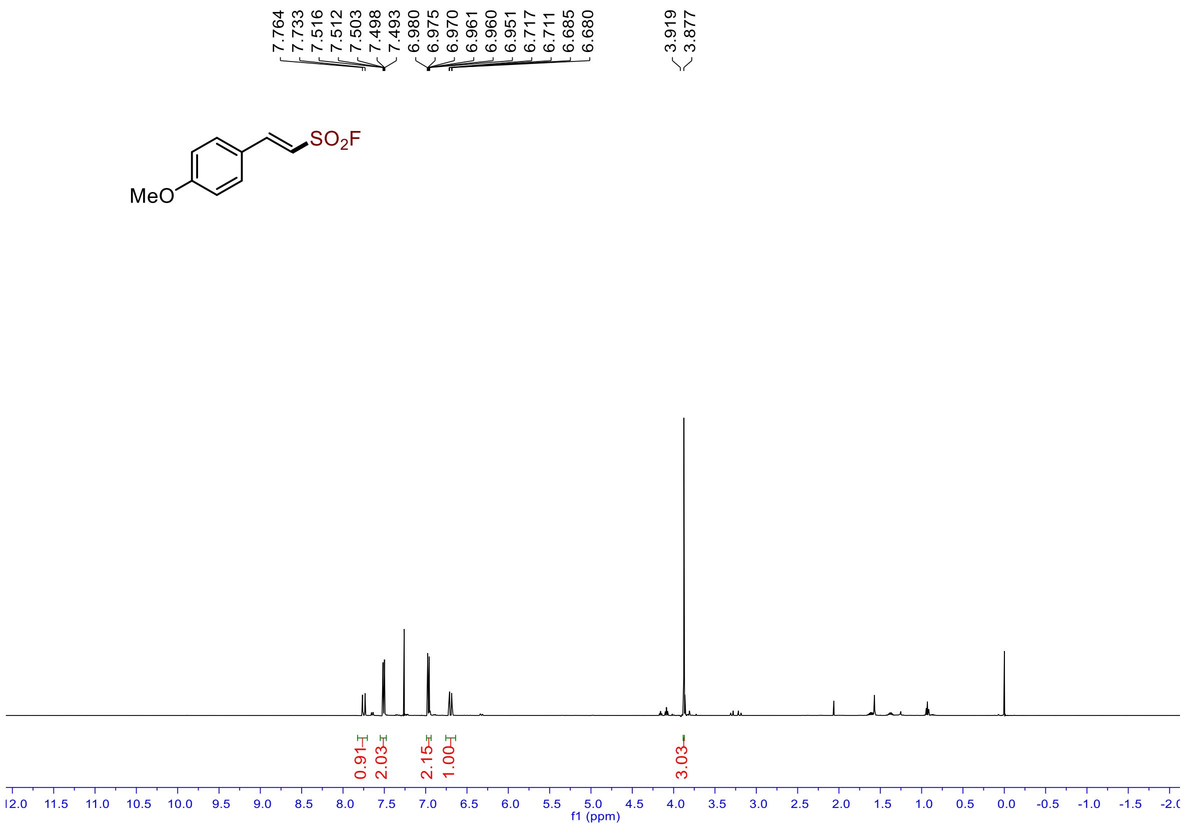

Supplementary Figure 40. ^{1}H NMR spectra of **3ad**

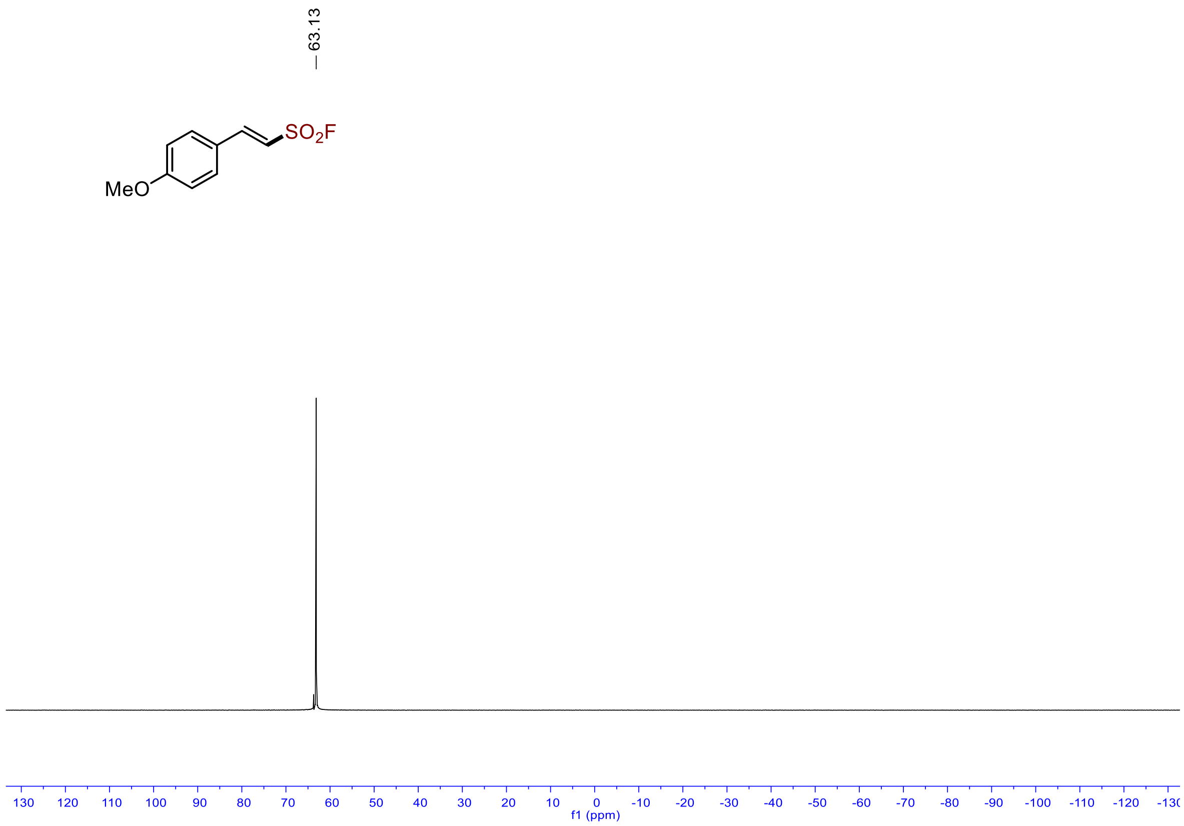

Supplementary Figure 41. ^{19}F NMR spectra of **3ad**

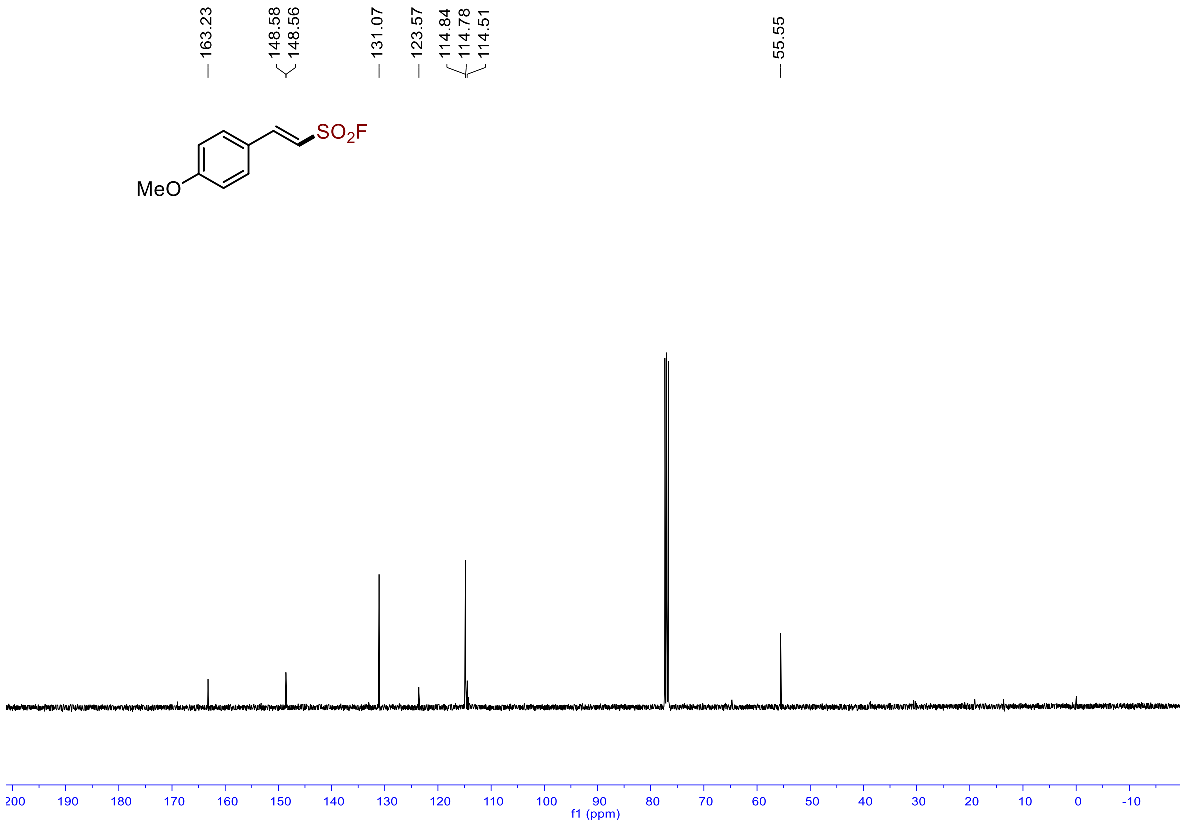

Supplementary Figure 42. ^{13}C NMR spectra of **3ad**

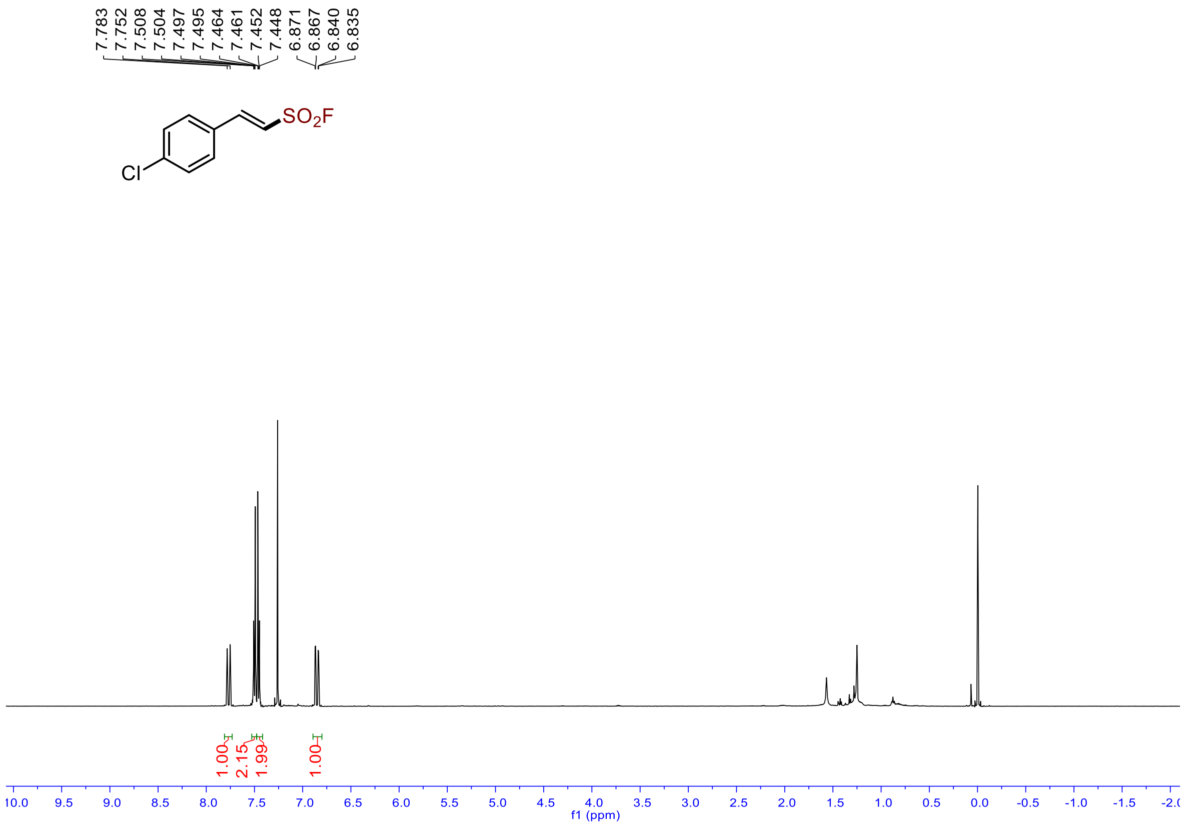

Supplementary Figure 43. ^{1}H NMR spectra of **3ae**

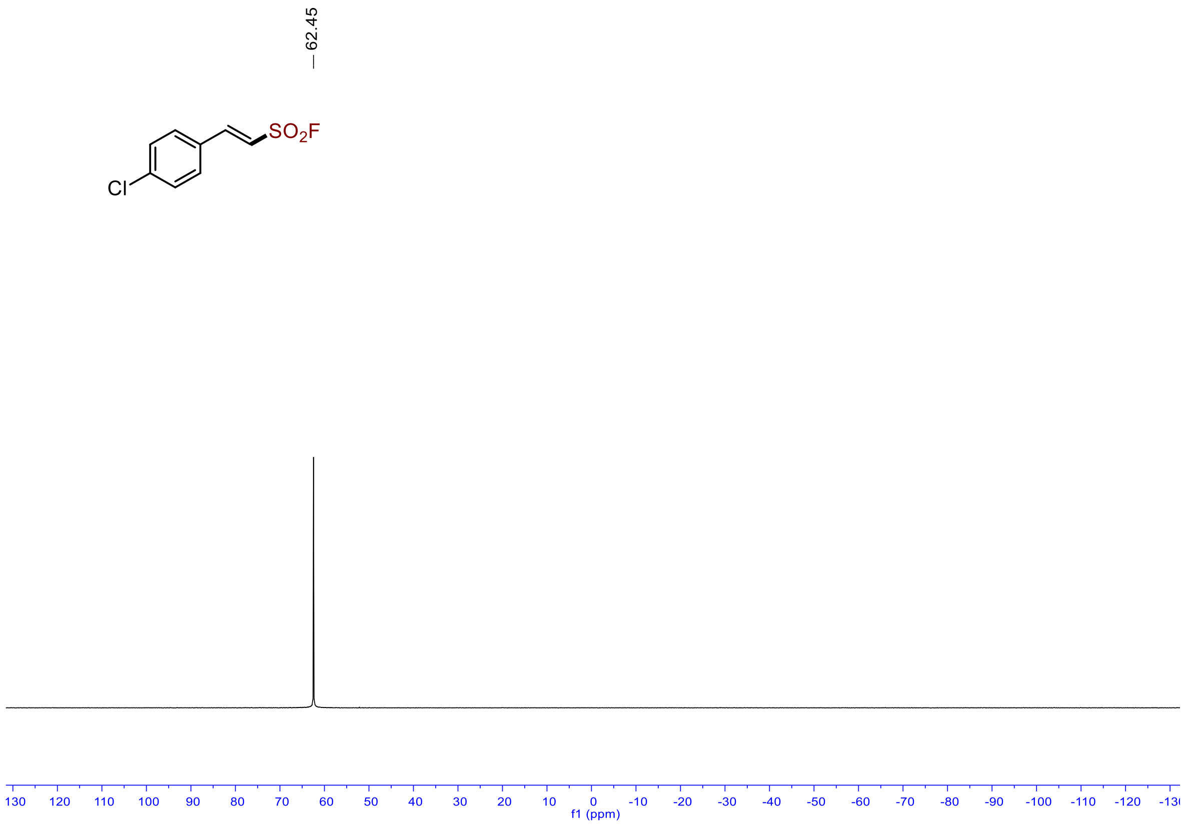

Supplementary Figure 44. ^{19}F NMR spectra of **3ae**

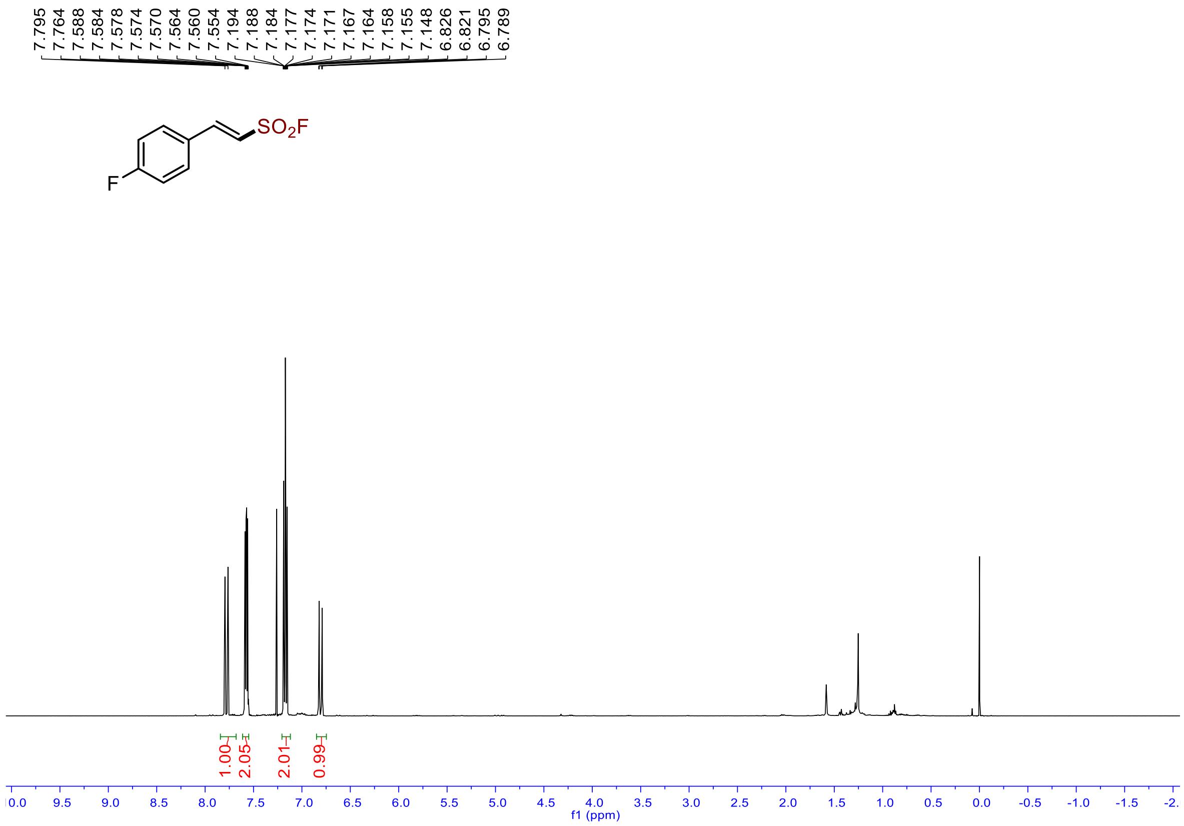

Supplementary Figure 45. ^{1}H NMR spectra of **3af**

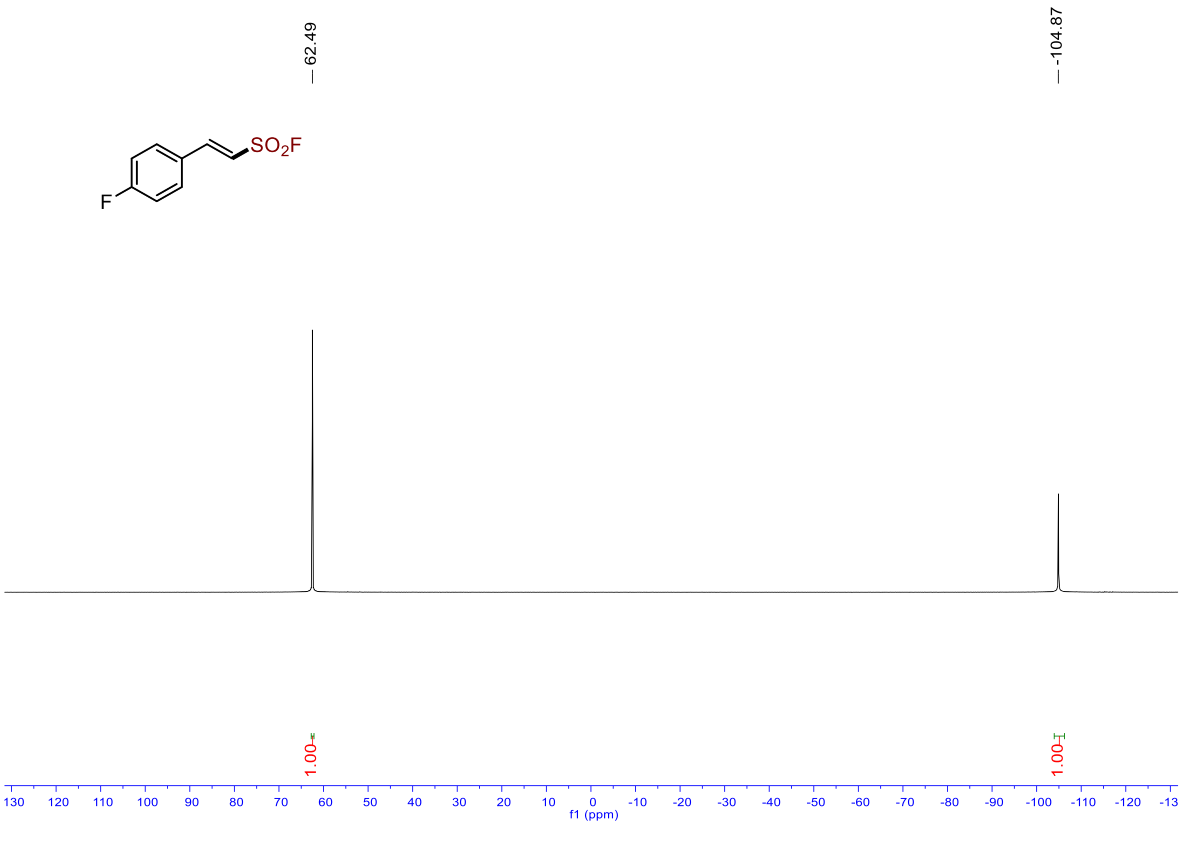

Supplementary Figure 46. ^{19}F NMR spectra of **3af**

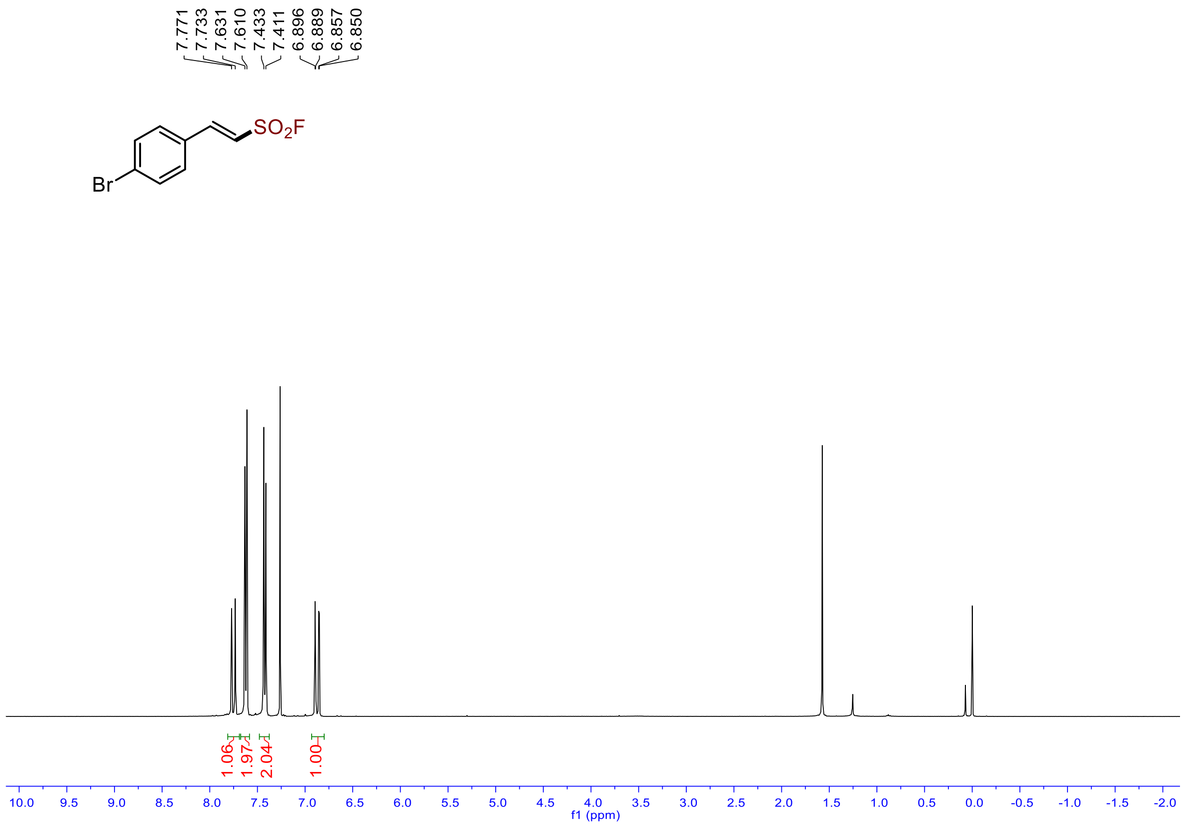

Supplementary Figure 47. ^{1}H NMR spectra of **3ag**

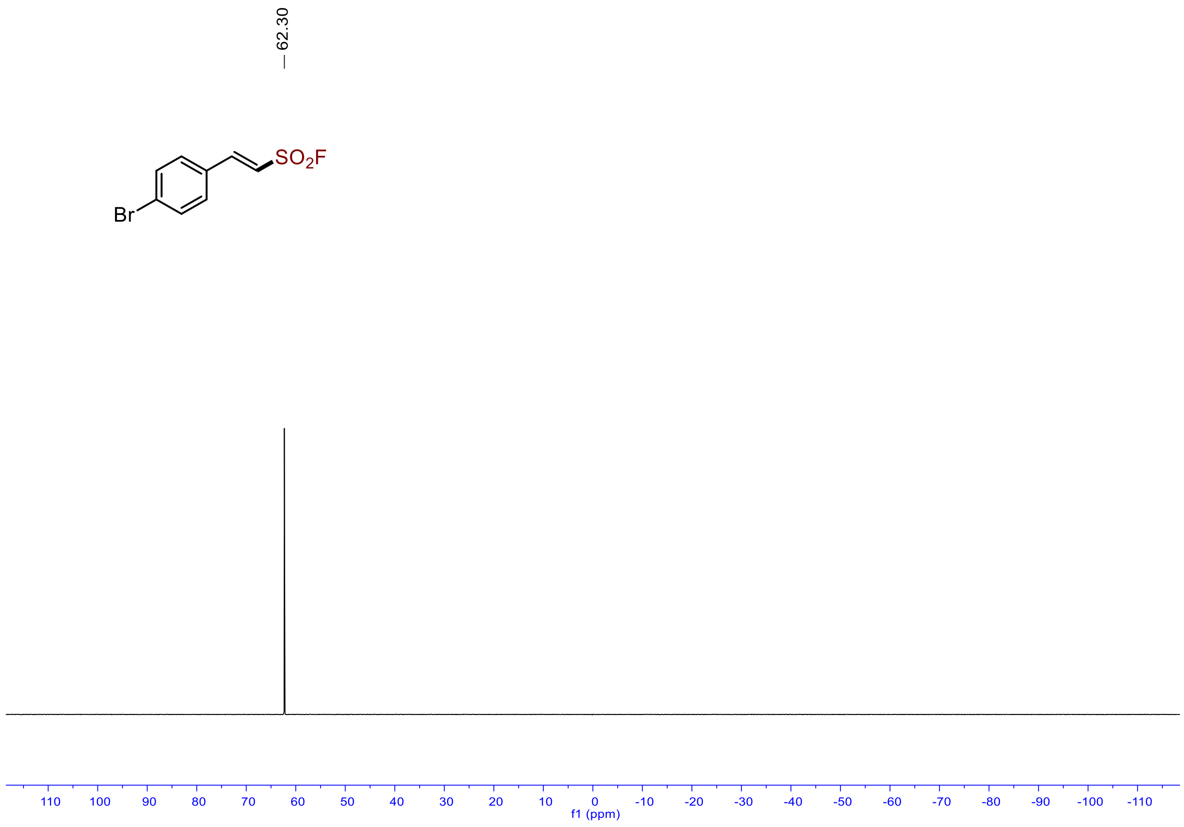

Supplementary Figure 48. ^{19}F NMR spectra of **3ag**

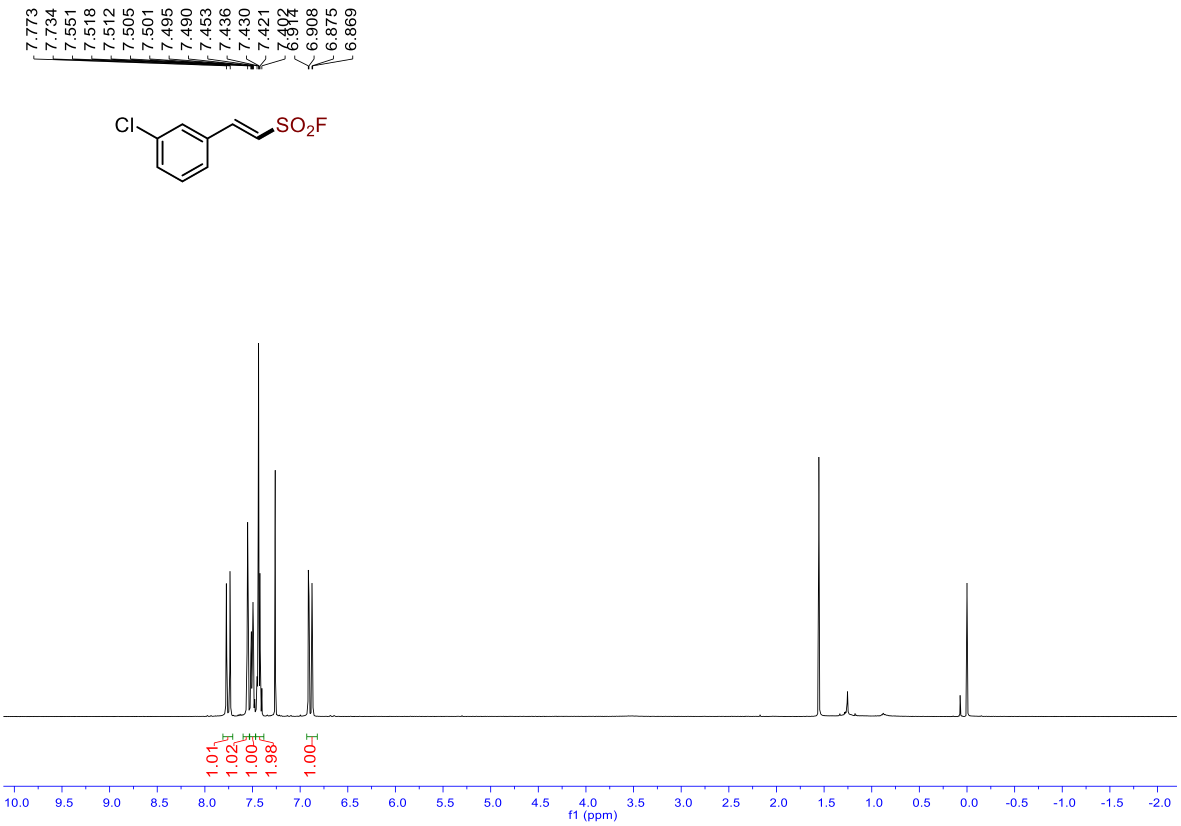

Supplementary Figure 49. ^{1}H NMR spectra of **3ah**

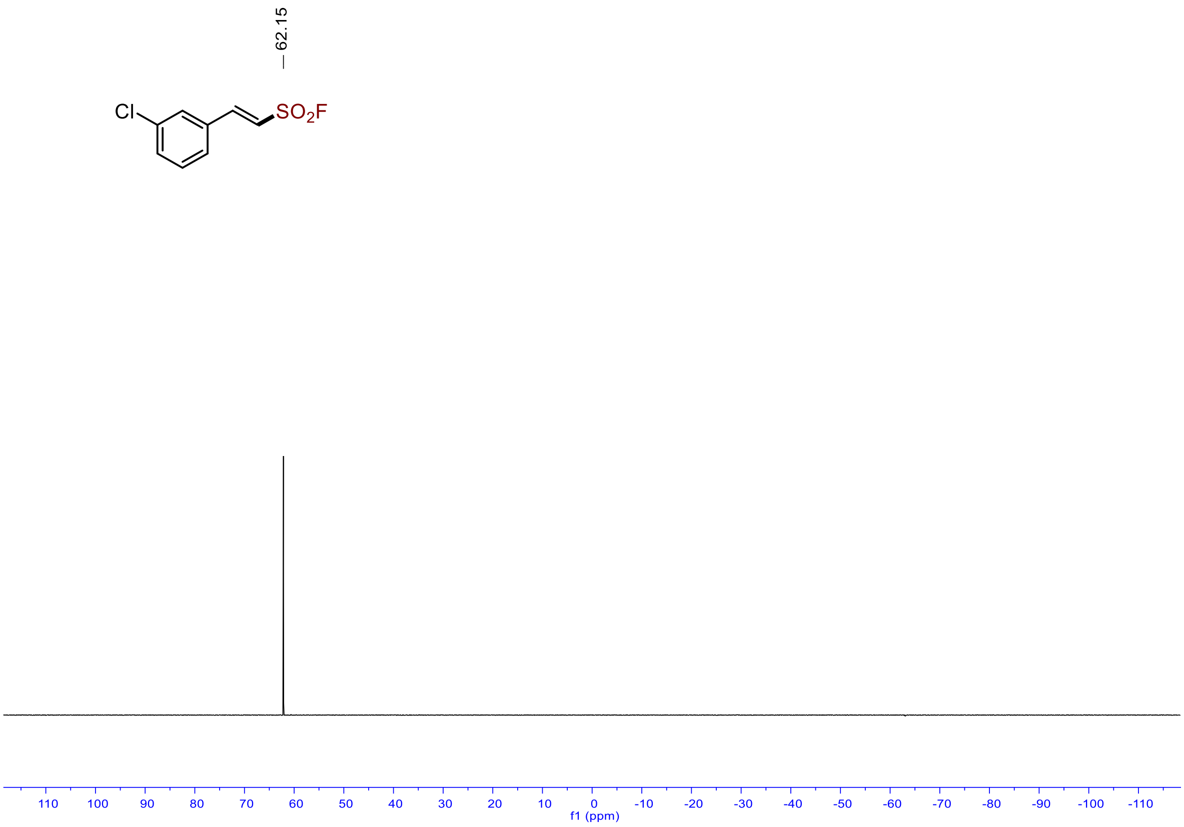

Supplementary Figure 50. ^{19}F NMR spectra of **3ah**

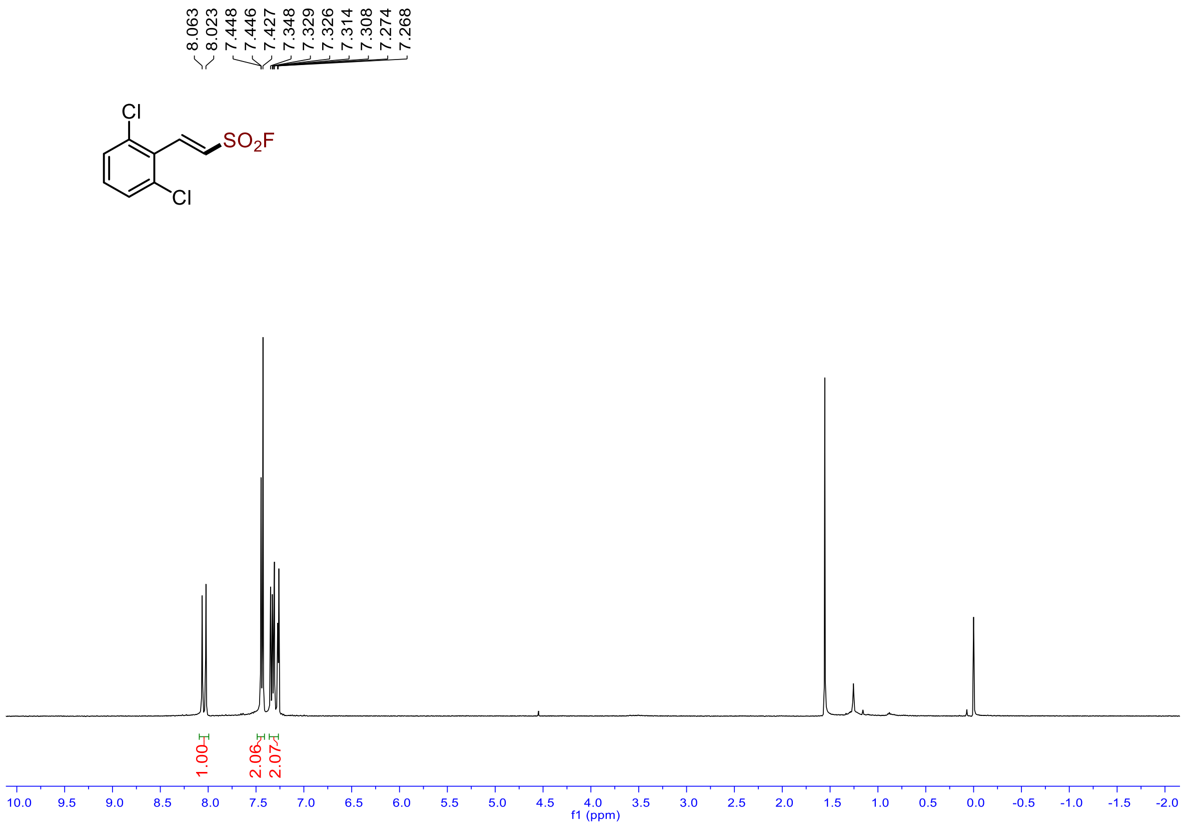

Supplementary Figure 51. ^{1}H NMR spectra of **3ai**

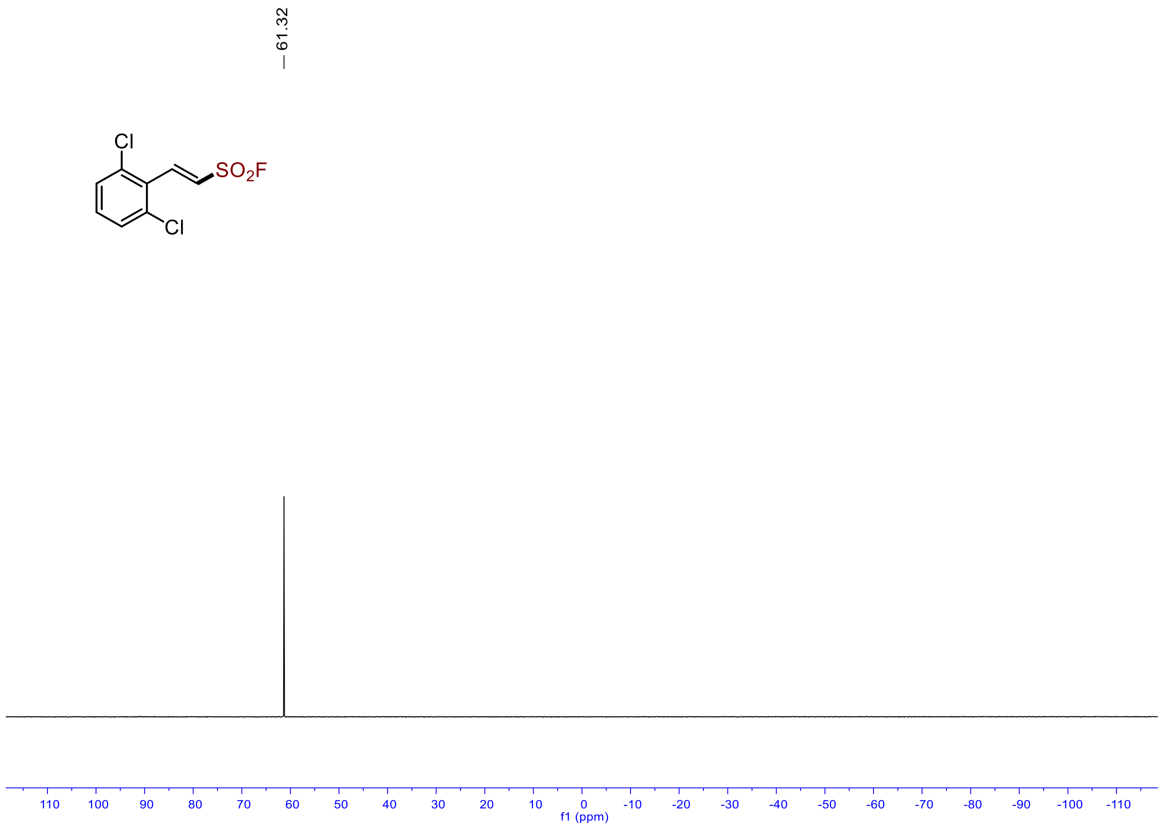

Supplementary Figure 52. ^{19}F NMR spectra of **3ai**

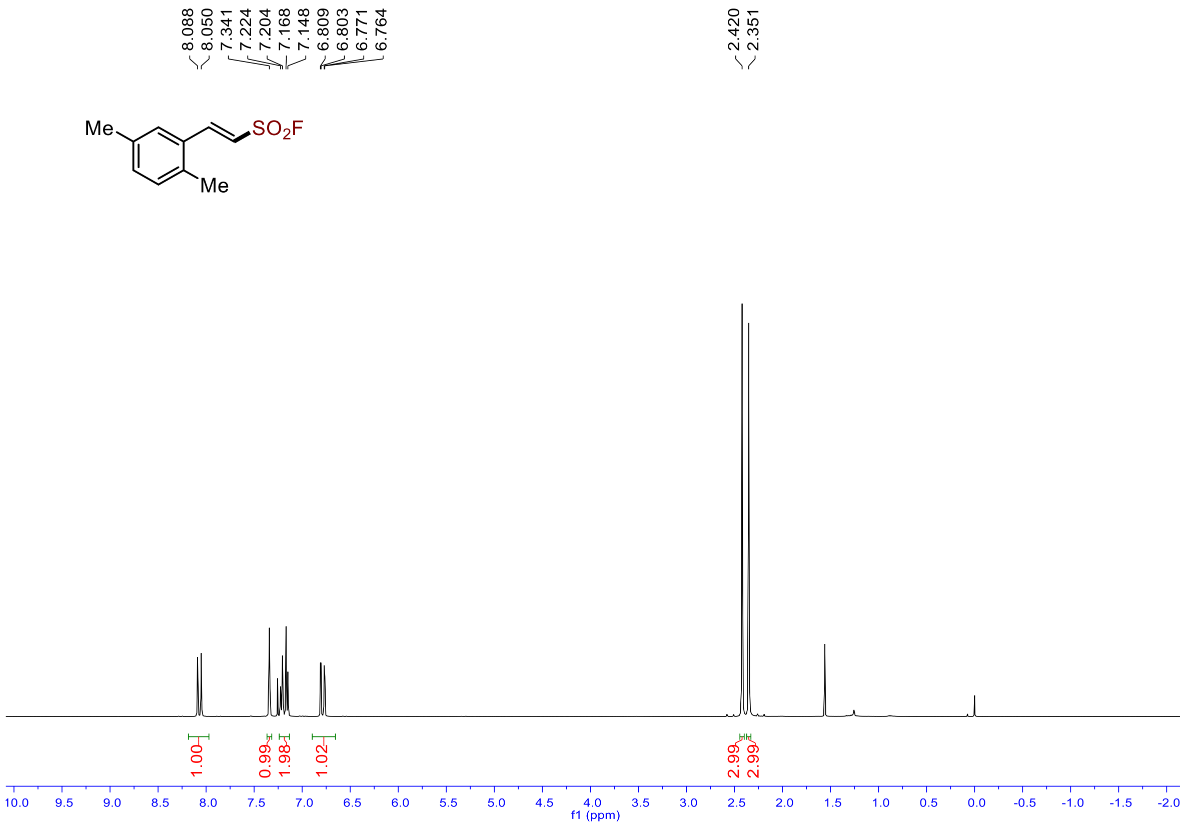

Supplementary Figure 53. ^{1}H NMR spectra of **3aj**

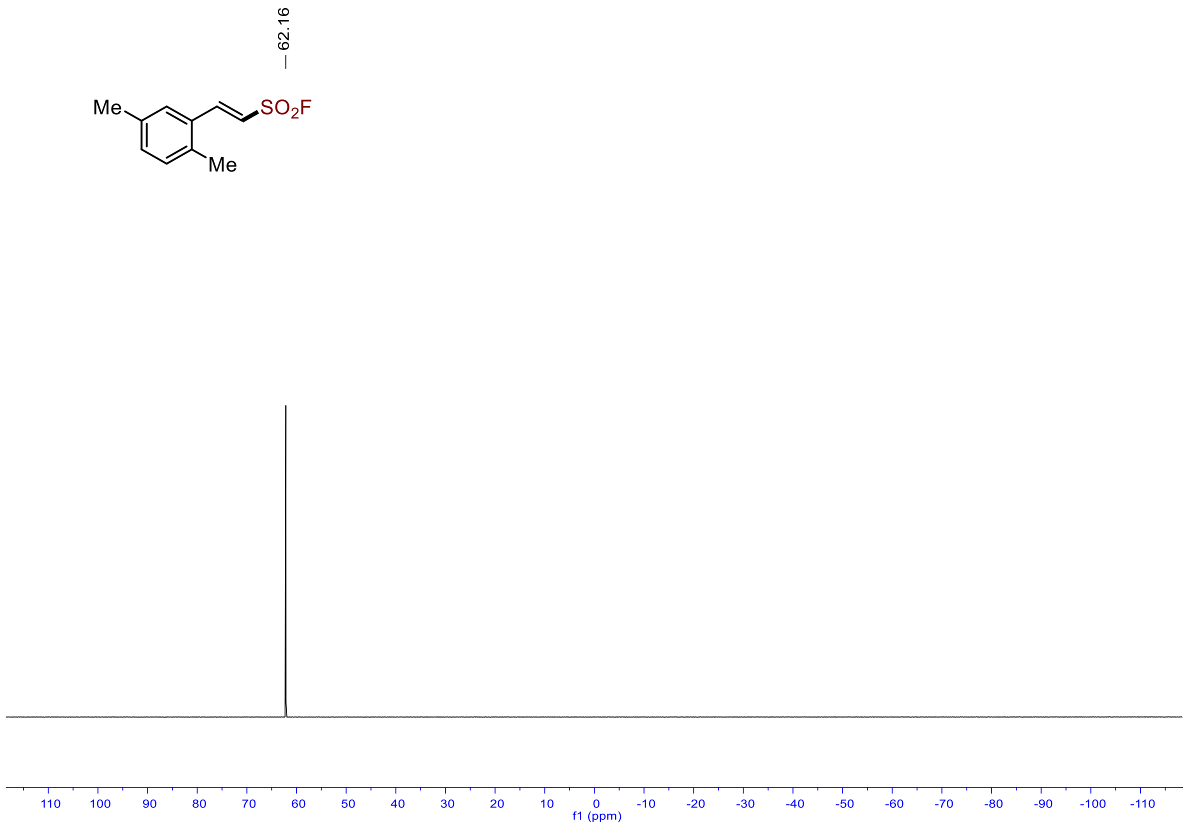

Supplementary Figure 54. ^{19}F NMR spectra of **3aj**

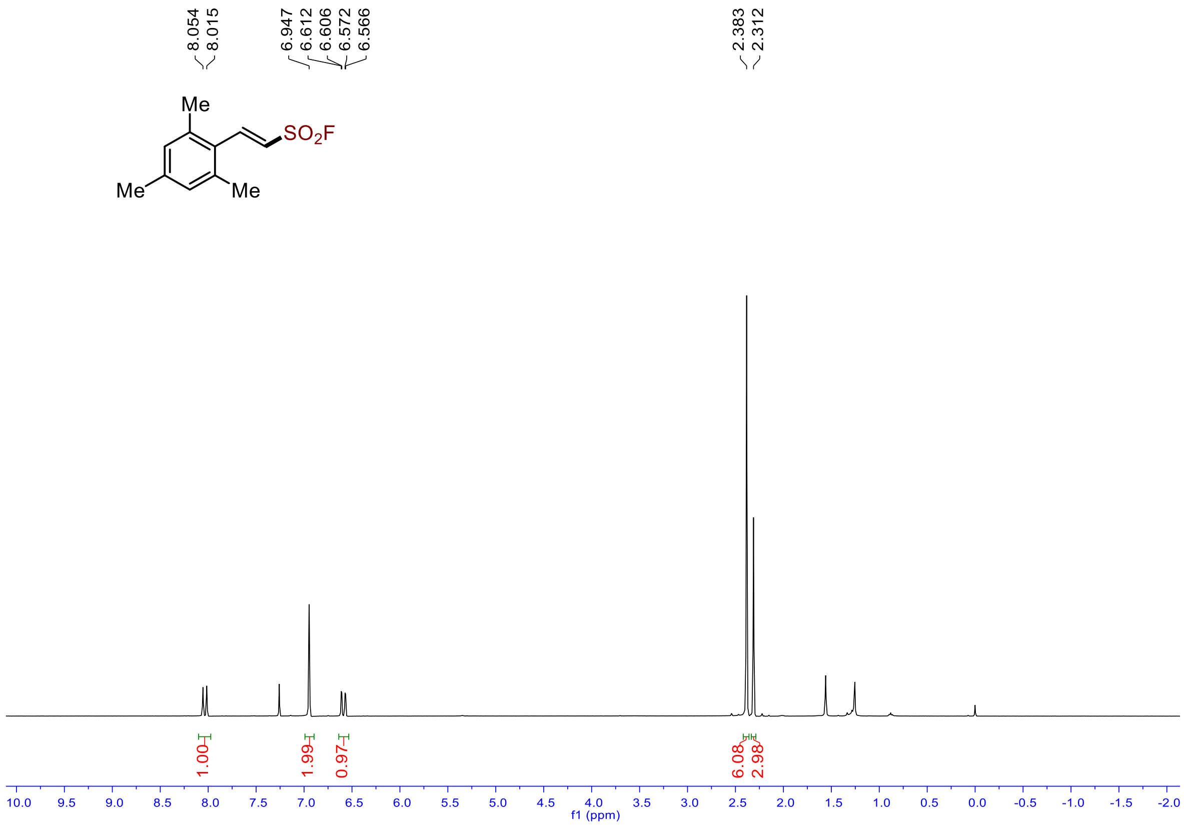

Supplementary Figure 55. ^{1}H NMR spectra of **3ak**

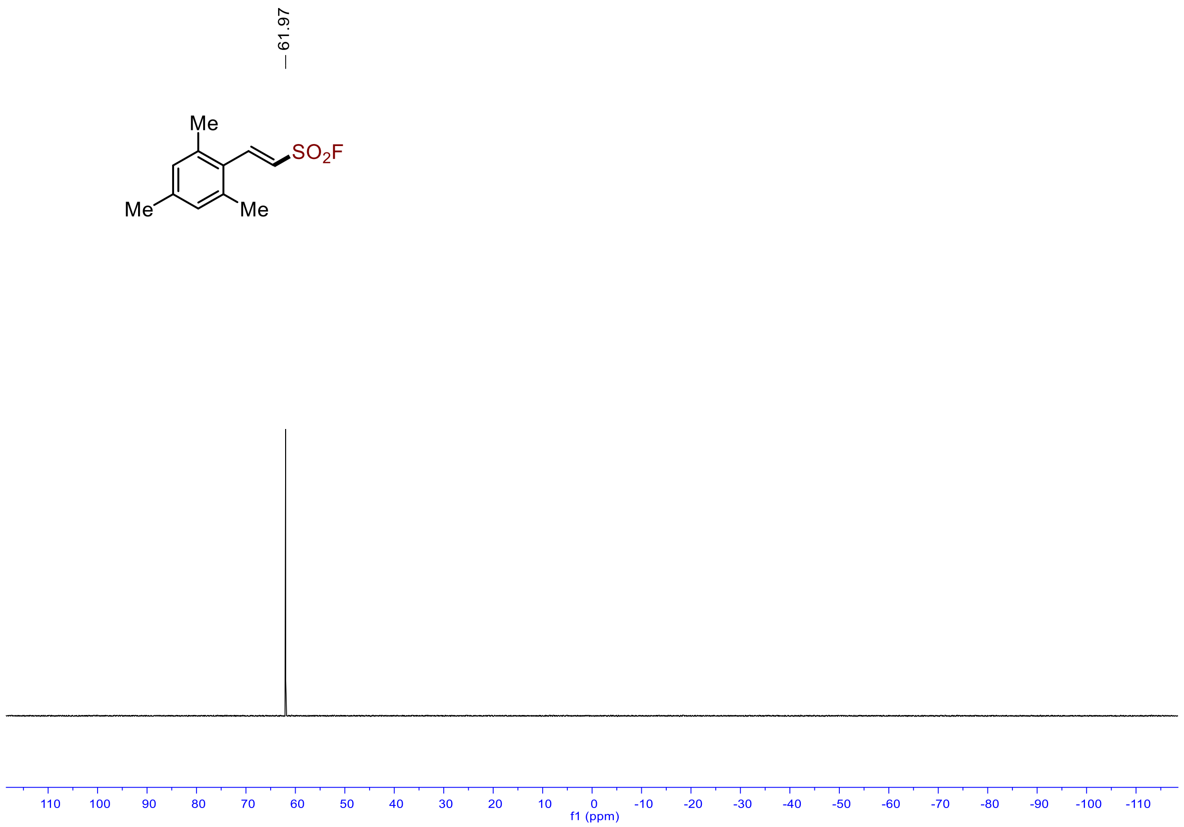

Supplementary Figure 56. ^{19}F NMR spectra of **3ak**

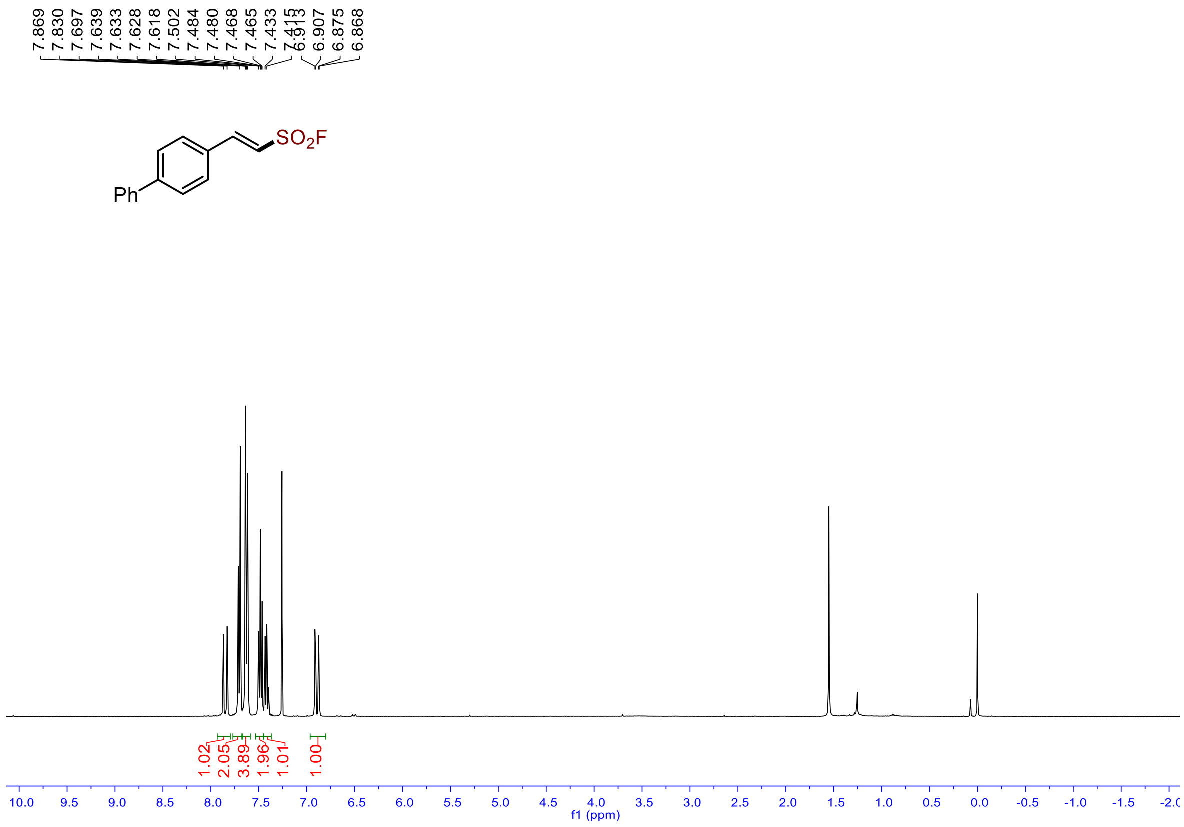

Supplementary Figure 57. ^{1}H NMR spectra of **3al**

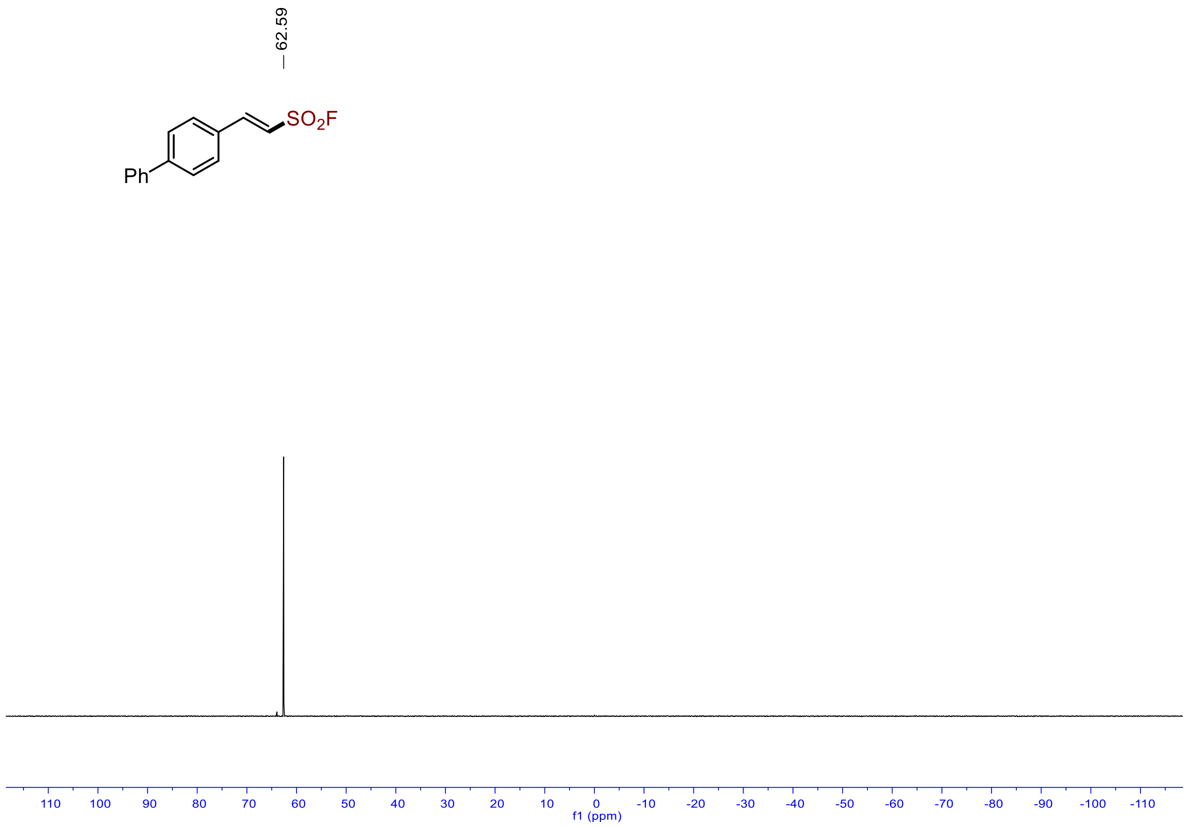

Supplementary Figure 58. ^{19}F NMR spectra of **3al**

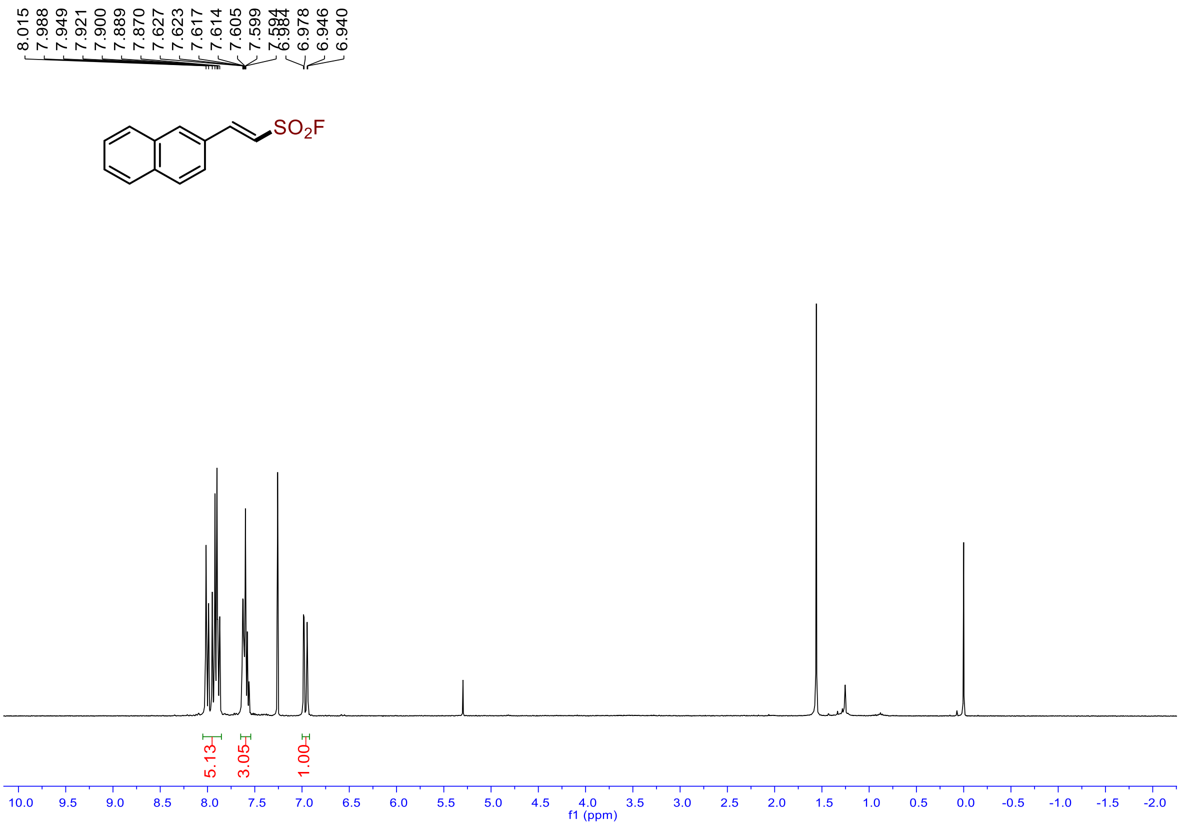

Supplementary Figure 59. ^{1}H NMR spectra of **3am**

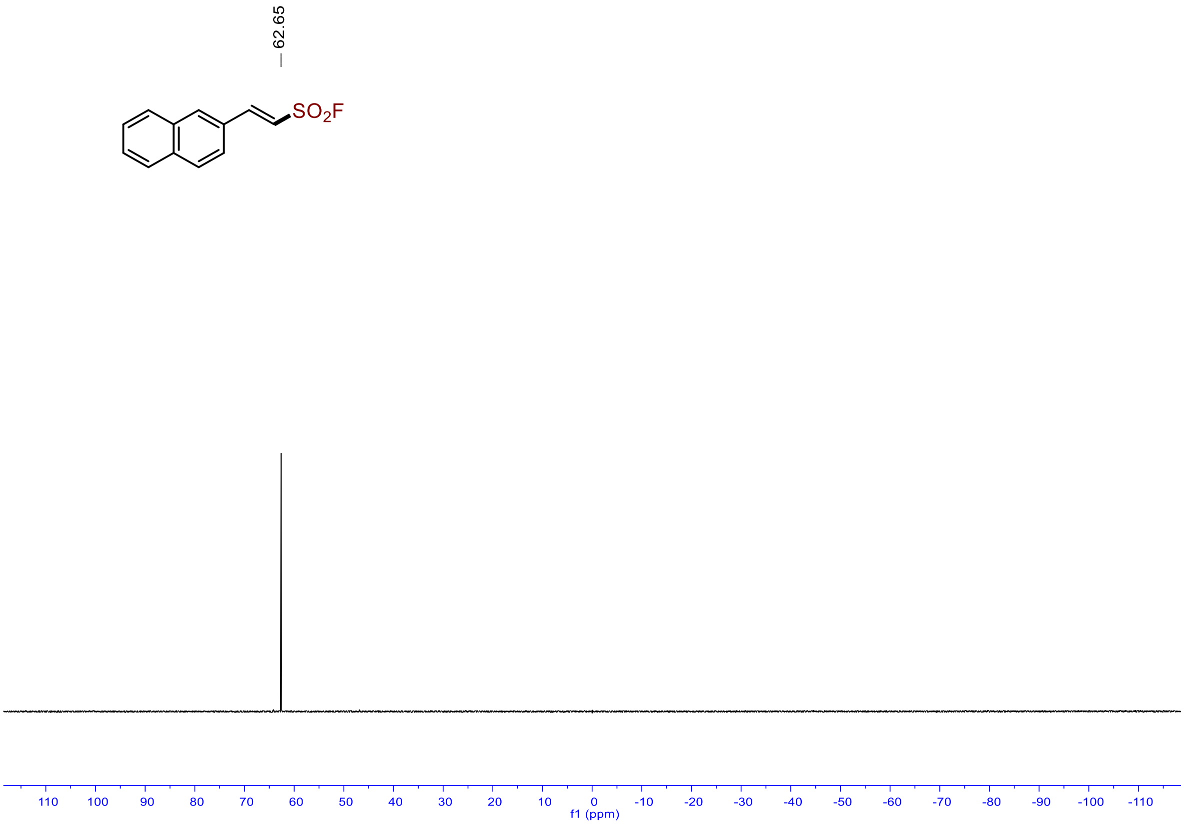

Supplementary Figure 60. ^{19}F NMR spectra of **3am**

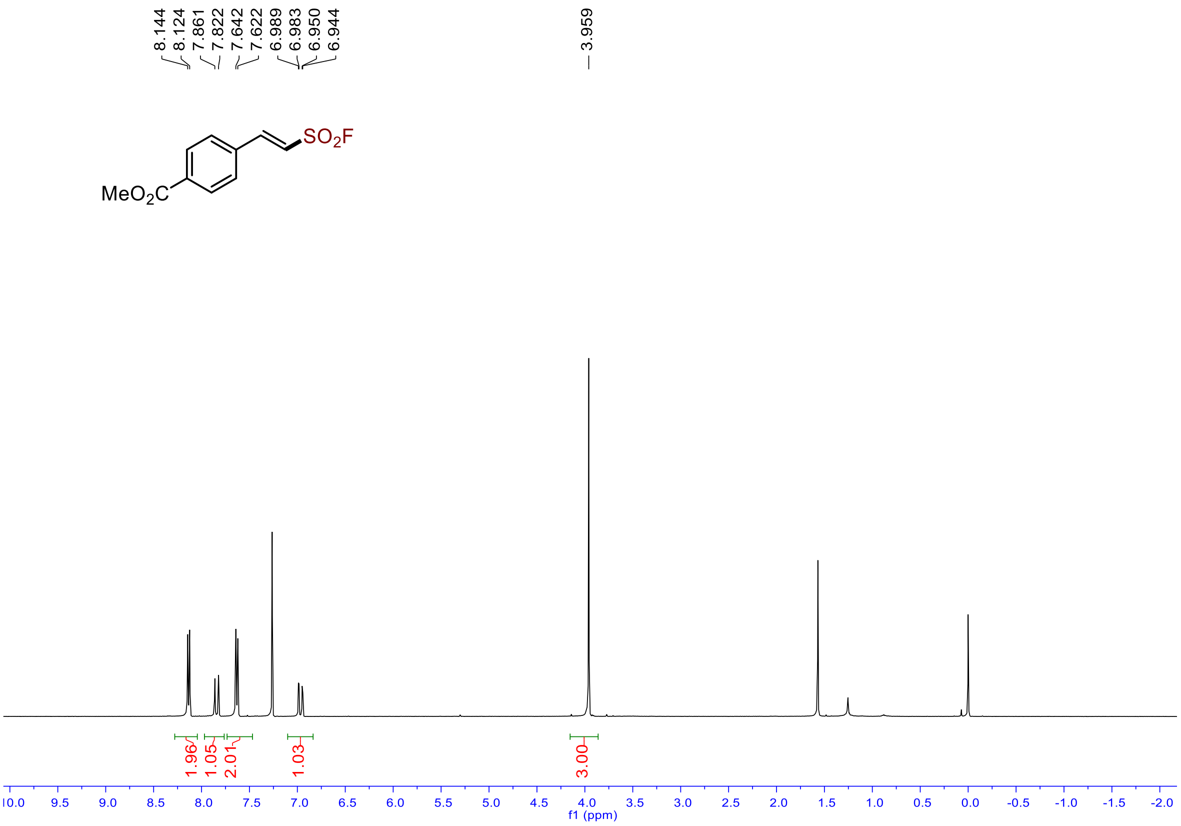

Supplementary Figure 61. ^{1}H NMR spectra of **3an**

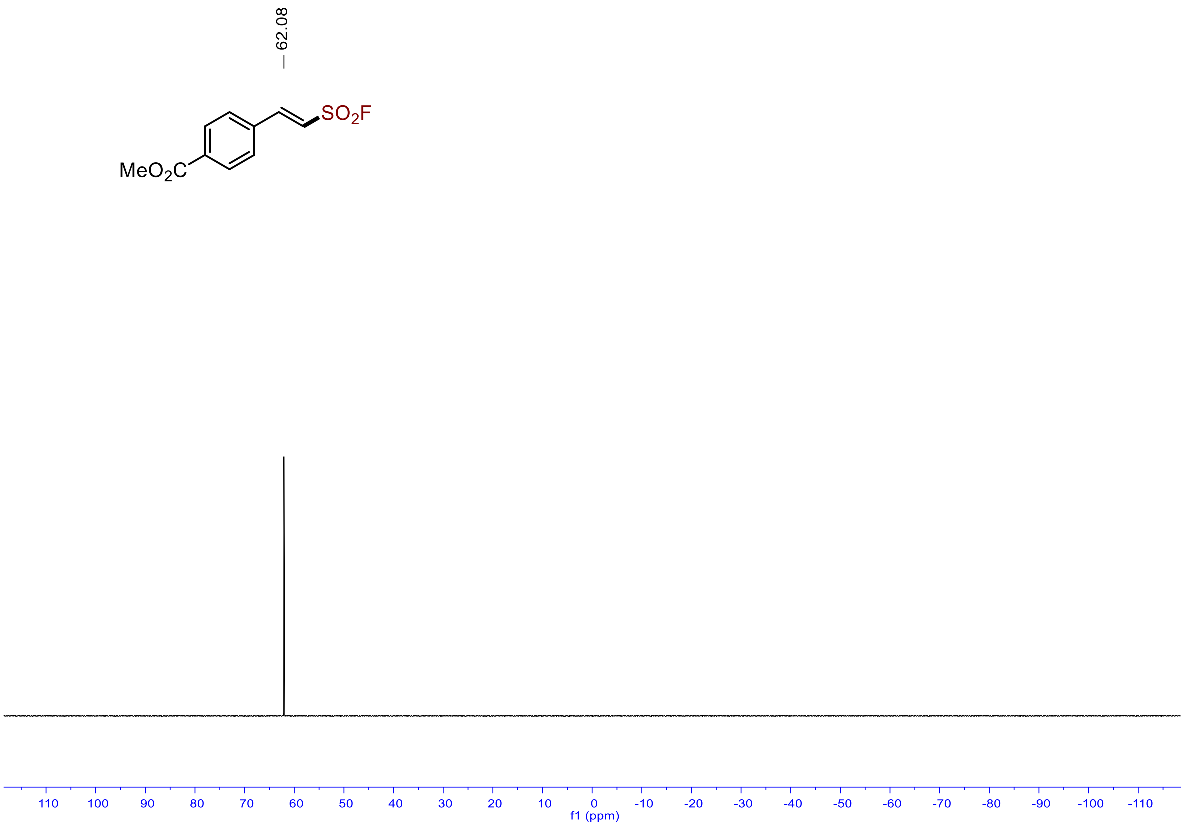

Supplementary Figure 62. ^{19}F NMR spectra of **3an**

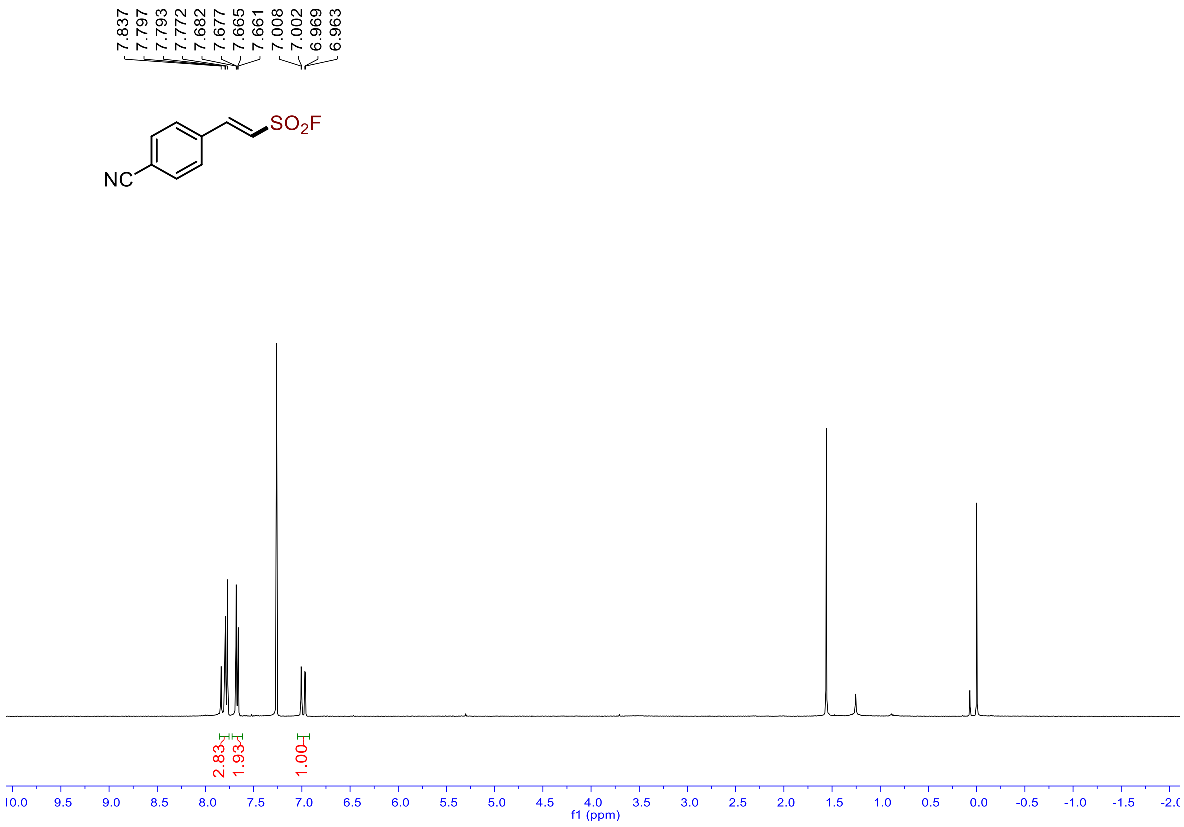

Supplementary Figure 63. ^{1}H NMR spectra of **3ao**

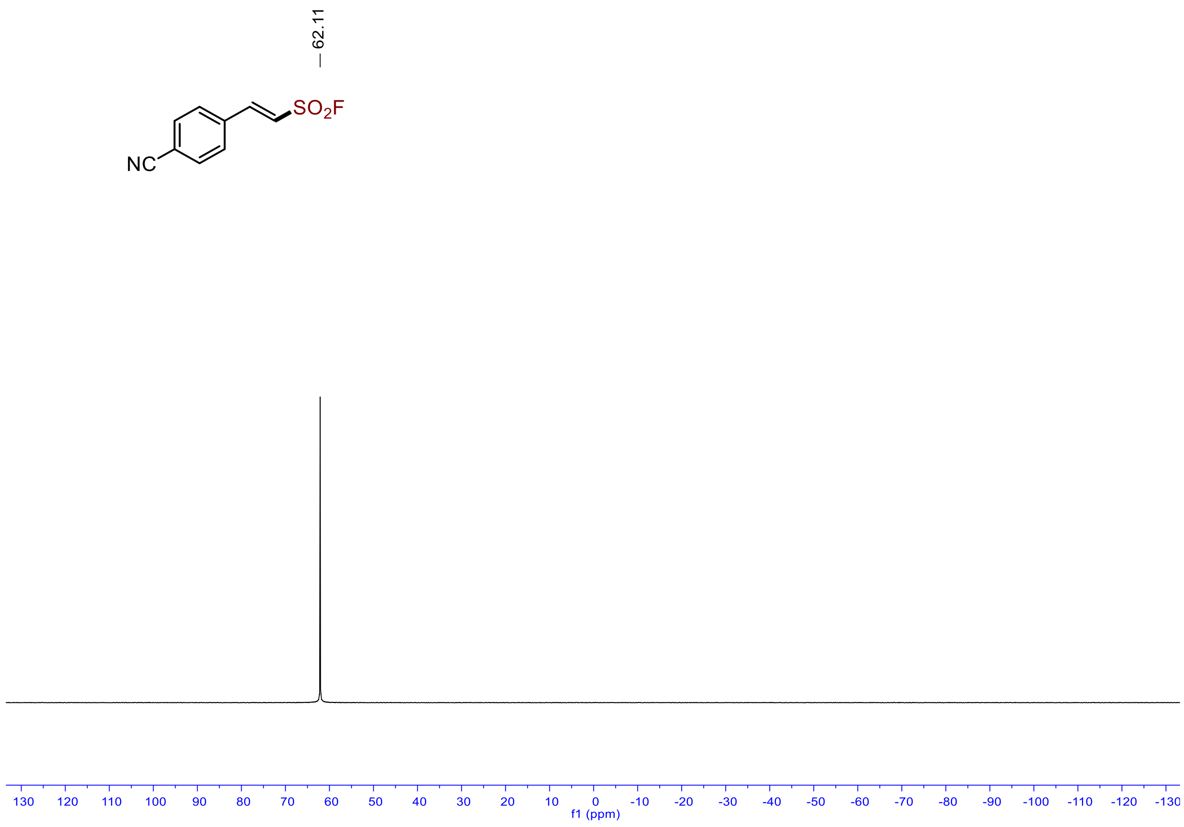

Supplementary Figure 64. ^{19}F NMR spectra of **3ao**

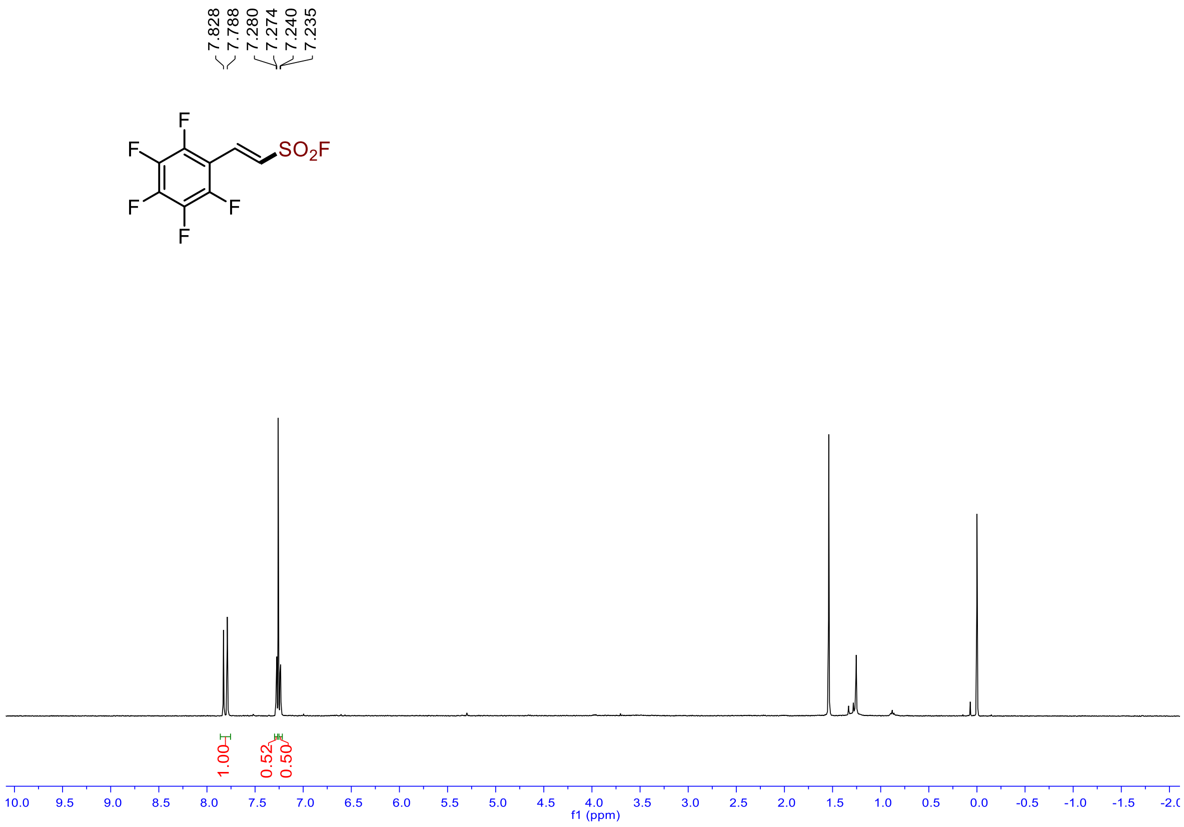

Supplementary Figure 65. ^{1}H NMR spectra of **3ap**

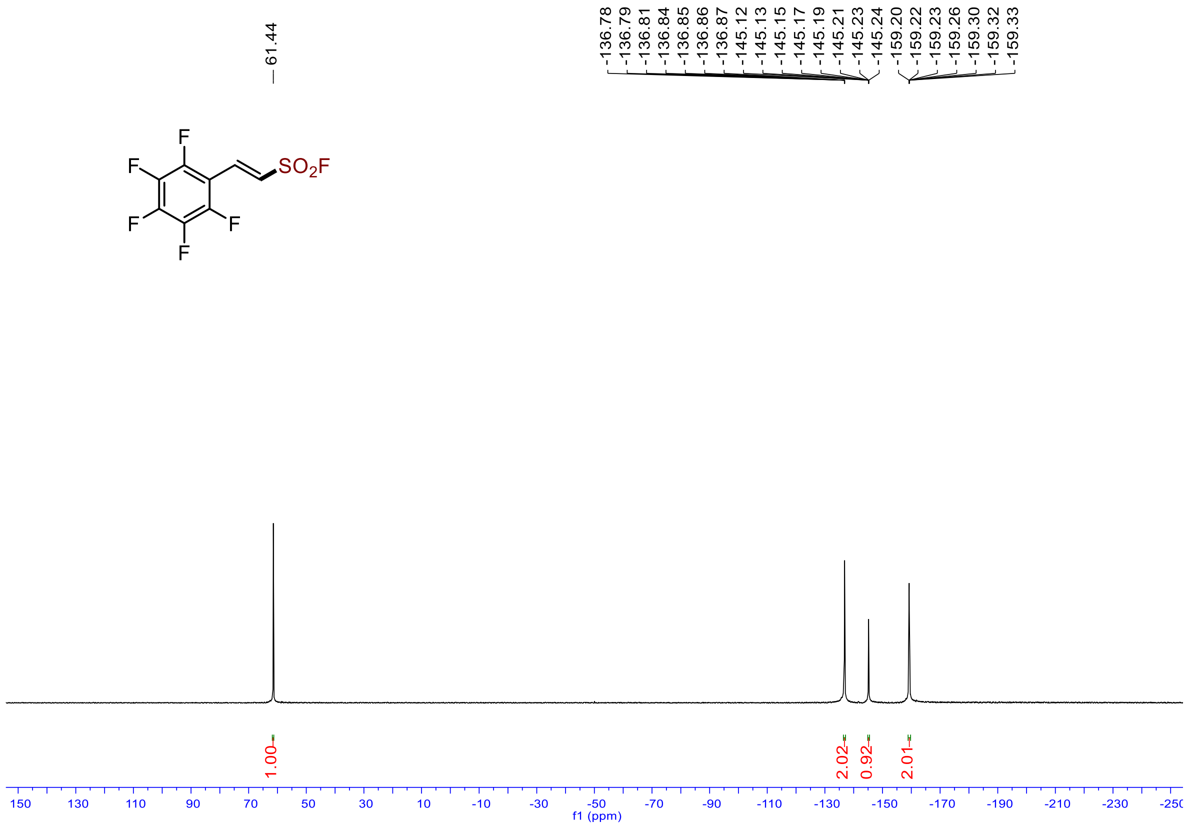

Supplementary Figure 66. ^{19}F NMR spectra of **3ap**

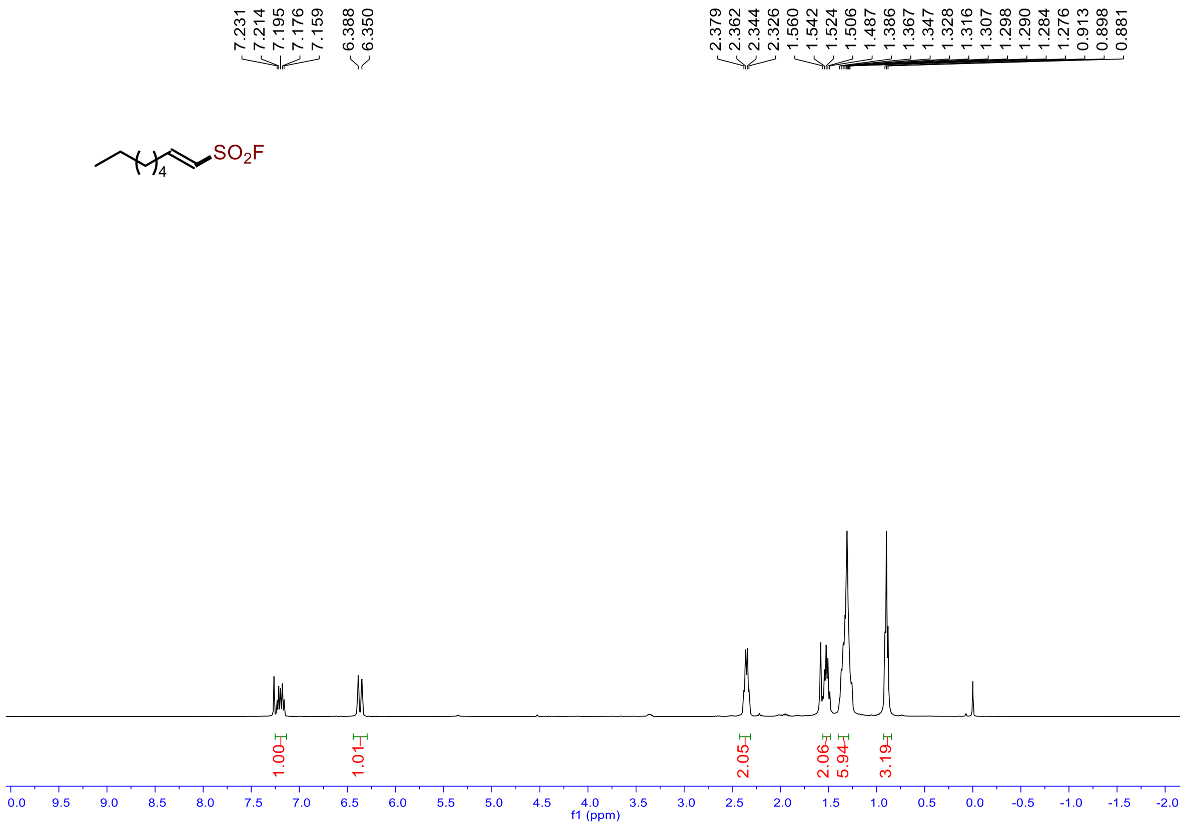

Supplementary Figure 67. ^{1}H NMR spectra of **3aq**

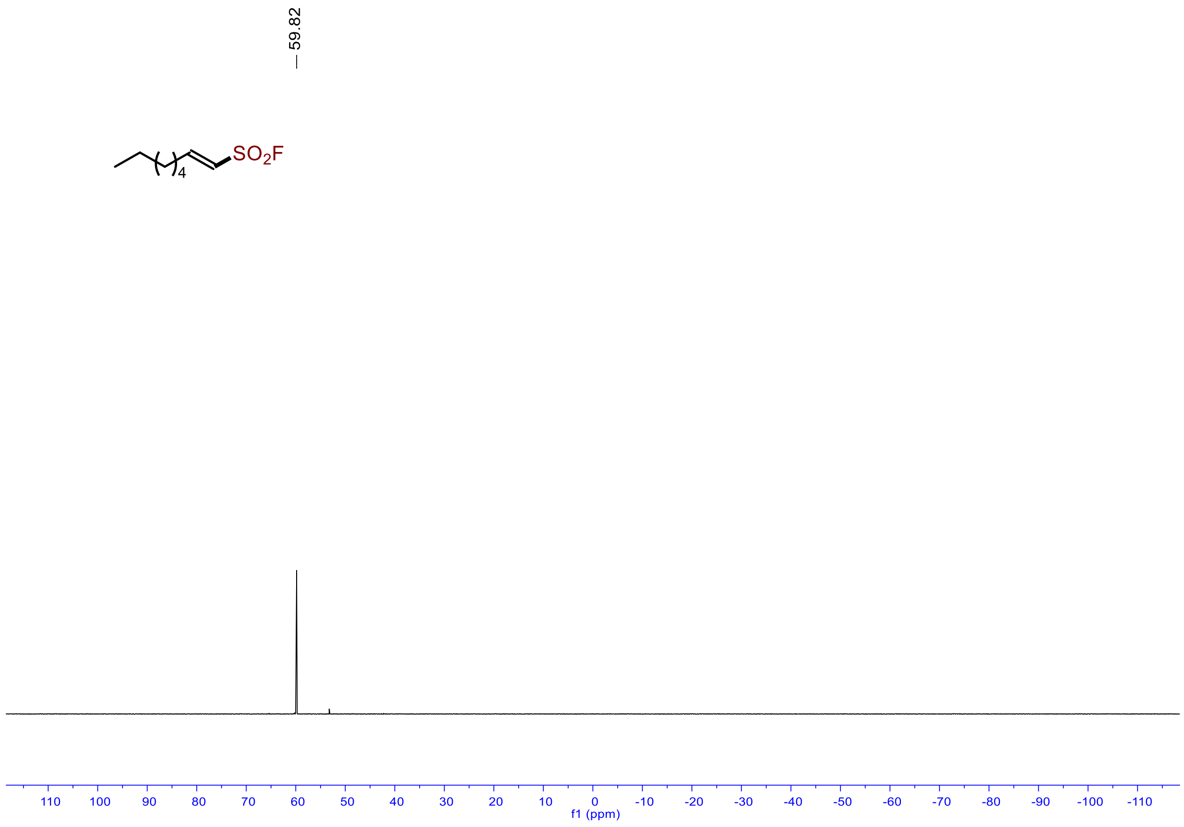

Supplementary Figure 68. ^{19}F NMR spectra of **3aq**

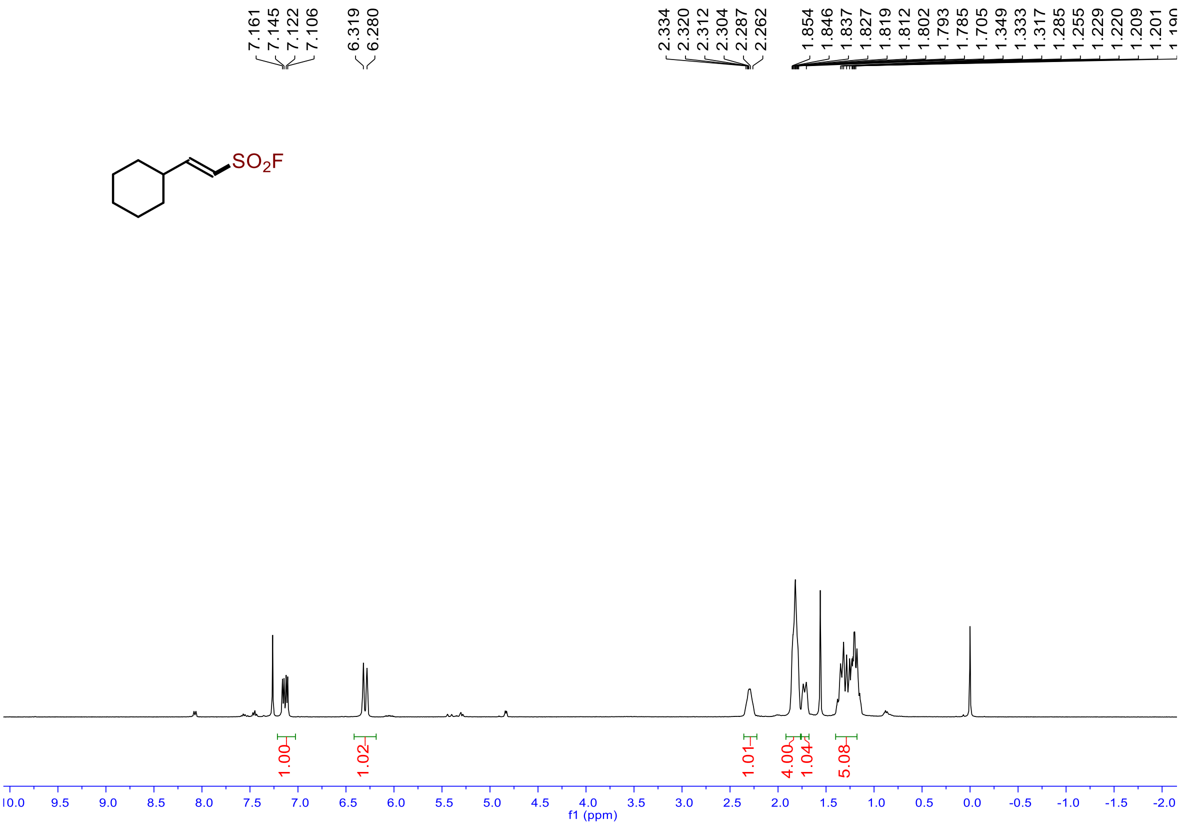

Supplementary Figure 69. ^{1}H NMR spectra of **3ar**

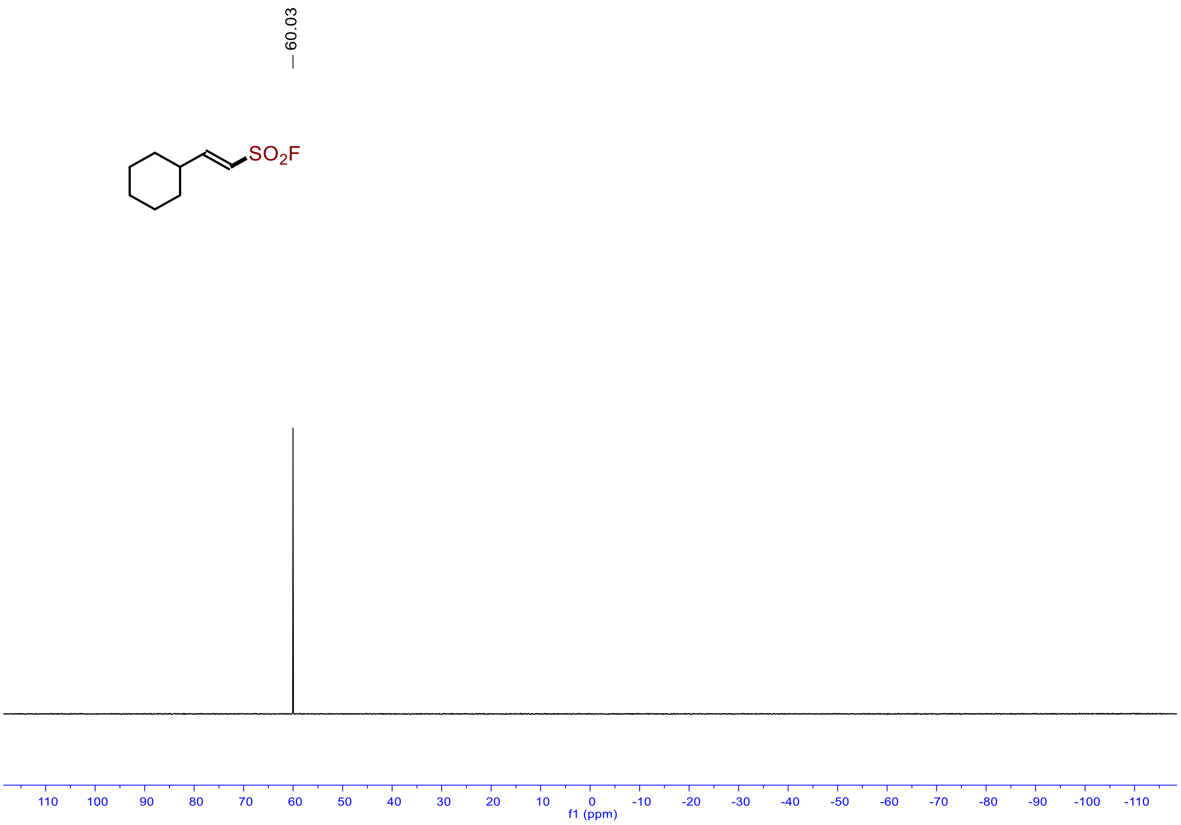

Supplementary Figure 70. ^{19}F NMR spectra of **3ar**

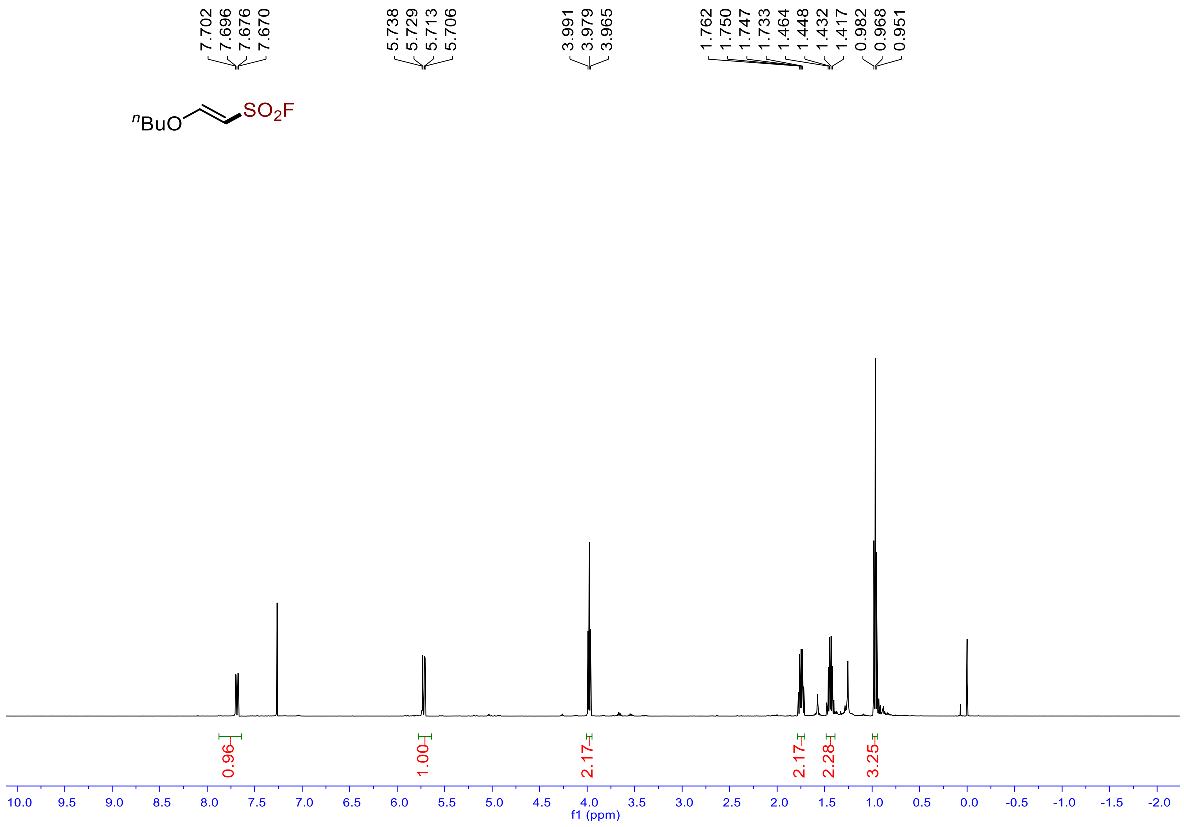

Supplementary Figure 71. ^{1}H NMR spectra of **3as**

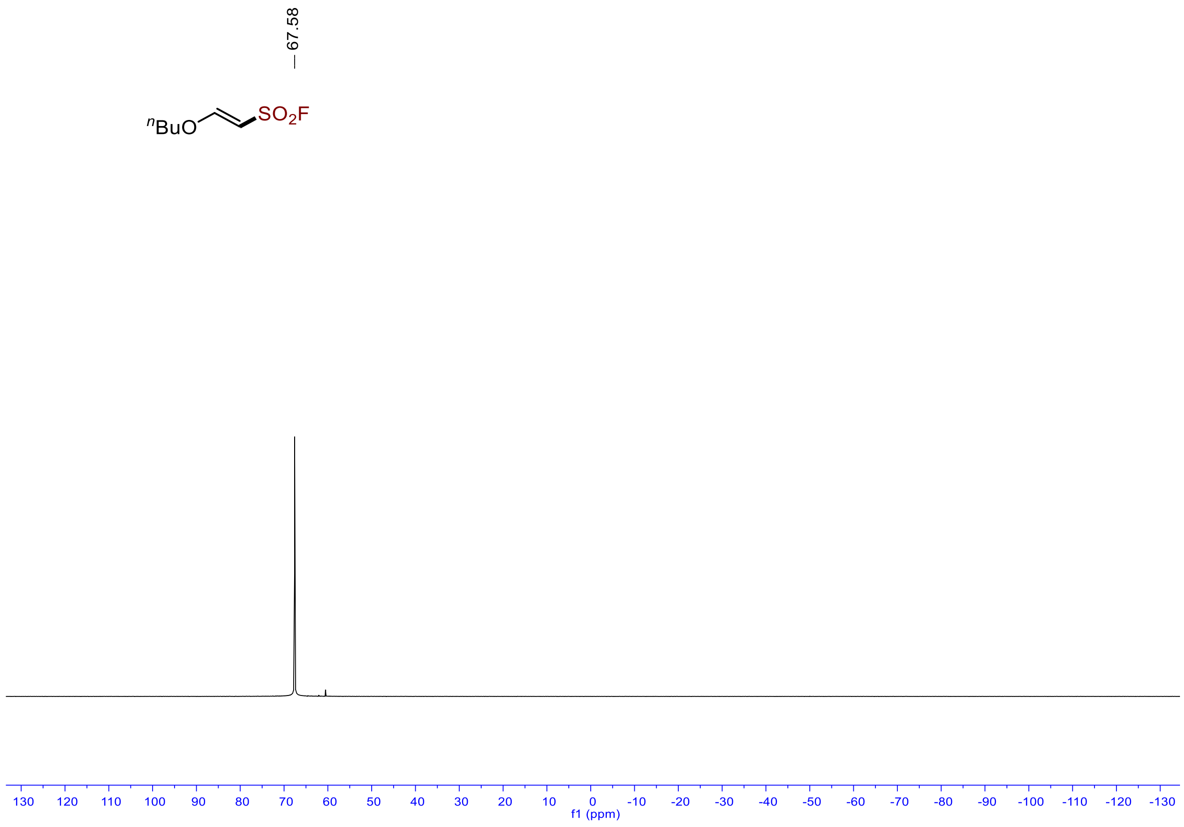

Supplementary Figure 72. ^{19}F NMR spectra of **3as**

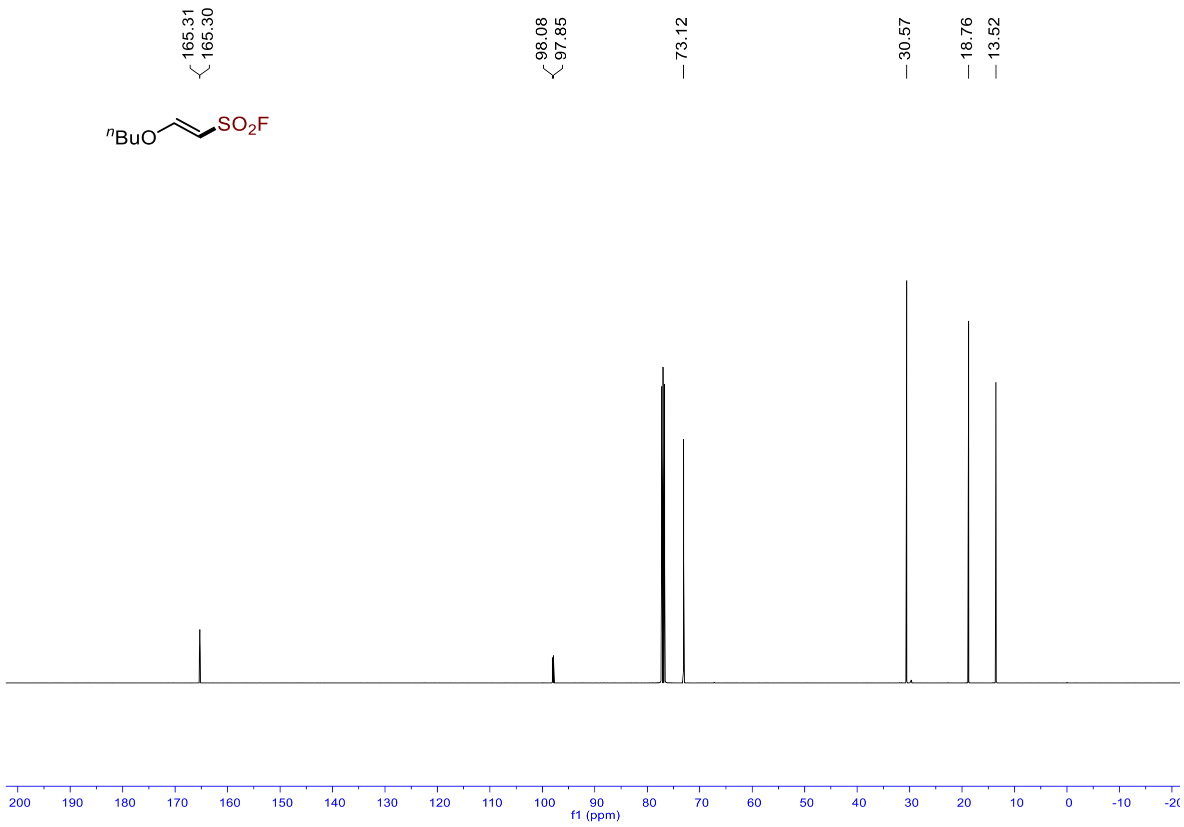

Supplementary Figure 73. ^{13}C NMR spectra of **3as**

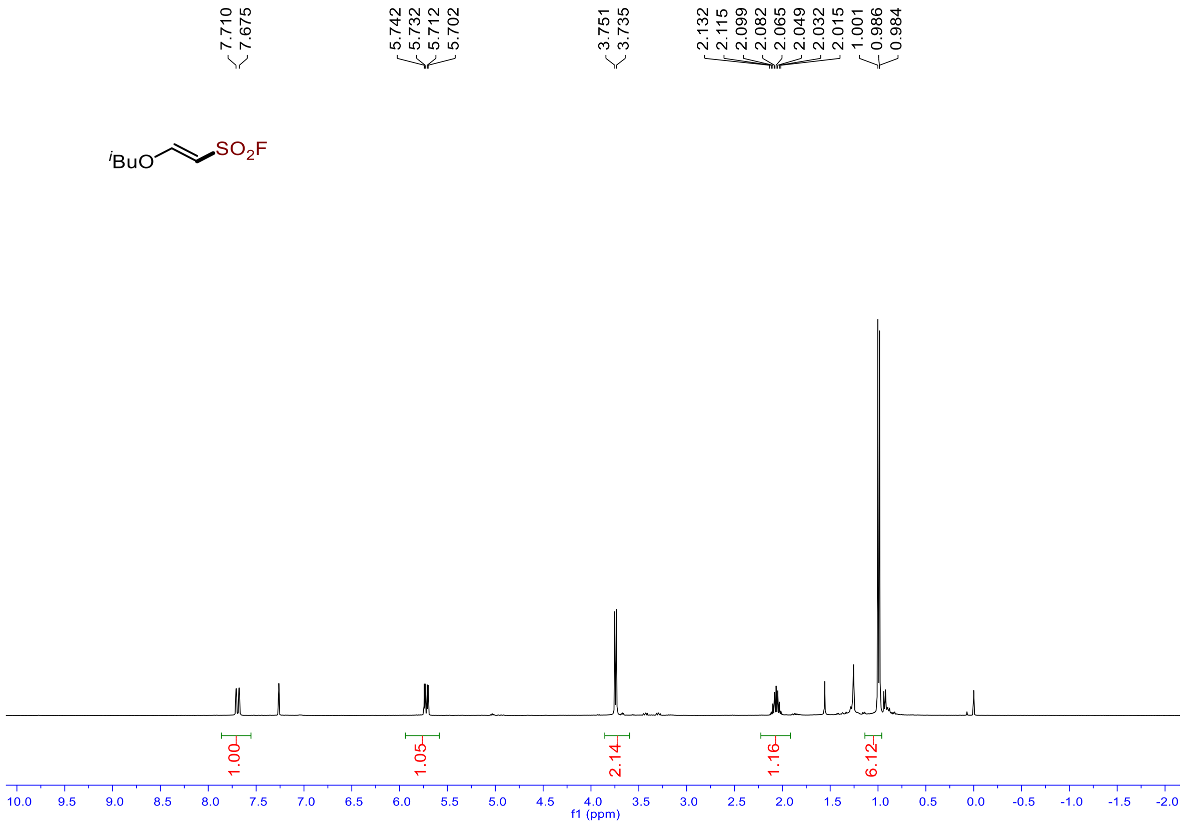

Supplementary Figure 74. ^{1}H NMR spectra of **3at**

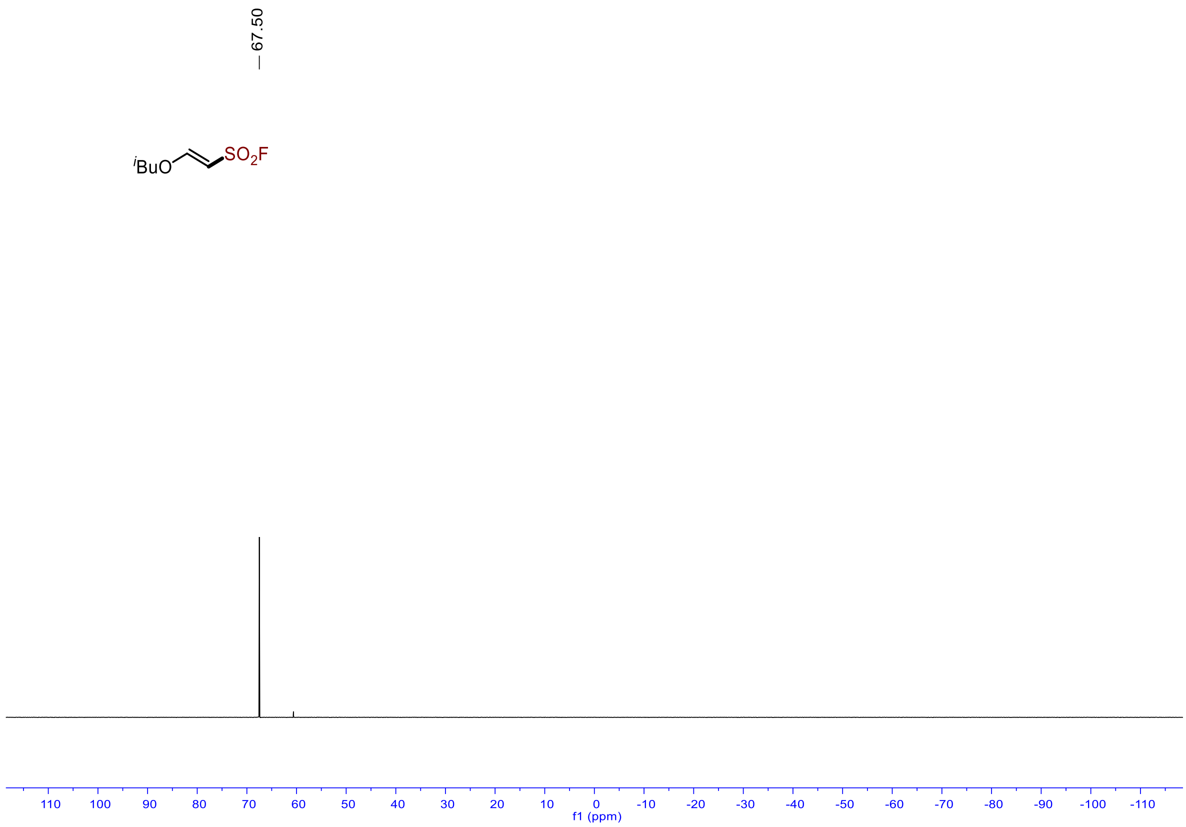

Supplementary Figure 75. ^{19}F NMR spectra of **3at**

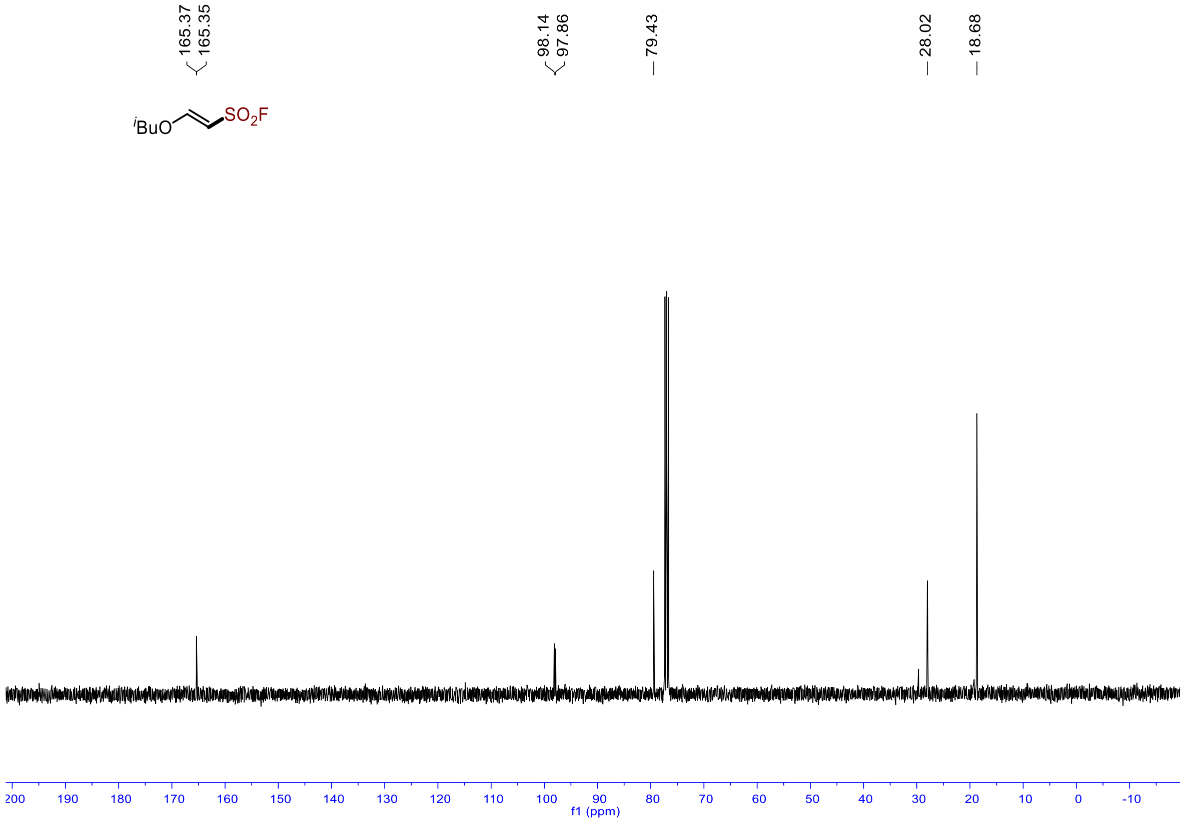

Supplementary Figure 76. ^{13}C NMR spectra of **3at**

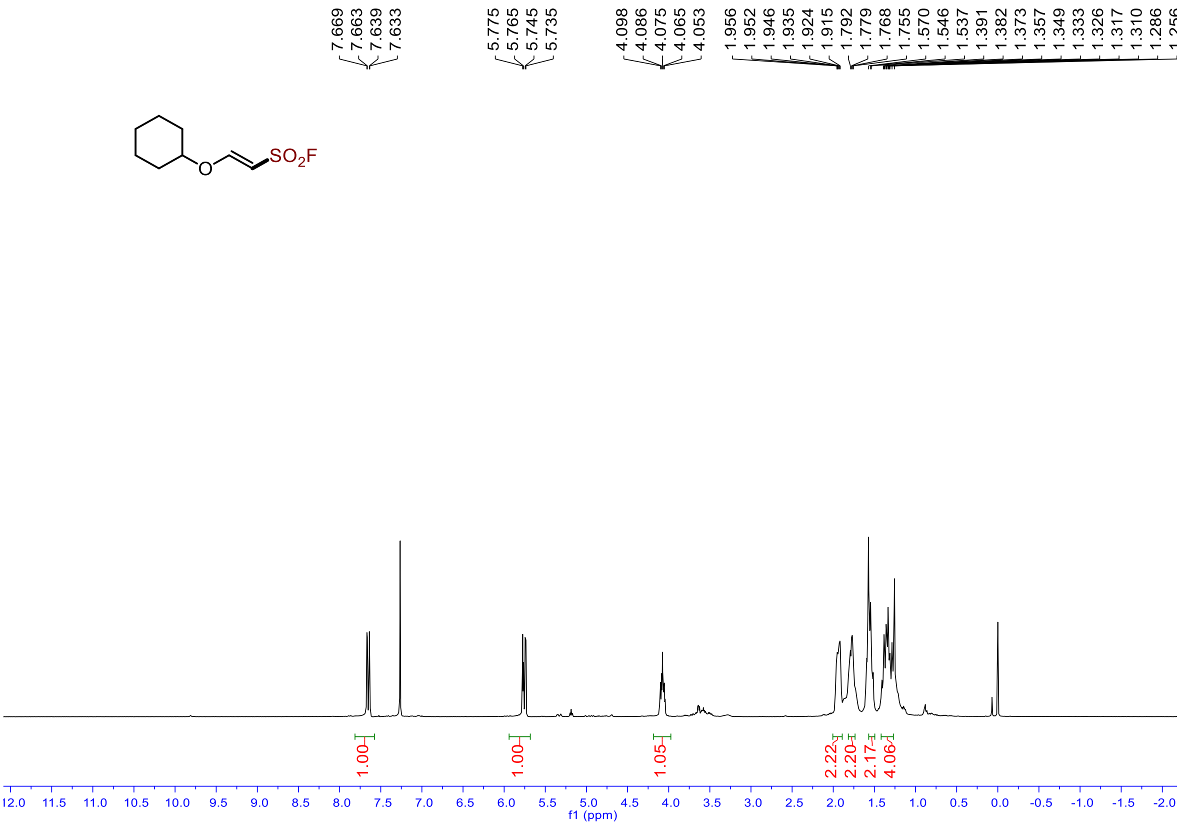

Supplementary Figure 77. ^{1}H NMR spectra of **3au**

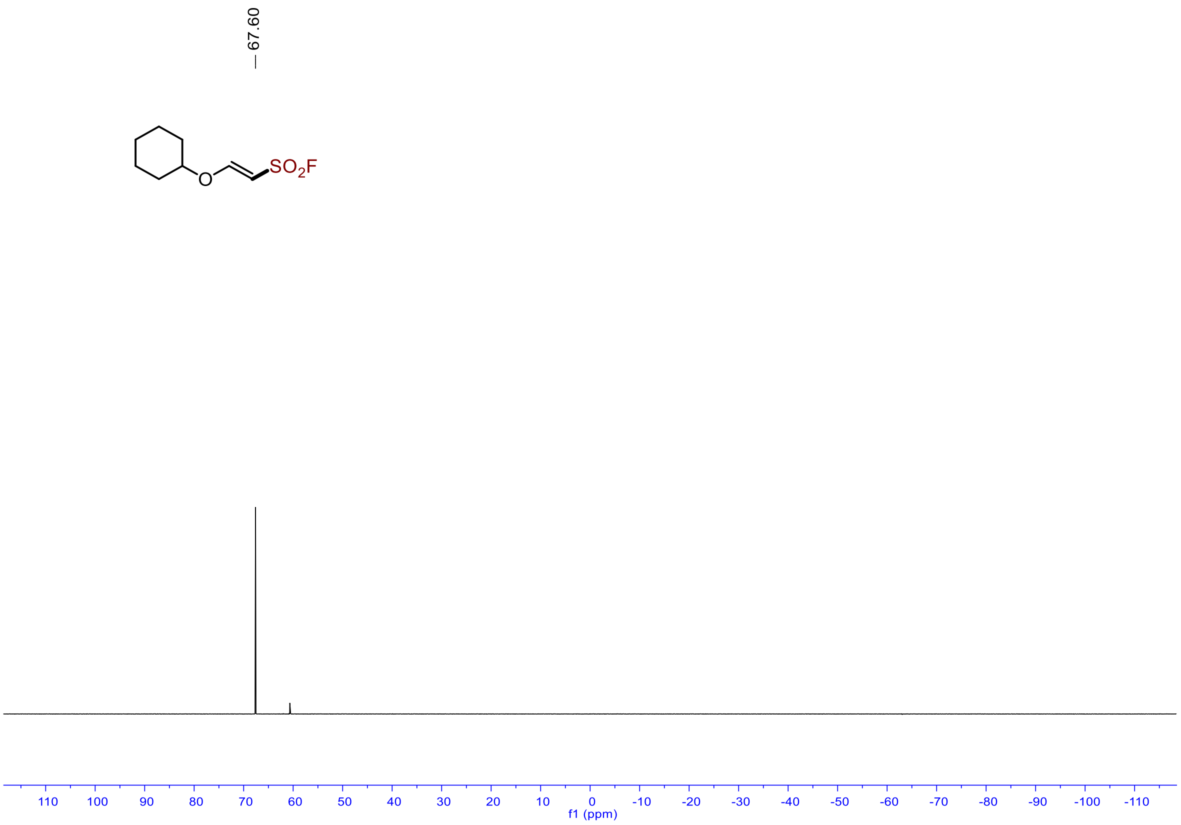

Supplementary Figure 78. ^{19}F NMR spectra of **3au**

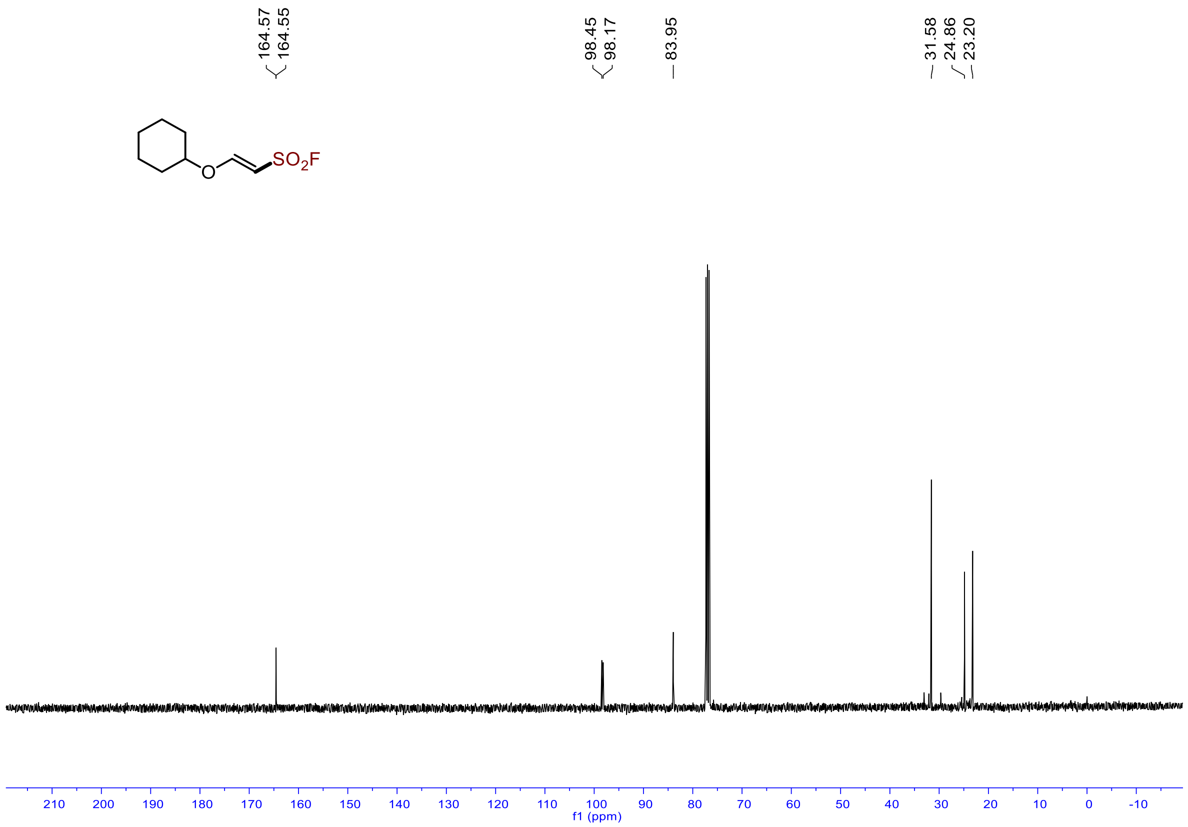

Supplementary Figure 79. ^{13}C NMR spectra of **3au**

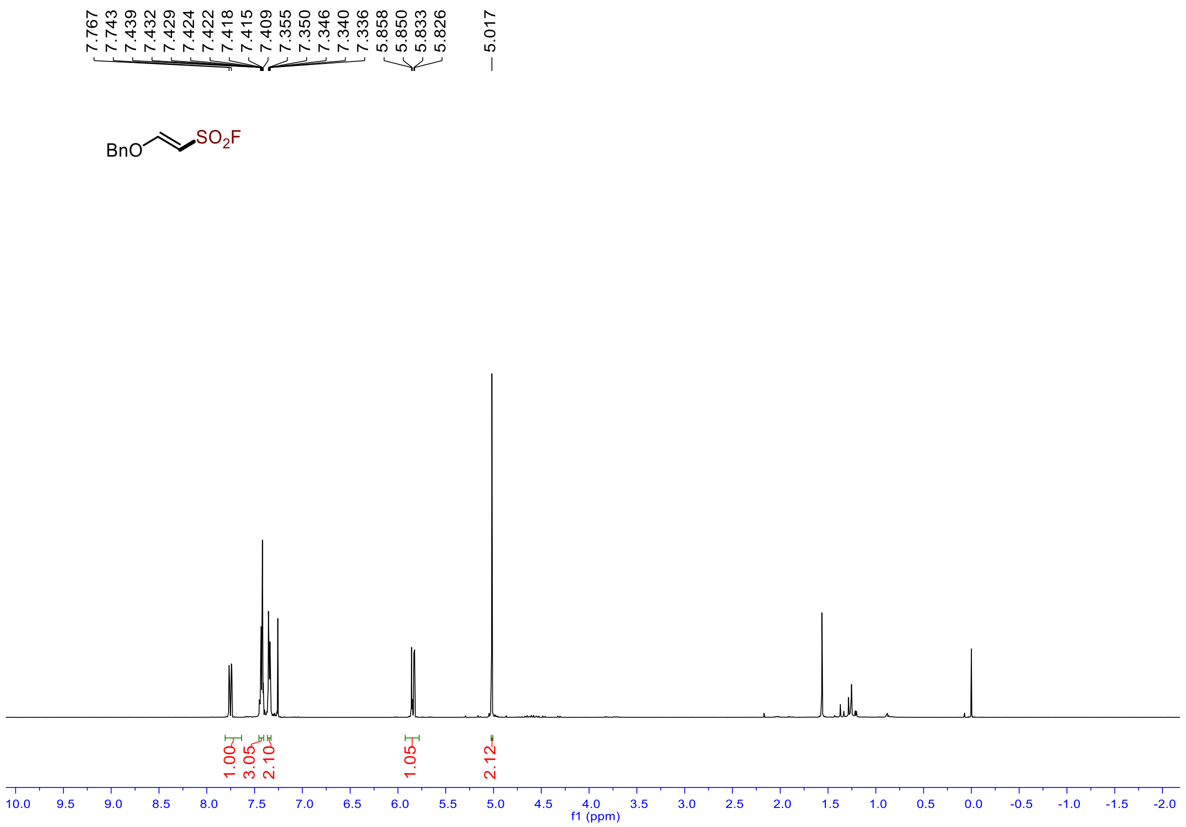

Supplementary Figure 80. ^{1}H NMR spectra of **3av**

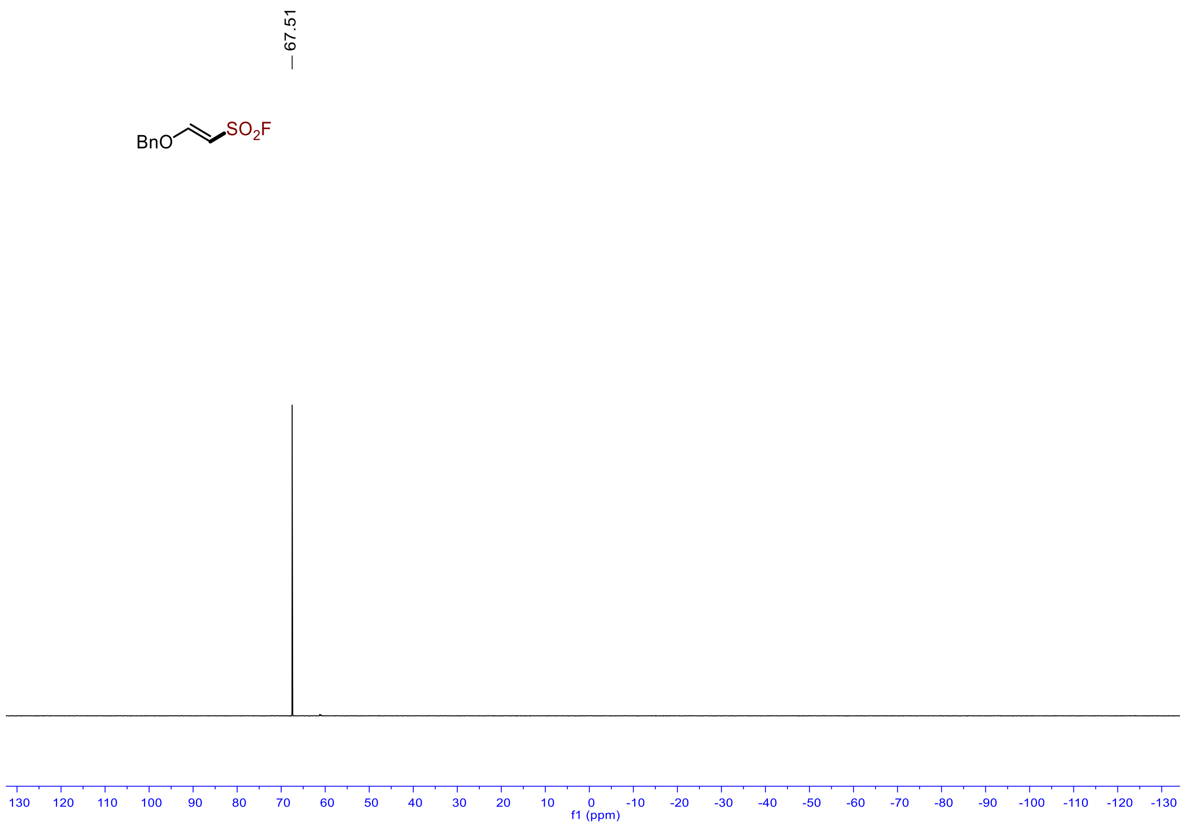

Supplementary Figure 81. ^{19}F NMR spectra of **3av**

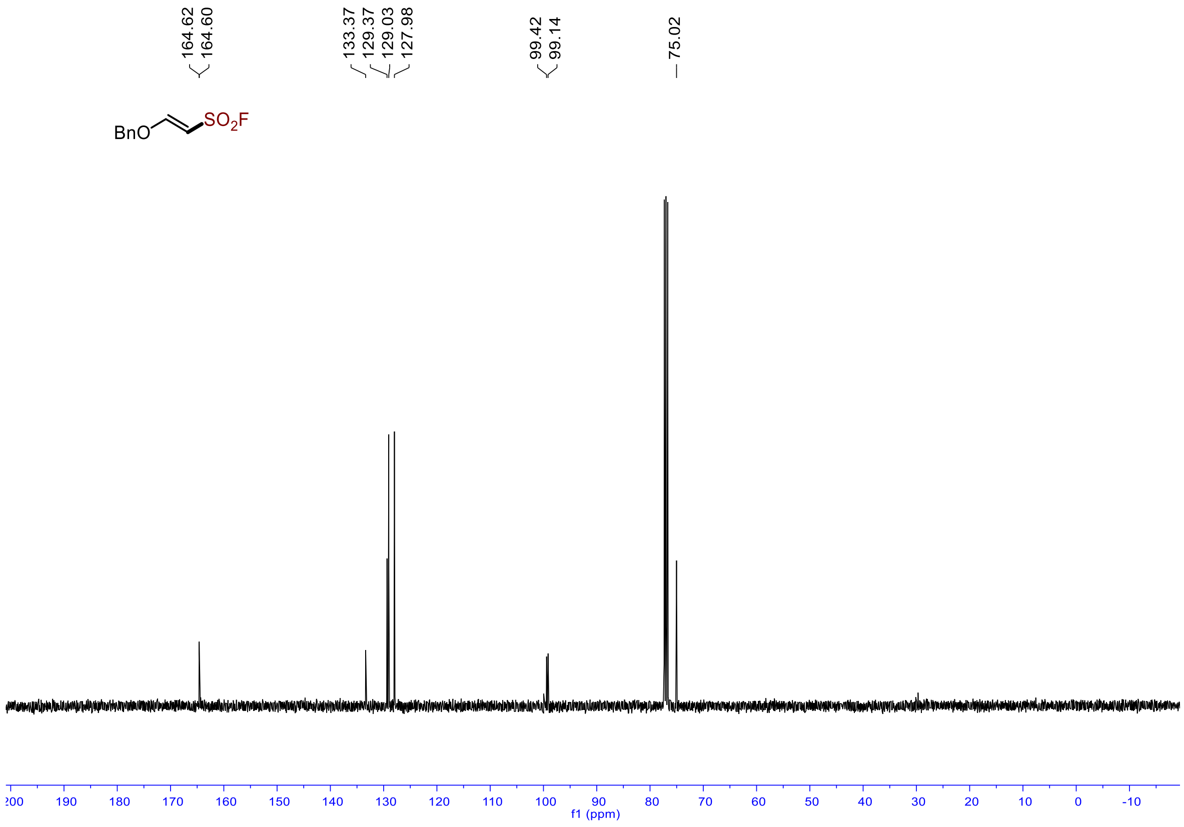

Supplementary Figure 82. ^{13}C NMR spectra of **3av**

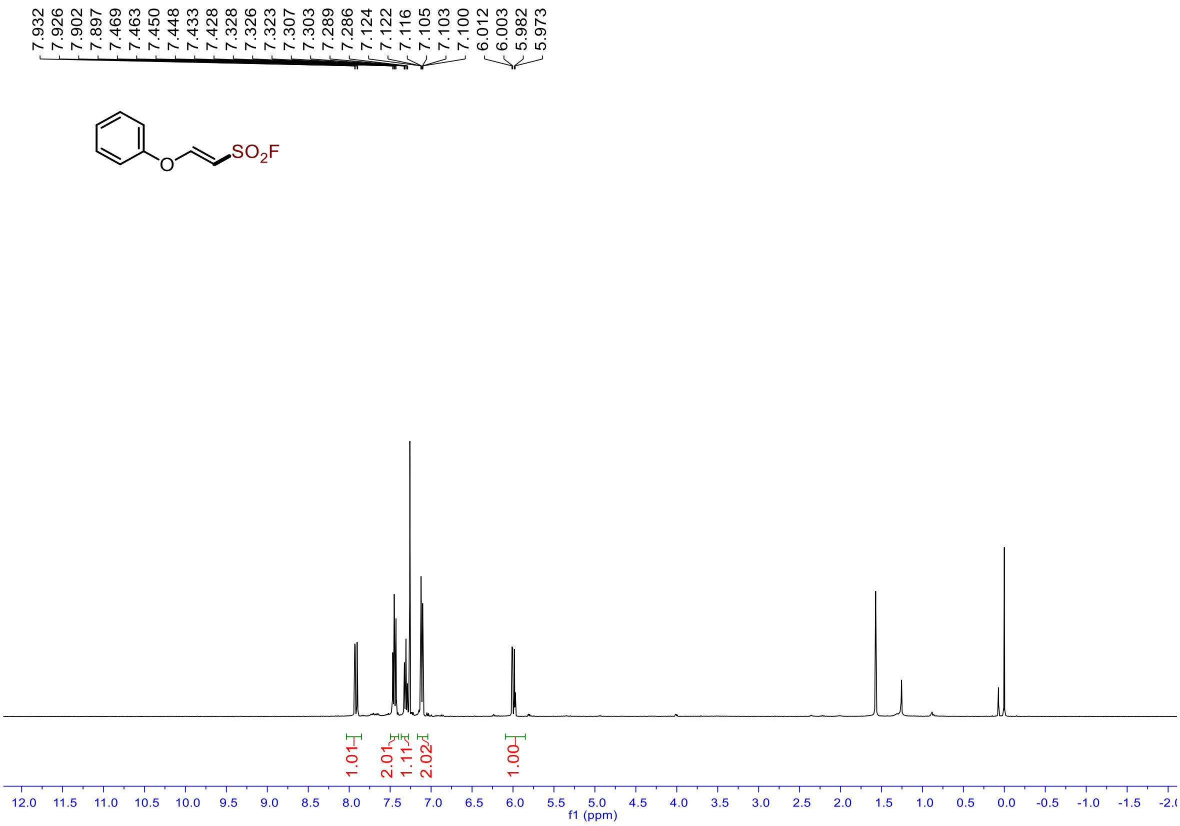

Supplementary Figure 83. ^{1}H NMR spectra of **3aw**

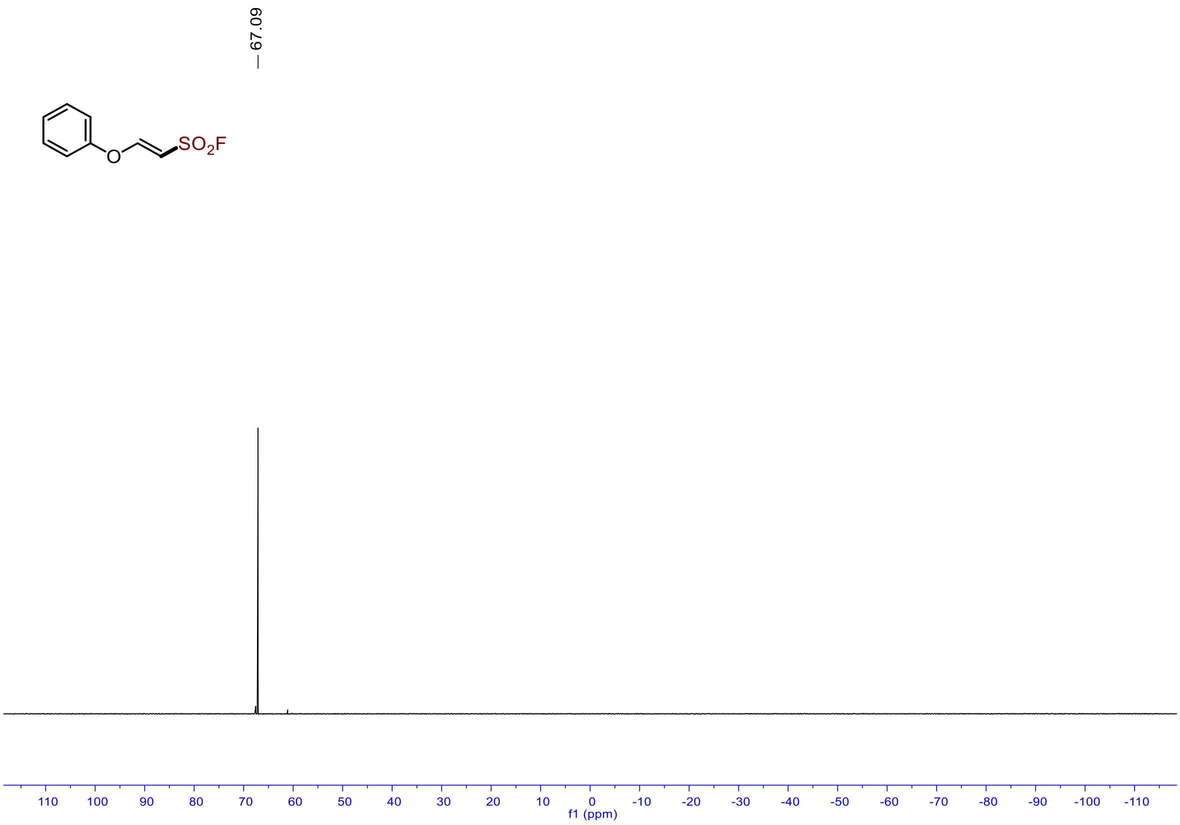

Supplementary Figure 84. ^{19}F NMR spectra of **3aw**

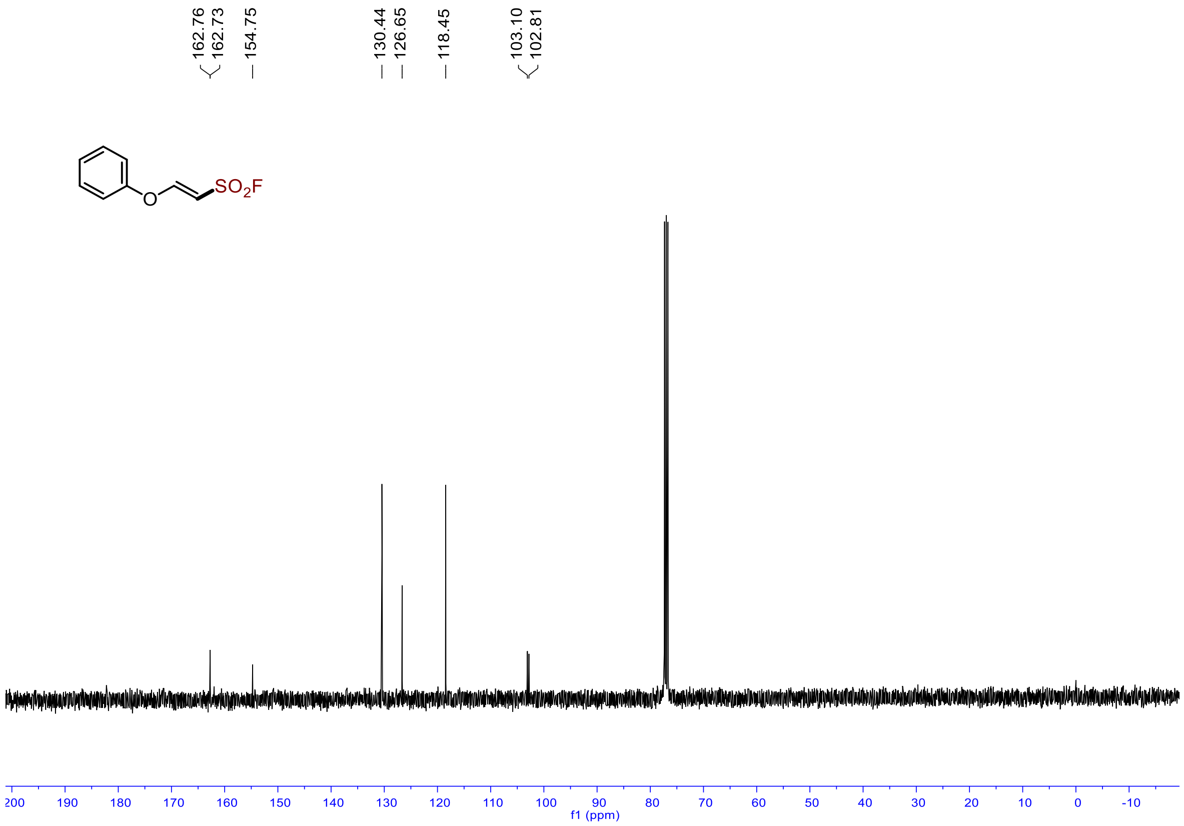

Supplementary Figure 85. ^{13}C NMR spectra of **3aw**

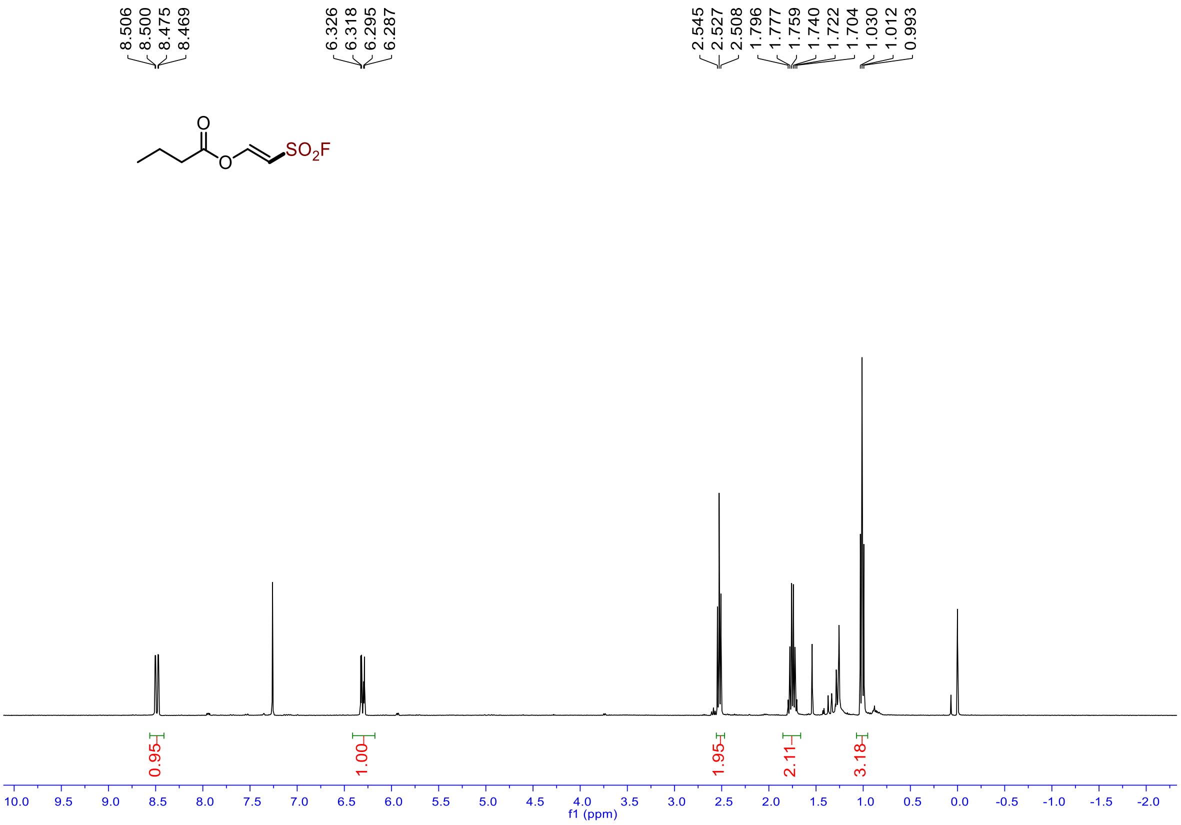

Supplementary Figure 86. ^{1}H NMR spectra of **3ax**

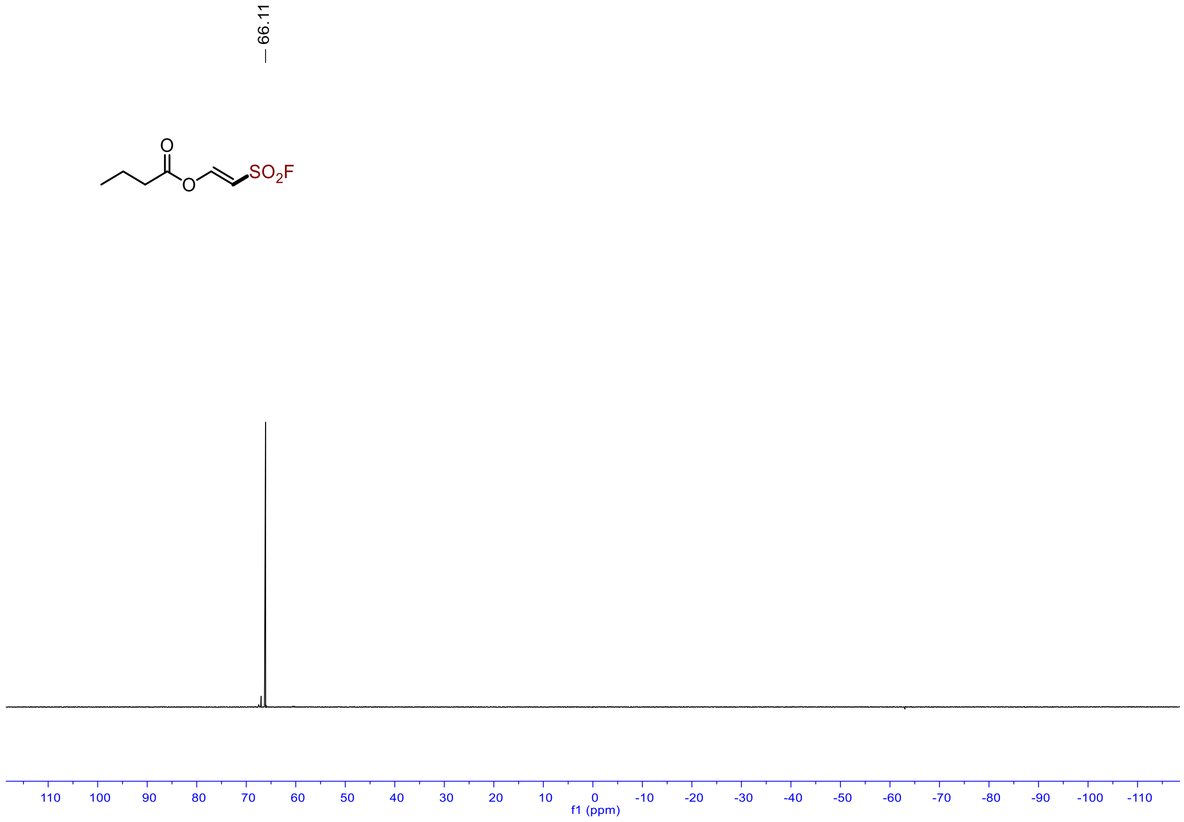

Supplementary Figure 87. ^{19}F NMR spectra of **3ax**

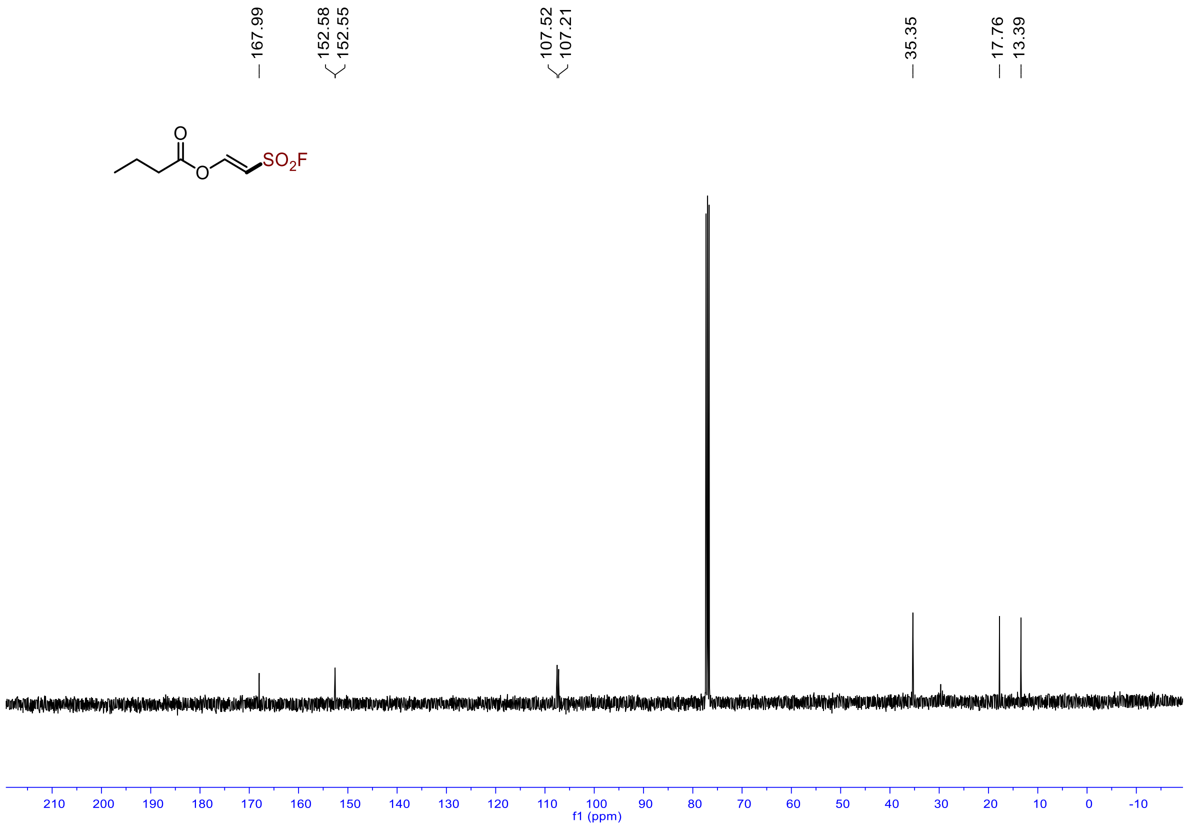

Supplementary Figure 88. ^{13}C NMR spectra of **3ax**

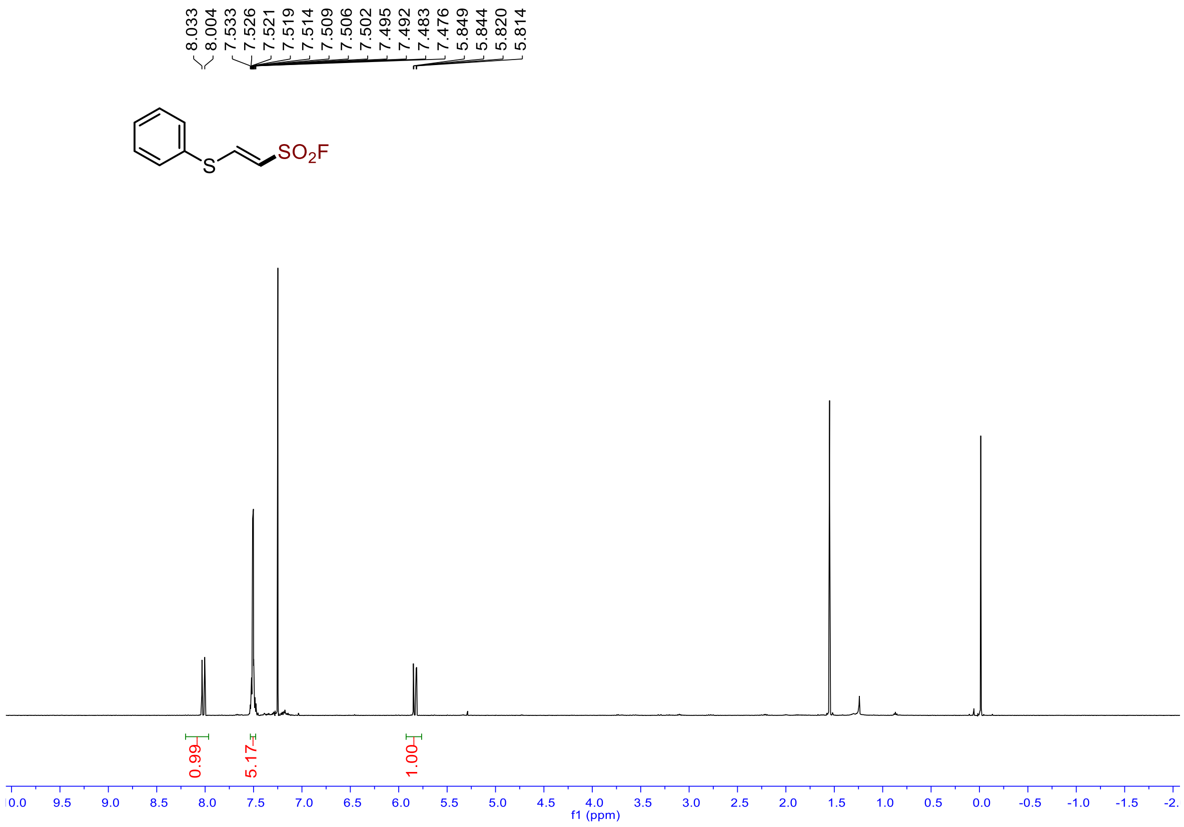

Supplementary Figure 89. ^{1}H NMR spectra of **3ay**

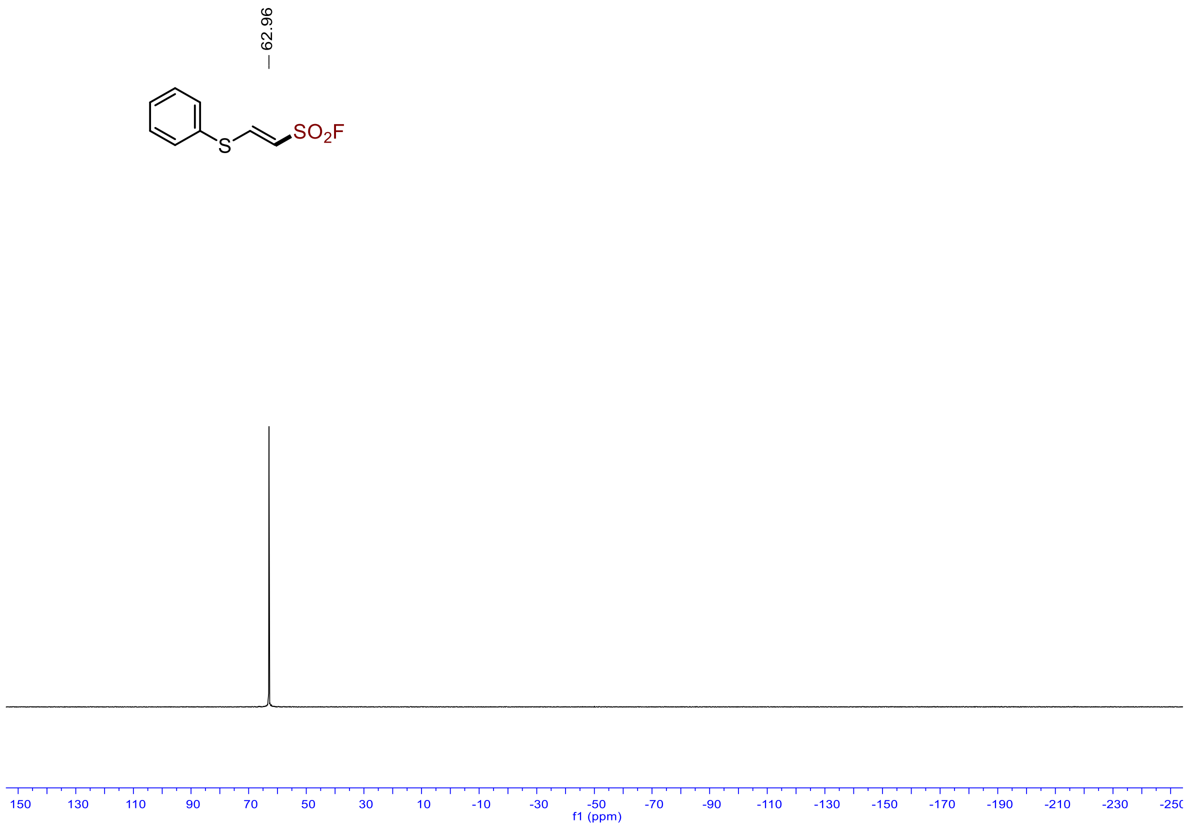

Supplementary Figure 90. ^{19}F NMR spectra of **3ay**

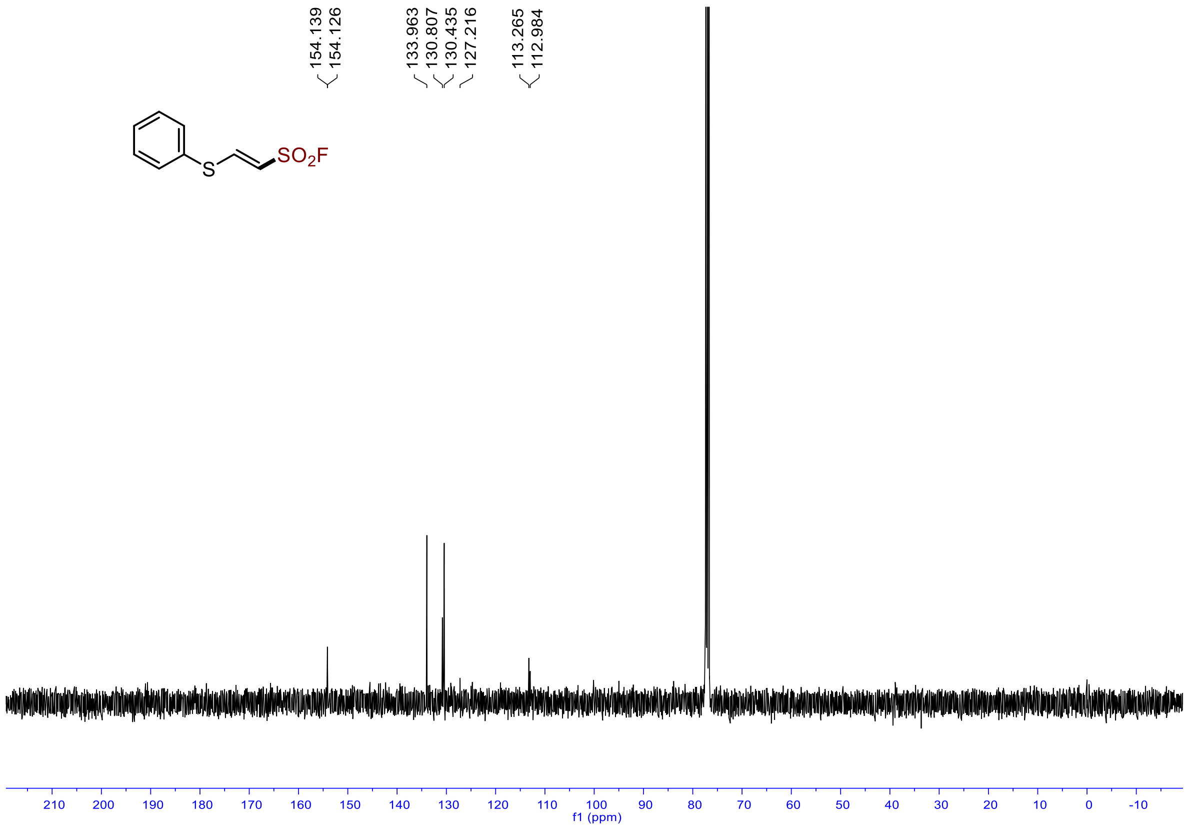

Supplementary Figure 91. ^{13}C NMR spectra of **3ay**

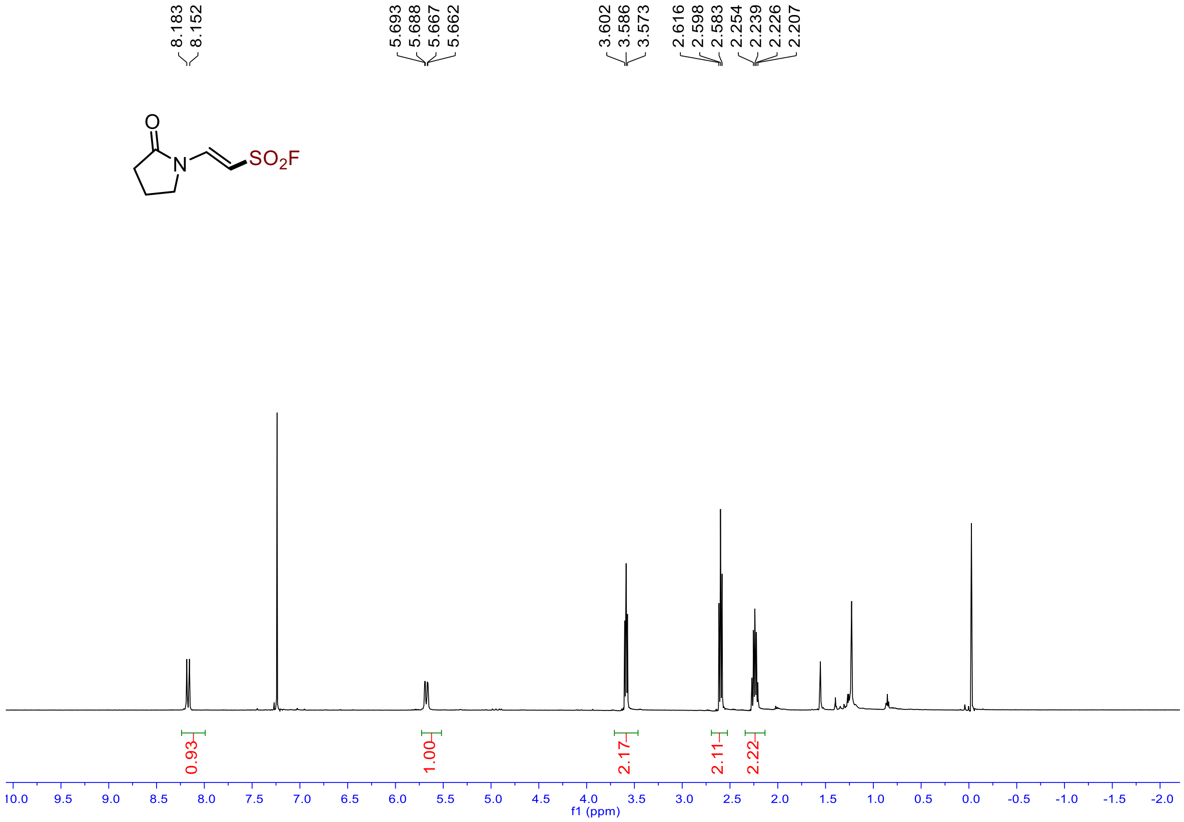

Supplementary Figure 92. ^{1}H NMR spectra of **3az**

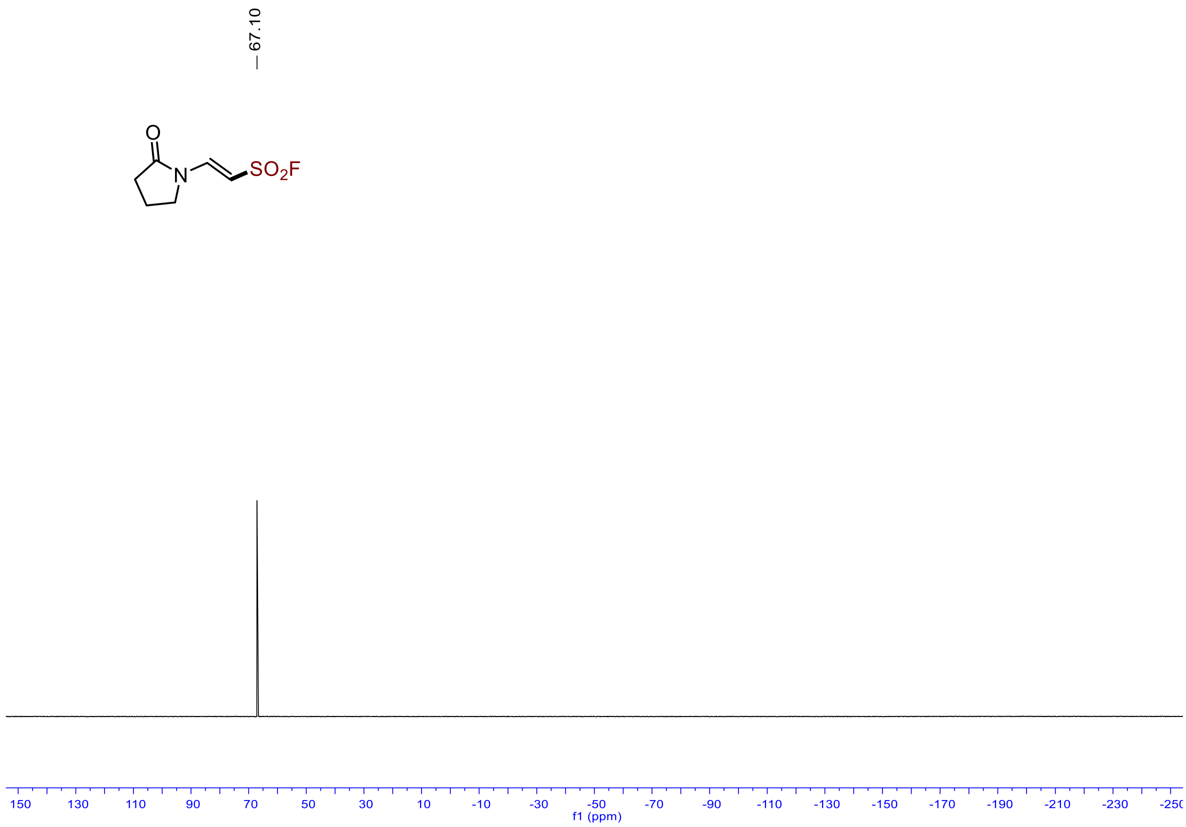

Supplementary Figure 93. ^{19}F NMR spectra of **3az**

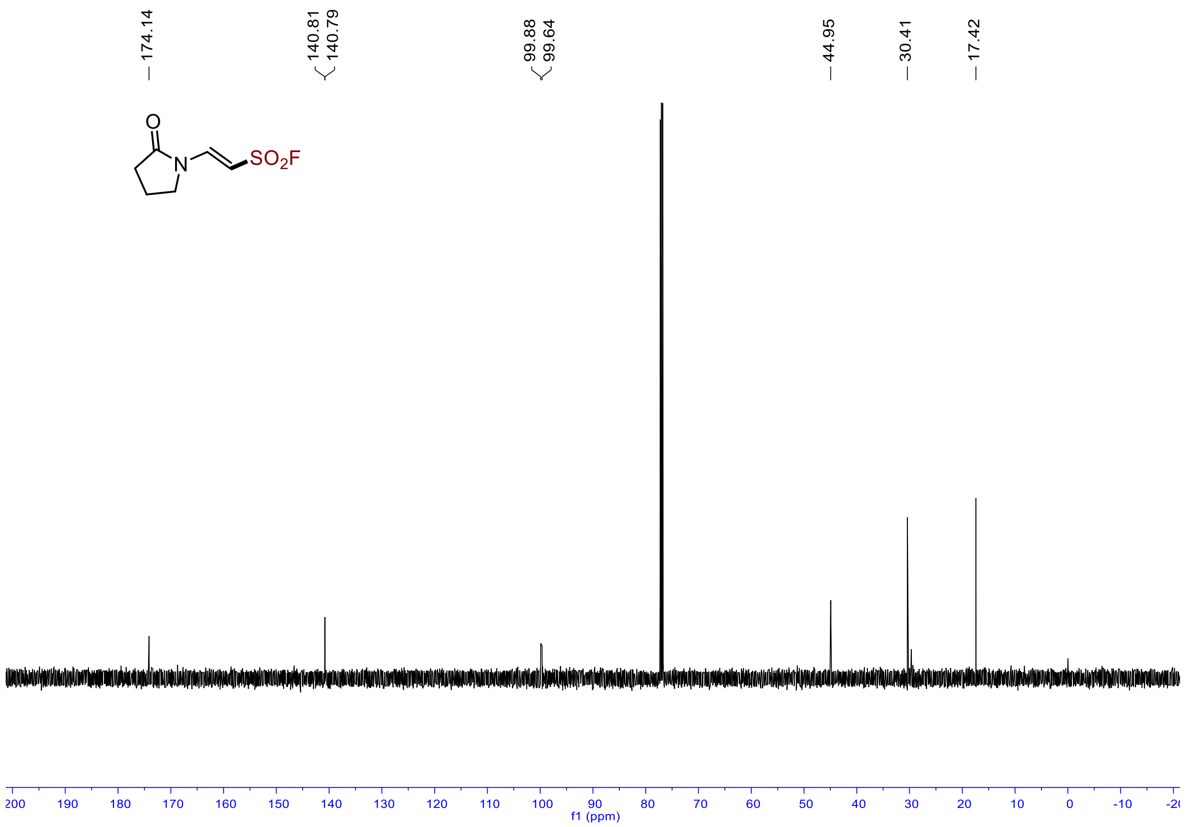

Supplementary Figure 94. ^{13}C NMR spectra of **3az**

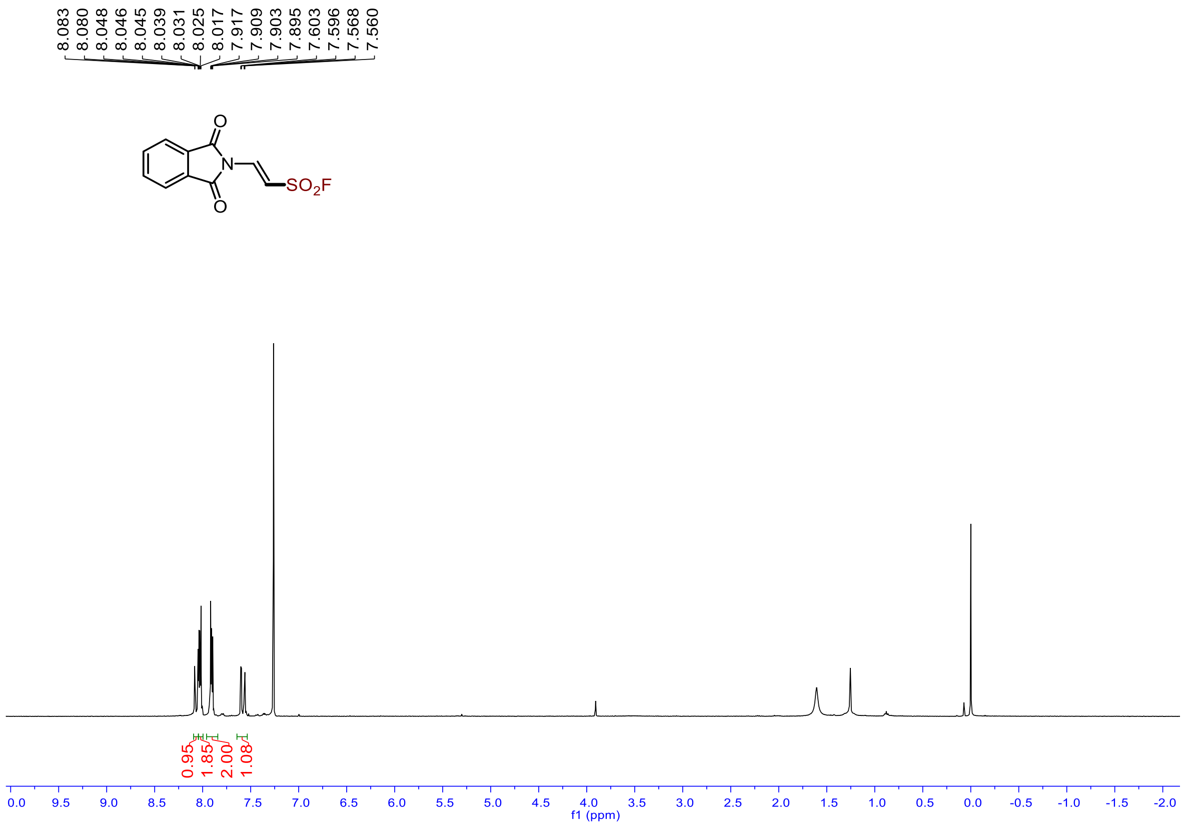

Supplementary Figure 95. ^{1}H NMR spectra of **3ba**

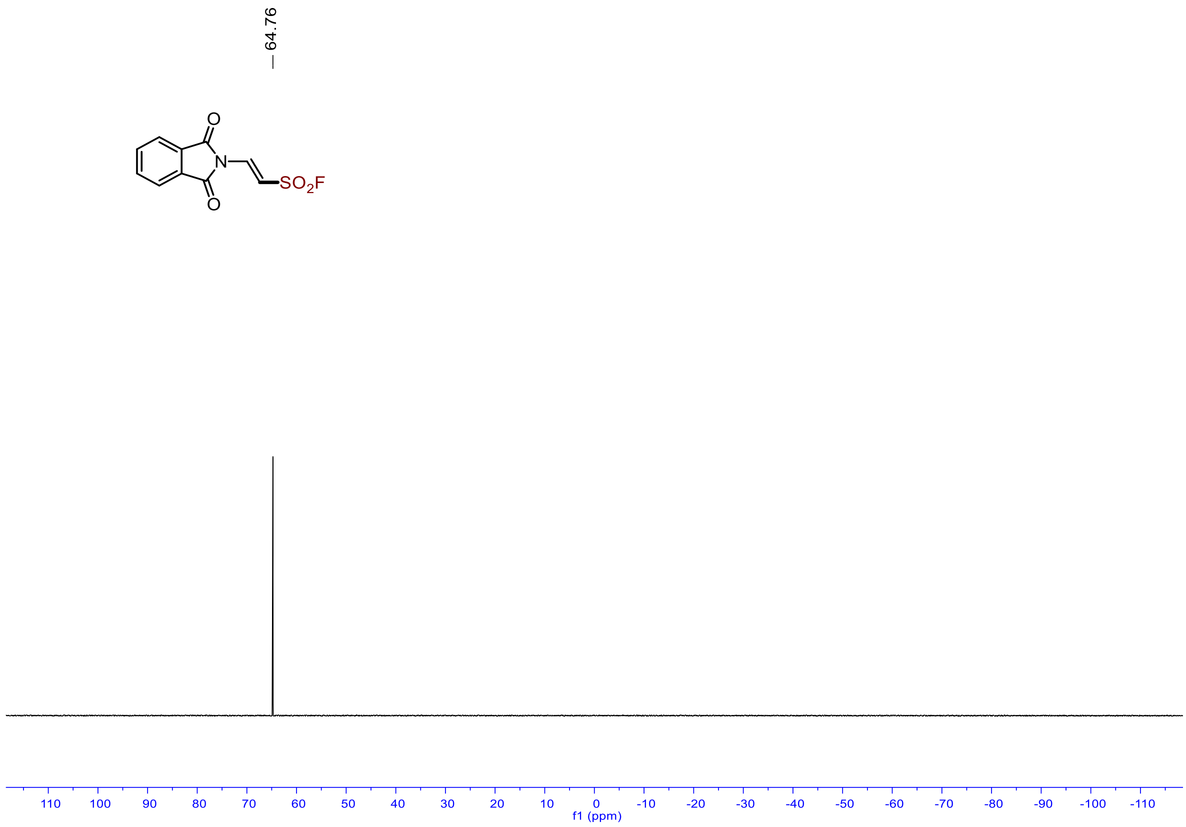

Supplementary Figure 96. ^{19}F NMR spectra of **3ba**

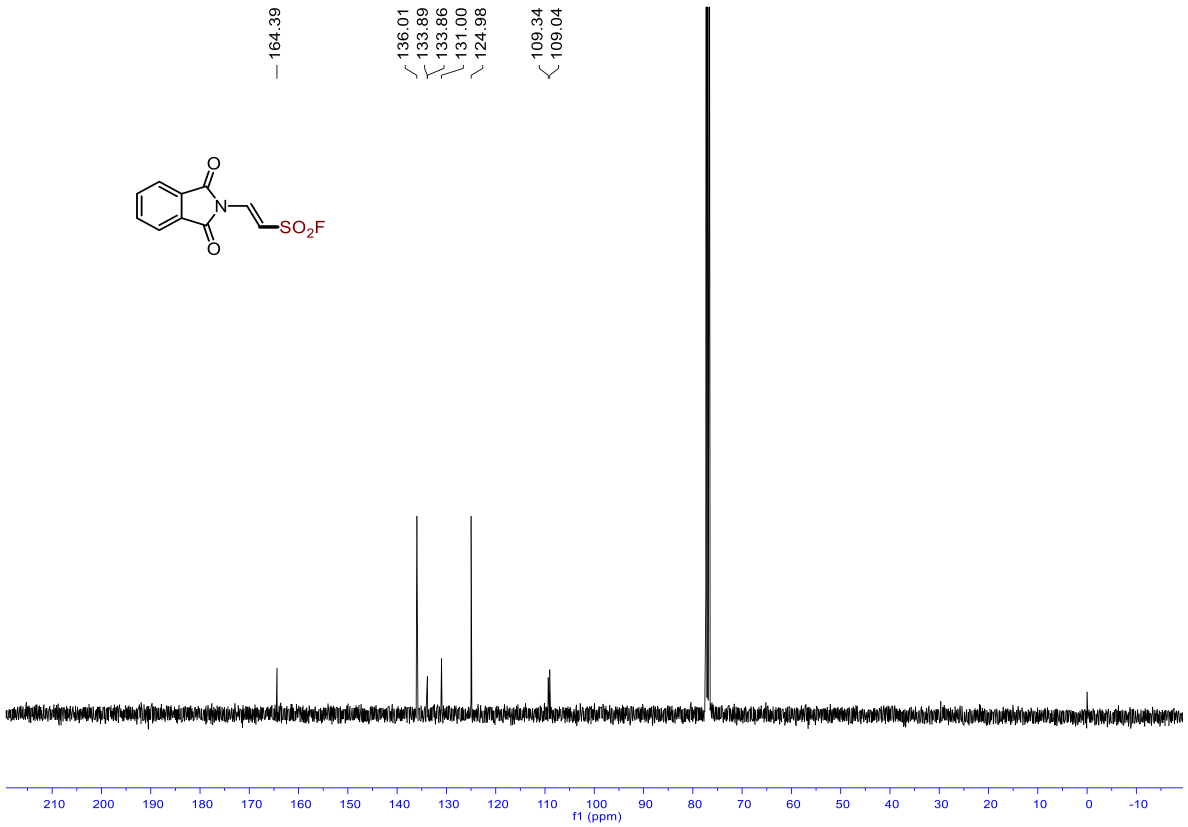

Supplementary Figure 97. ^{13}C NMR spectra of **3ba**

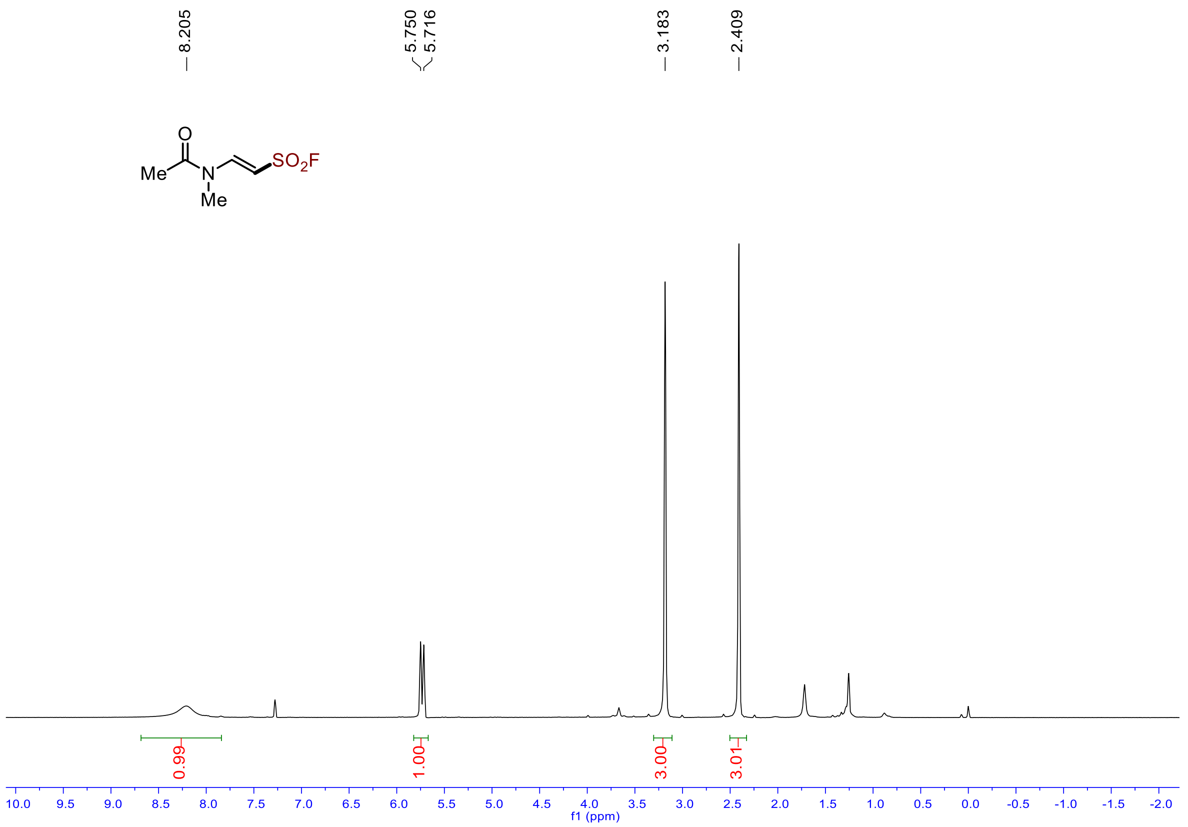

Supplementary Figure 98. ^{1}H NMR spectra of **3bb**

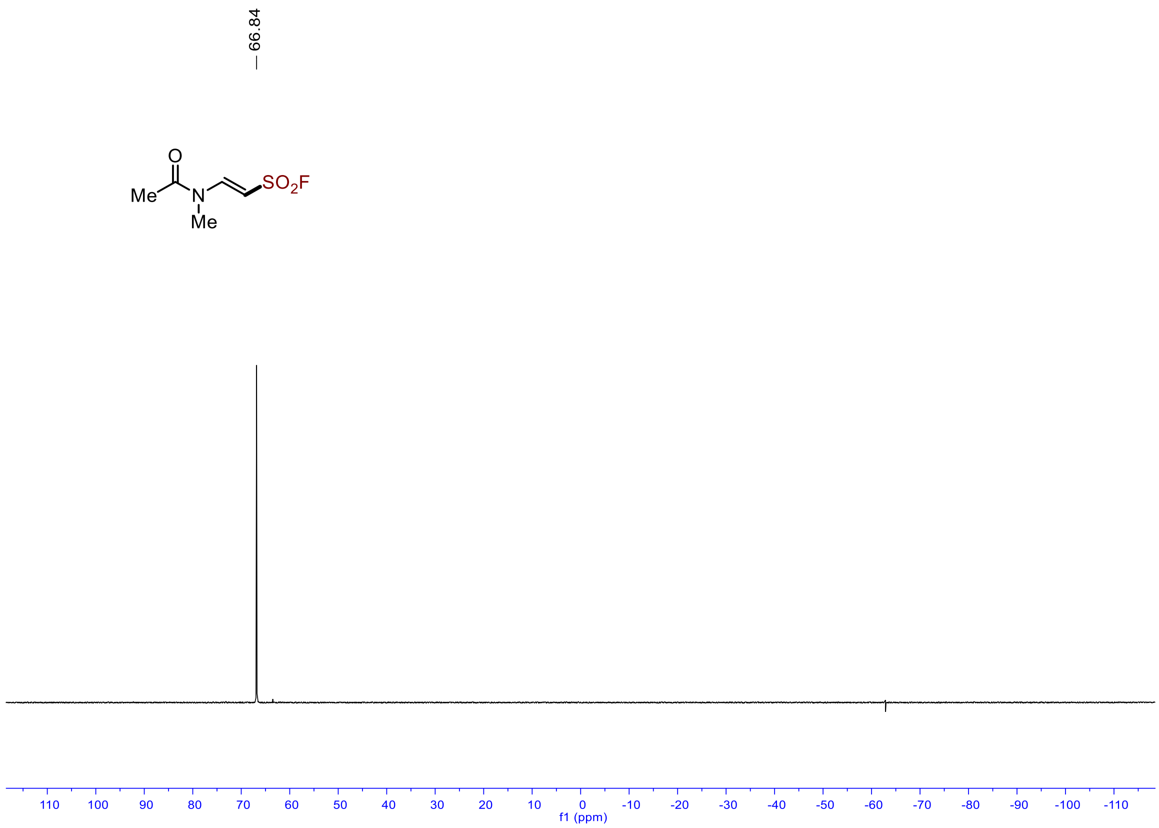

Supplementary Figure 99. ^{19}F NMR spectra of **3bb**

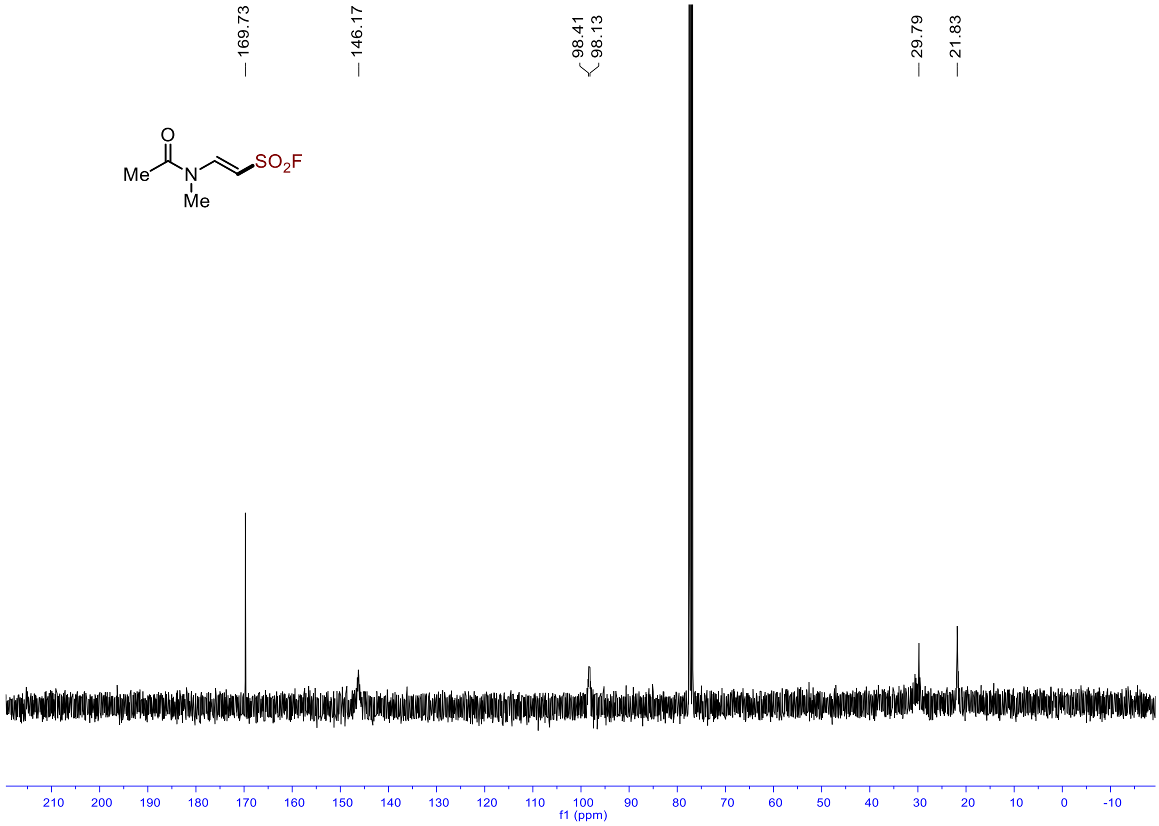

Supplementary Figure 100. ^{13}C NMR spectra of **3bb**

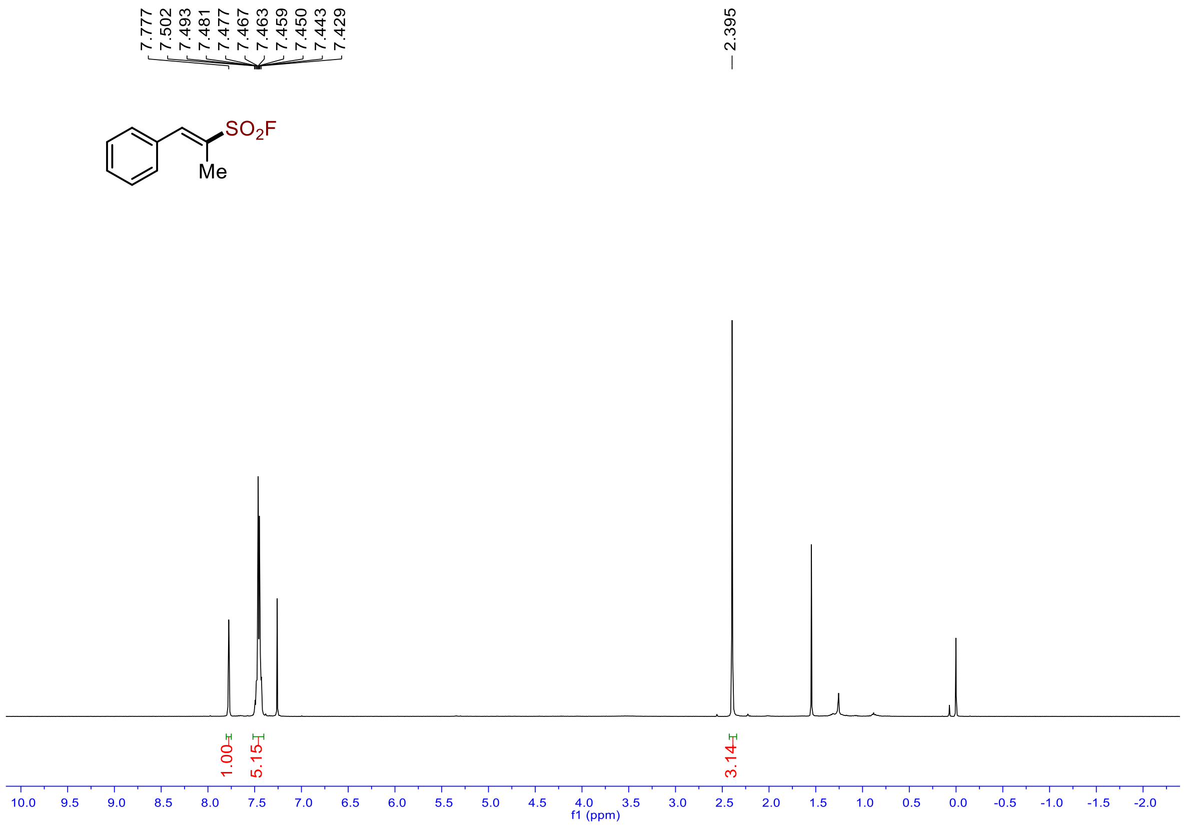

Supplementary Figure 101. ^{1}H NMR spectra of **5aa**

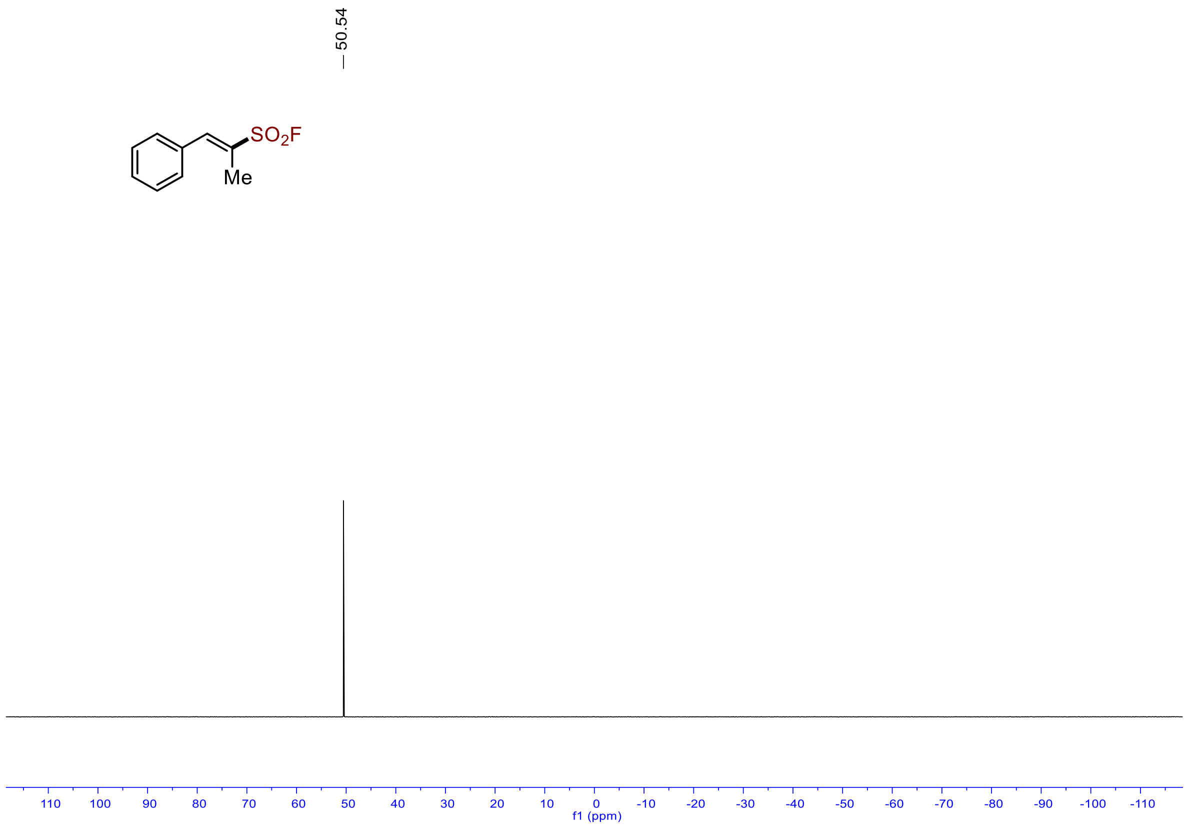

Supplementary Figure 102. ^{19}F NMR spectra of **5aa**

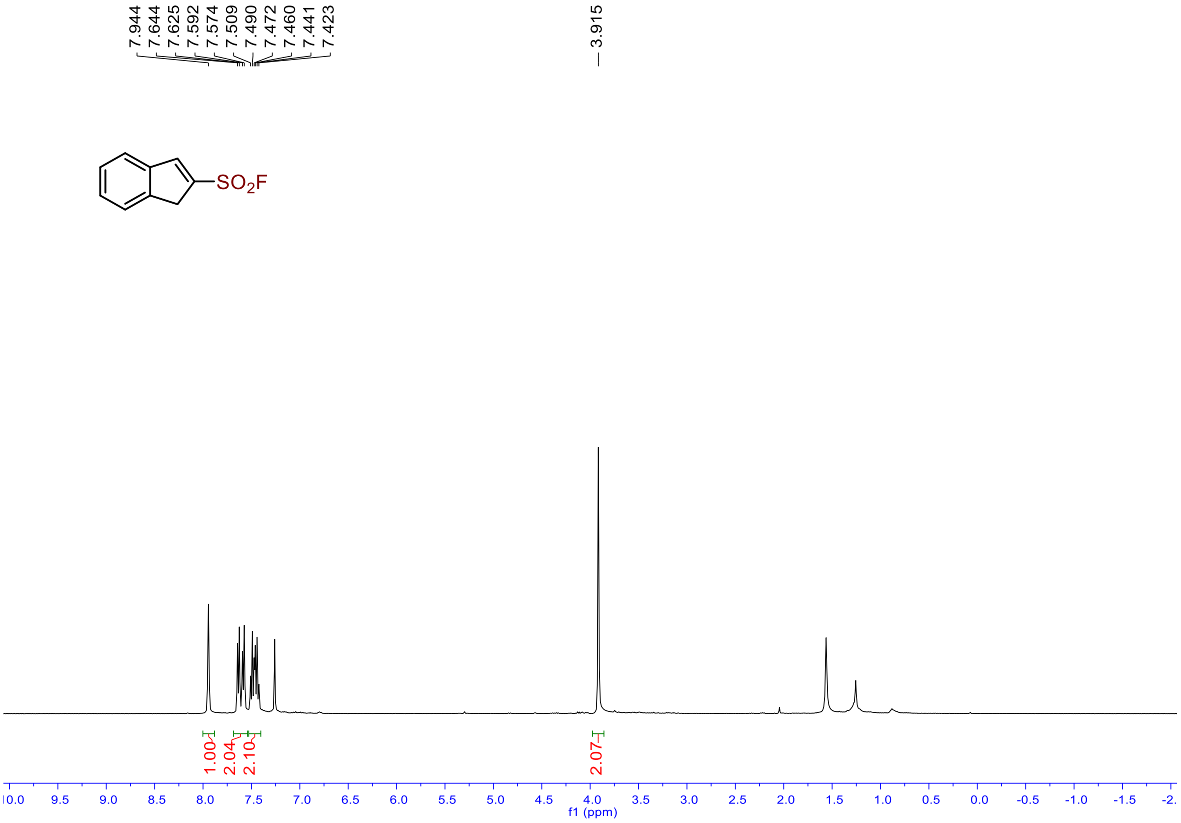

Supplementary Figure 103. ^{1}H NMR spectra of **5ab**

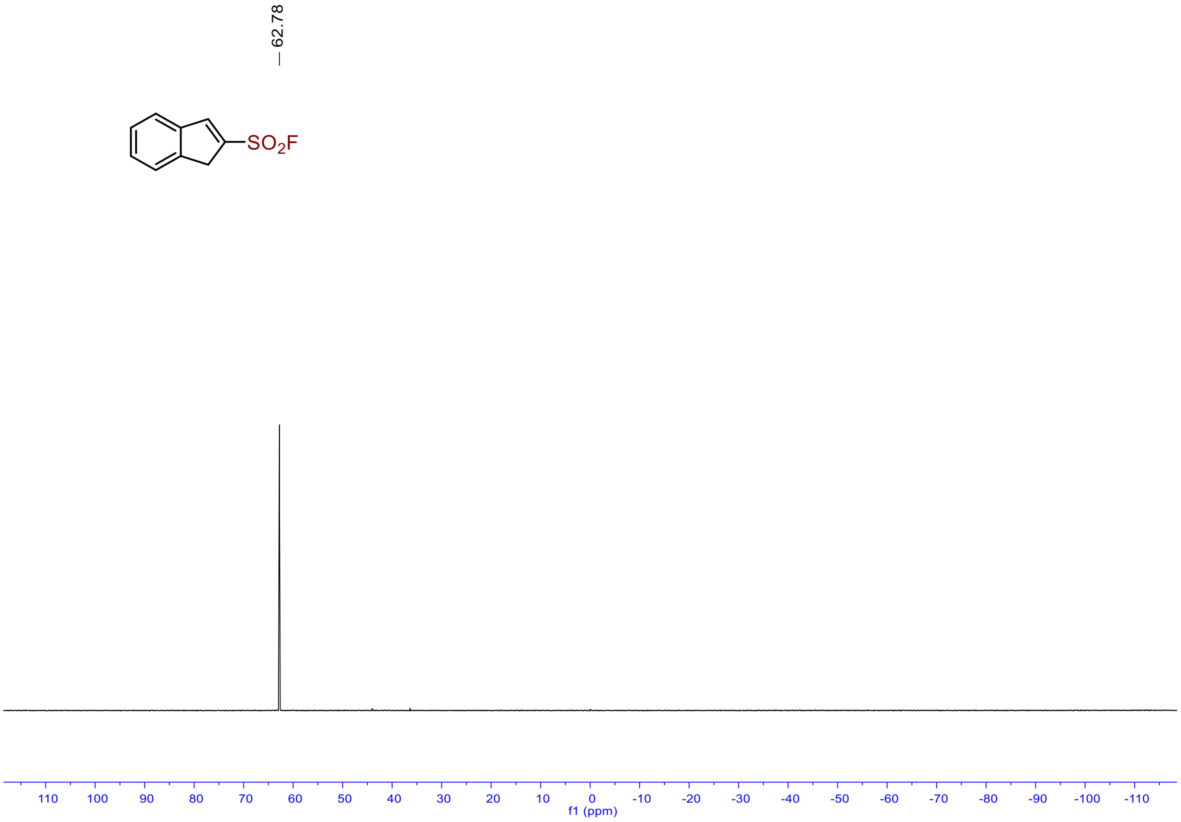

Supplementary Figure 104. ^{19}F NMR spectra of **5ab**

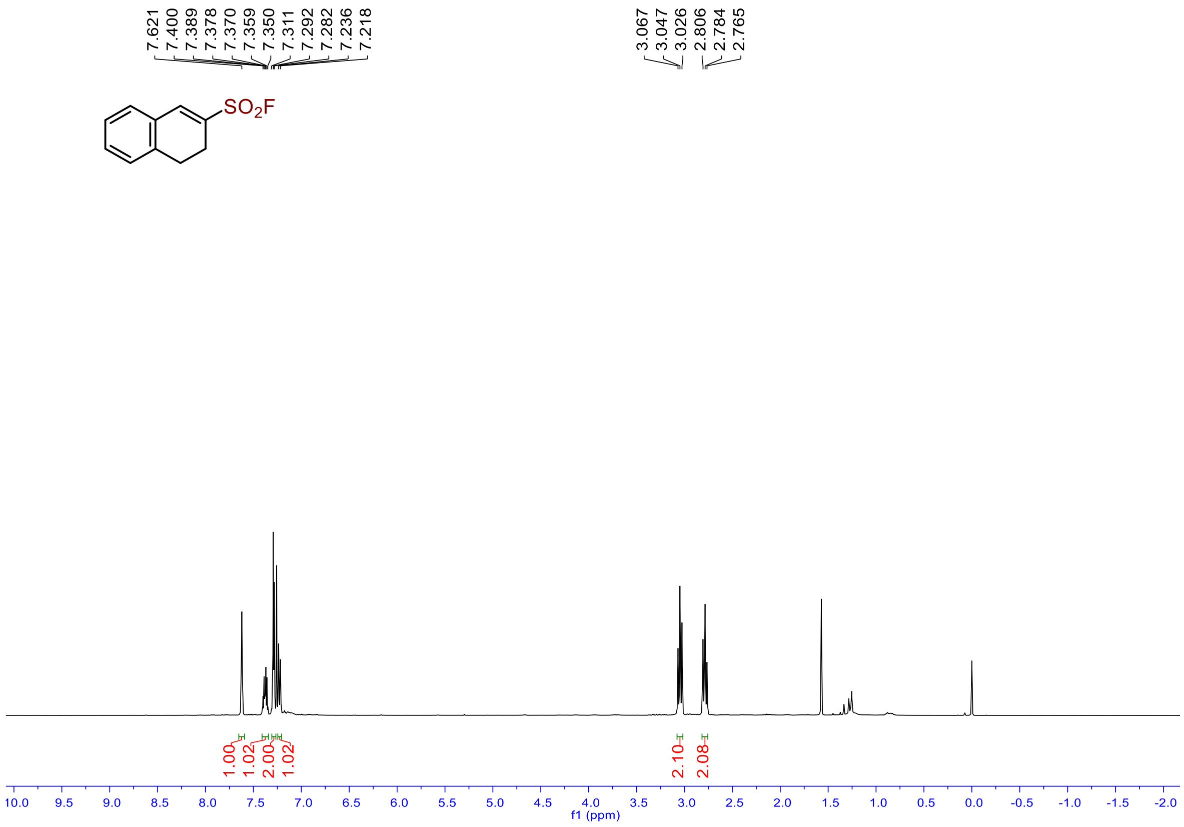

Supplementary Figure 105. ^{1}H NMR spectra of **5ac**

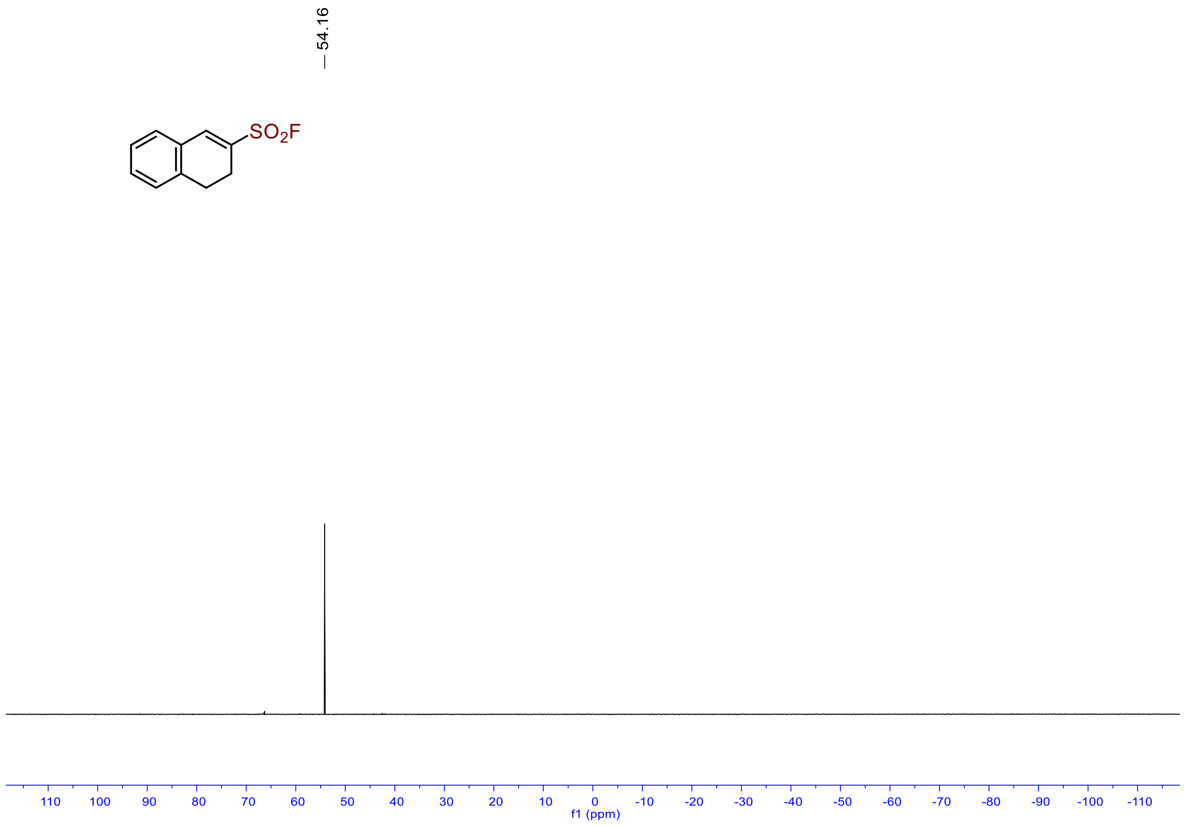

Supplementary Figure 106. ^{19}F NMR spectra of **5ac**

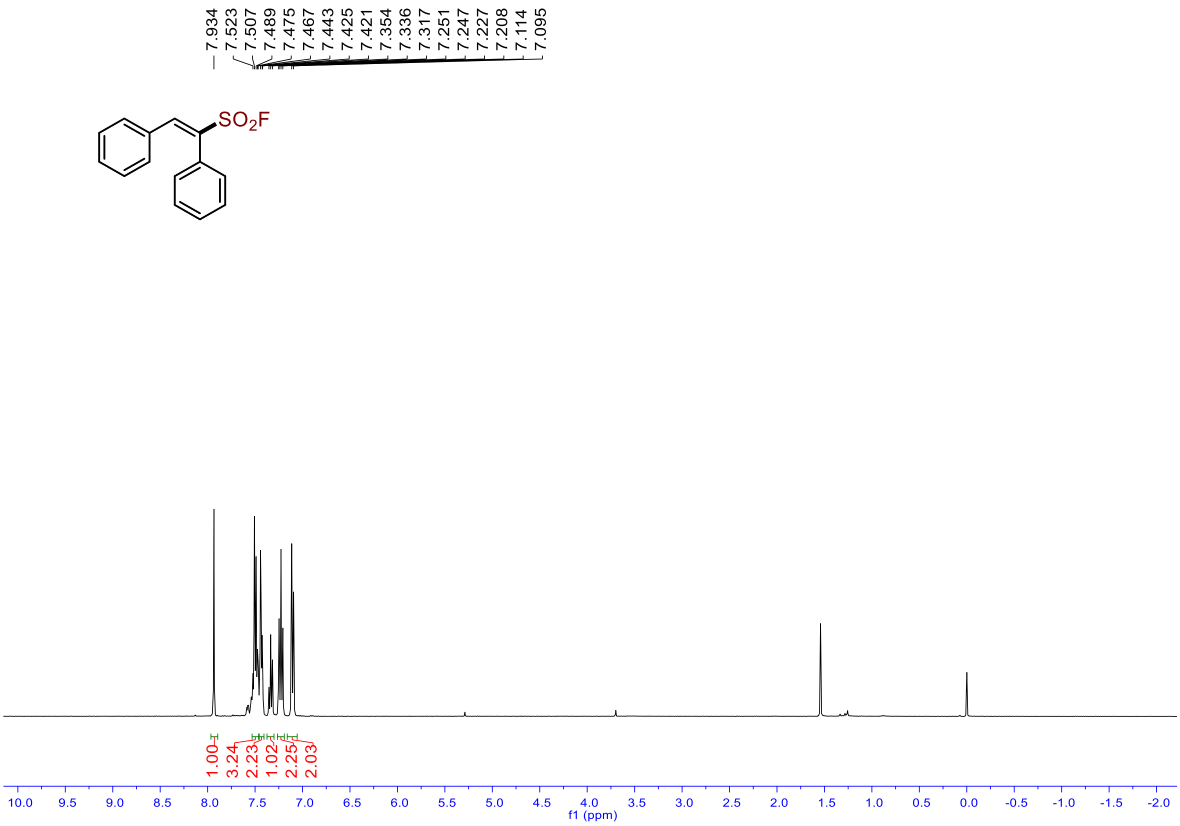

Supplementary Figure 107. ^{1}H NMR spectra of **5ad**

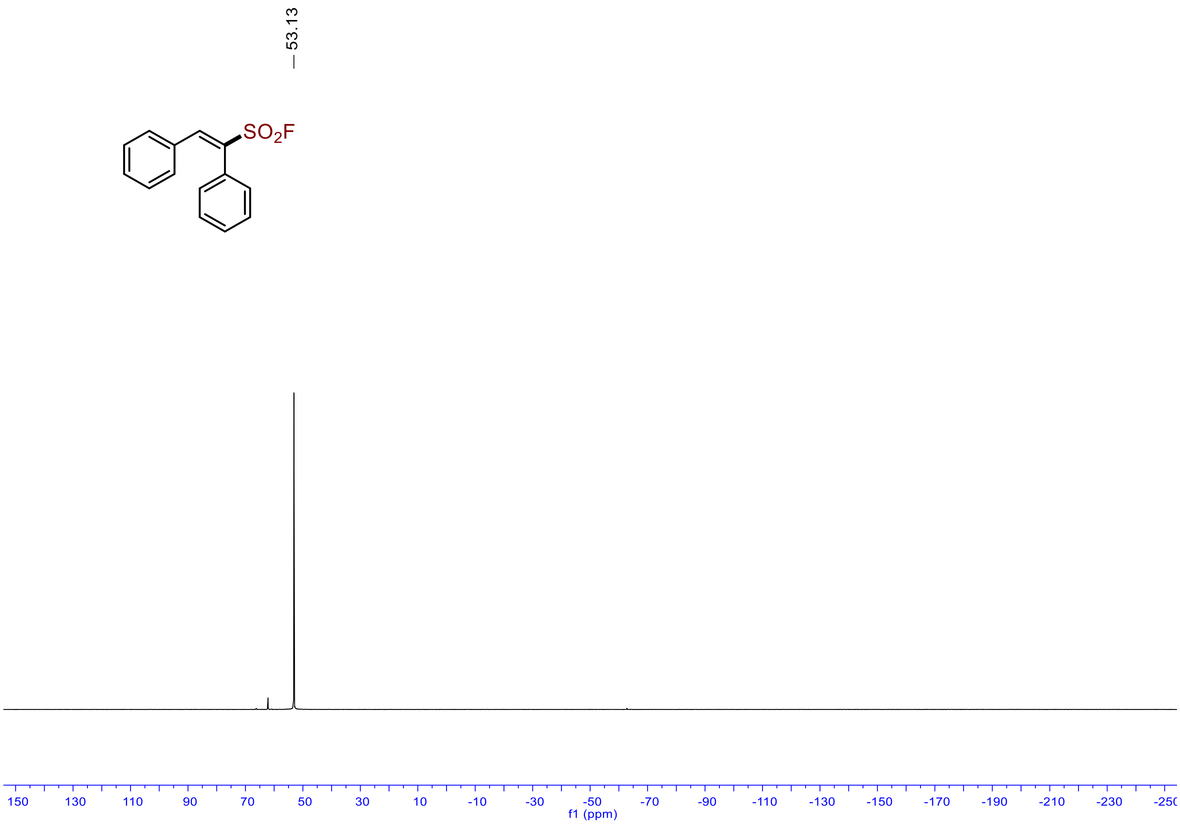

Supplementary Figure 108. ^{19}F NMR spectra of **5ad**

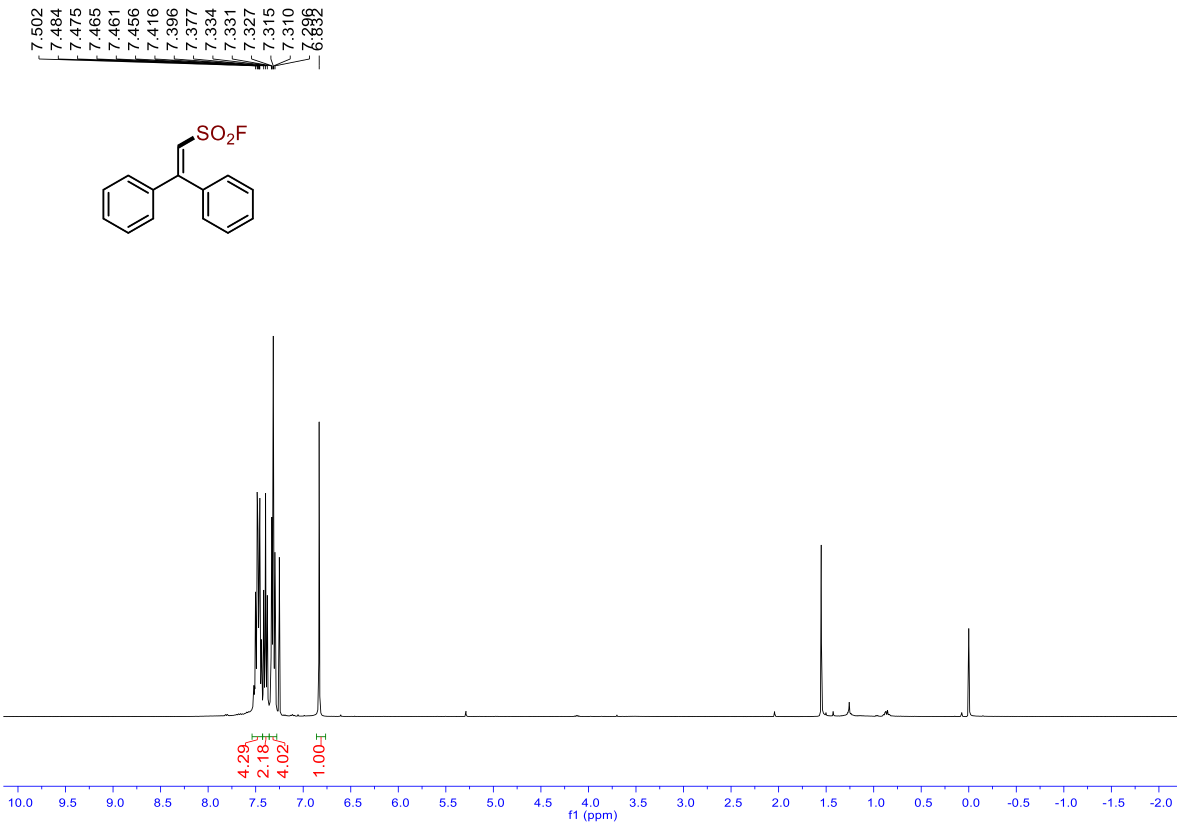

Supplementary Figure 109. ^{1}H NMR spectra of **5ae**

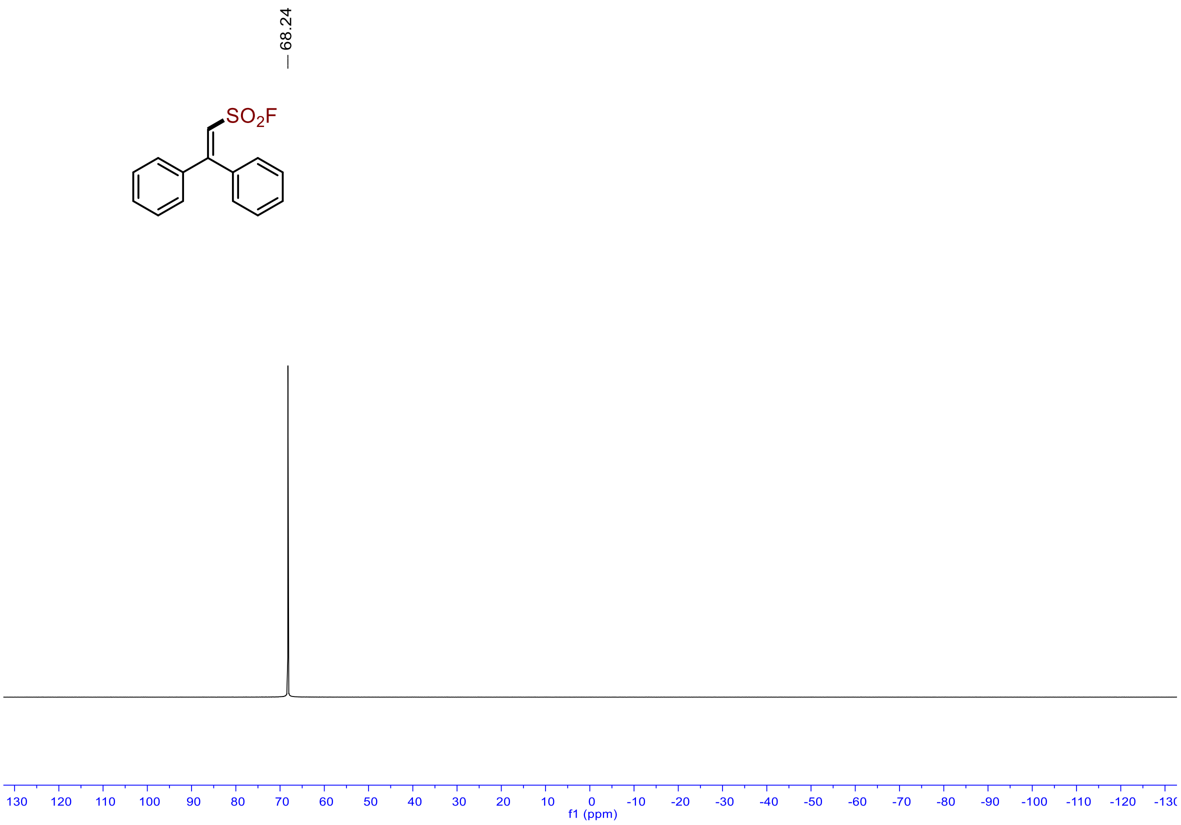

Supplementary Figure 110. ^{19}F NMR spectra of **5ae**

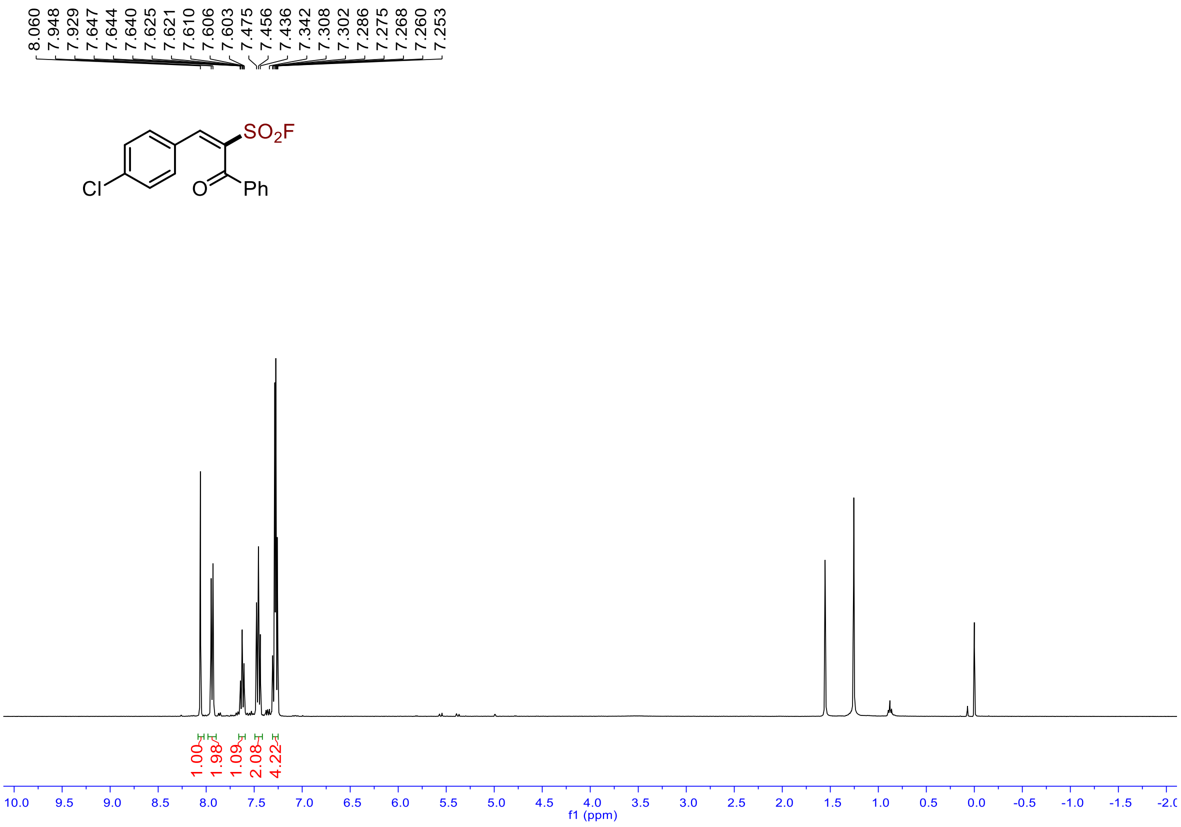

Supplementary Figure 111. ^{1}H NMR spectra of **5af**

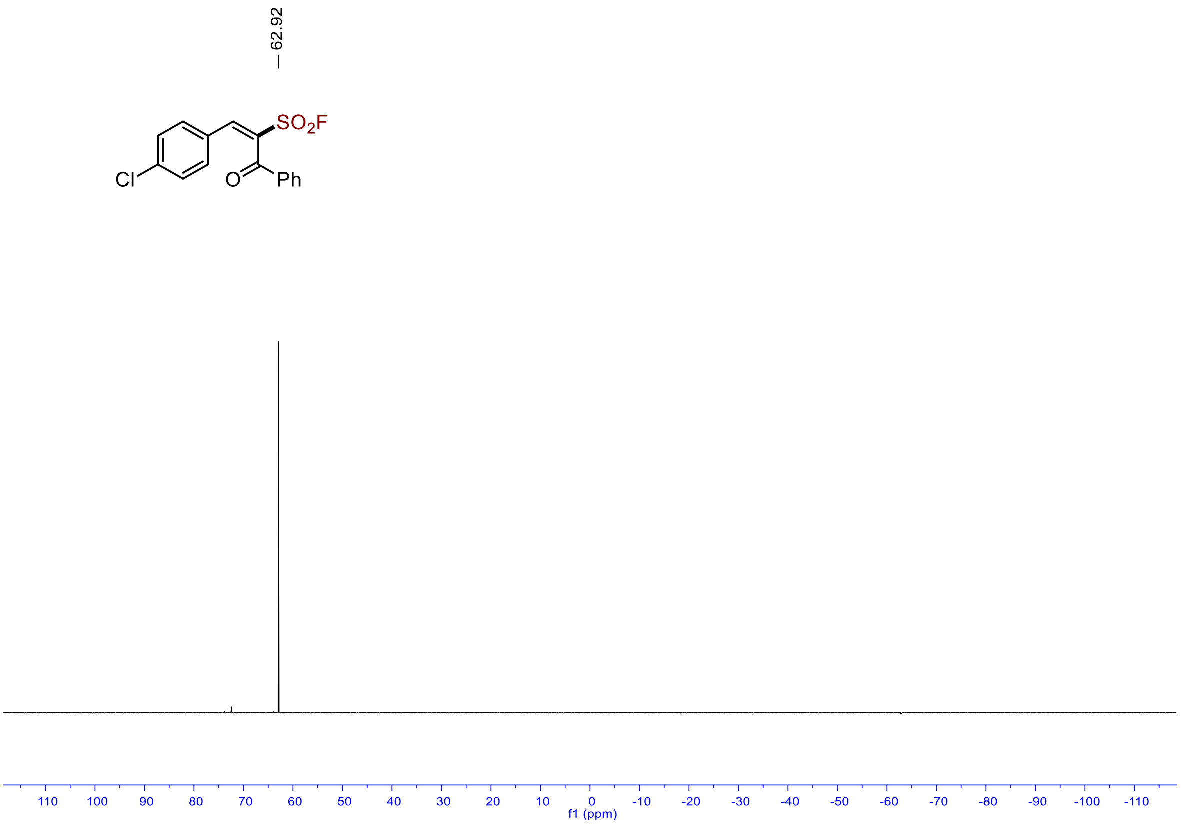

Supplementary Figure 112. ^{19}F NMR spectra of **5af**

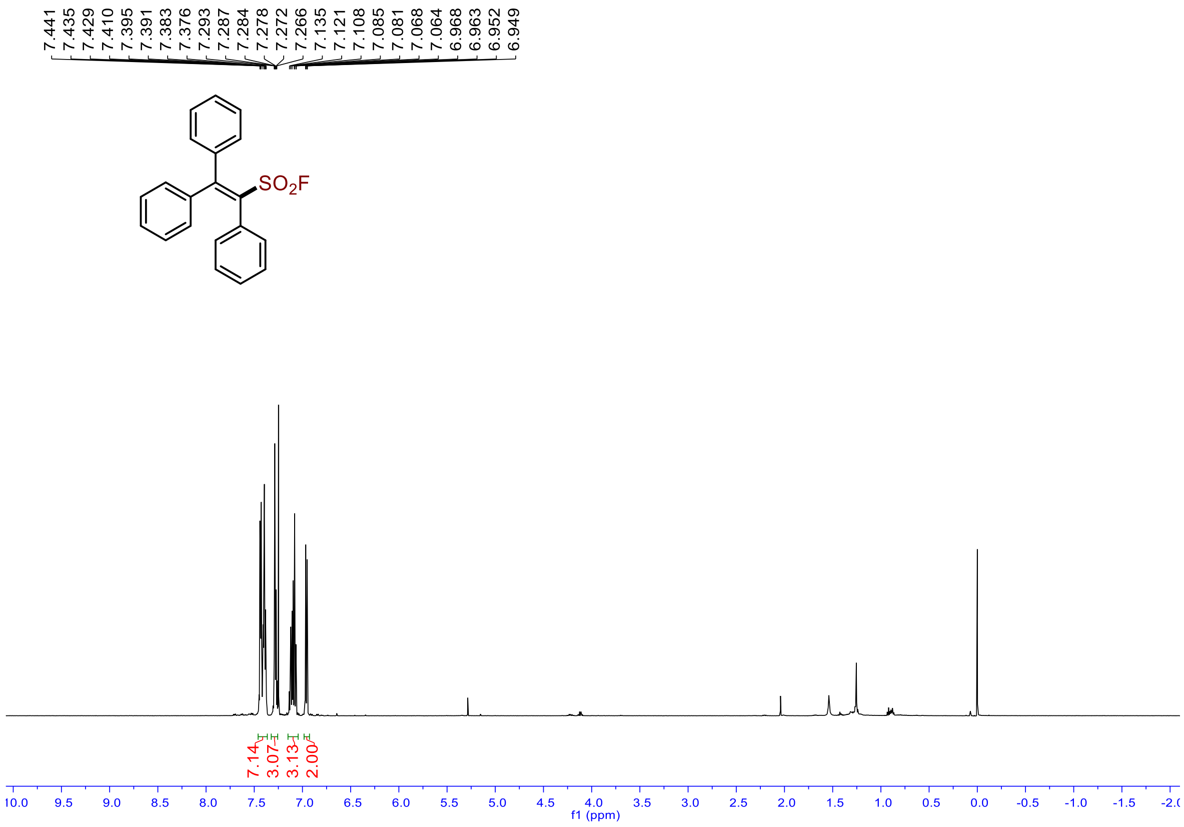

Supplementary Figure 113. ^{1}H NMR spectra of **5ag**

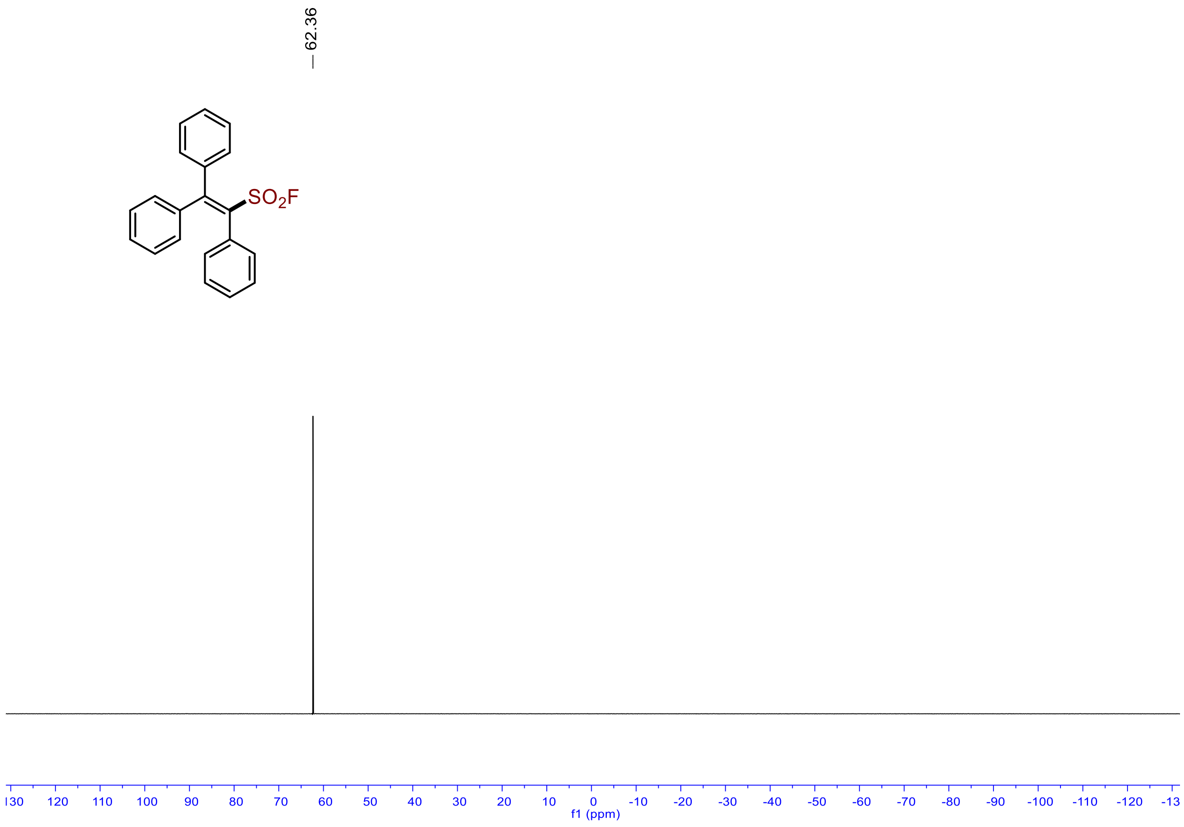

Supplementary Figure 114. ^{19}F NMR spectra of **5ag**

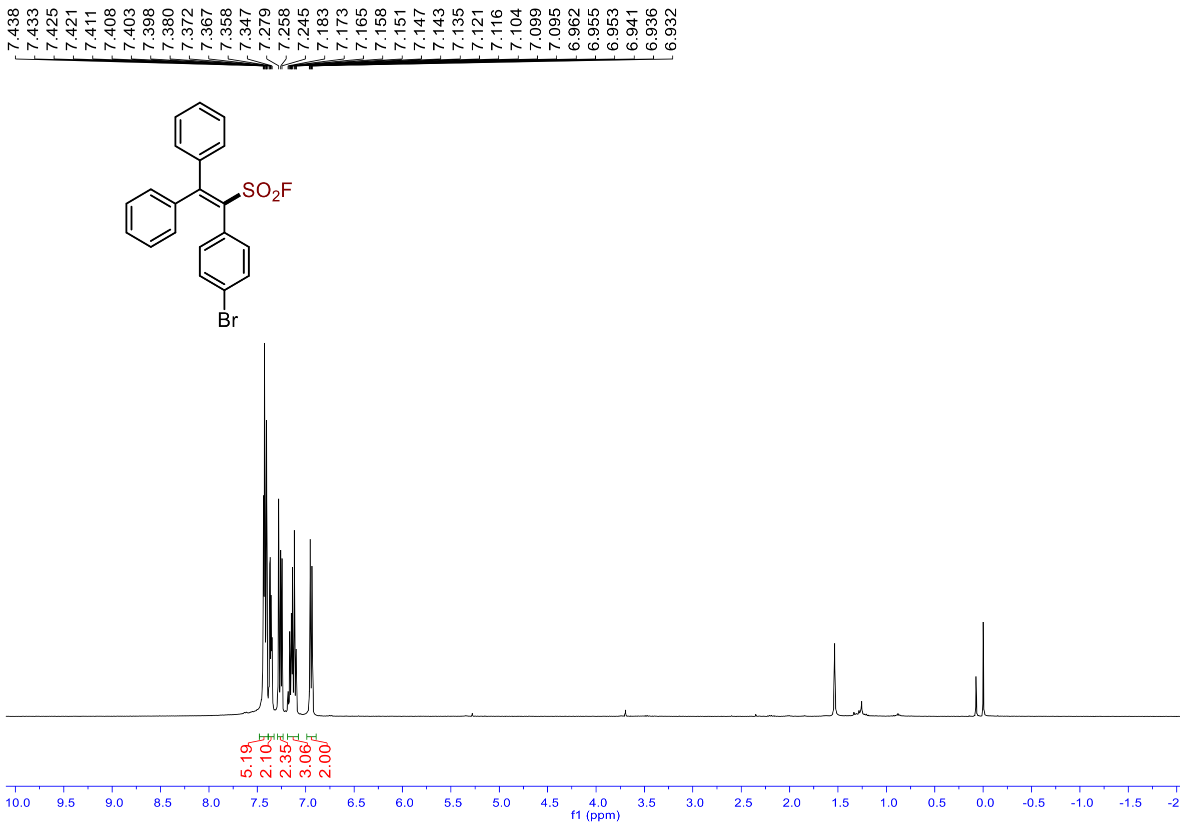

Supplementary Figure 115. ^{1}H NMR spectra of **5ah**

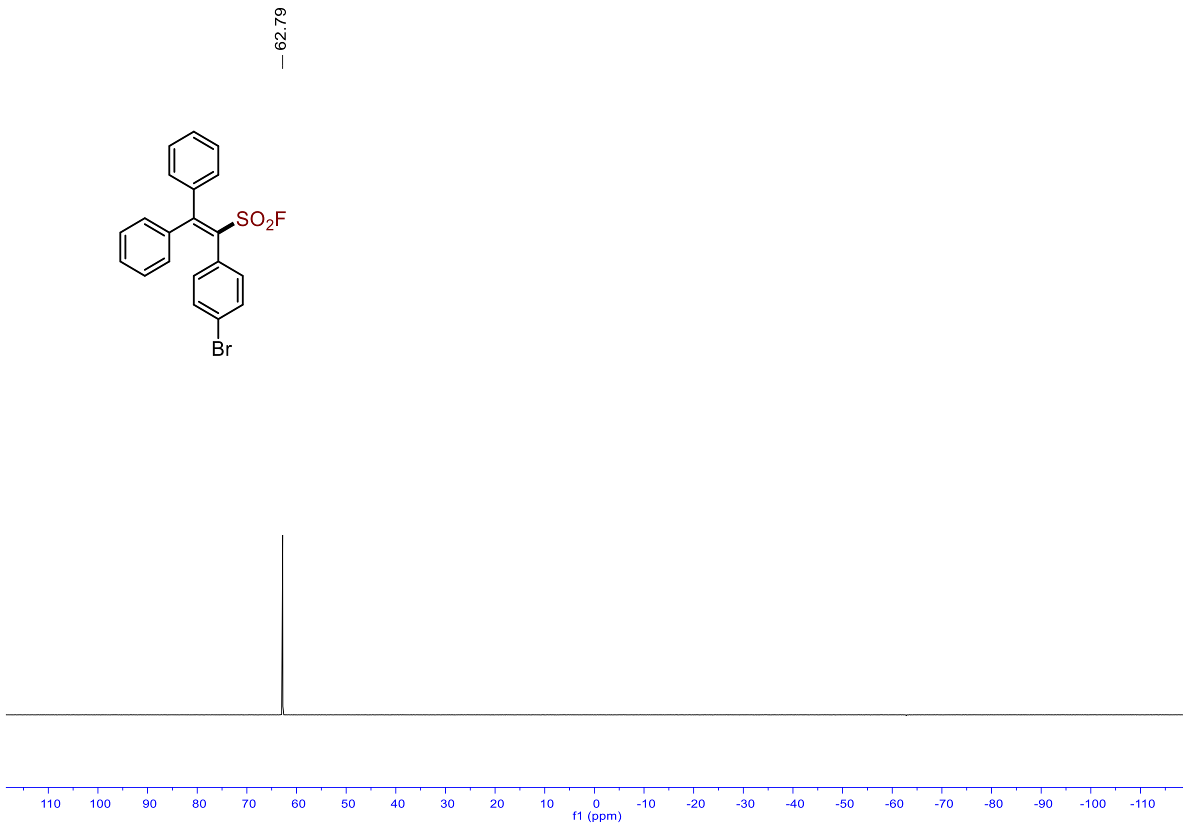

Supplementary Figure 116. ^{19}F NMR spectra of **5ah**

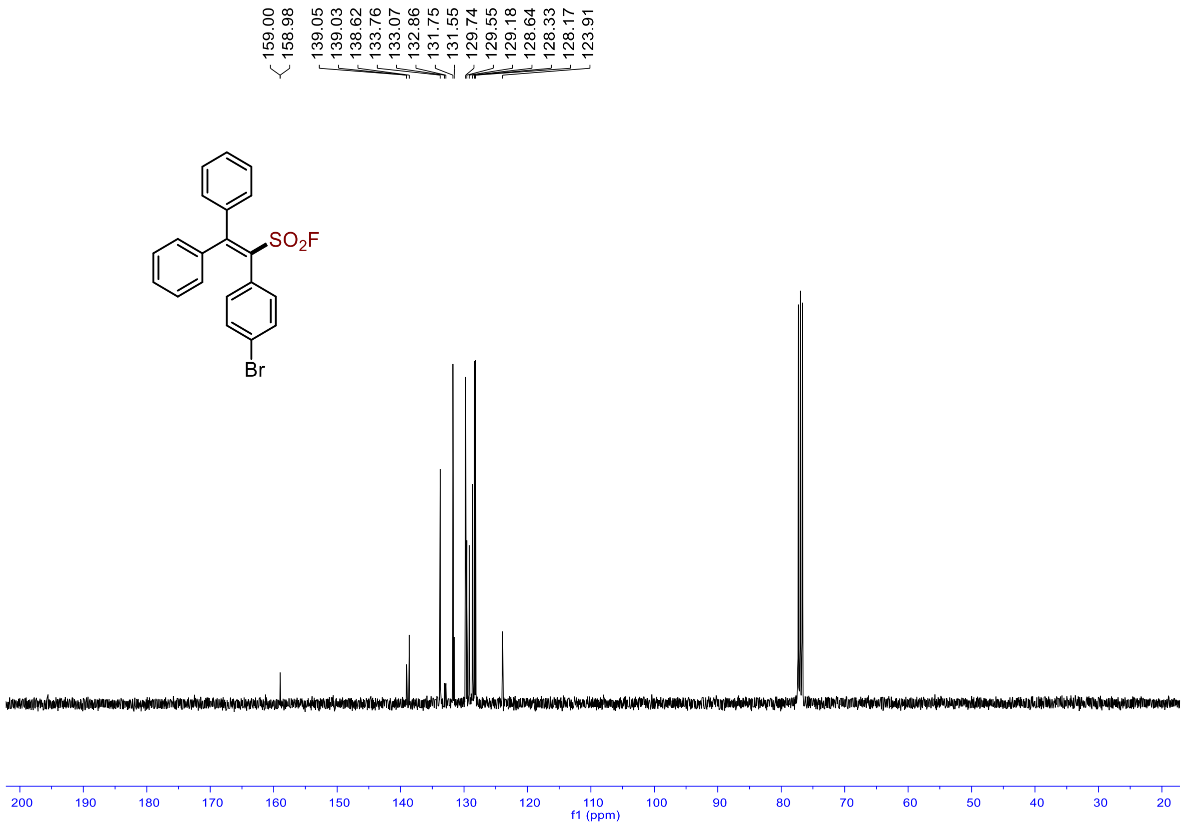

Supplementary Figure 117. ^{13}C NMR spectra of **5ah**

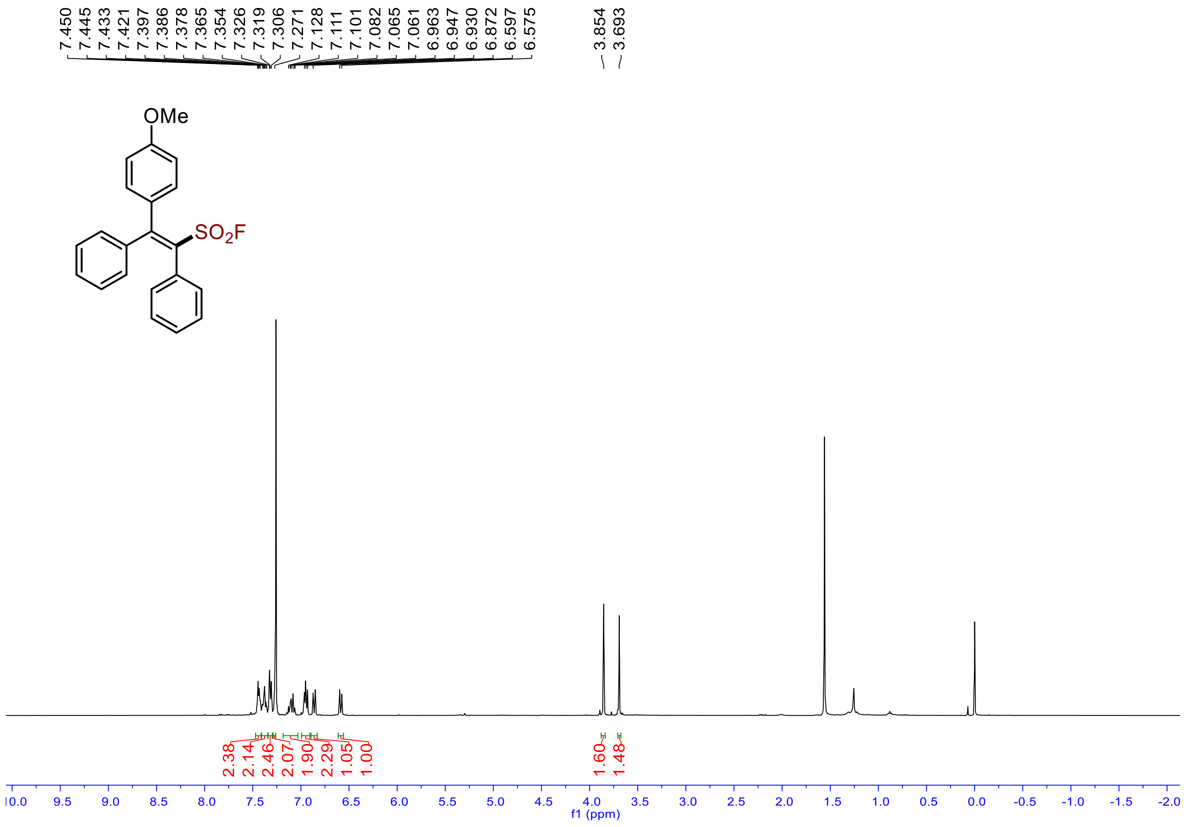

Supplementary Figure 118. ^{1}H NMR spectra of **5ai**

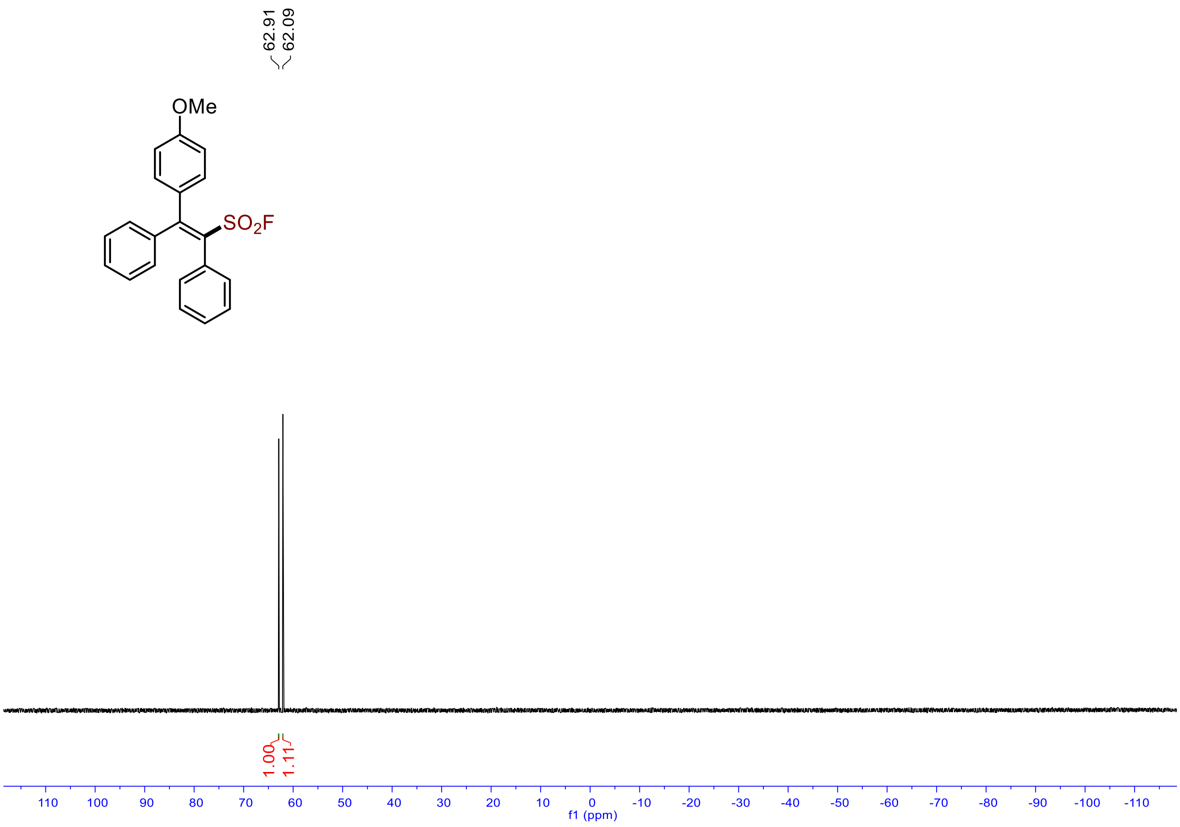

Supplementary Figure 119. ^{19}F NMR spectra of **5ai**

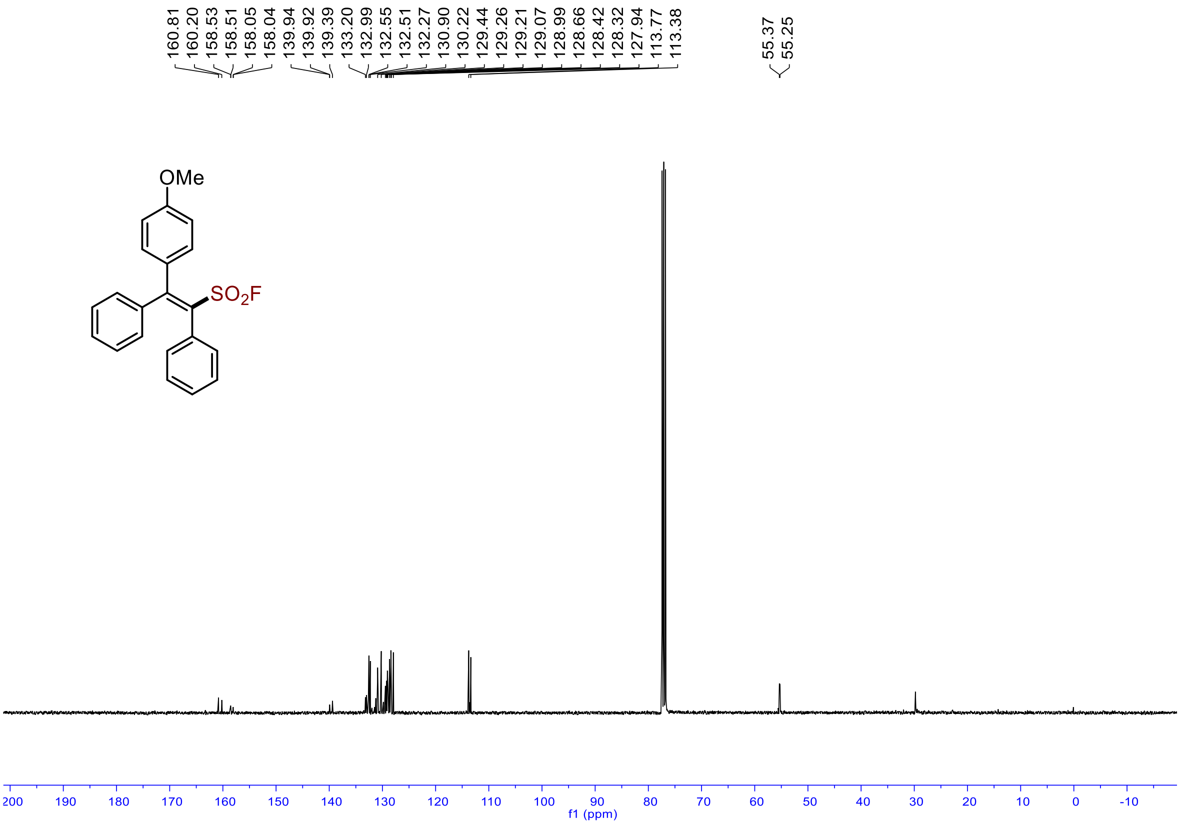

Supplementary Figure 120. ^{13}C NMR spectra of **5ai**

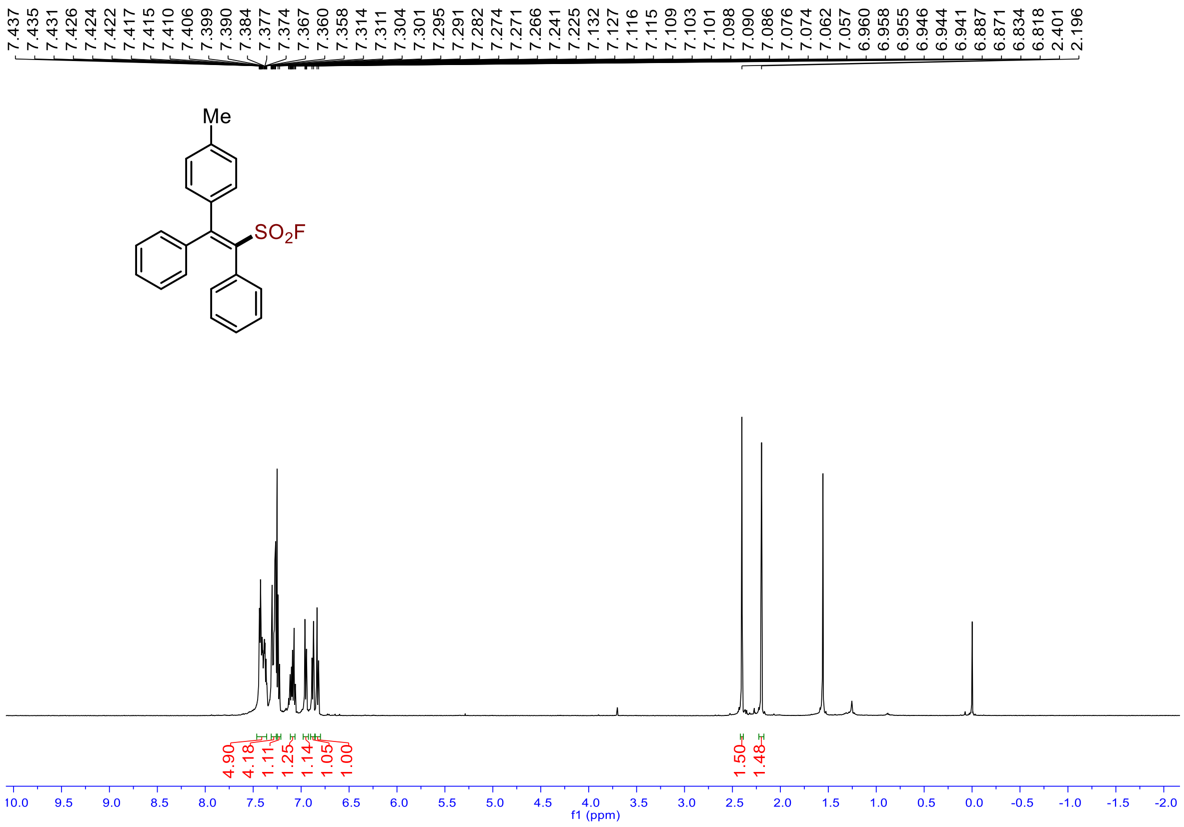

Supplementary Figure 121. ^{1}H NMR spectra of **5aj**

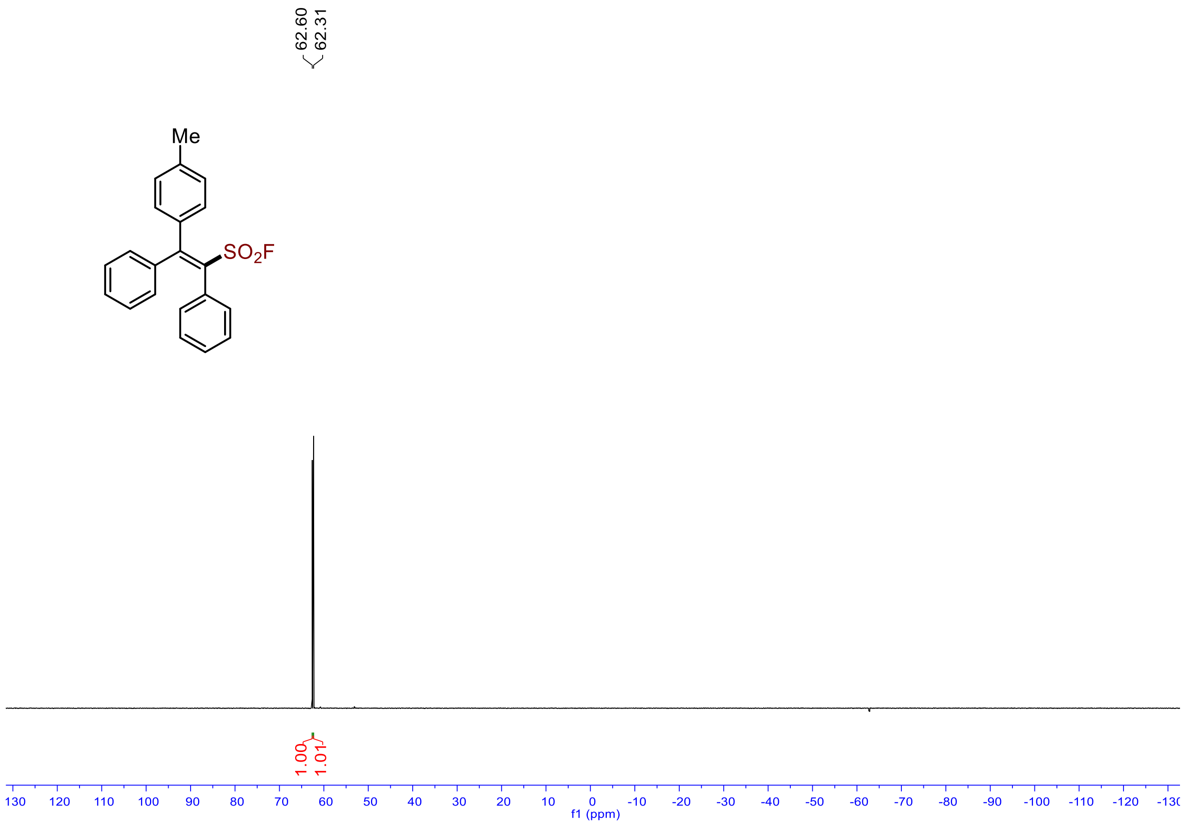

Supplementary Figure 122. ^{19}F NMR spectra of **5aj**

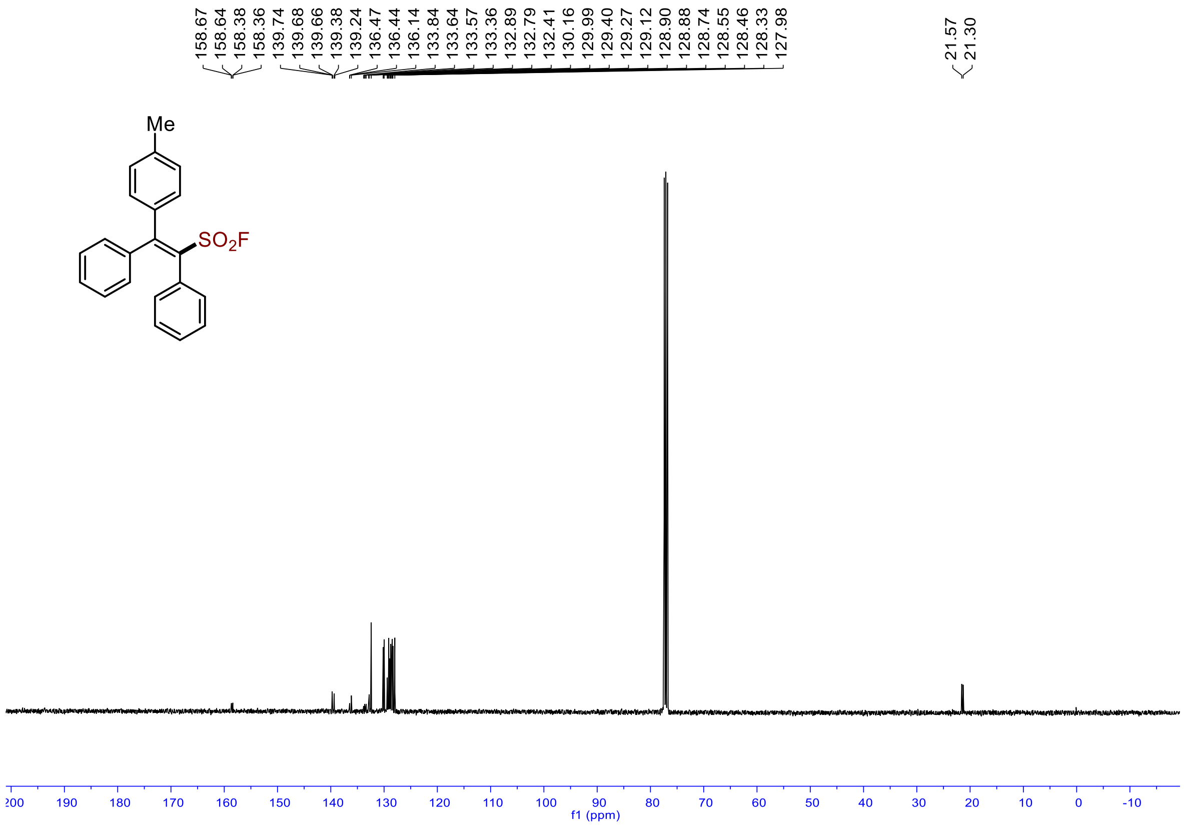

Supplementary Figure 123. ^{13}C NMR spectra of **5aj**

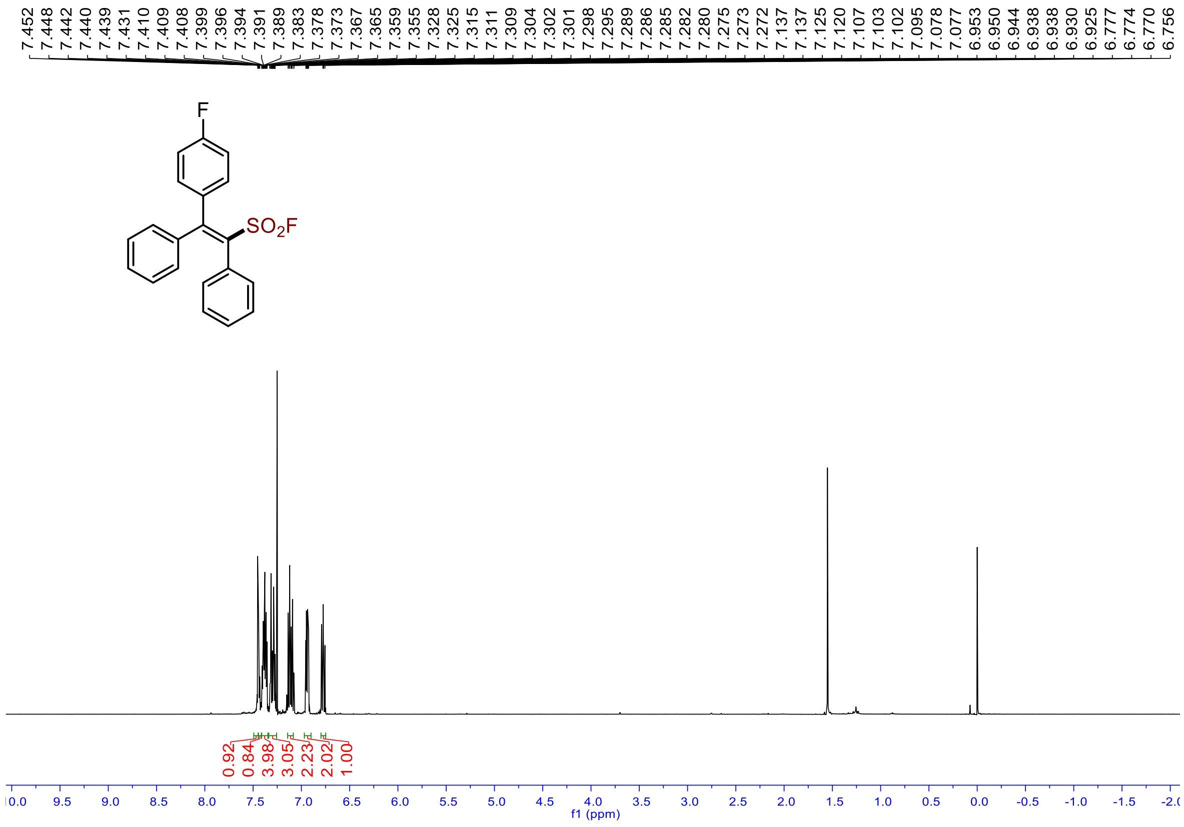

Supplementary Figure 124. ^{1}H NMR spectra of **5ak**

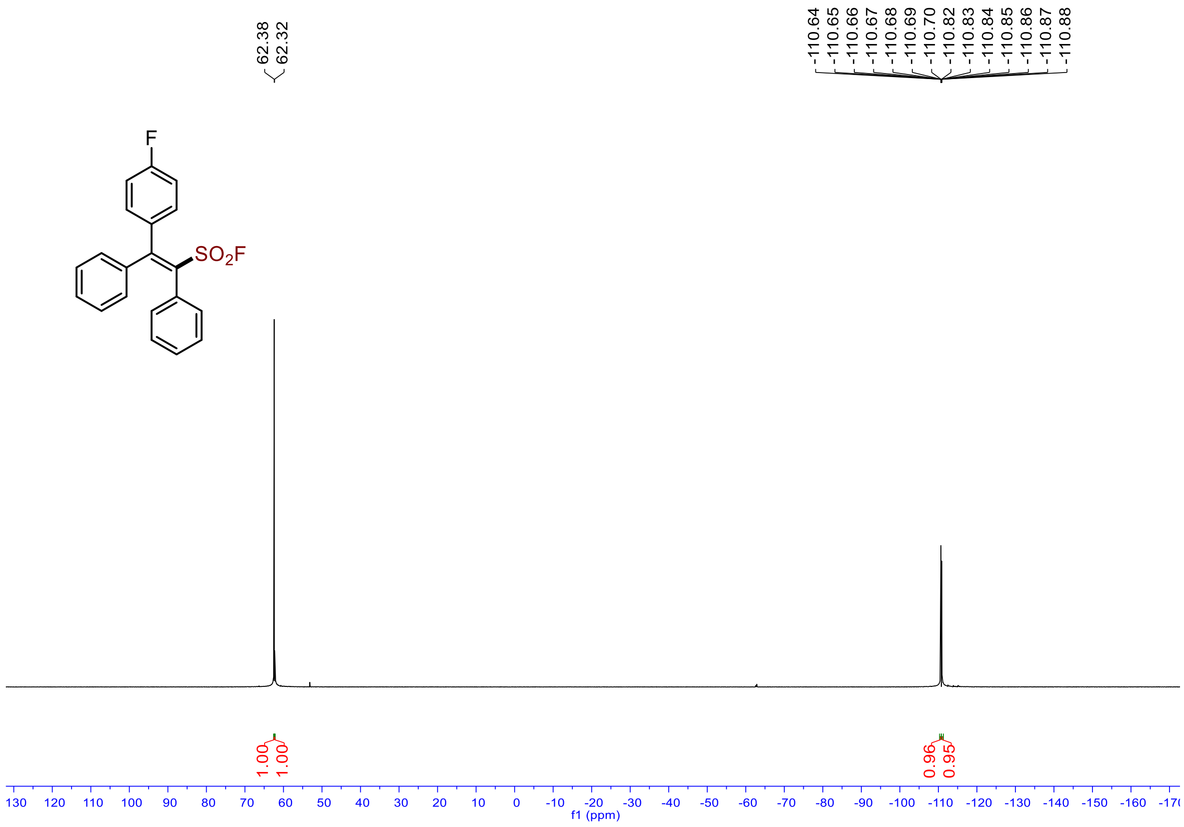

Supplementary Figure 125. ^{19}F NMR spectra of **5ak**

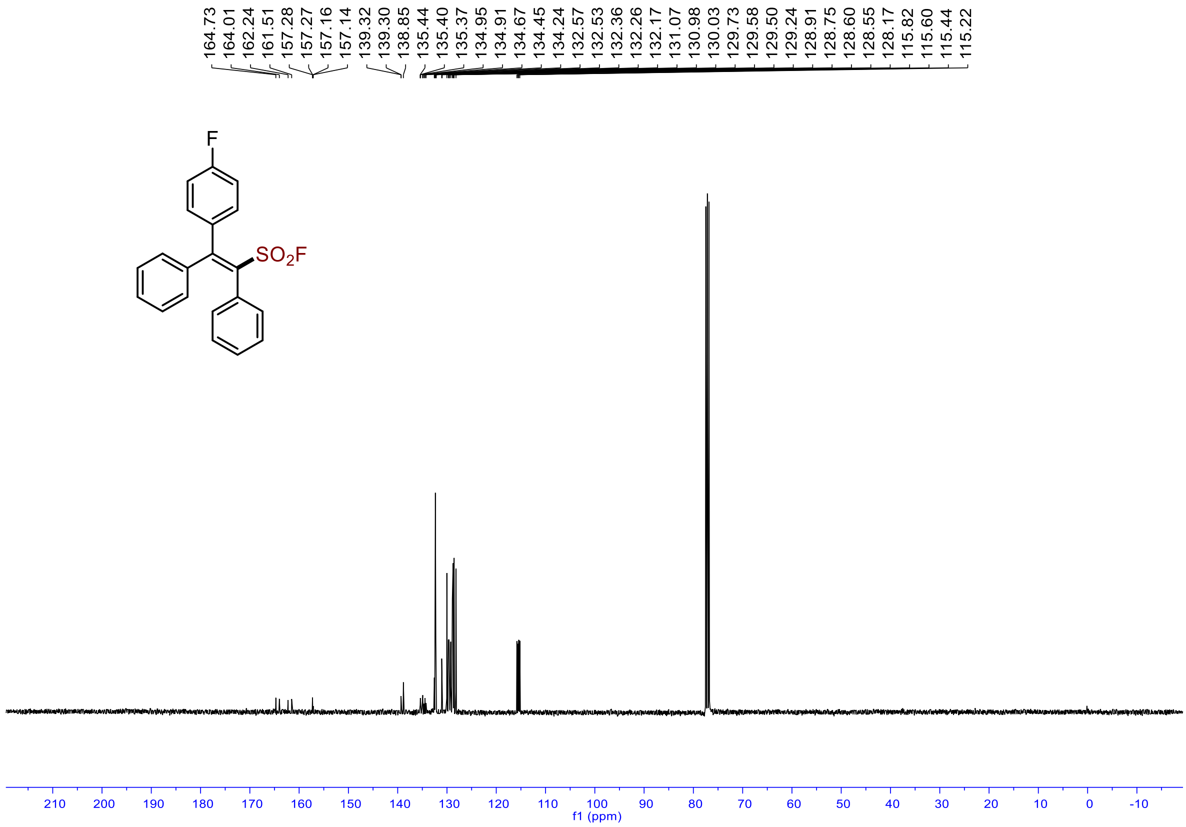

Supplementary Figure 126. ^{13}C NMR spectra of **5ak**

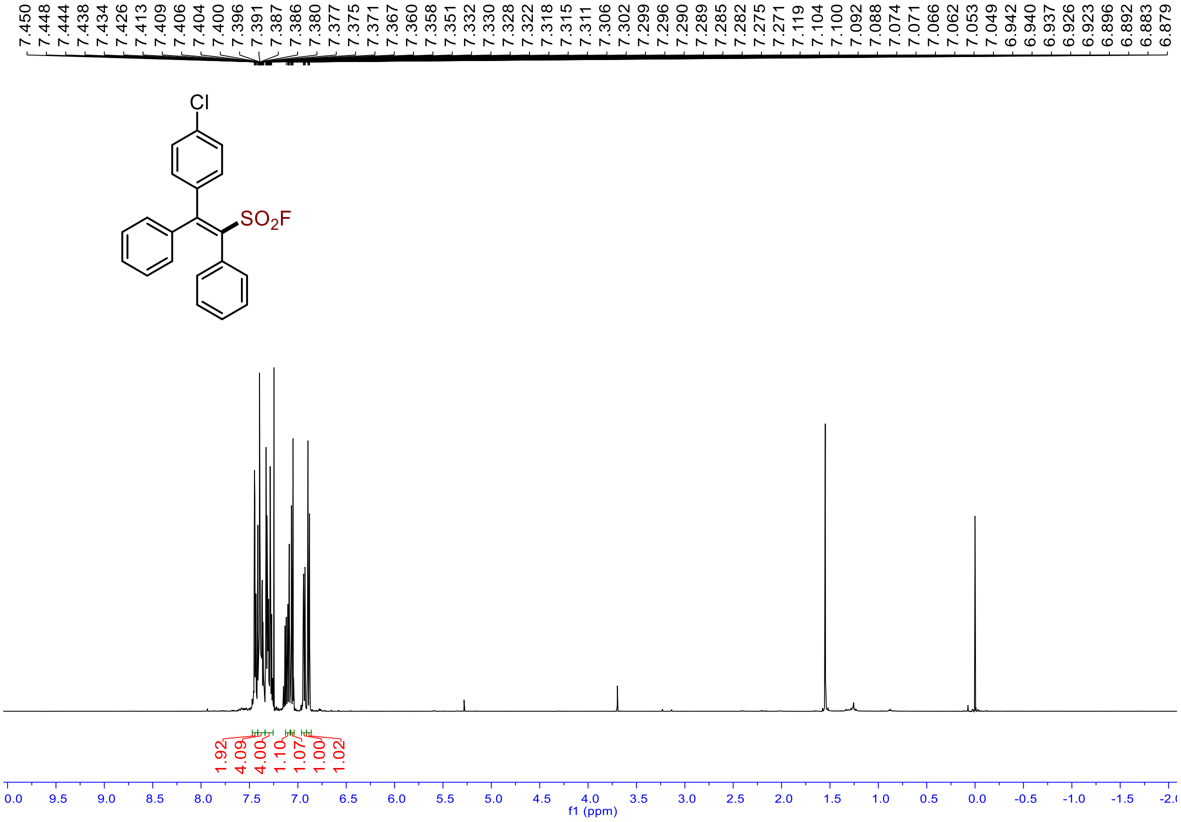

Supplementary Figure 127. ^{1}H NMR spectra of **5al**

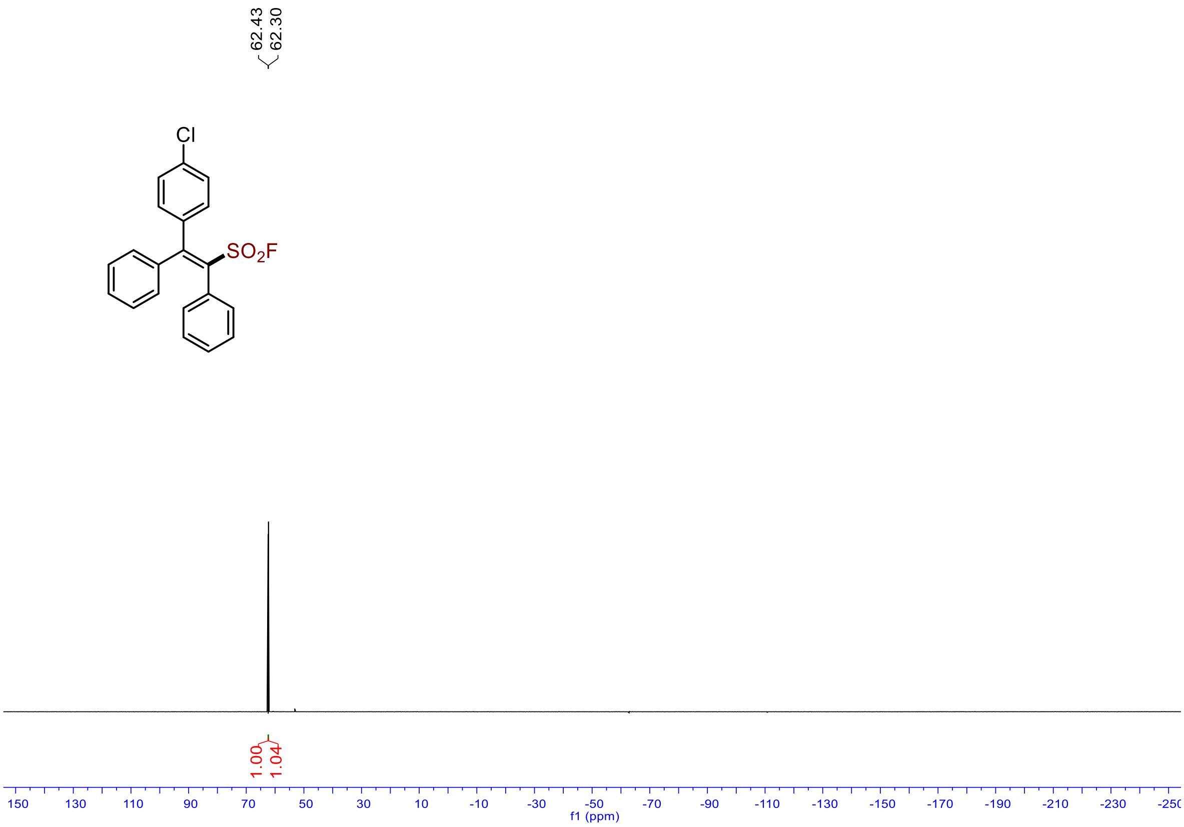

Supplementary Figure 128. ^{19}F NMR spectra of **5al**

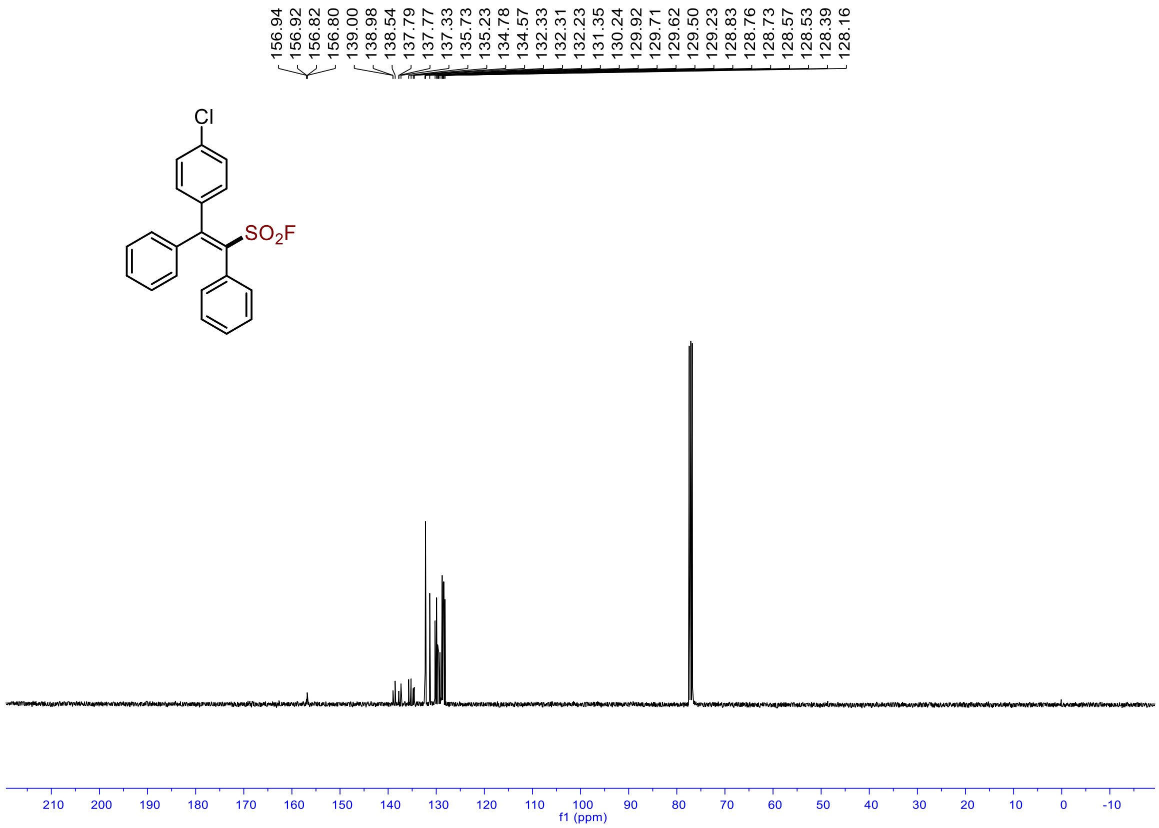

Supplementary Figure 129. ^{13}C NMR spectra of **5al**

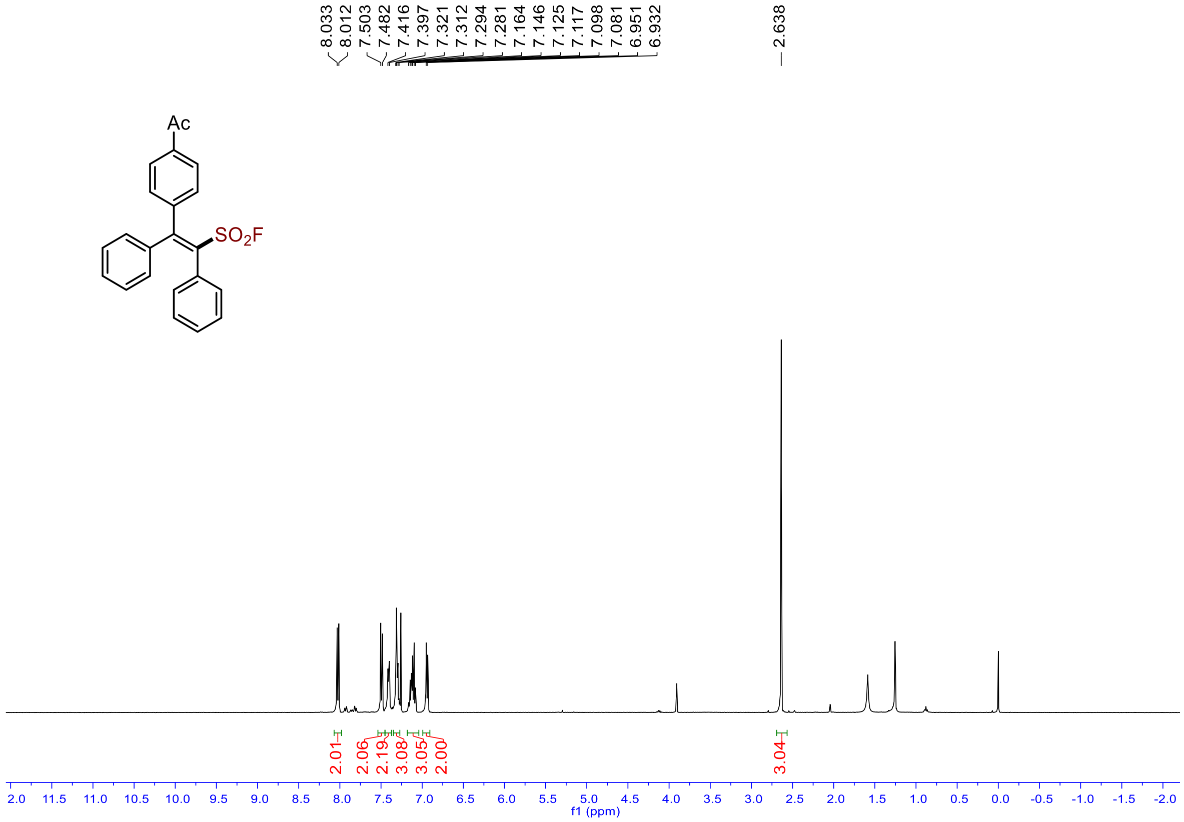

Supplementary Figure 130. ^{1}H NMR spectra of **5am**

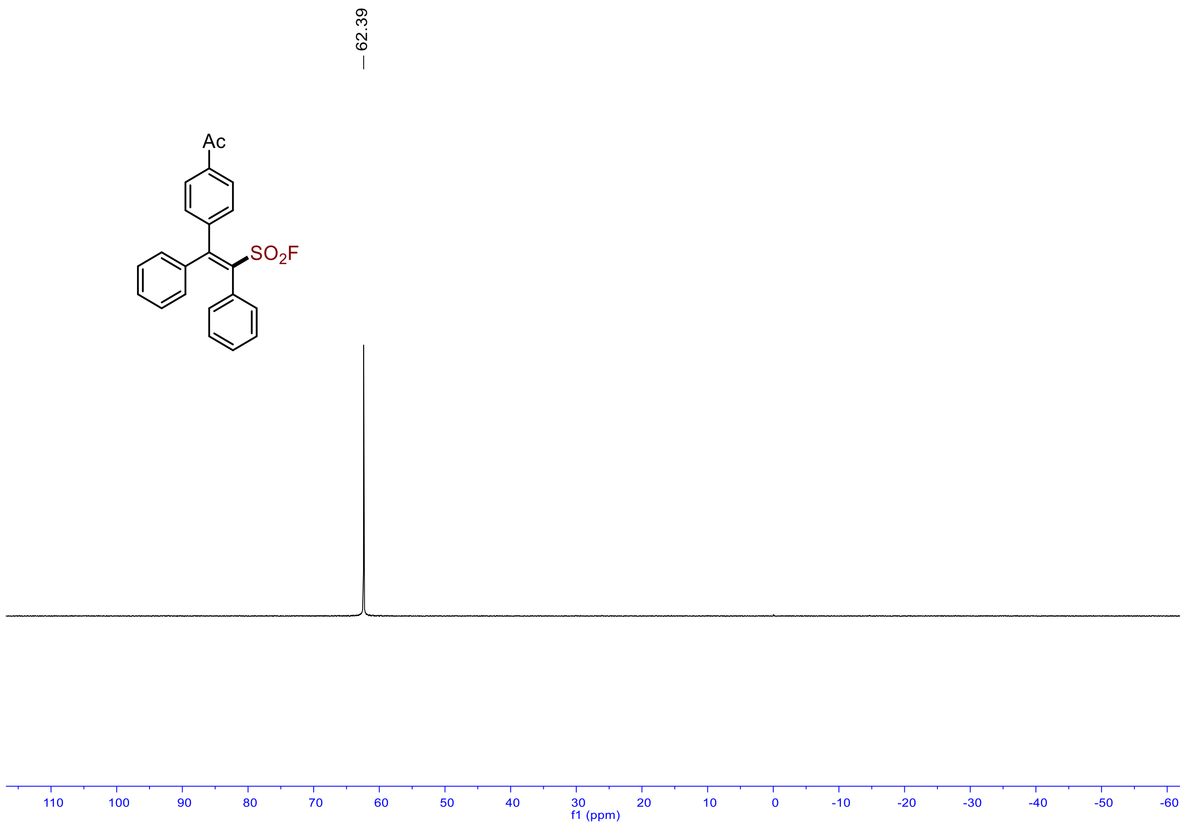

Supplementary Figure 131. ^{19}F NMR spectra of **5am**

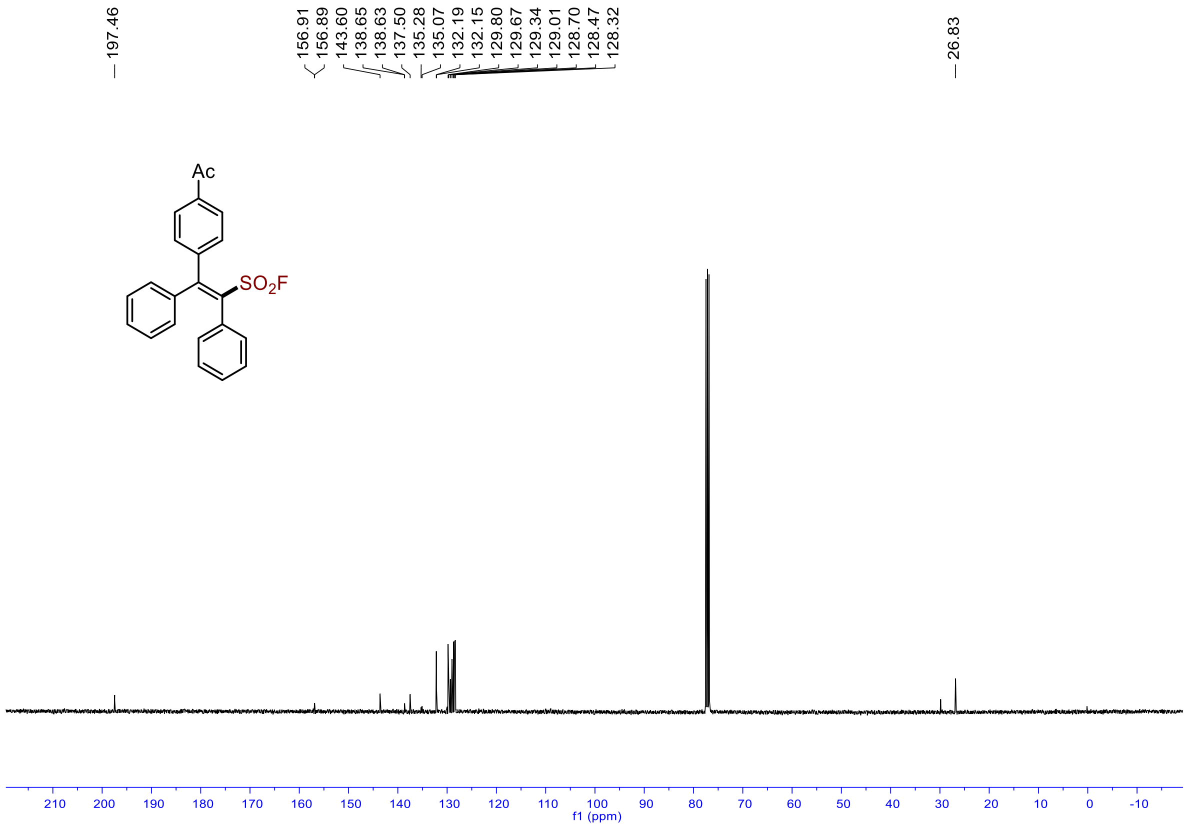

Supplementary Figure 132. ^{13}C NMR spectra of **5am**

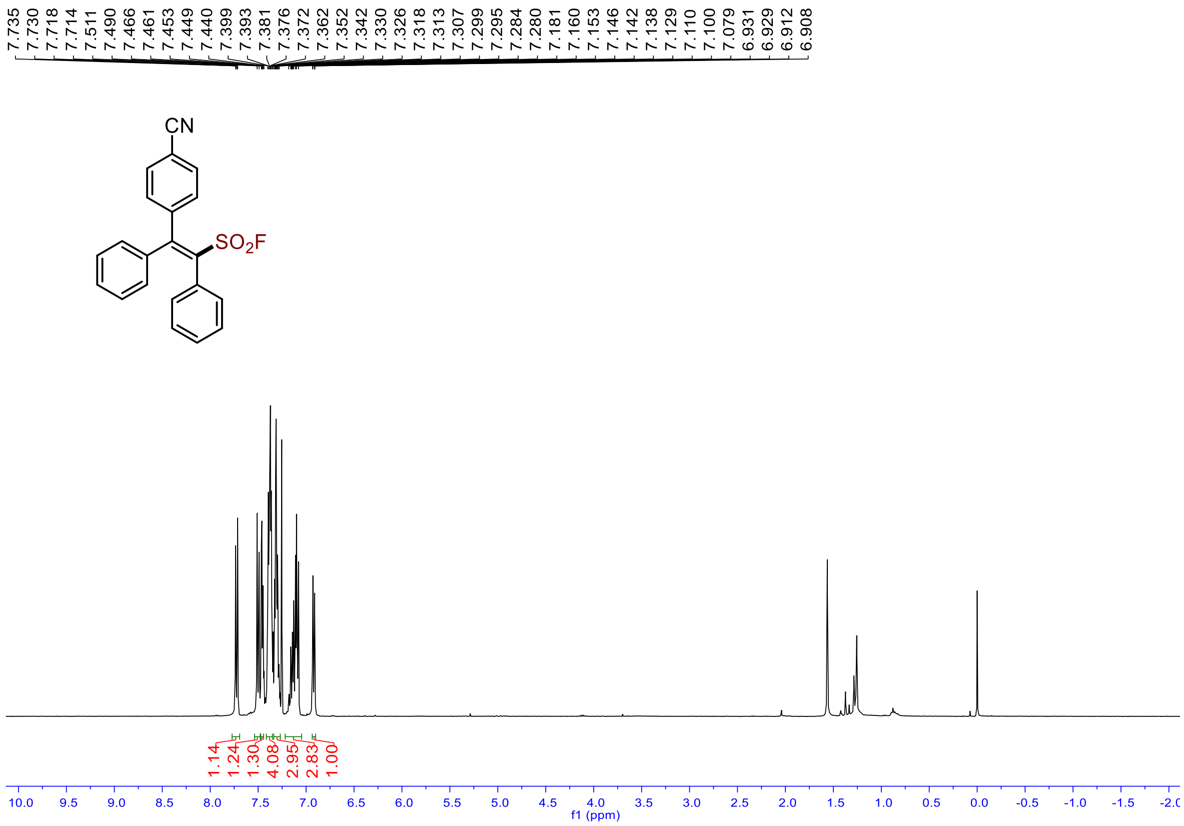

Supplementary Figure 133. ^{1}H NMR spectra of **5an**

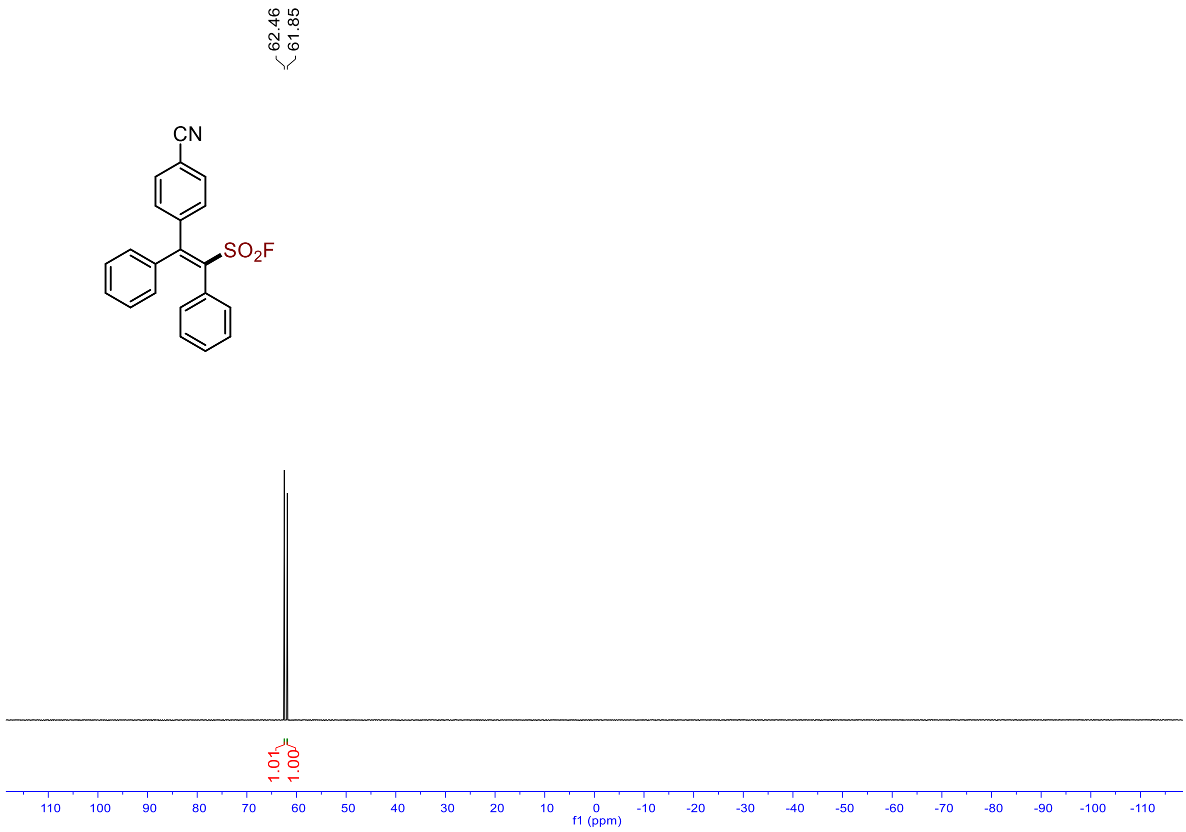

Supplementary Figure 134. ^{19}F NMR spectra of **5an**

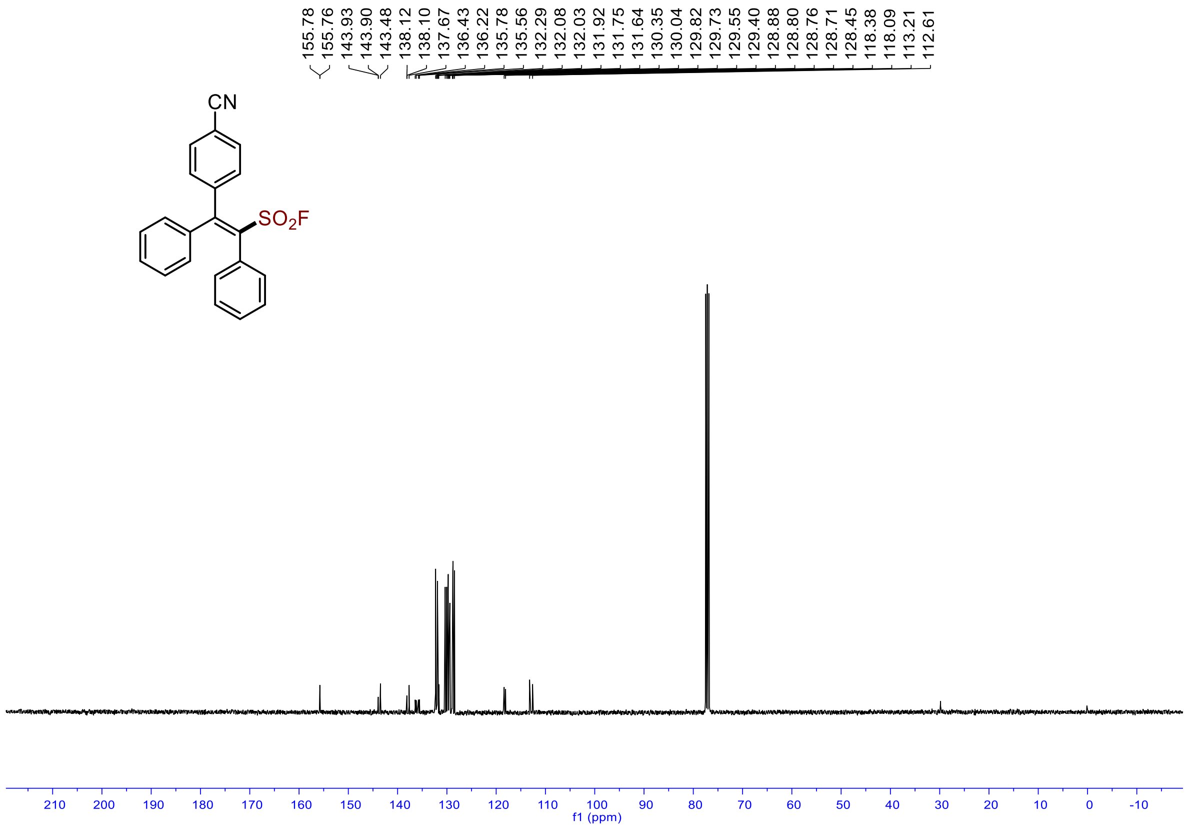

Supplementary Figure 135. ^{13}C NMR spectra of **5an**

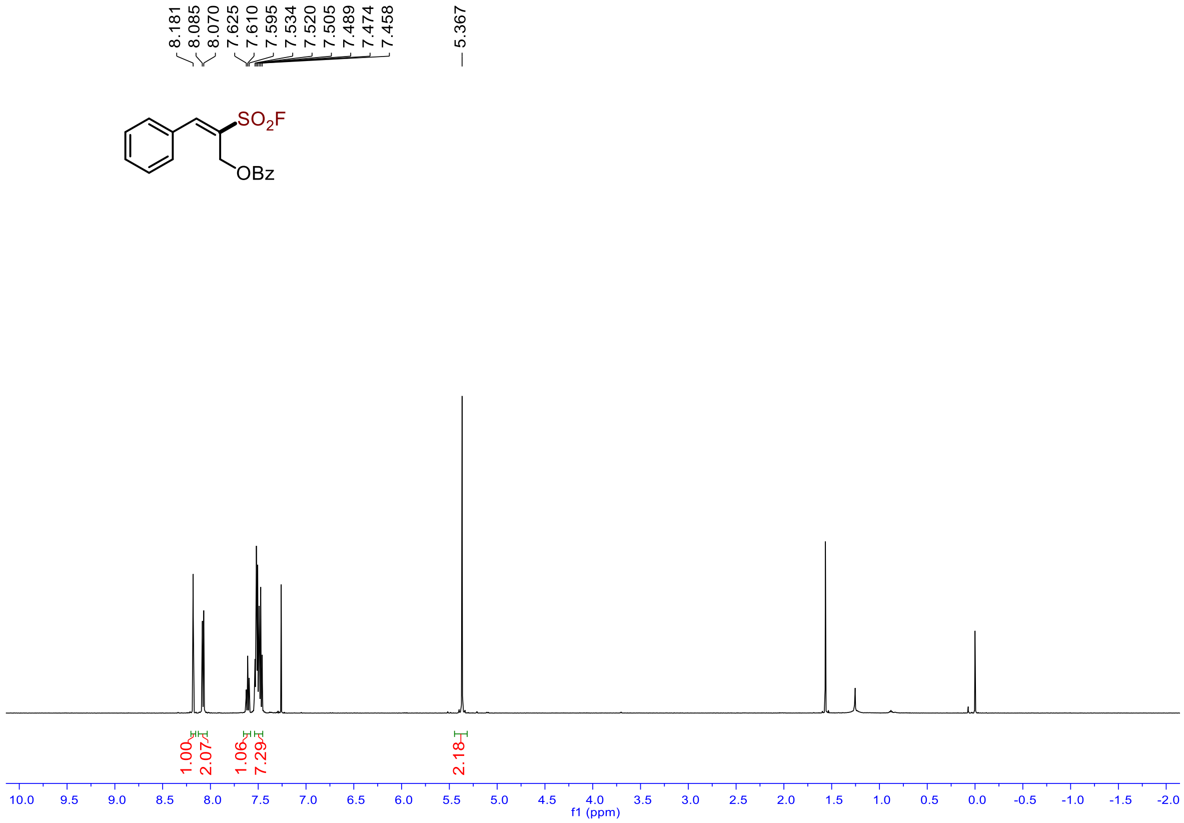

Supplementary Figure 136. ^{1}H NMR spectra of **5ao**

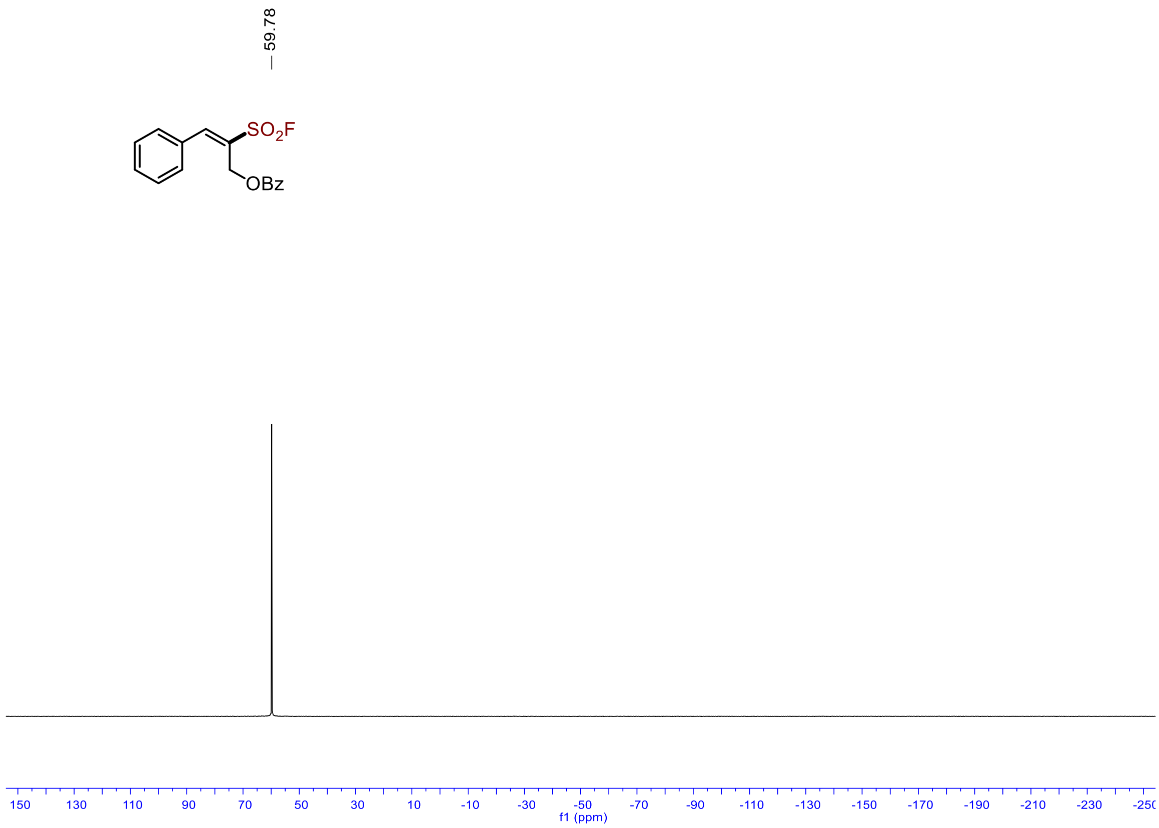

Supplementary Figure 137. ^{19}F NMR spectra of **5ao**

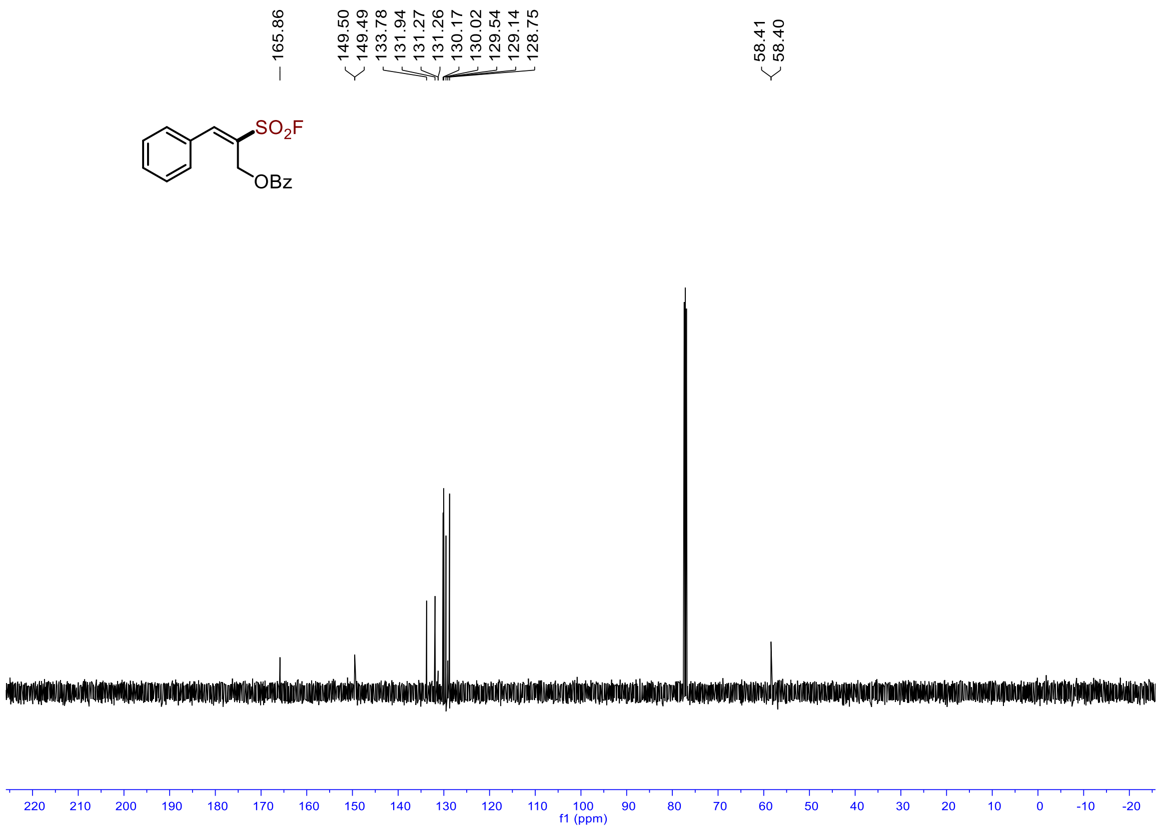

Supplementary Figure 138. ^{13}C NMR spectra of **5ao**

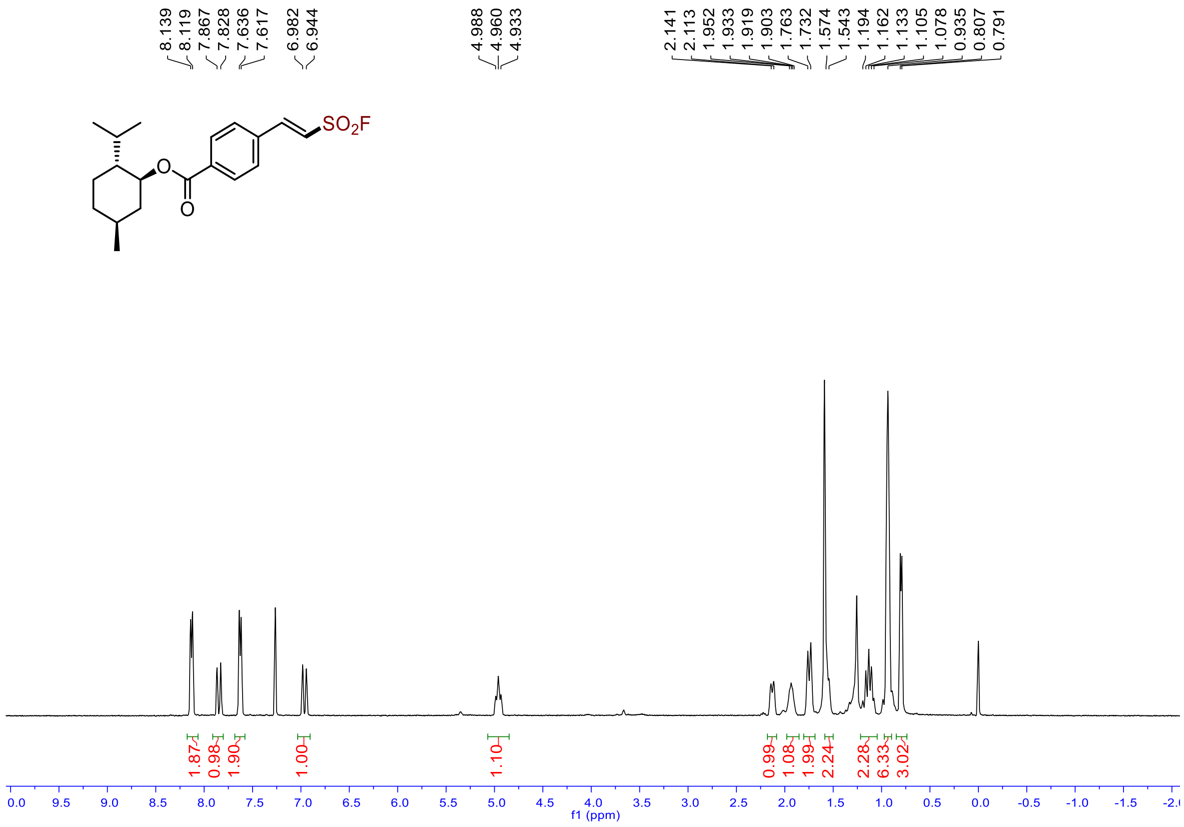

Supplementary Figure 139. ^{1}H NMR spectra of **5ap**

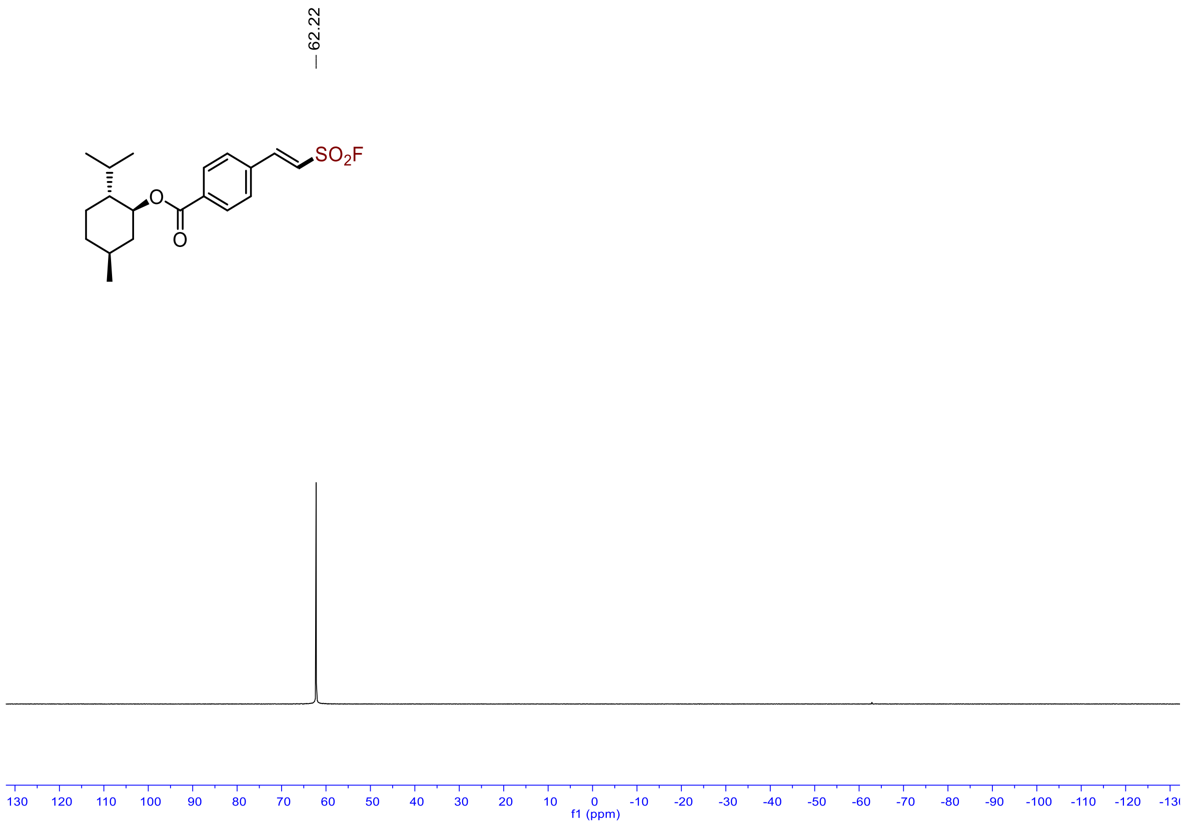

Supplementary Figure 140. ^{19}F NMR spectra of **5ap**

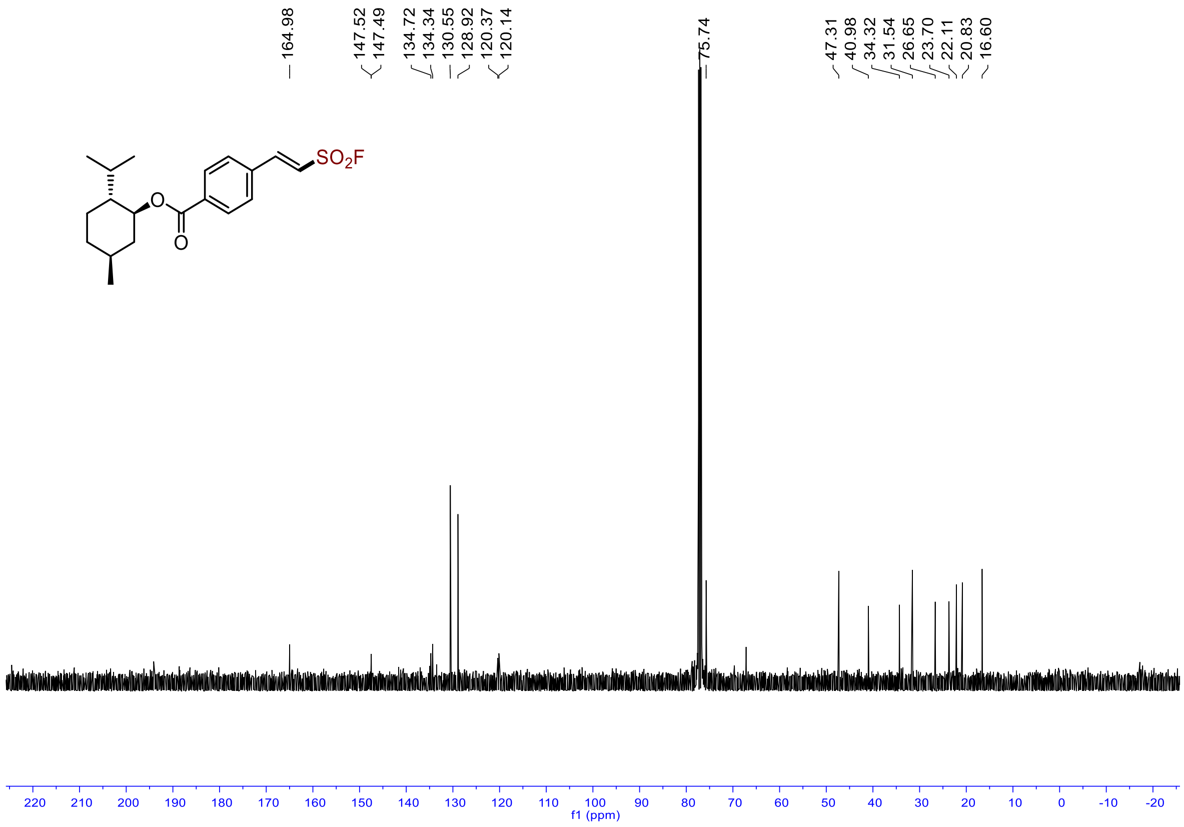

Supplementary Figure 141. ^{13}C NMR spectra of **5ap**

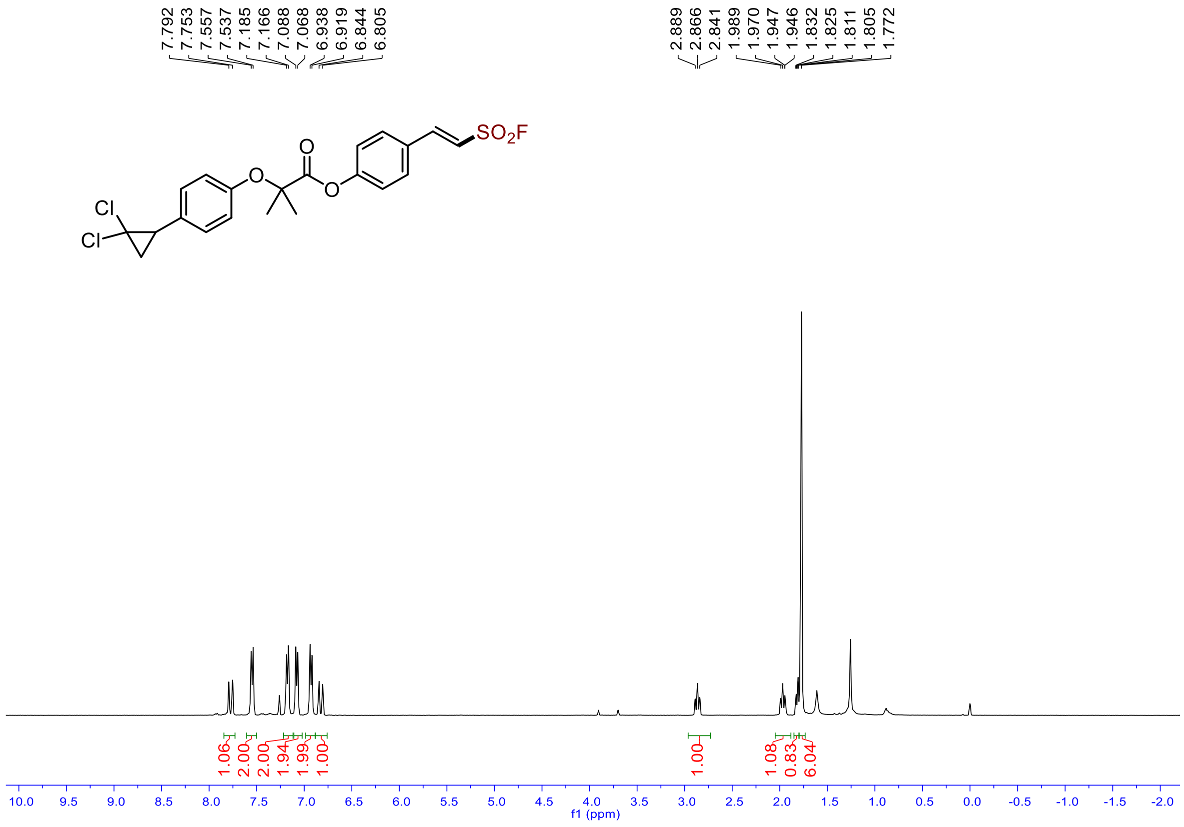

Supplementary Figure 142. ^{1}H NMR spectra of **5aq**

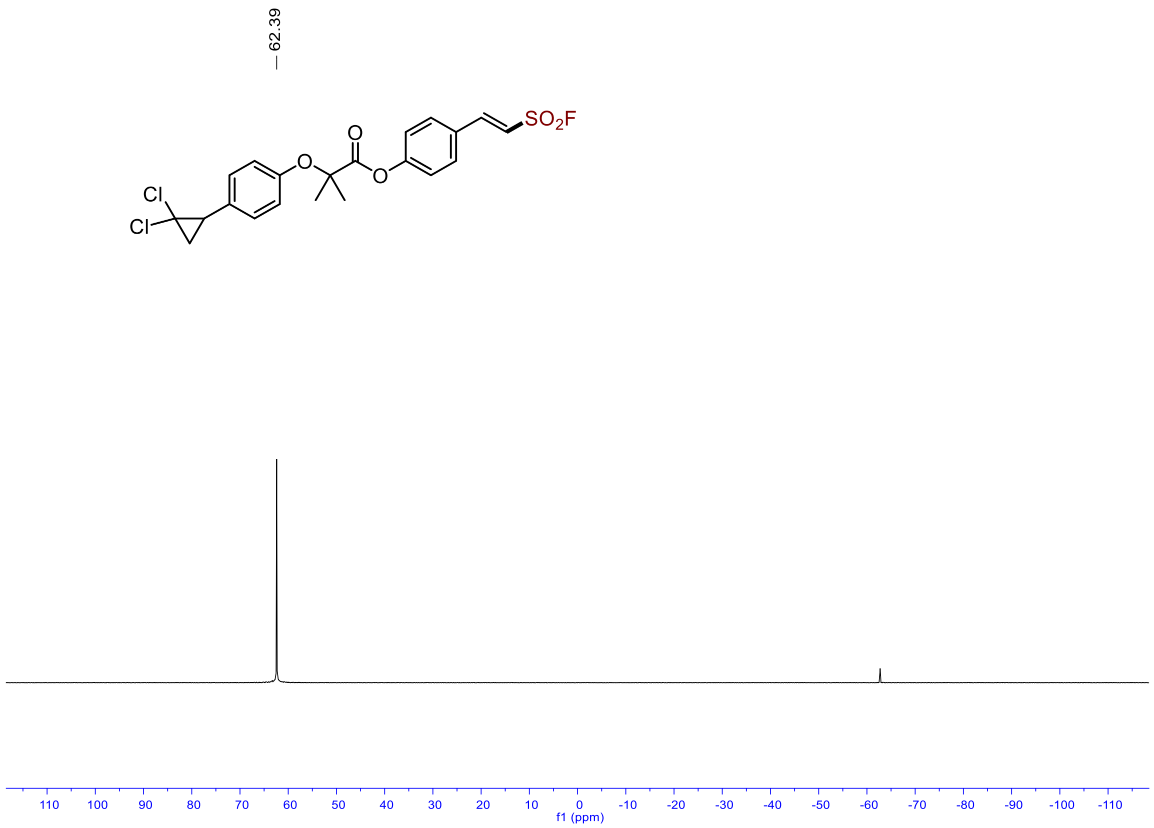

Supplementary Figure 143. ^{19}F NMR spectra of **5aq**

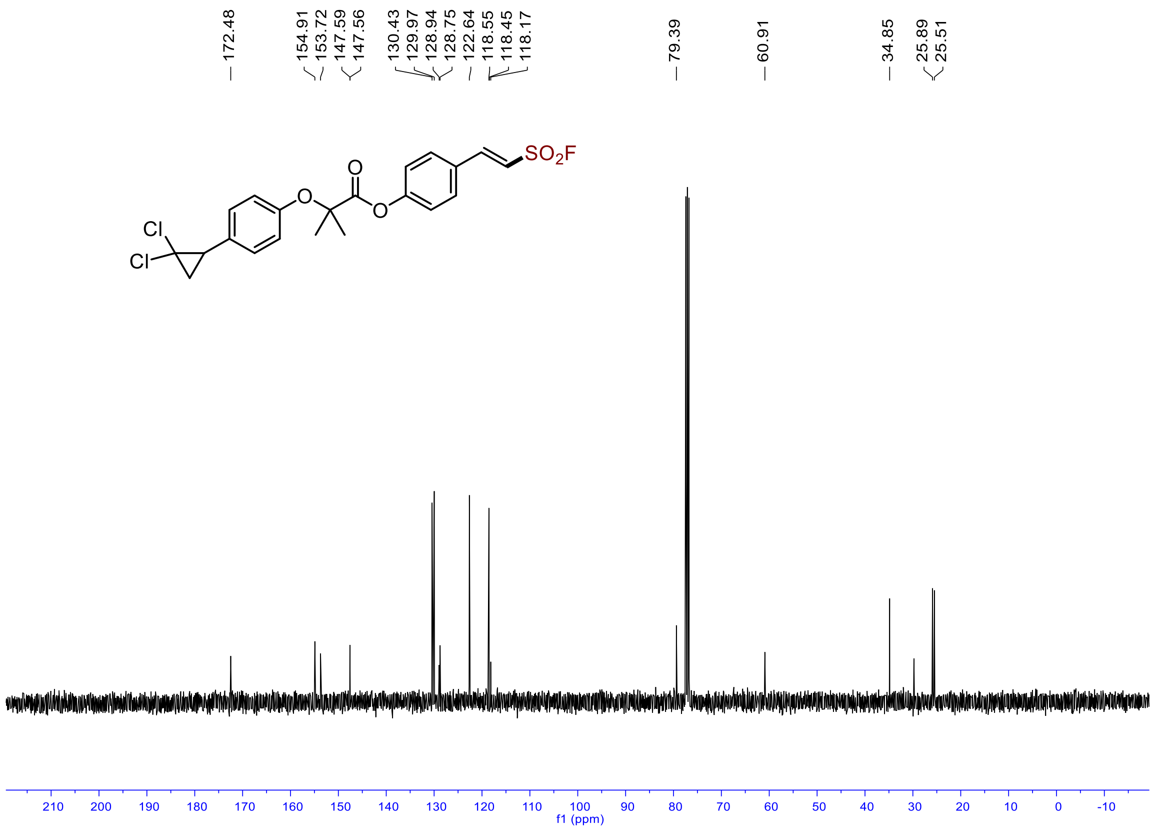

Supplementary Figure 144. ^{13}C NMR spectra of **5aq**

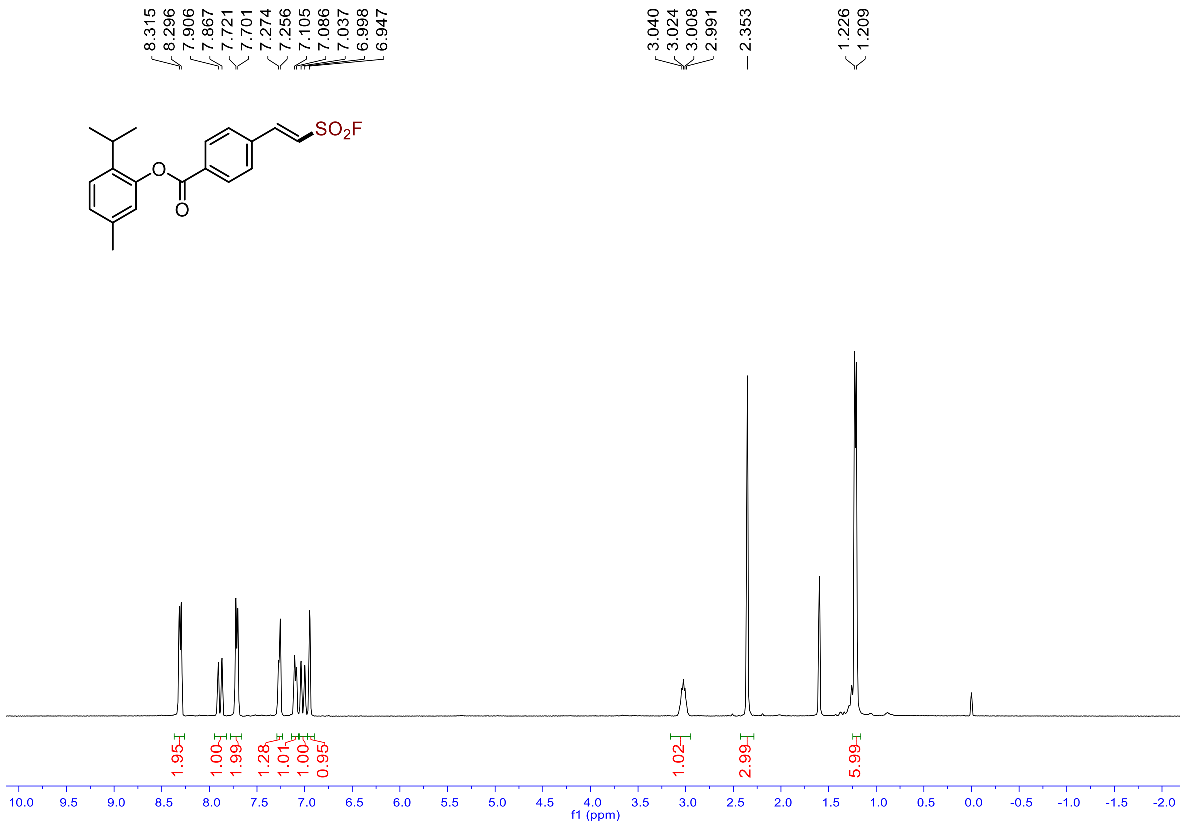

Supplementary Figure 145. ^{1}H NMR spectra of **5ar**

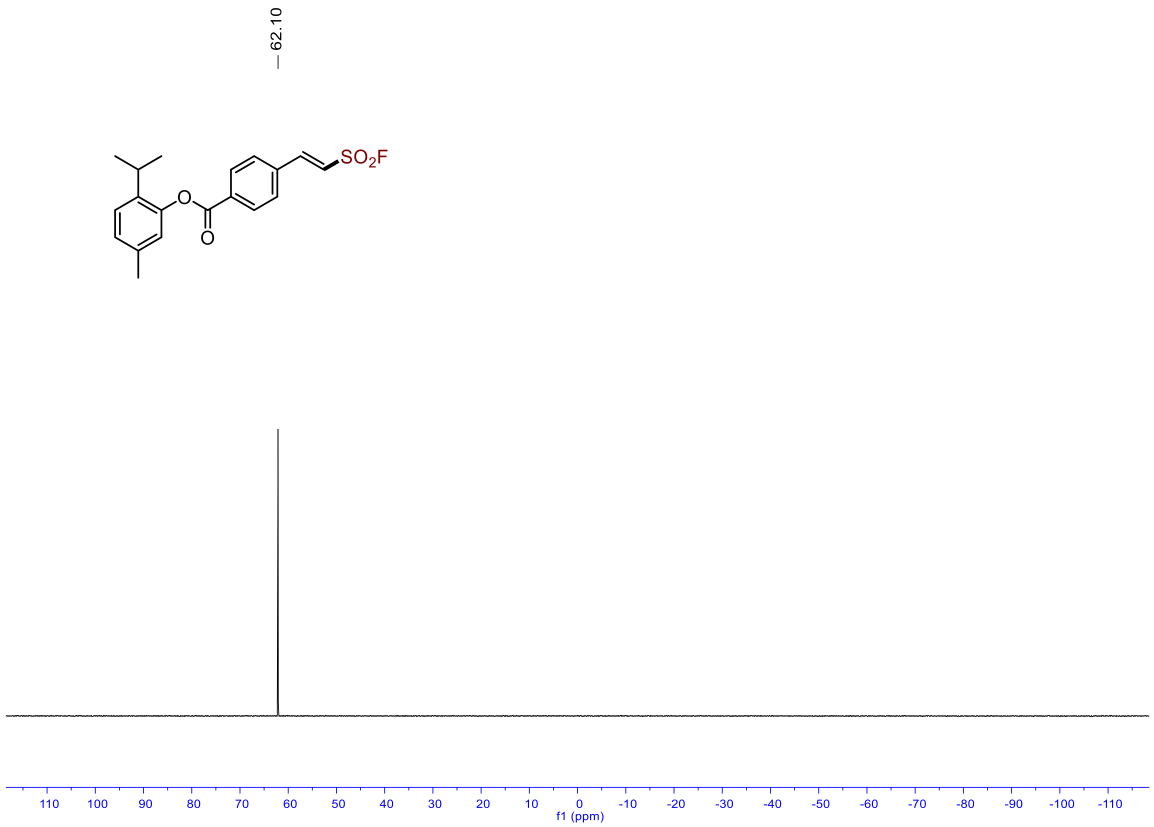

Supplementary Figure 146. ^{19}F NMR spectra of **5ar**

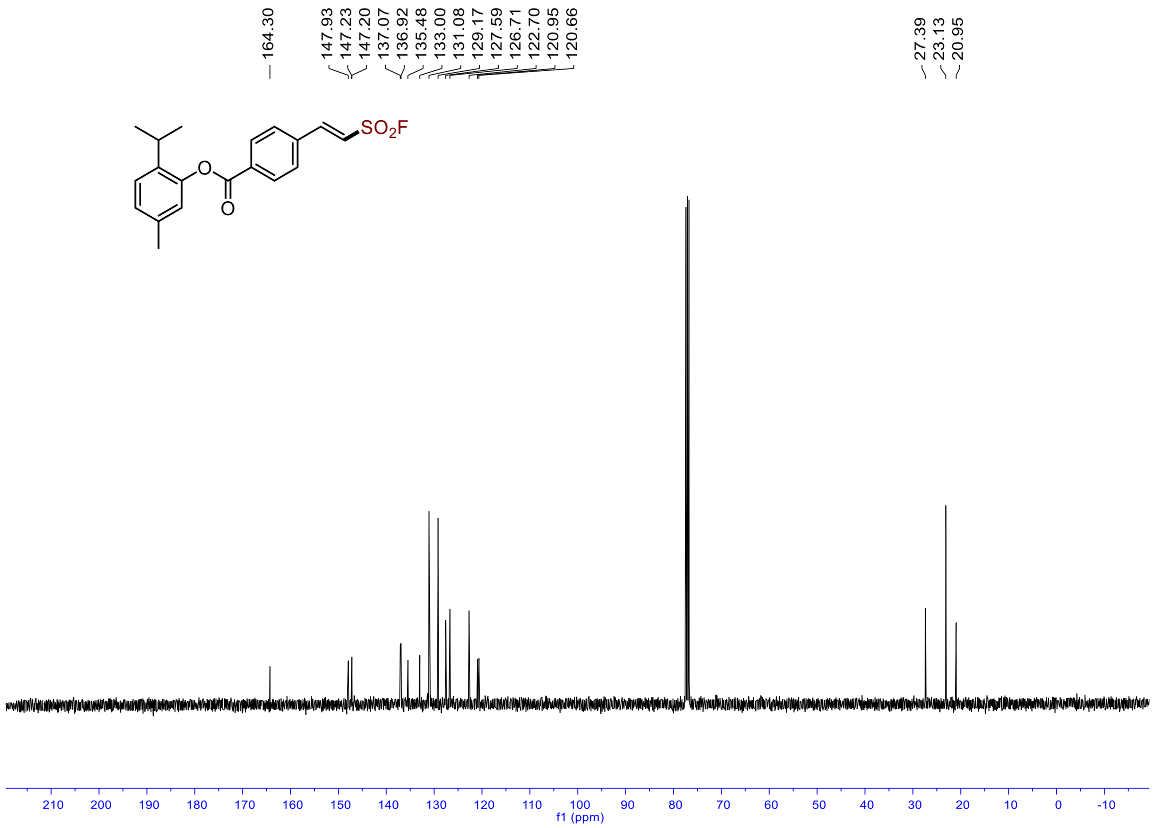

Supplementary Figure 147. ^{13}C NMR spectra of **5ar**

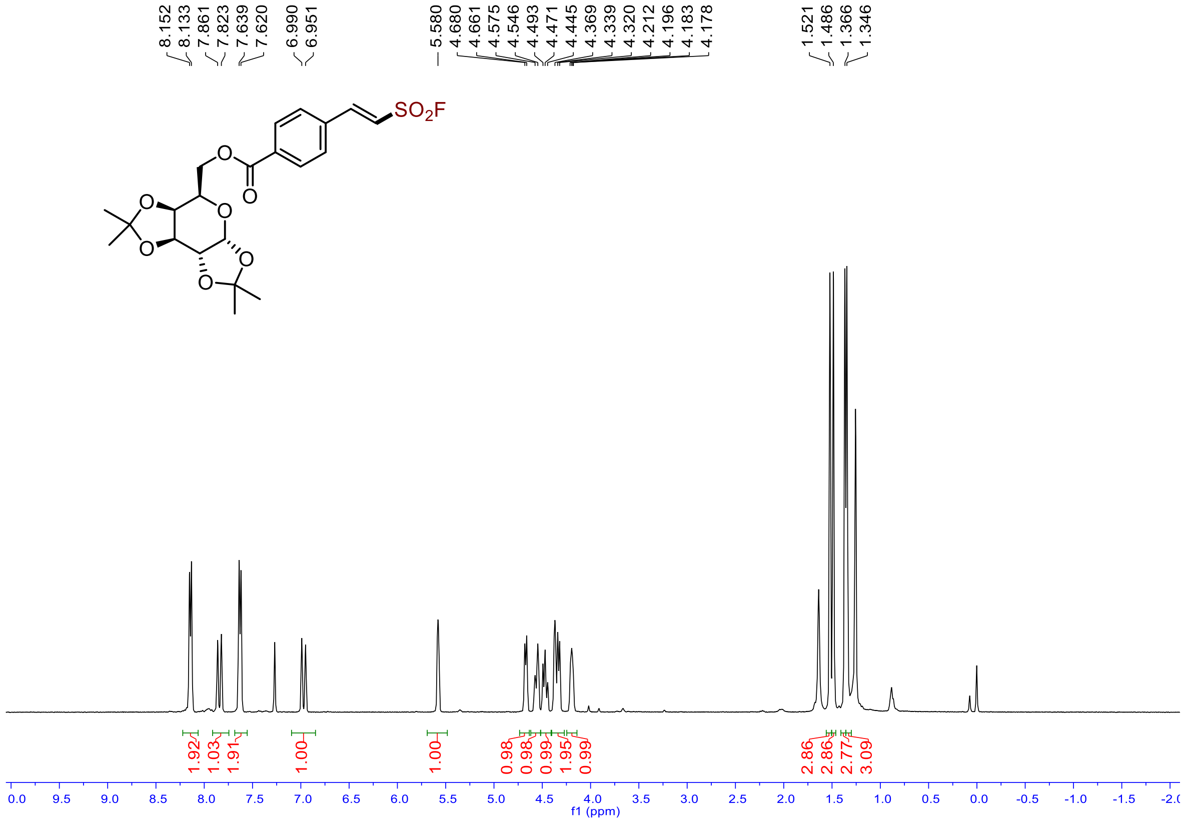

Supplementary Figure 148. ^{1}H NMR spectra of **5as**

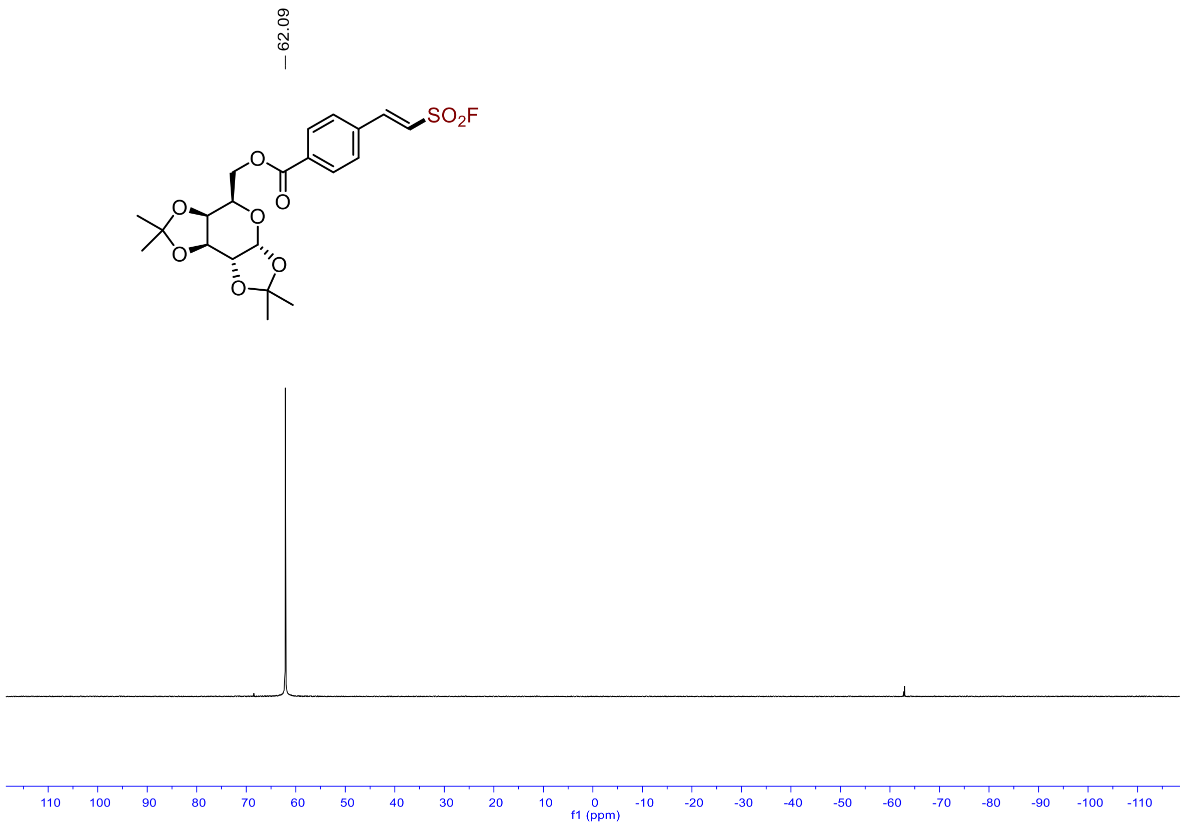

Supplementary Figure 149. ^{19}F NMR spectra of **5as**

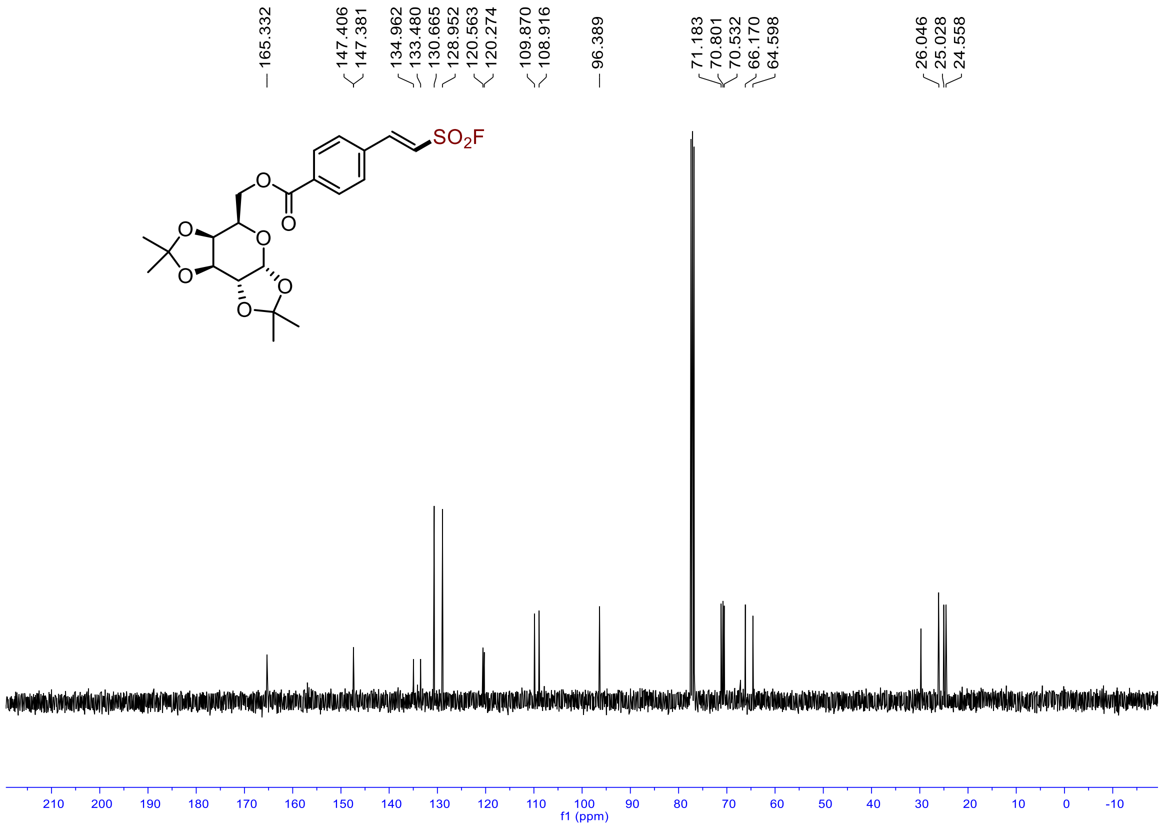

Supplementary Figure 150. ^{13}C NMR spectra of **5as**

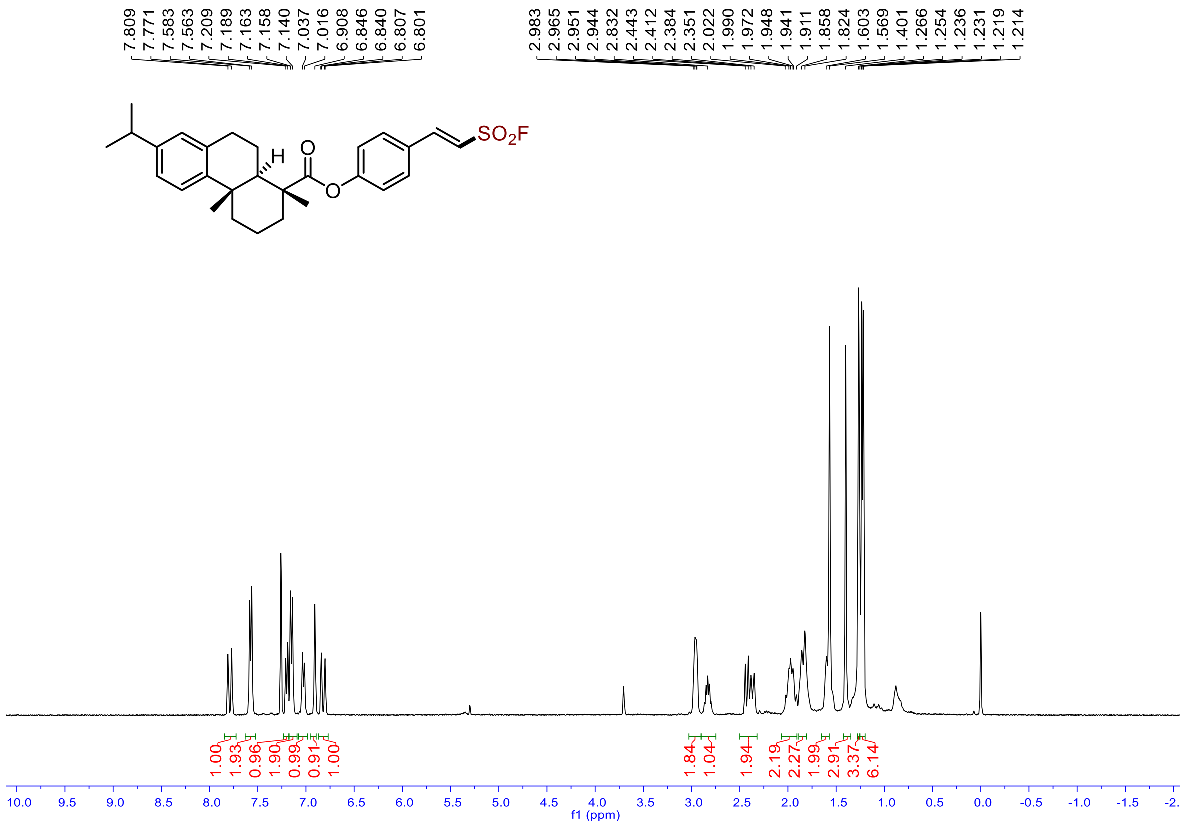

Supplementary Figure 151. ^{1}H NMR spectra of **5at**

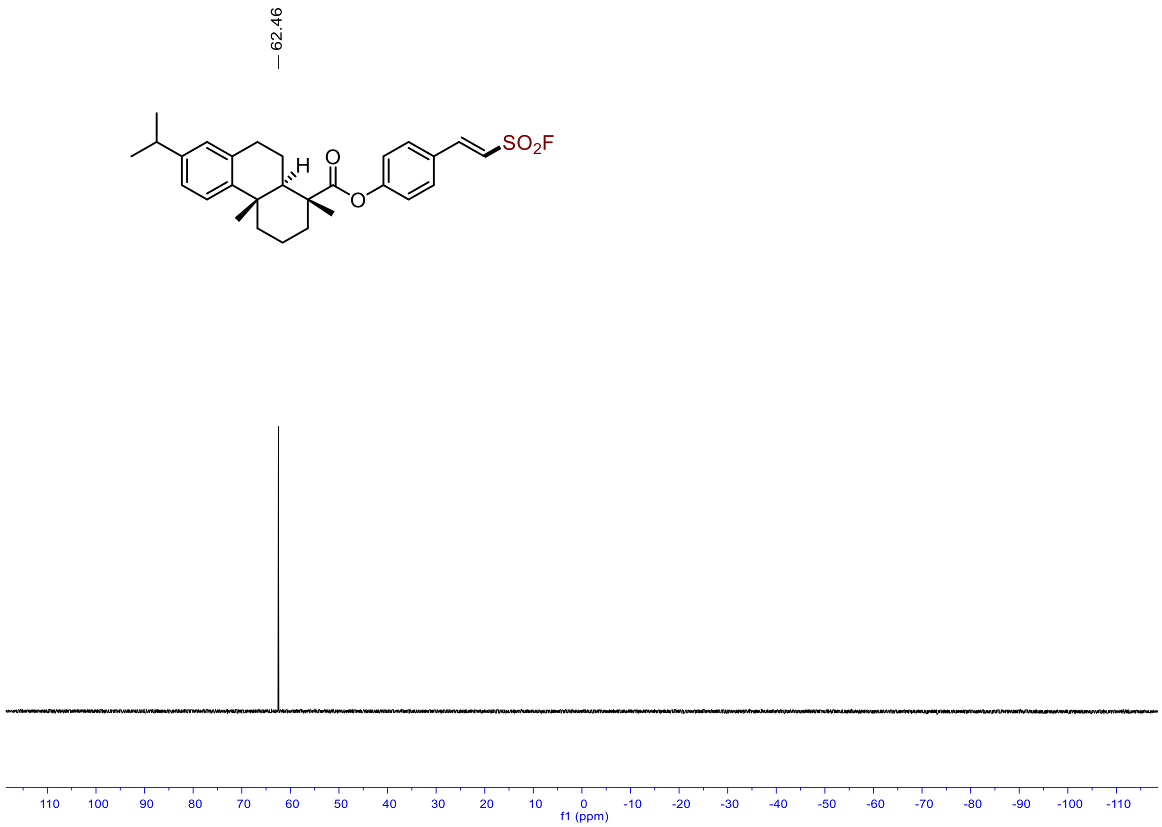

Supplementary Figure 152. ^{19}F NMR spectra of **5at**

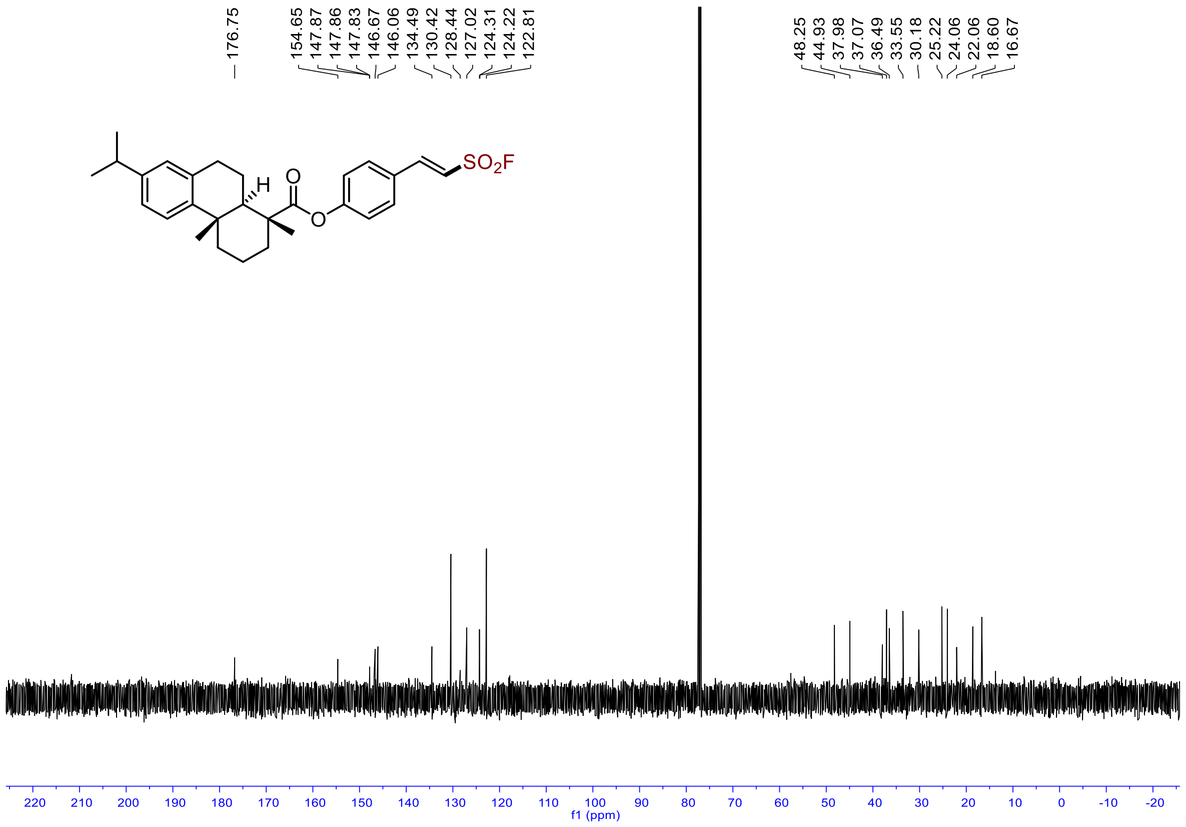

Supplementary Figure 153. ^{13}C NMR spectra of **5at**

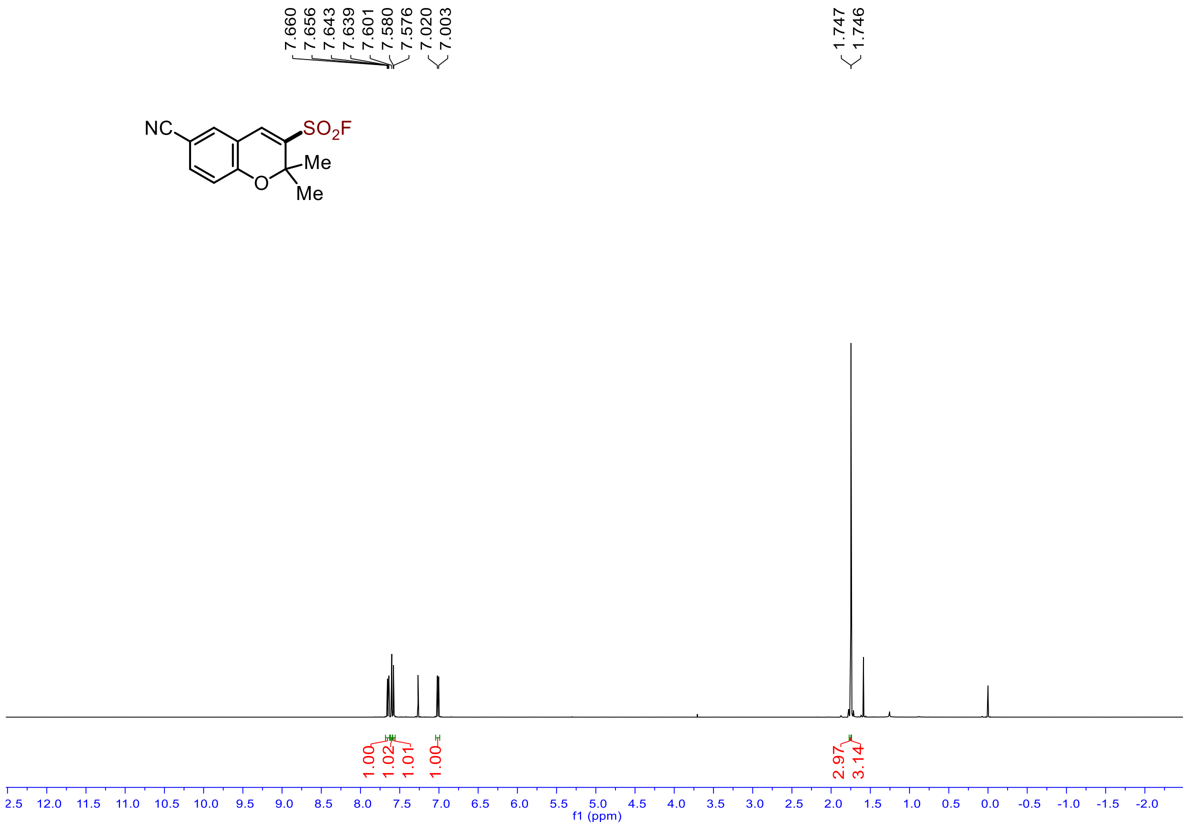

Supplementary Figure 154. ^{1}H NMR spectra of **5au**

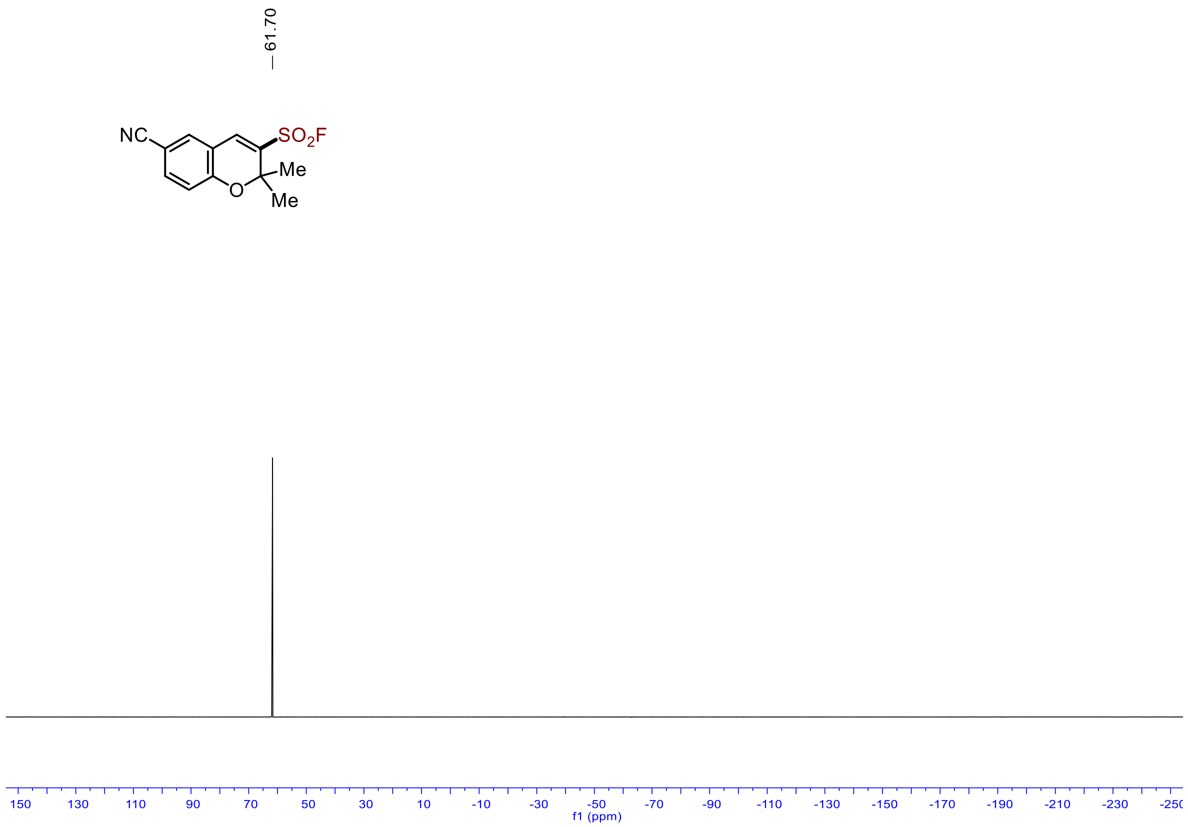

Supplementary Figure 155. ^{19}F NMR spectra of **5au**

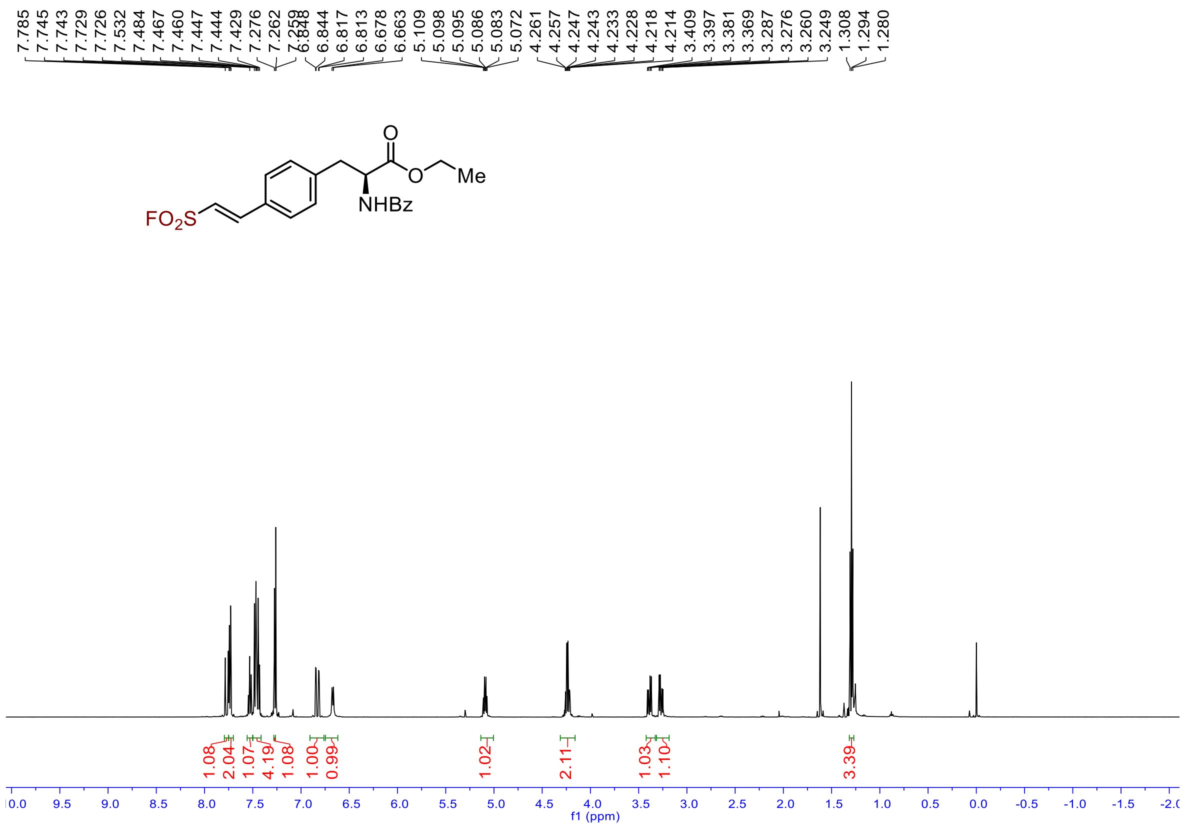

Supplementary Figure 156. ^{1}H NMR spectra of **5av**

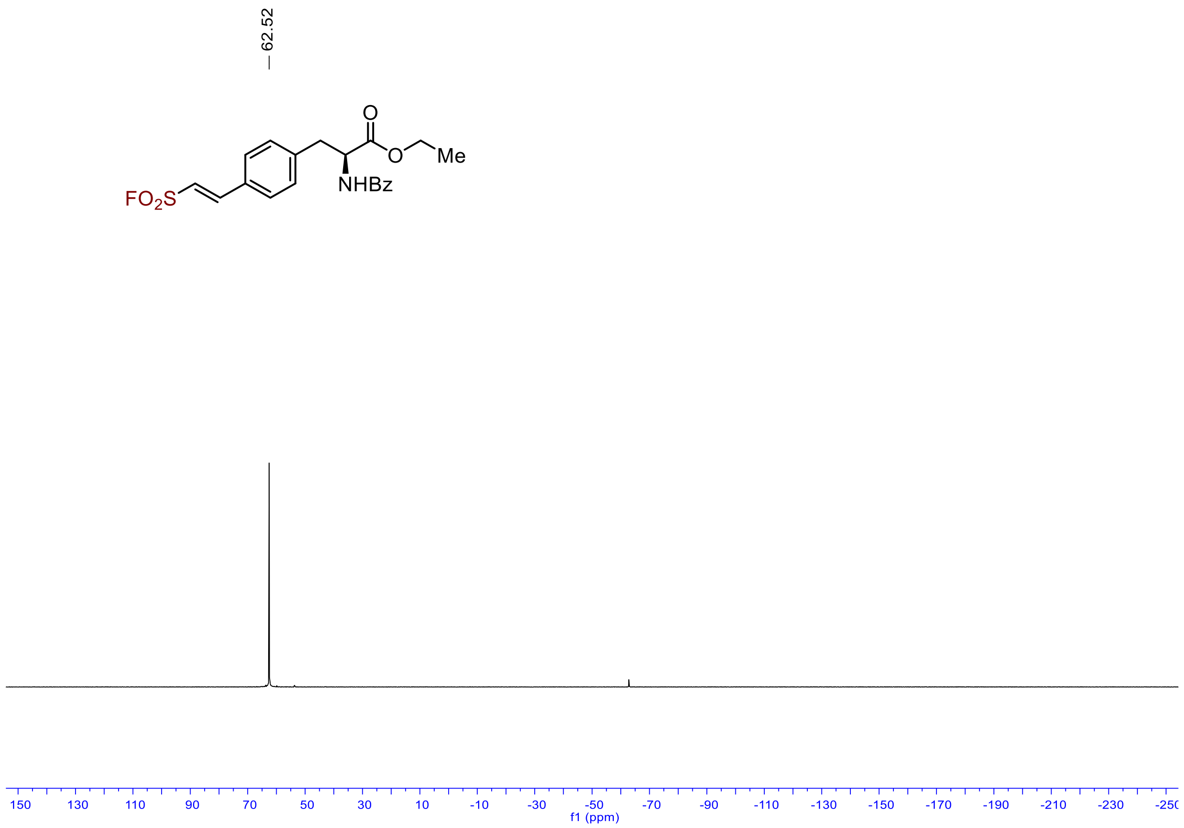

Supplementary Figure 157. ^{19}F NMR spectra of **5av**

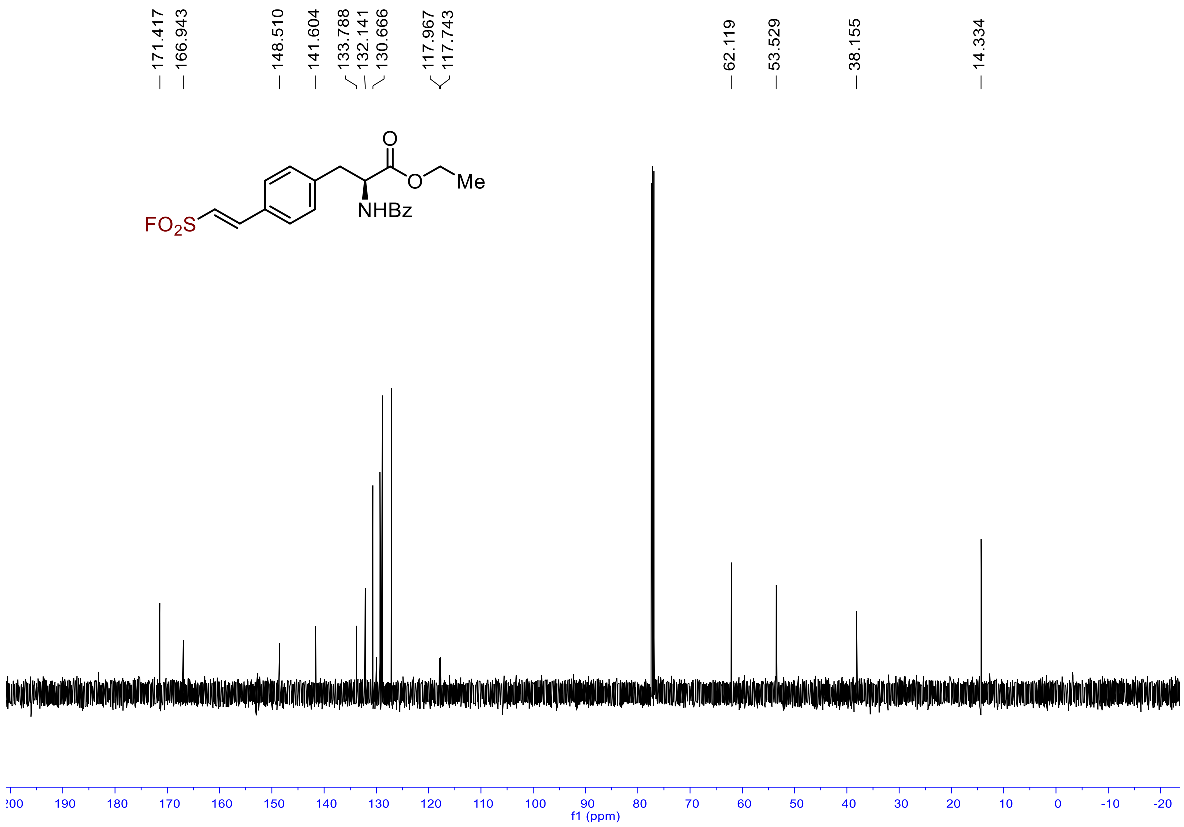

Supplementary Figure 158. ^{13}C NMR spectra of **5av**

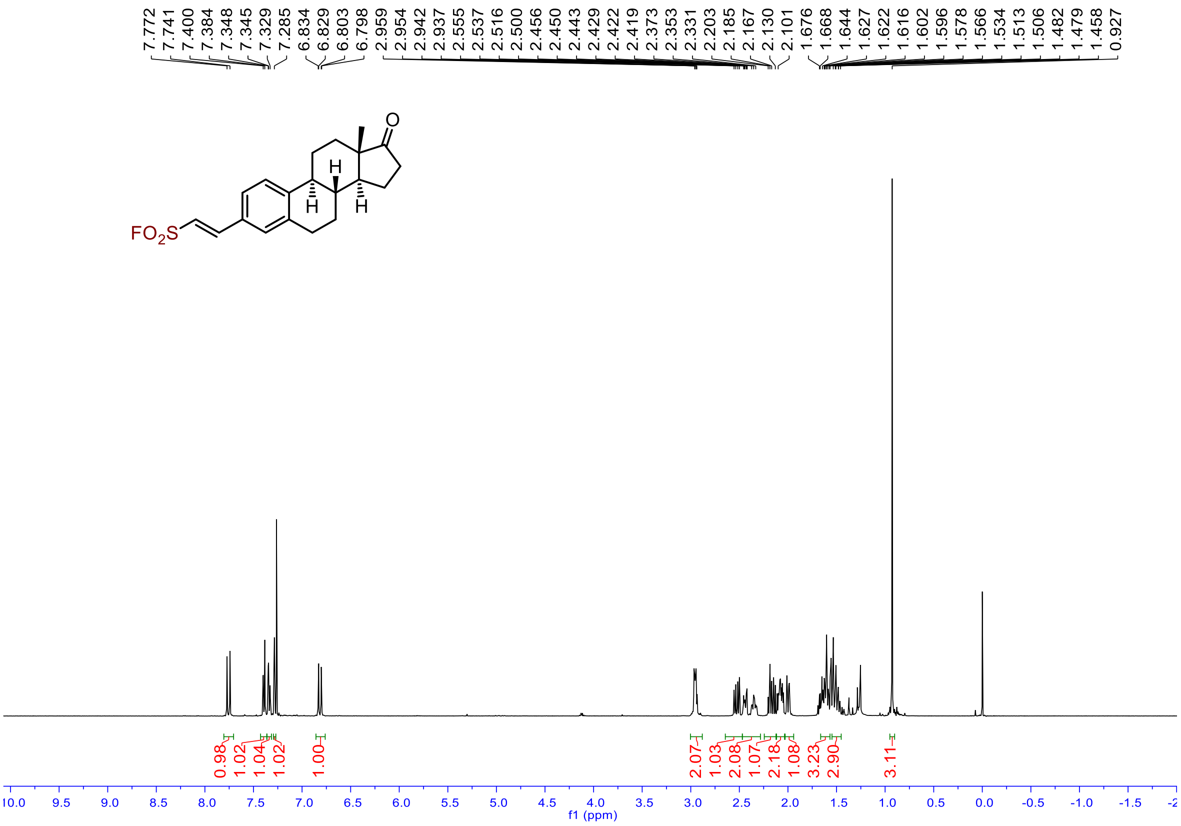

Supplementary Figure 159. ^{1}H NMR spectra of **5aw**

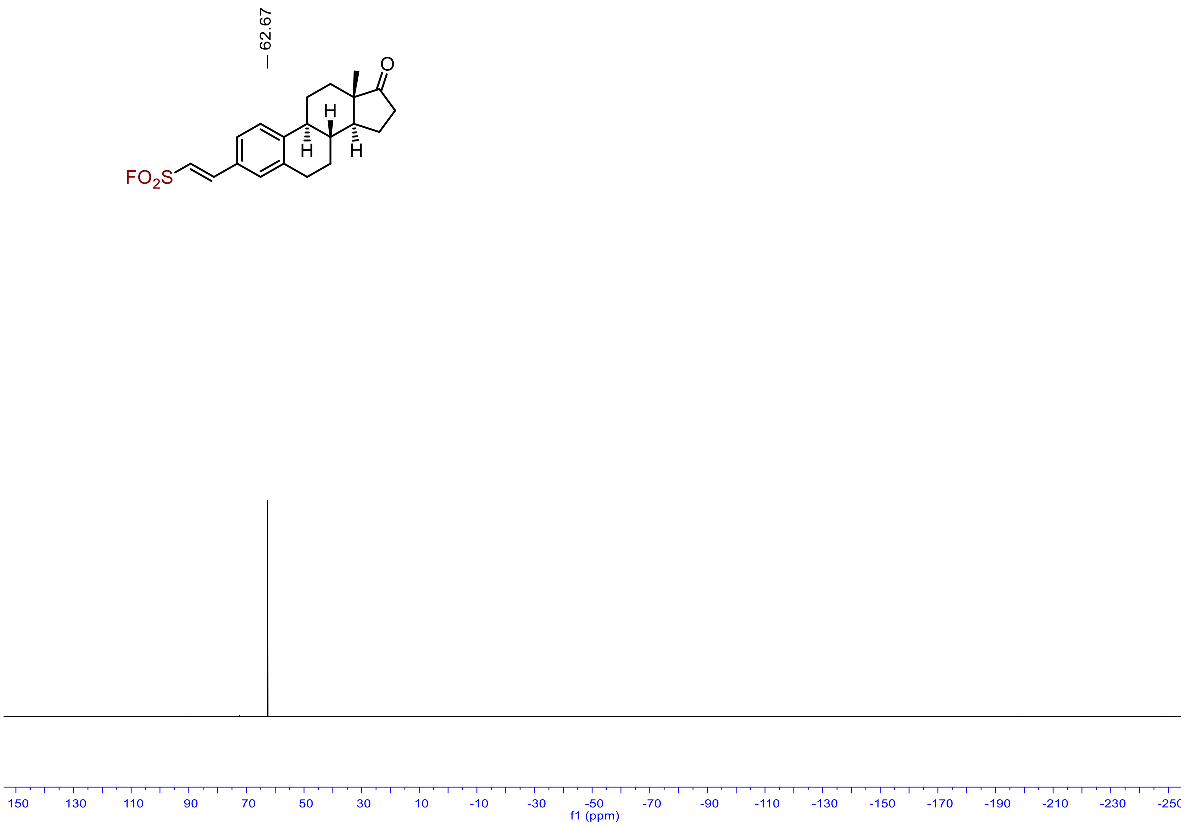

Supplementary Figure 160. ^{19}F NMR spectra of **5aw**

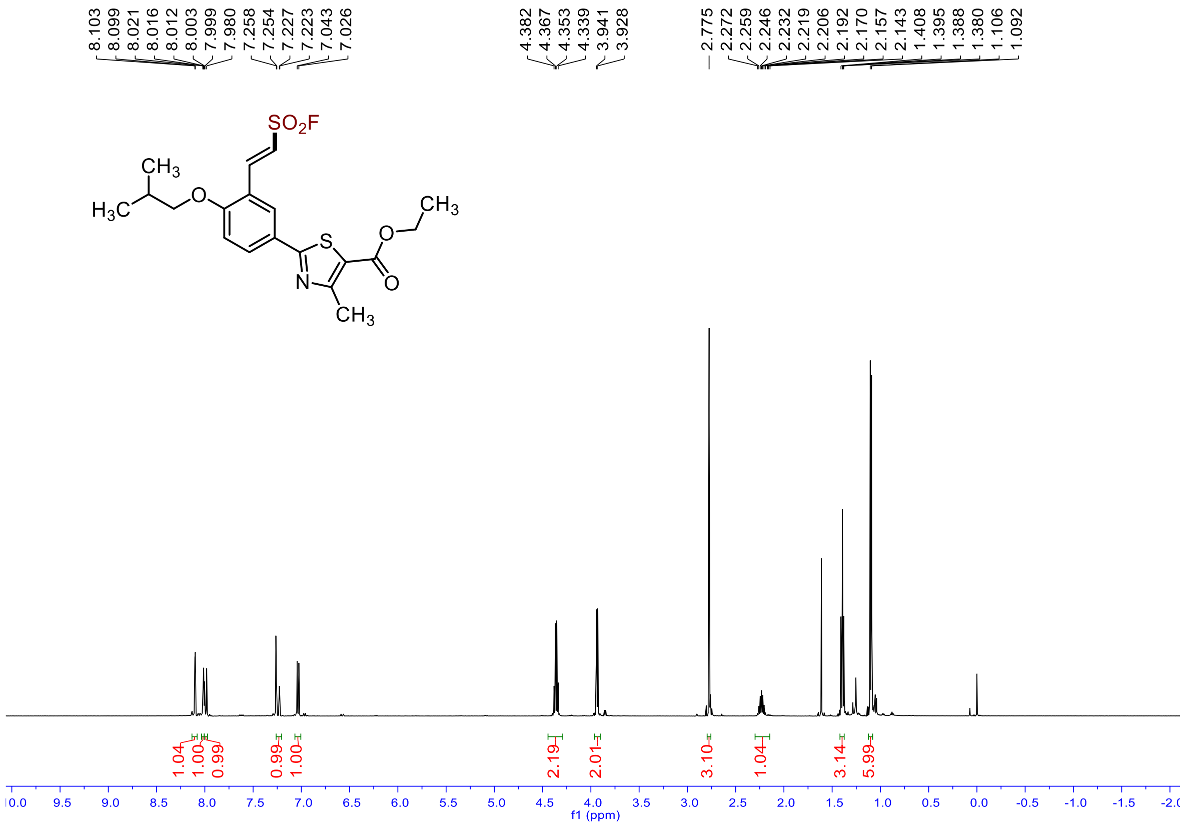

Supplementary Figure 161. ^{1}H NMR spectra of **5ax**

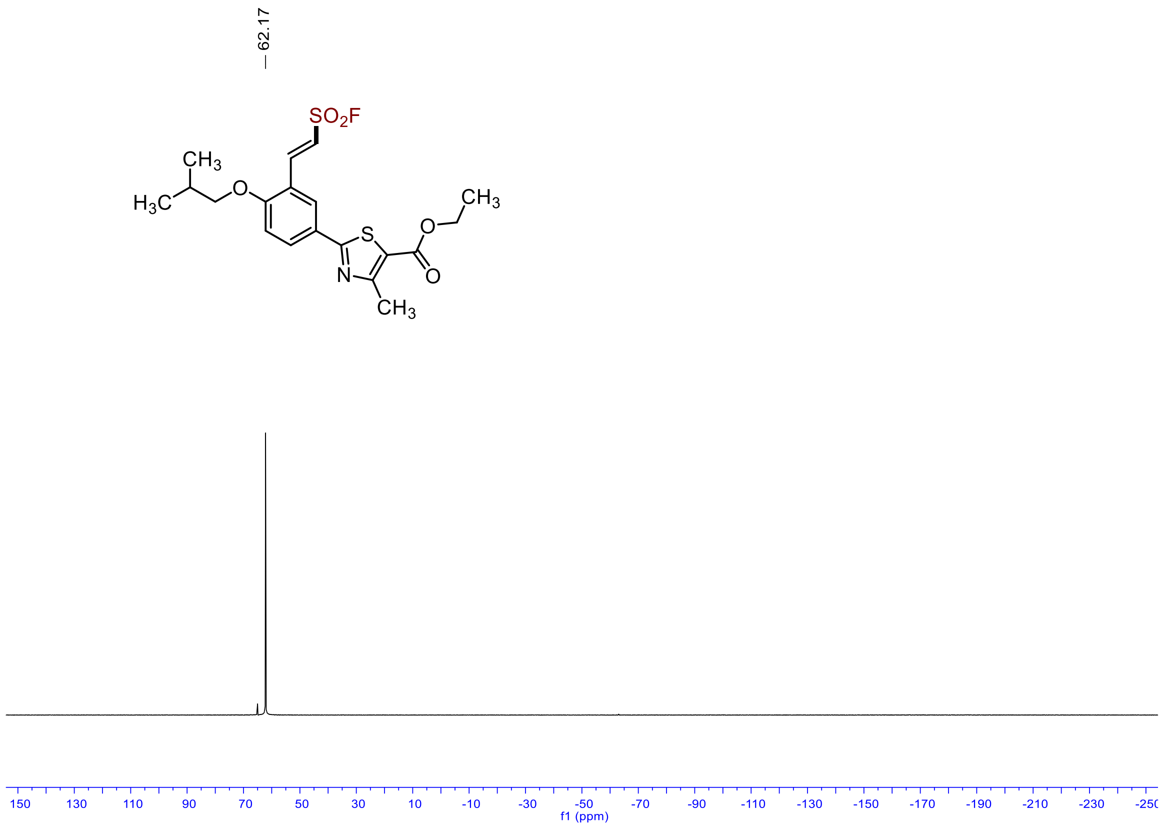

Supplementary Figure 162. ^{19}F NMR spectra of **5ax**

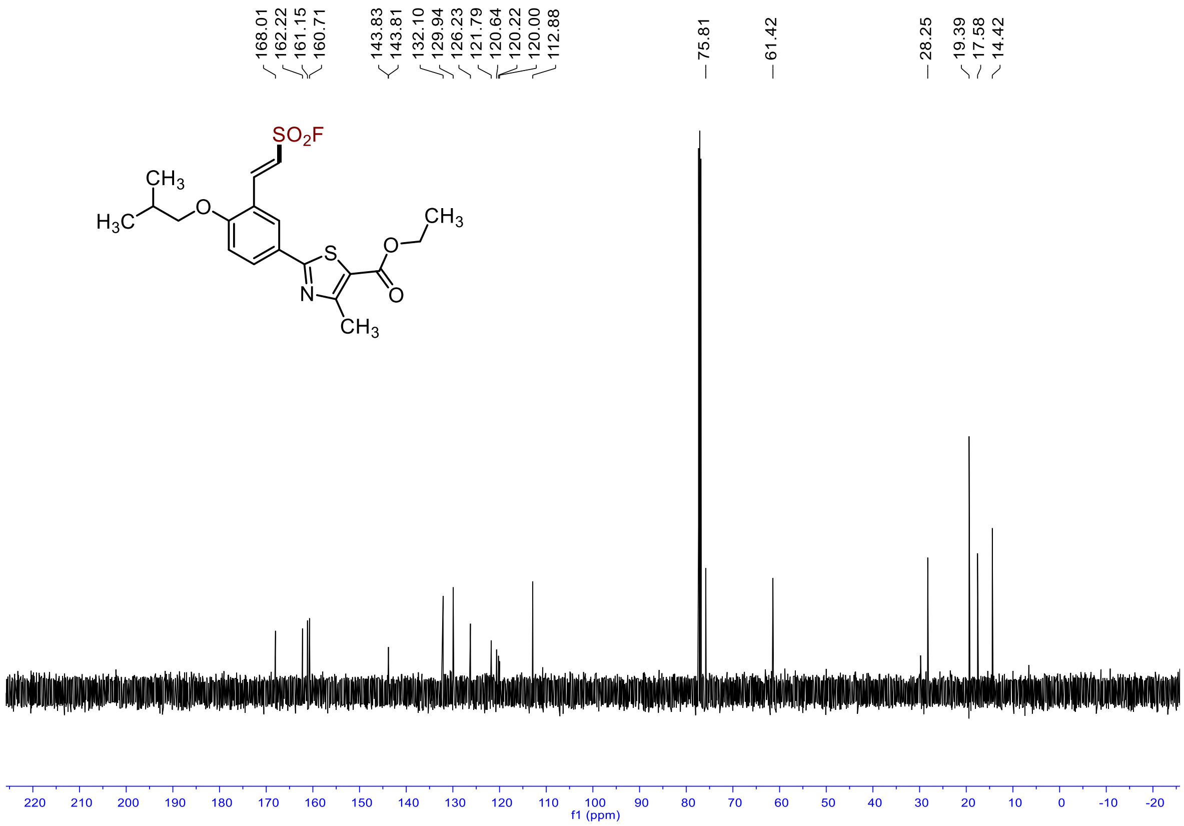

Supplementary Figure 163. ^{13}C NMR spectra of **5ax**

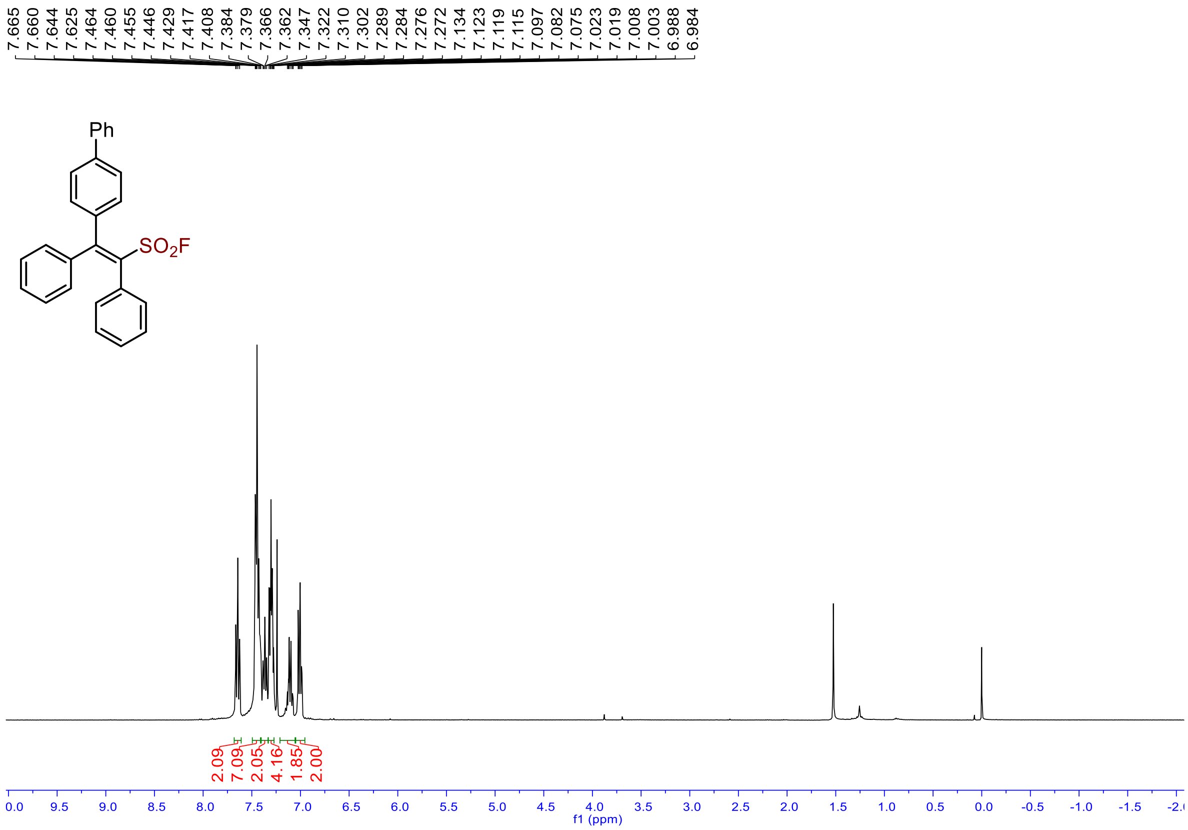

Supplementary Figure 164. ^{1}H NMR spectra of **5ay**

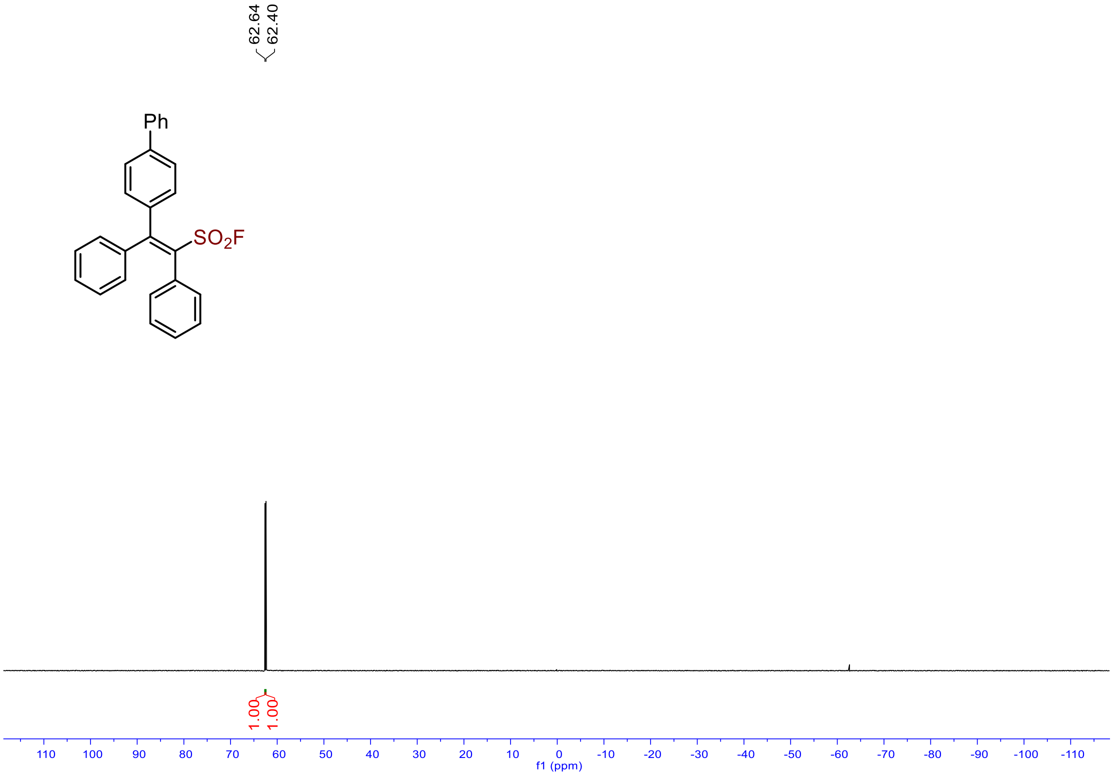

Supplementary Figure 165. ^{19}F NMR spectra of **5ay**

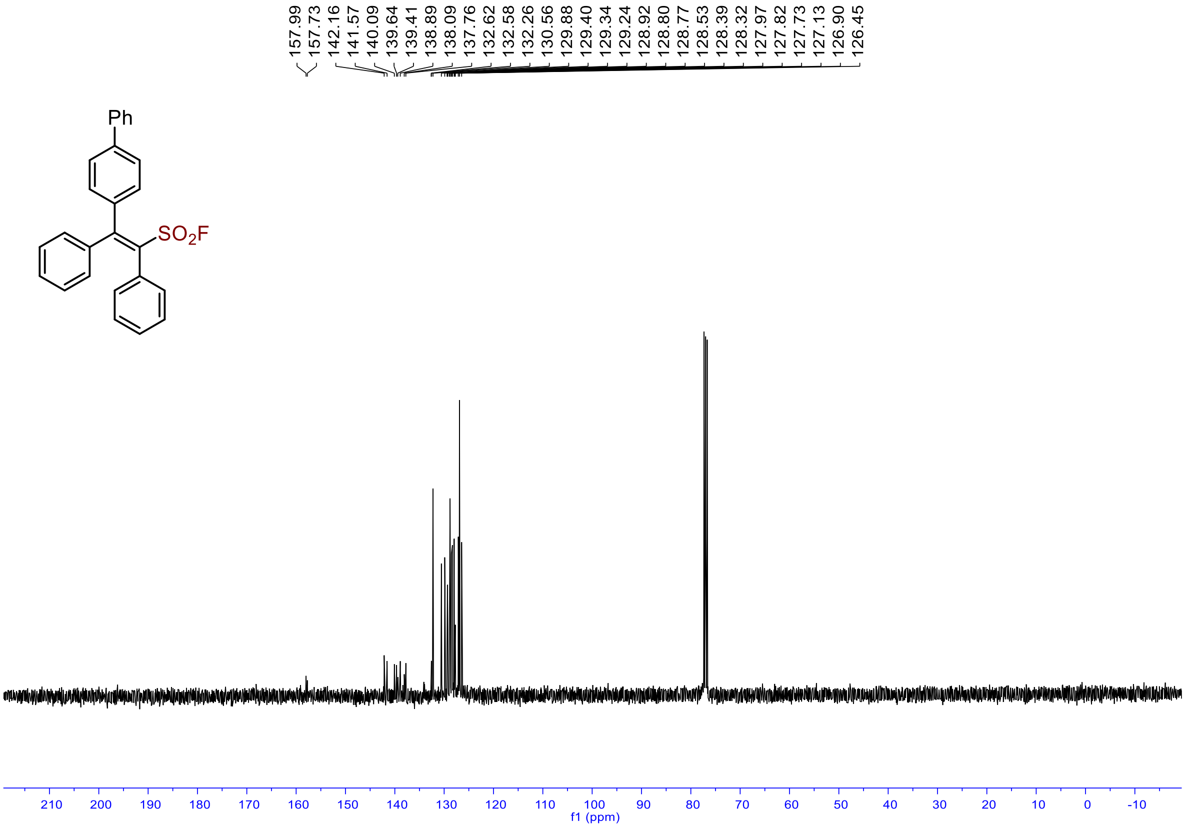

Supplementary Figure 166. ^{13}C NMR spectra of **5ay**

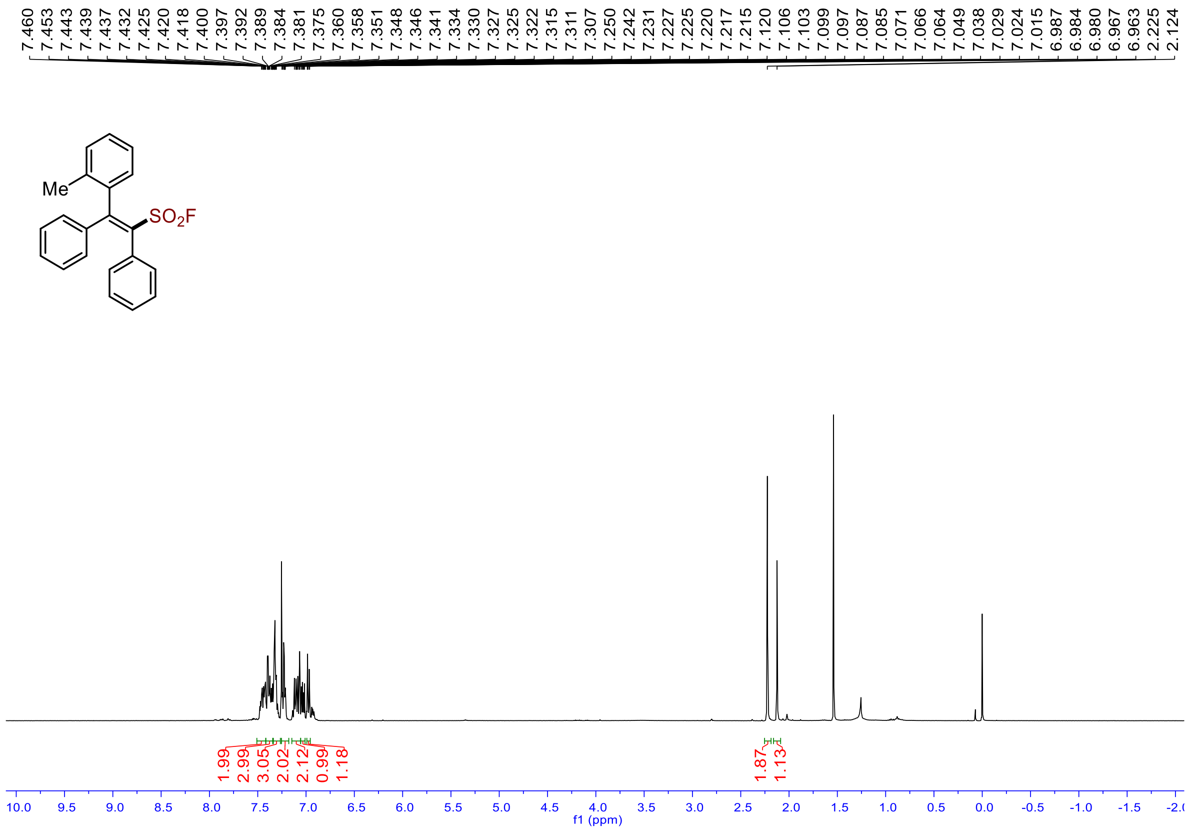

Supplementary Figure 167. ^{1}H NMR spectra of **5az**

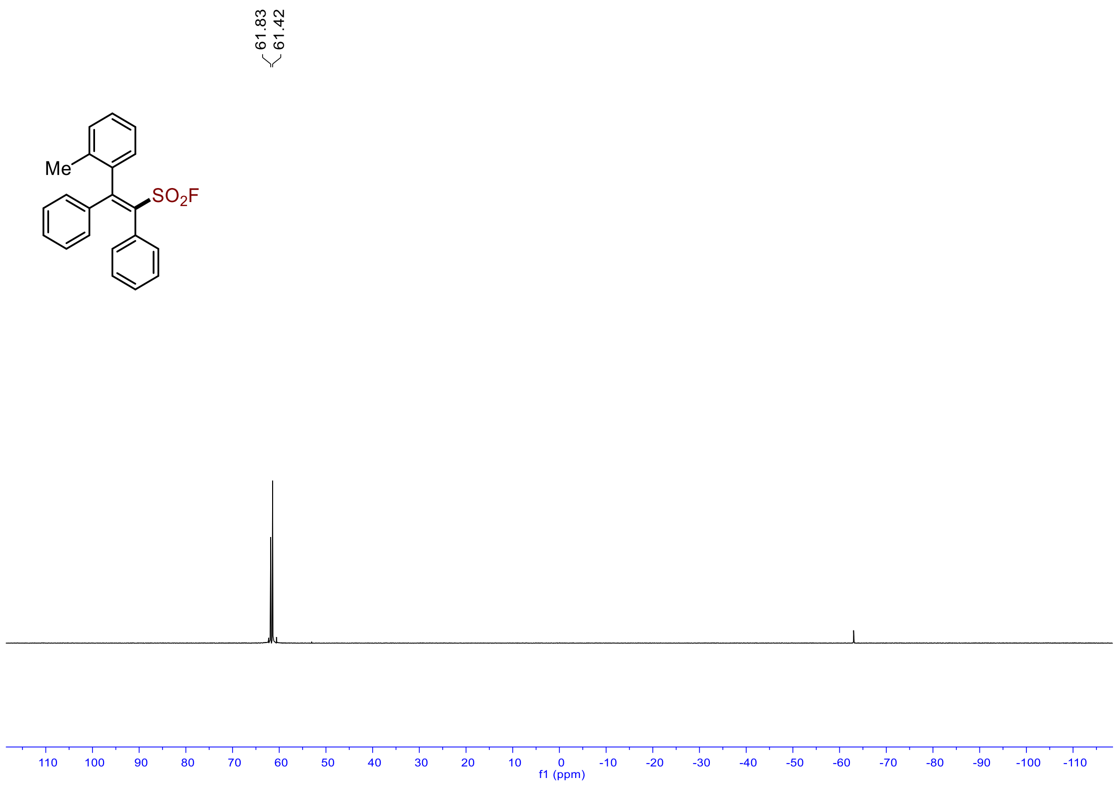

Supplementary Figure 168. ^{19}F NMR spectra of **5az**

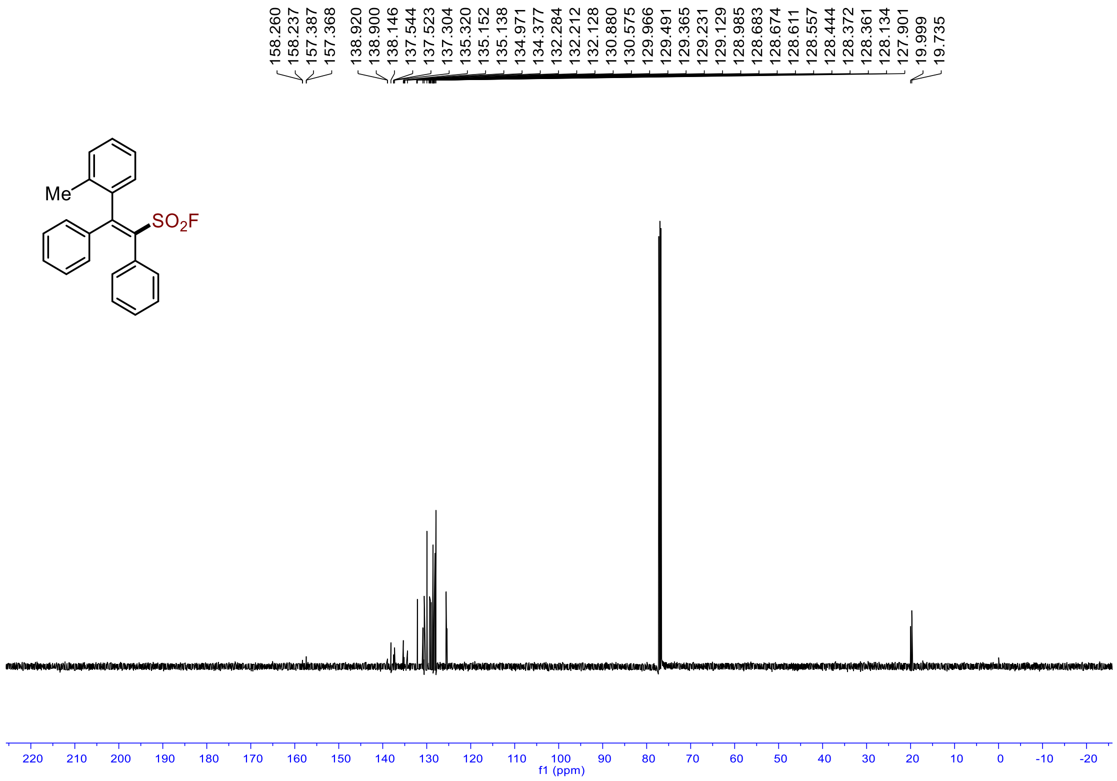

Supplementary Figure 169. ^{13}C NMR spectra of **5az**

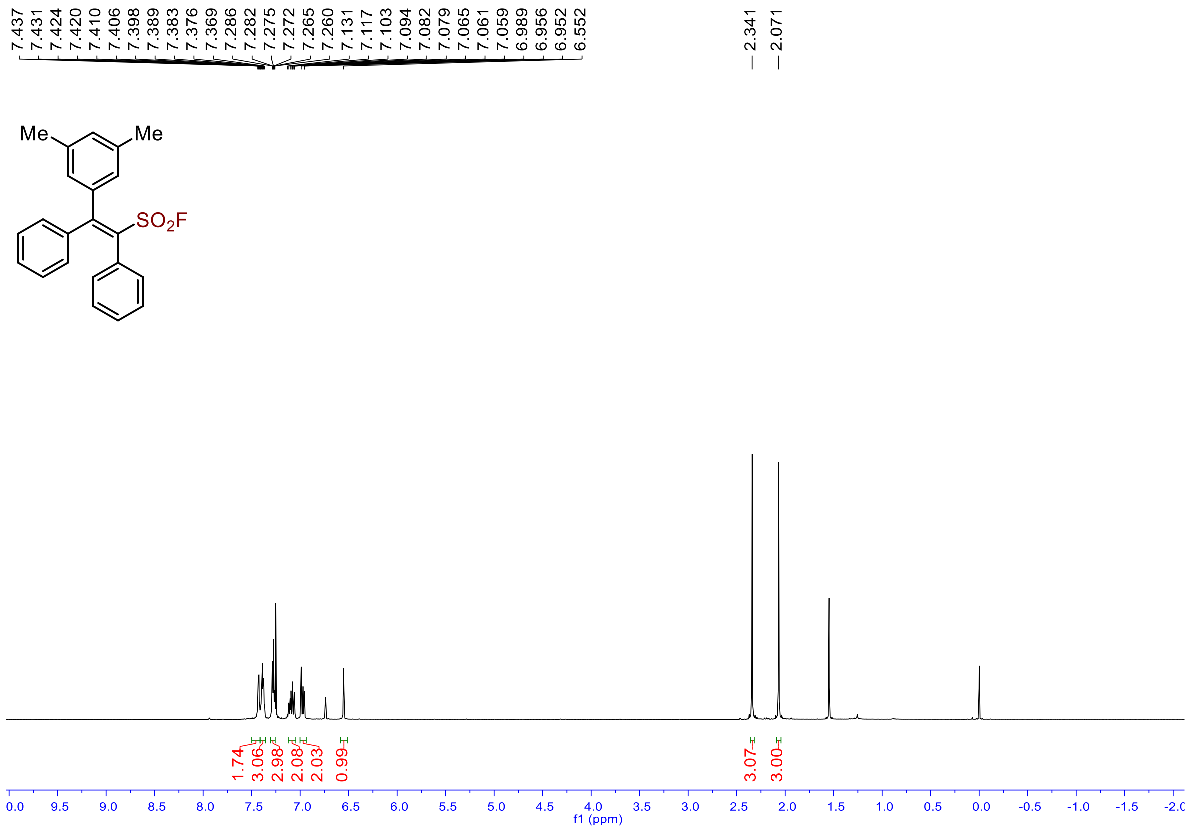

Supplementary Figure 170. ^{1}H NMR spectra of **5ba**

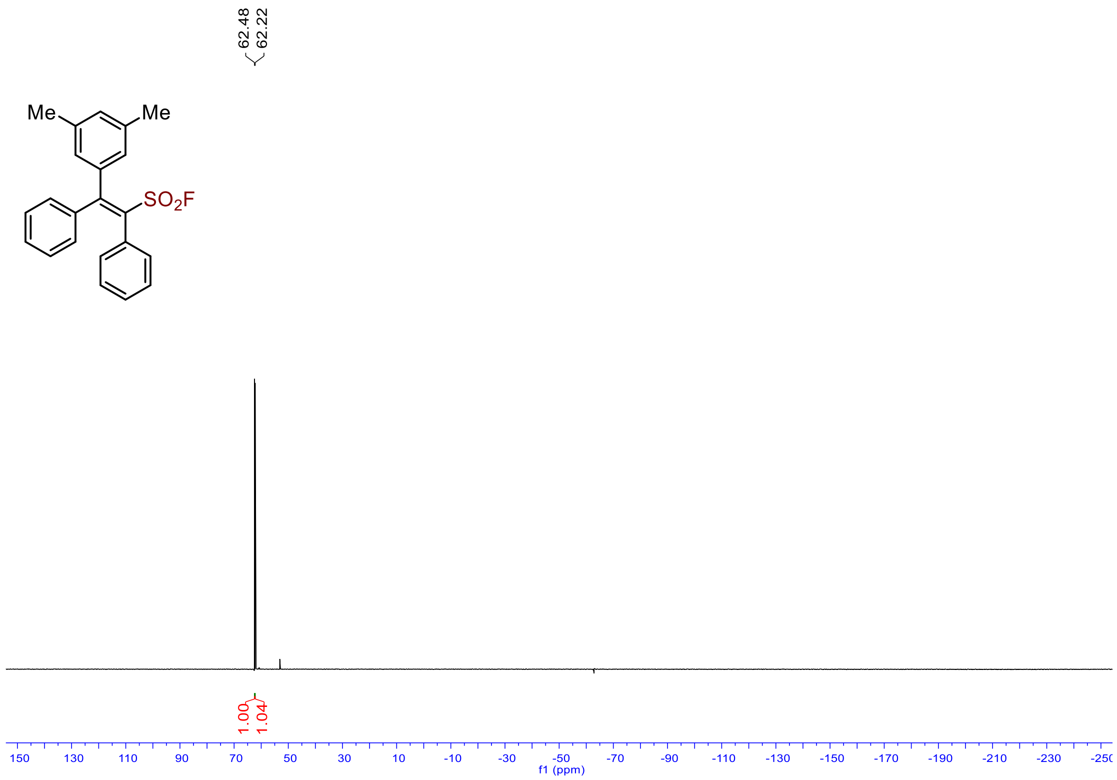

Supplementary Figure 171. ^{19}F NMR spectra of **5ba**

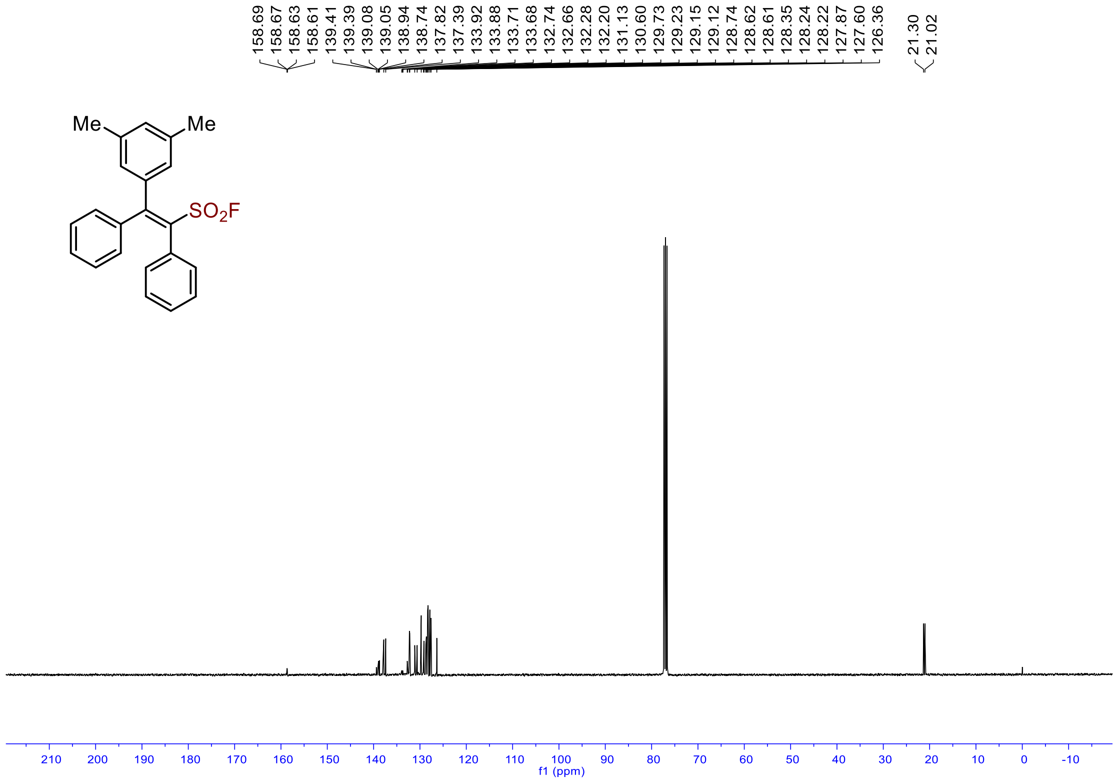

Supplementary Figure 172. ^{13}C NMR spectra of **5ba**

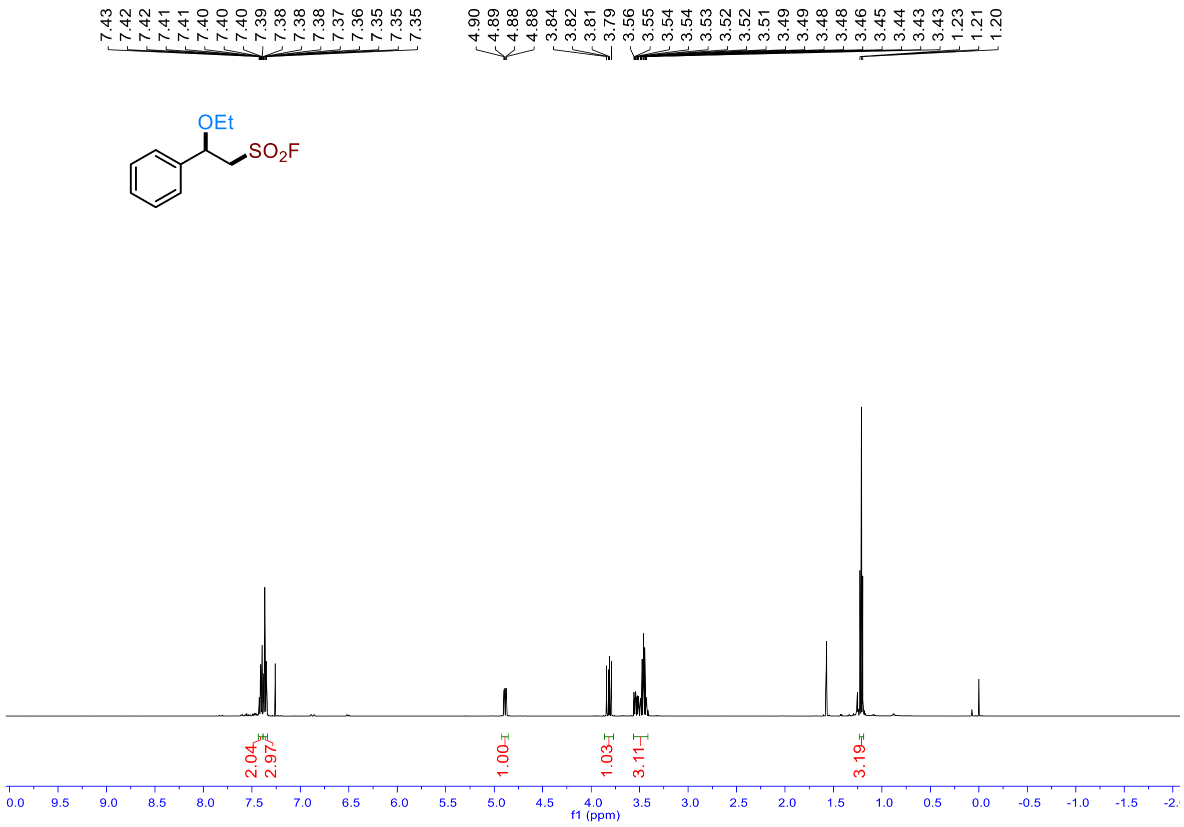

Supplementary Figure 173. ^{1}H NMR spectra of **7a**

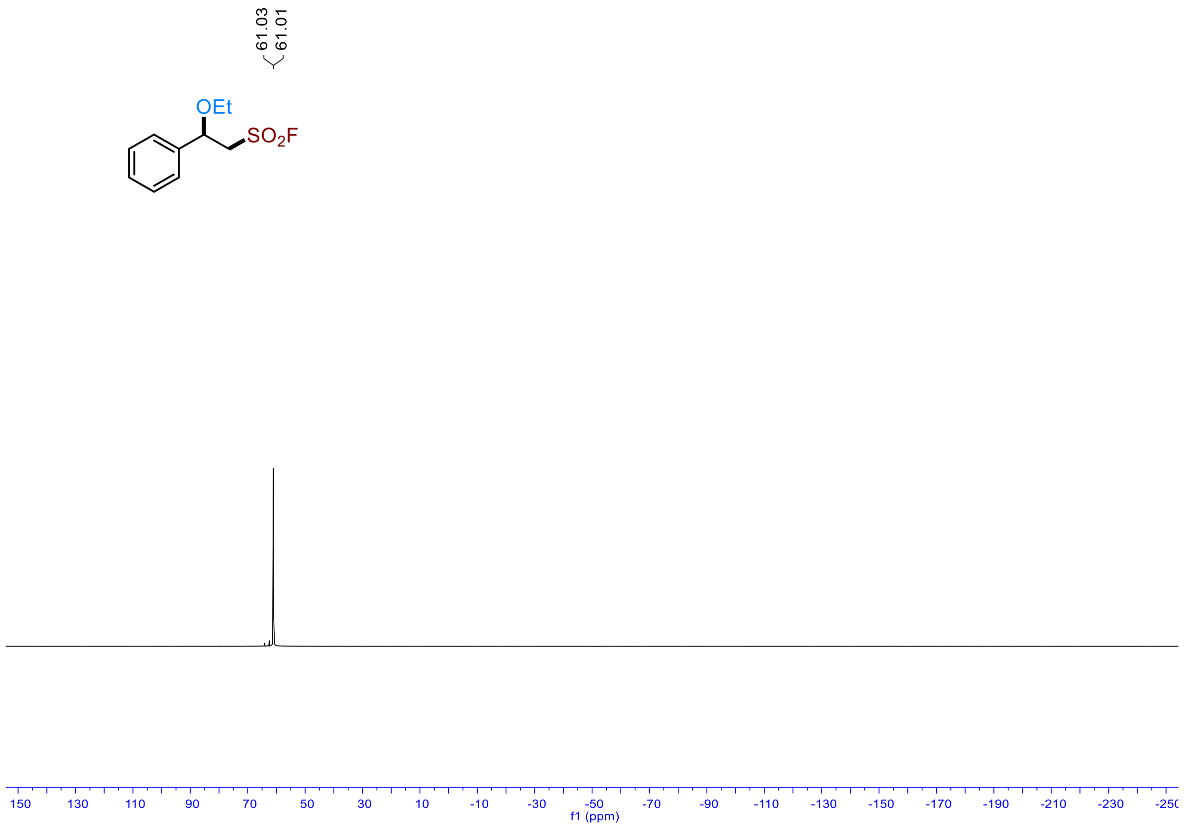

Supplementary Figure 174. ^{19}F NMR spectra of **7a**

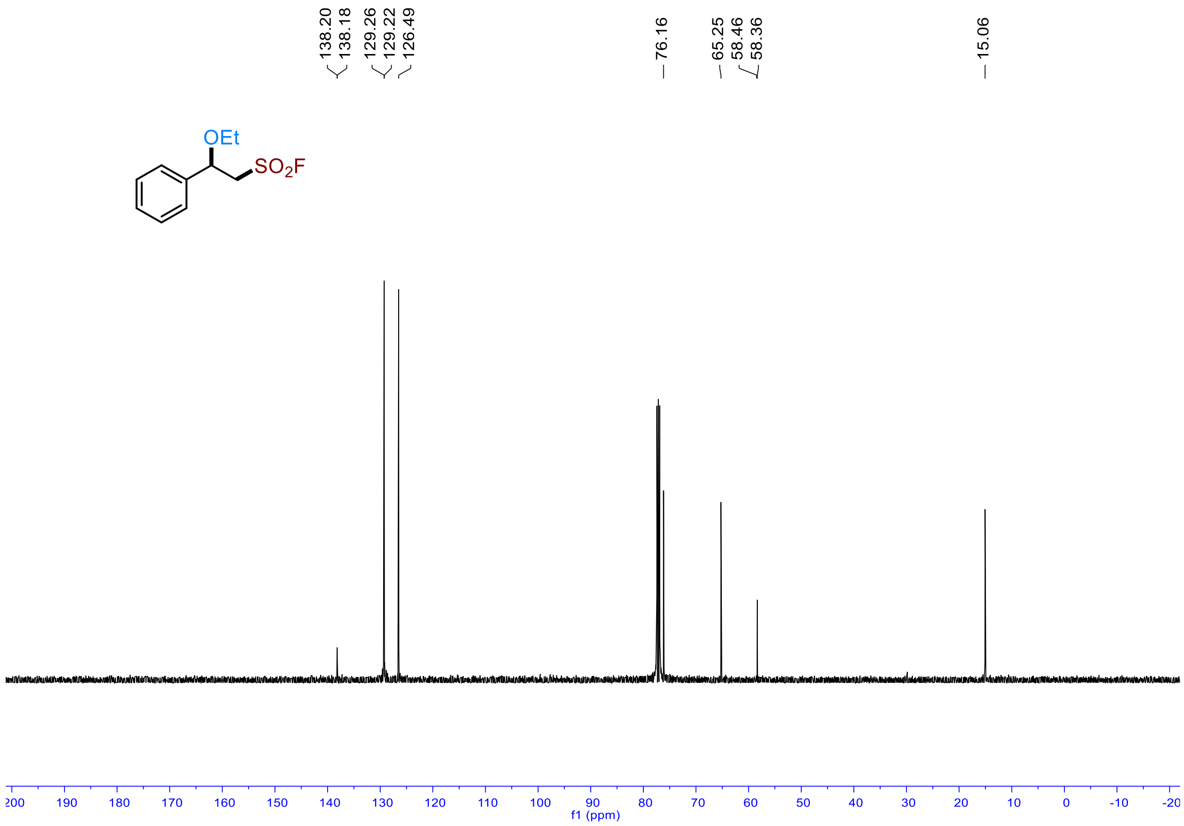

Supplementary Figure 175. ^{13}C NMR spectra of **7a**

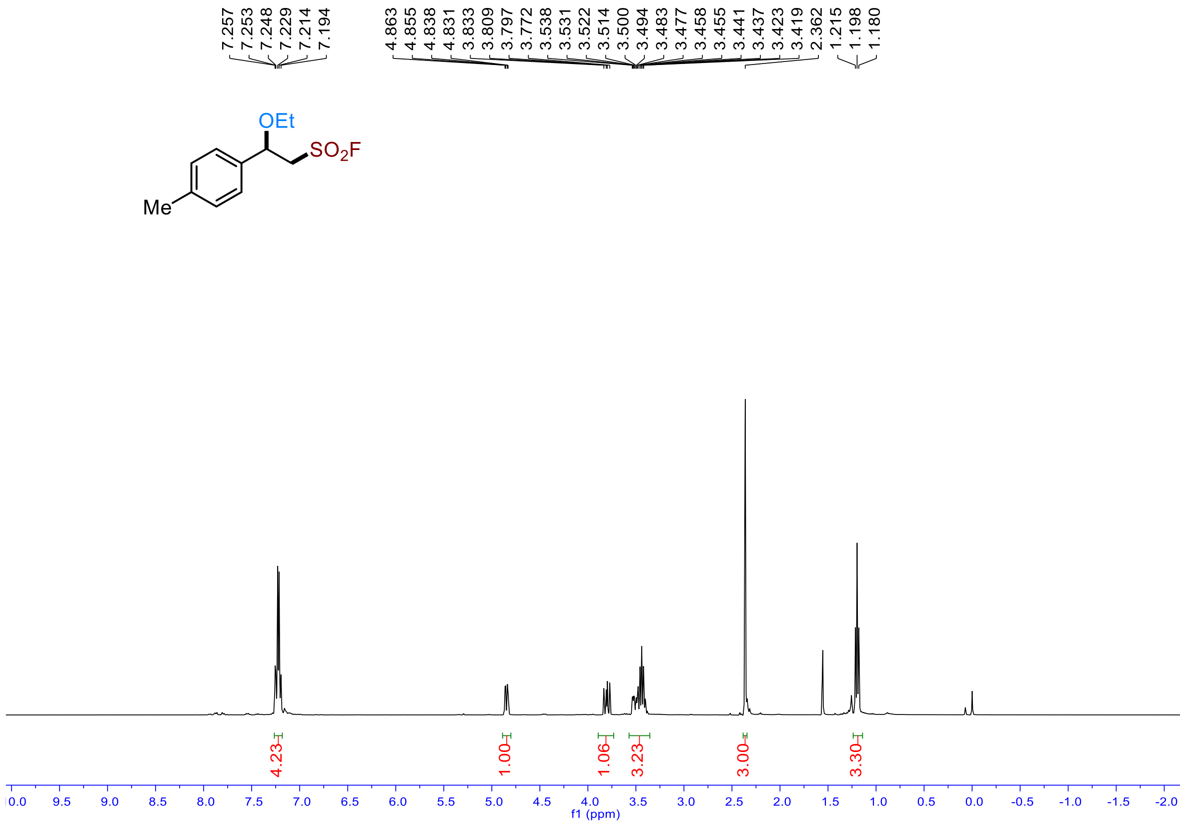

Supplementary Figure 176. ^{1}H NMR spectra of **7b**

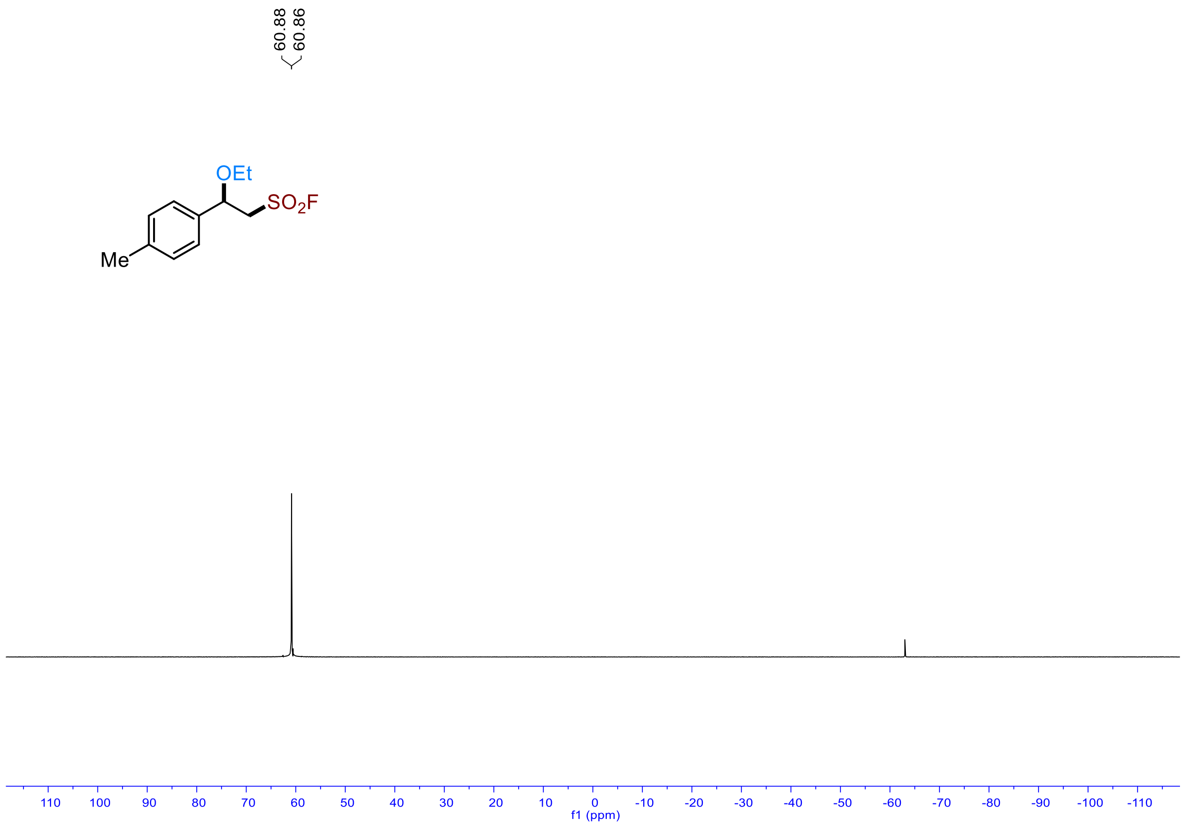

Supplementary Figure 177. ^{19}F NMR spectra of **7b**

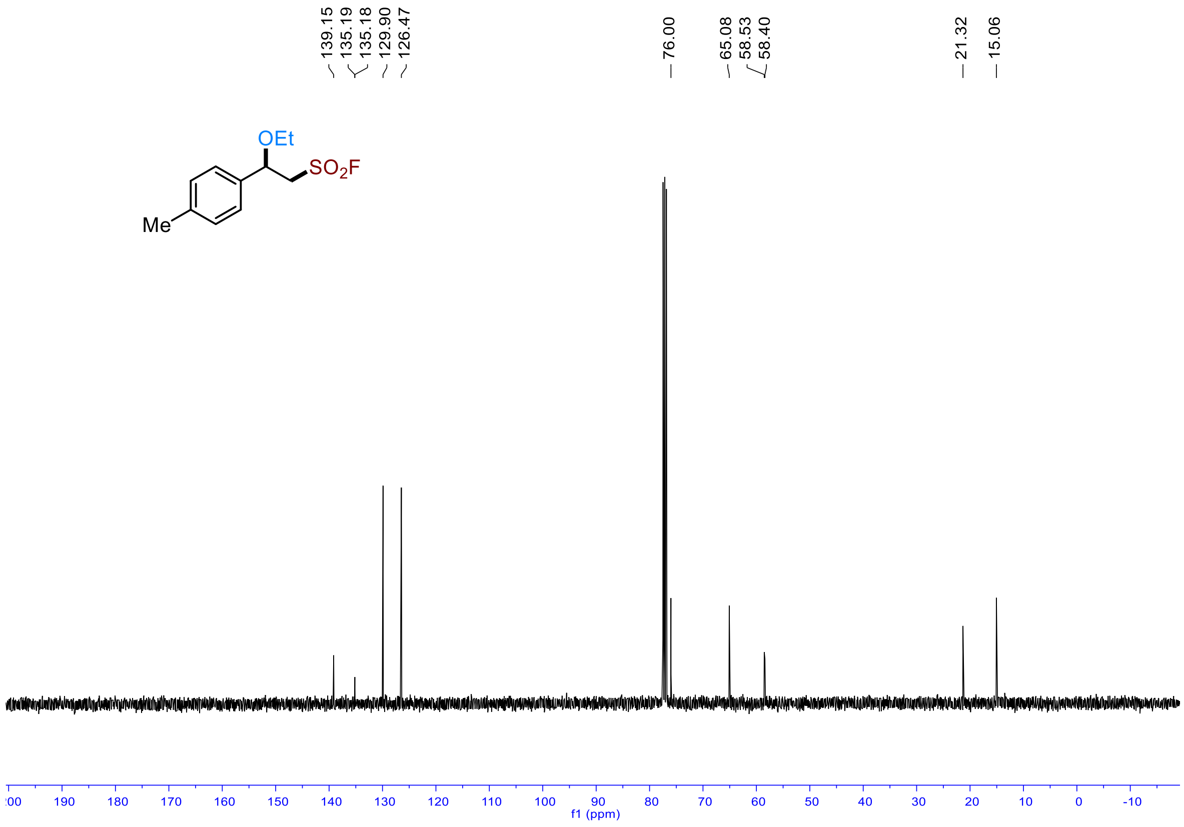

Supplementary Figure 178. ^{13}C NMR spectra of **7b**

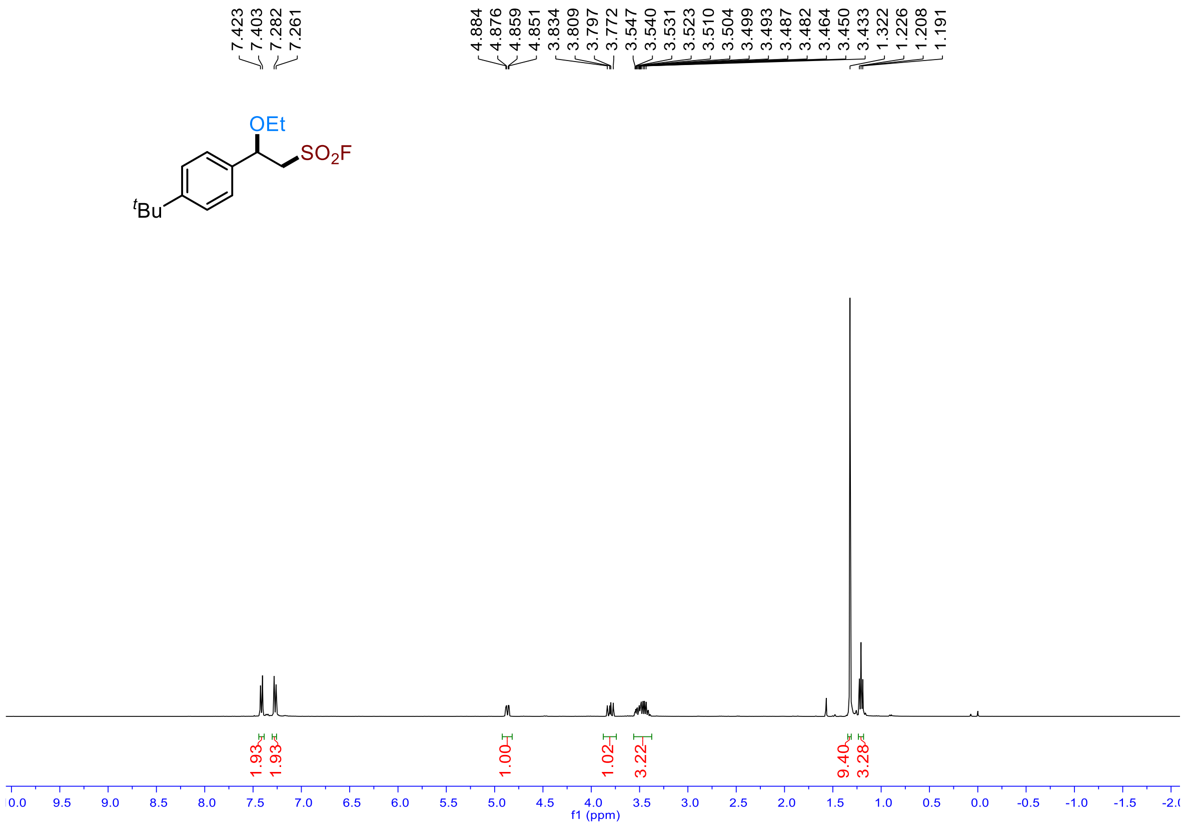

Supplementary Figure 179. ^{1}H NMR spectra of **7c**

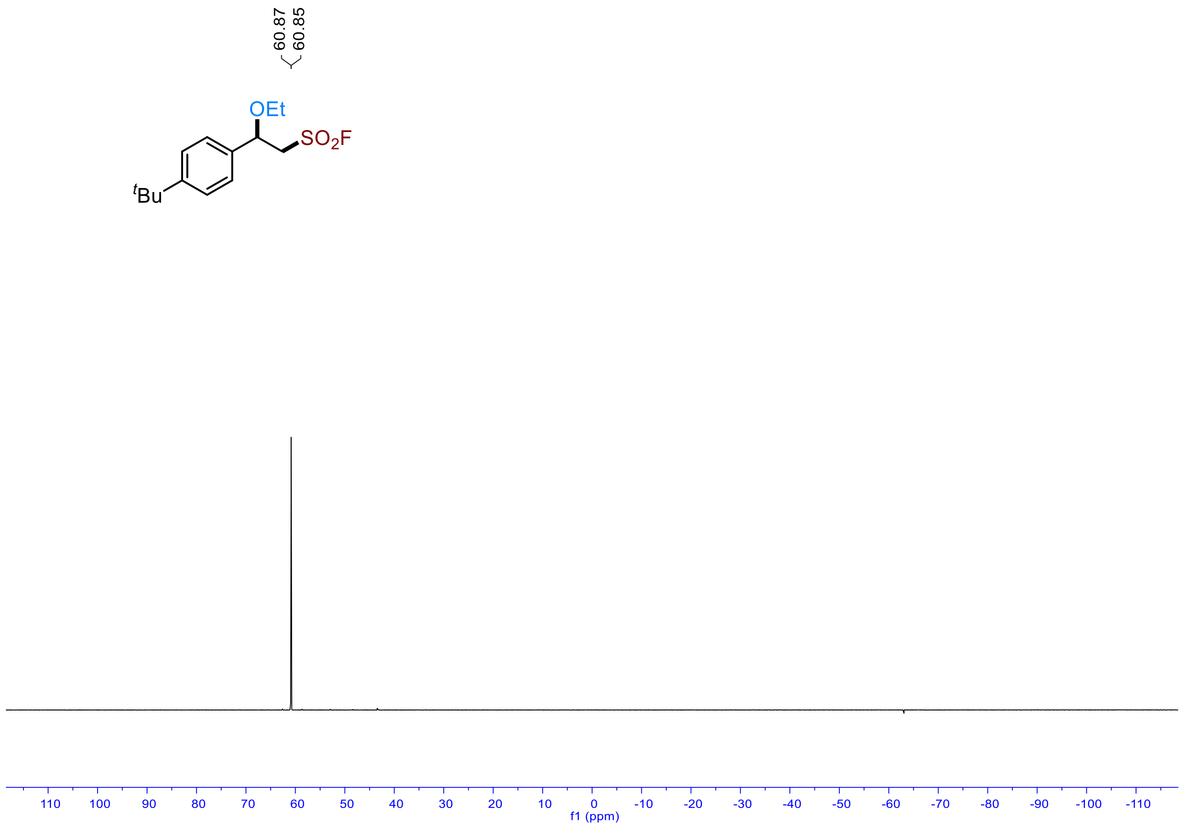

Supplementary Figure 180. ^{19}F NMR spectra of **7c**

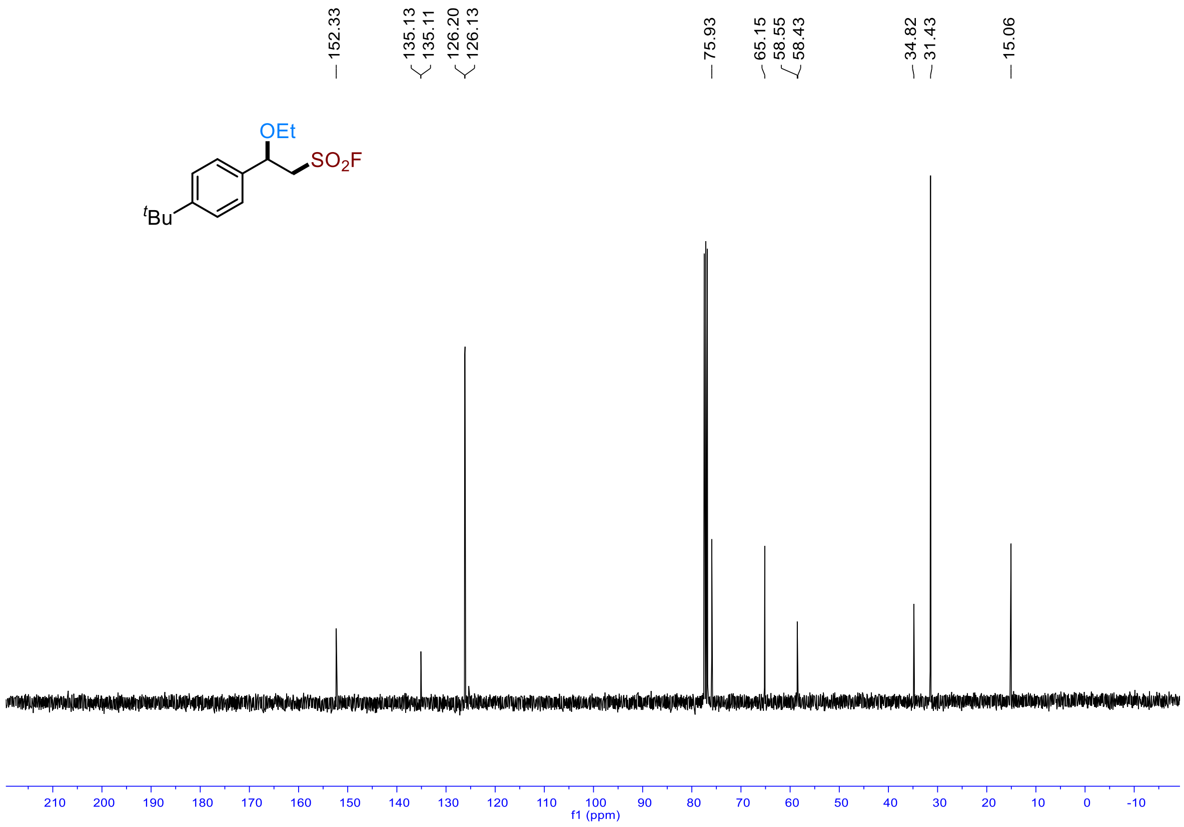

Supplementary Figure 181. ^{13}C NMR spectra of **7c**

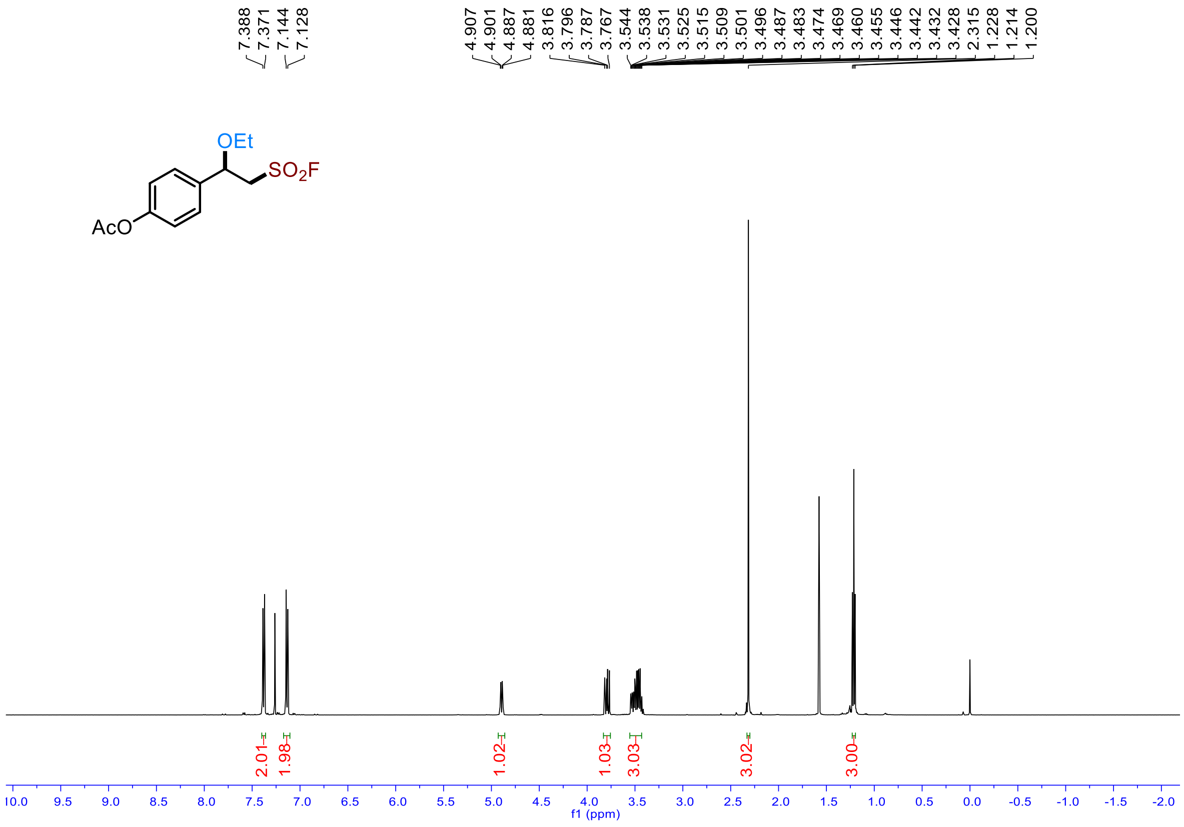

Supplementary Figure 182. ^{1}H NMR spectra of **7d**

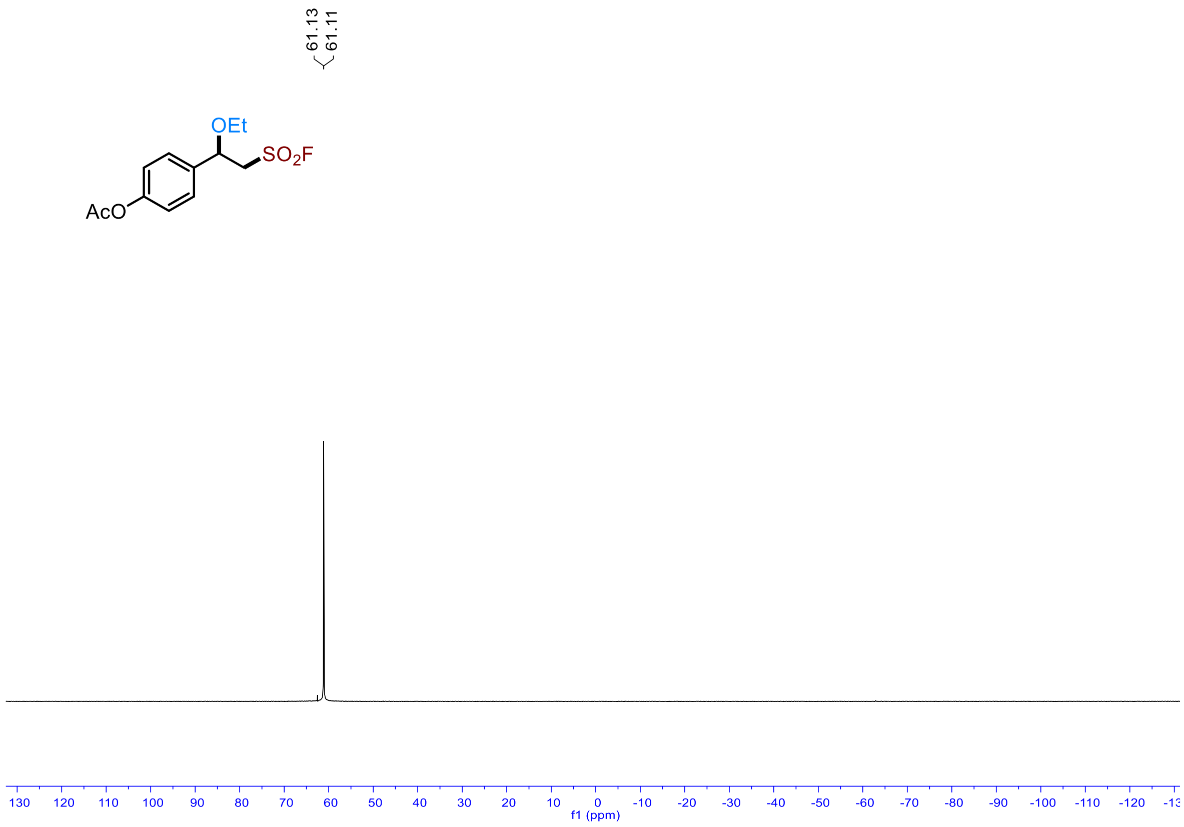

Supplementary Figure 183. ^{19}F NMR spectra of **7d**

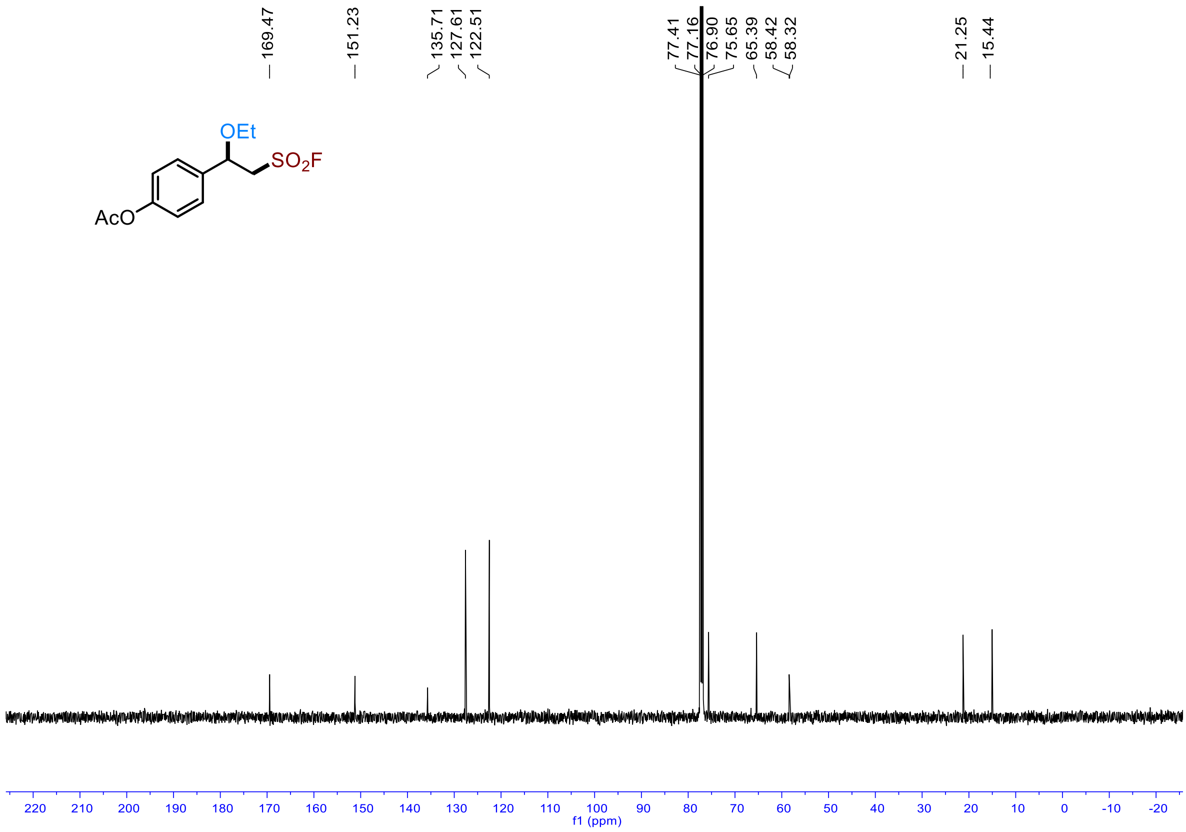

Supplementary Figure 184. ^{13}C NMR spectra of **7d**

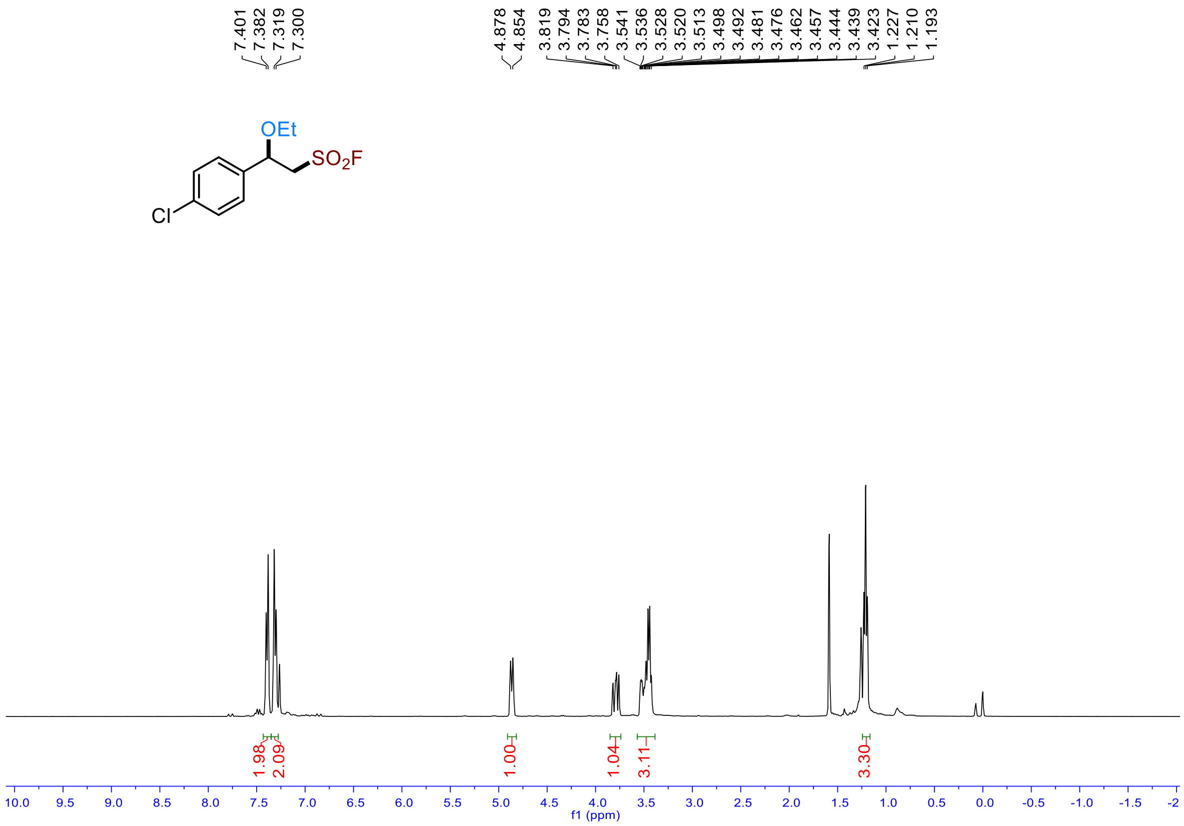

Supplementary Figure 185. ^{1}H NMR spectra of **7e**

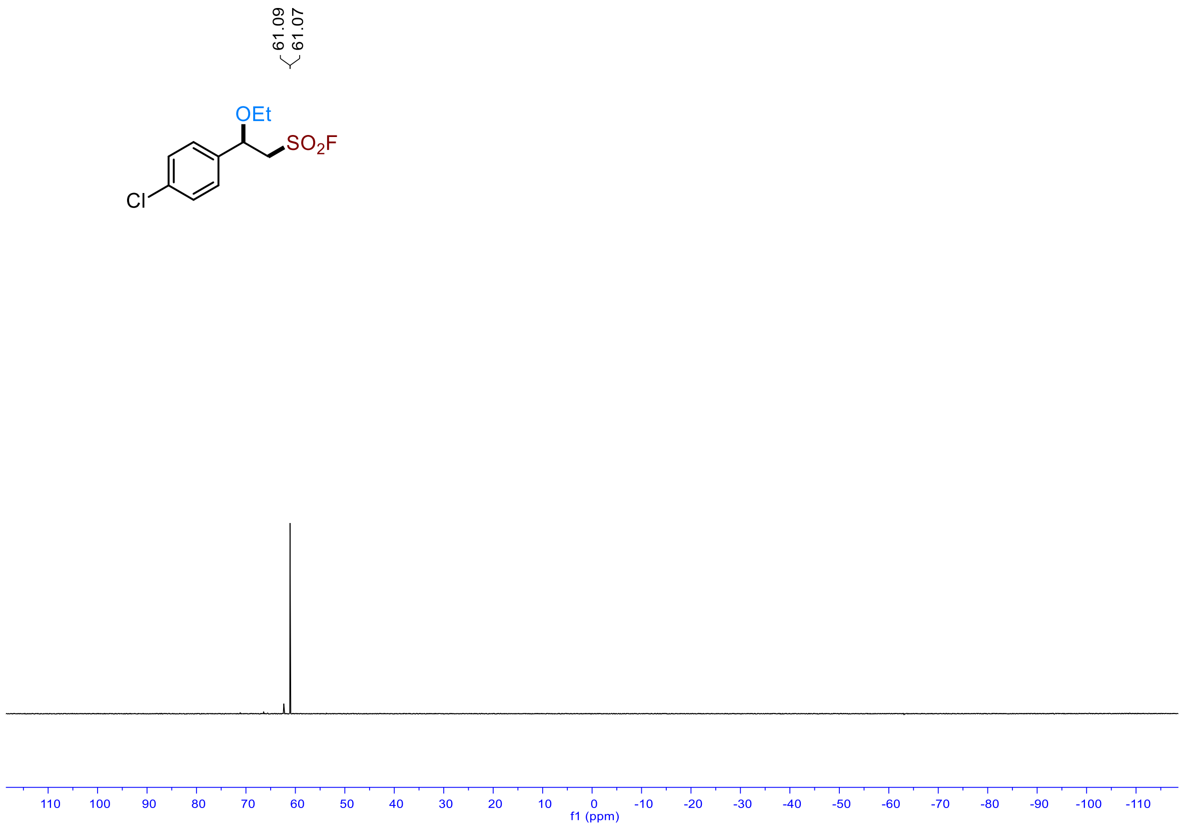

Supplementary Figure 186. ^{19}F NMR spectra of **7e**

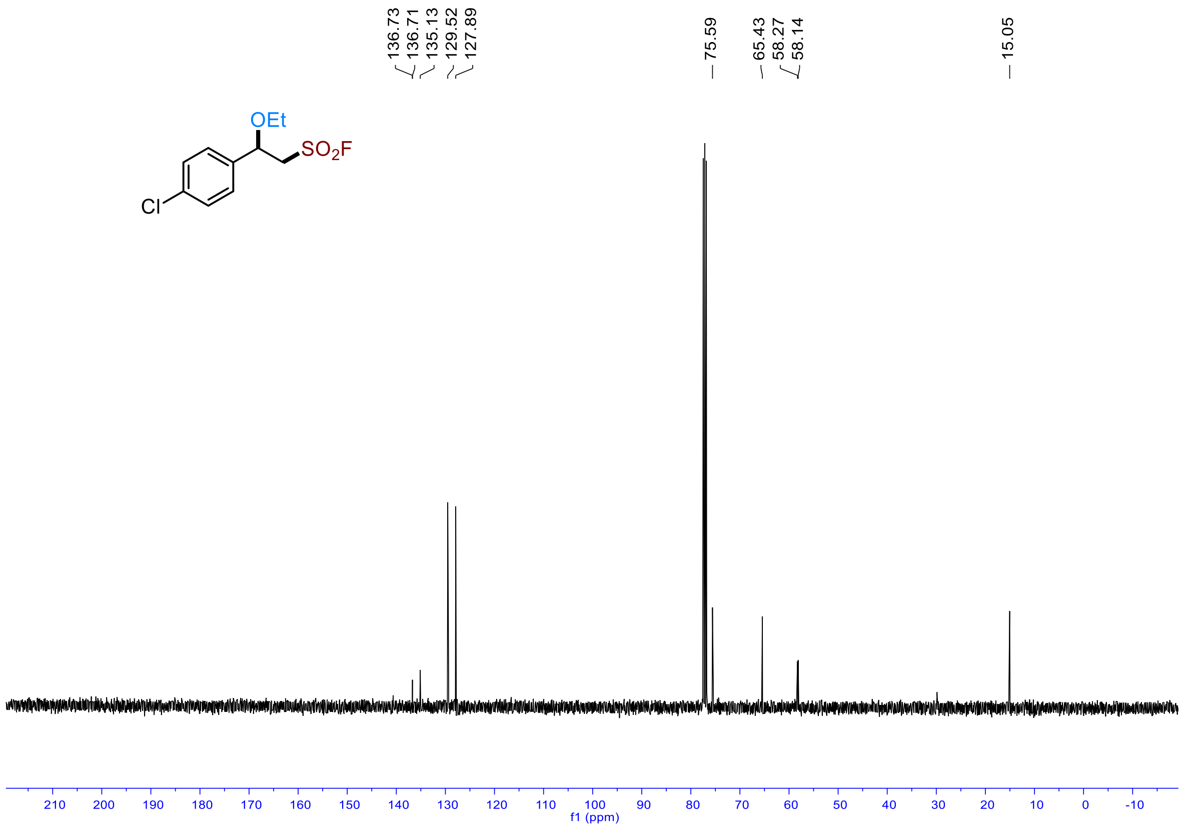

Supplementary Figure 187. ^{13}C NMR spectra of **7e**

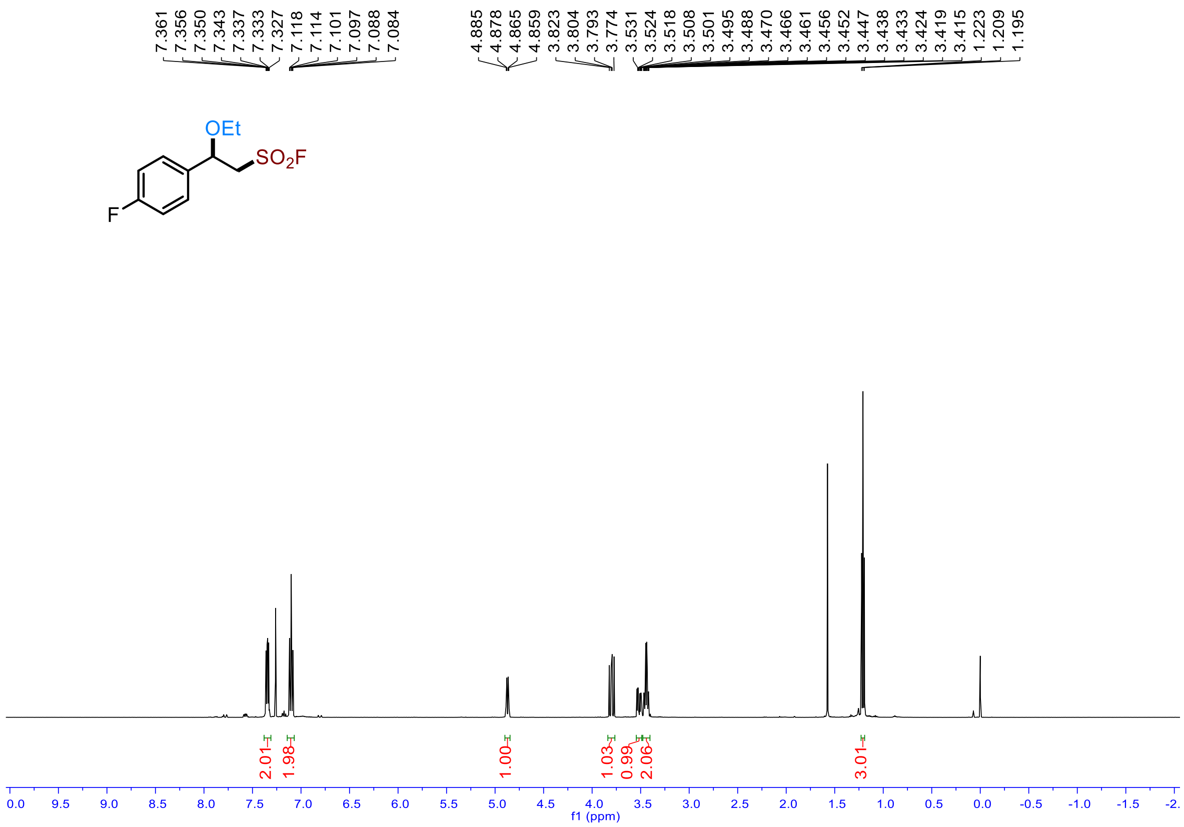

Supplementary Figure 188. ^{1}H NMR spectra of **7f**

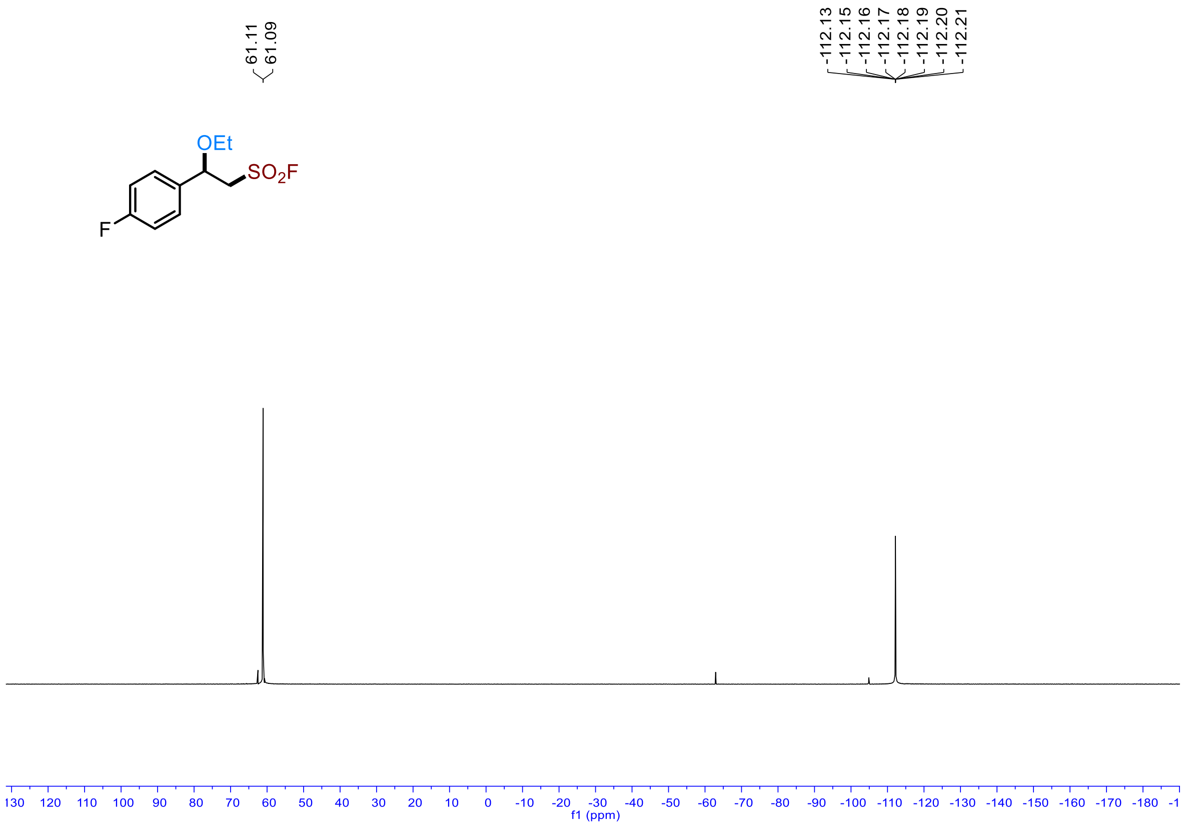

Supplementary Figure 189. ^{19}F NMR spectra of **7f**

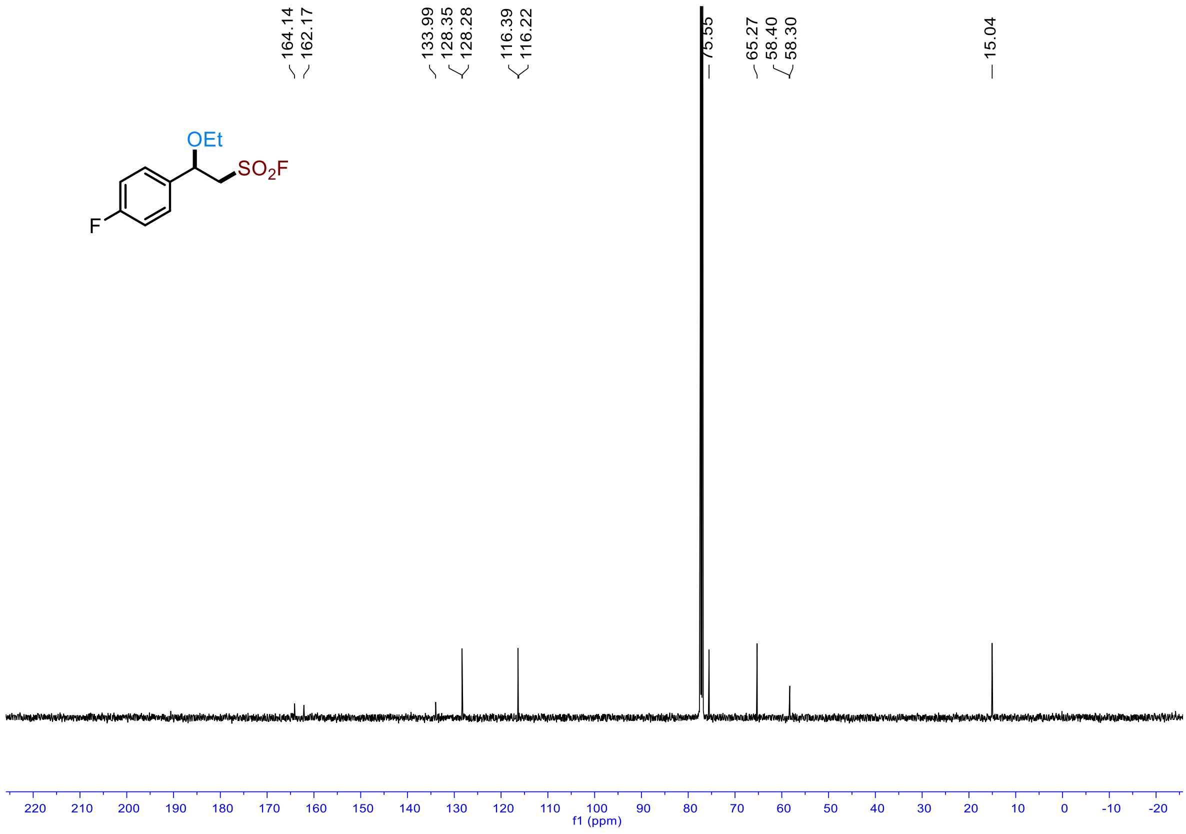

Supplementary Figure 190. ^{13}C NMR spectra of **7f**

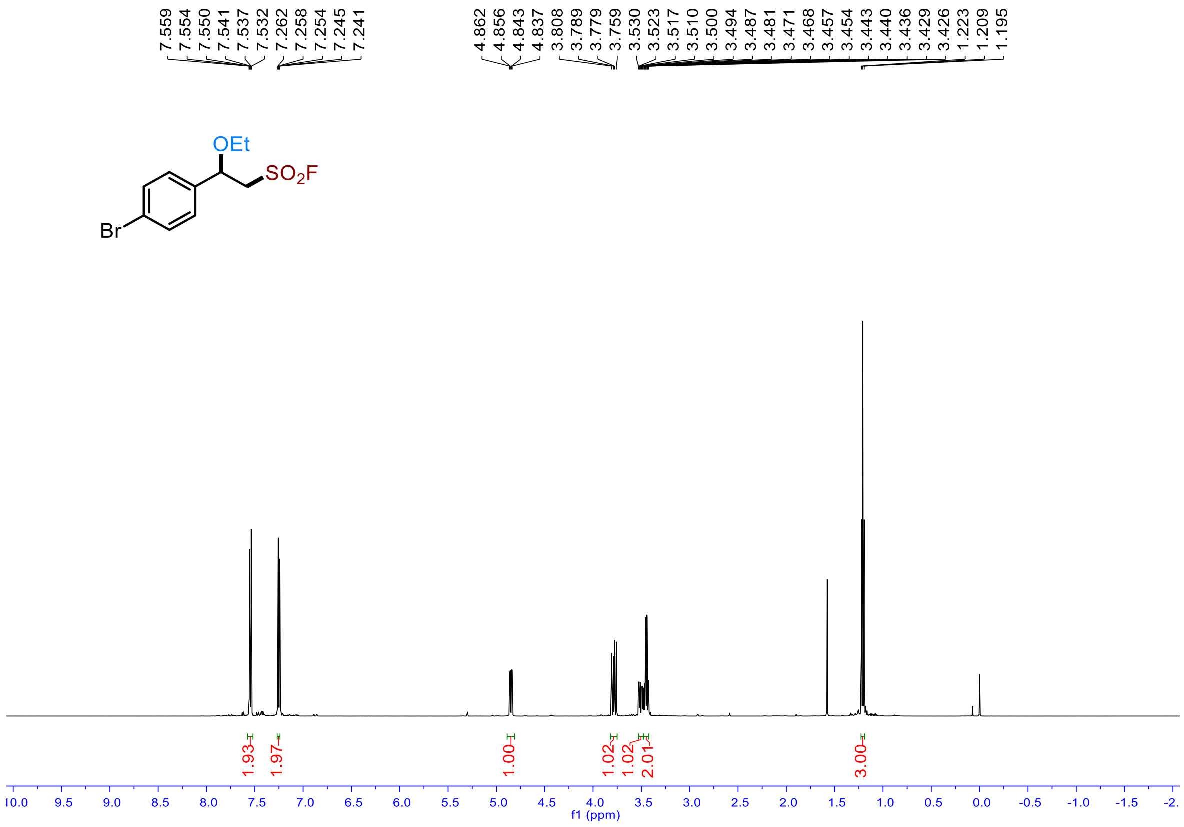

Supplementary Figure 191. ^{1}H NMR spectra of **7g**

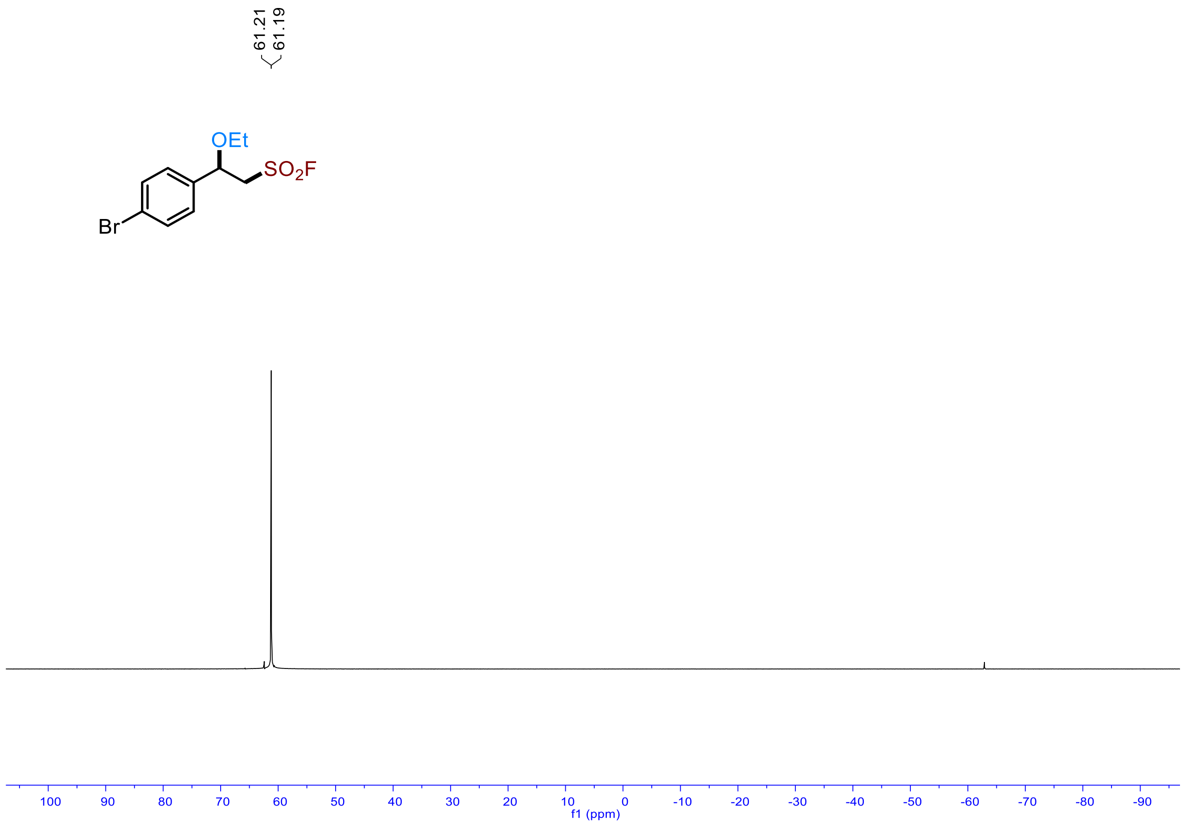

Supplementary Figure 192. ^{19}F NMR spectra of **7g**

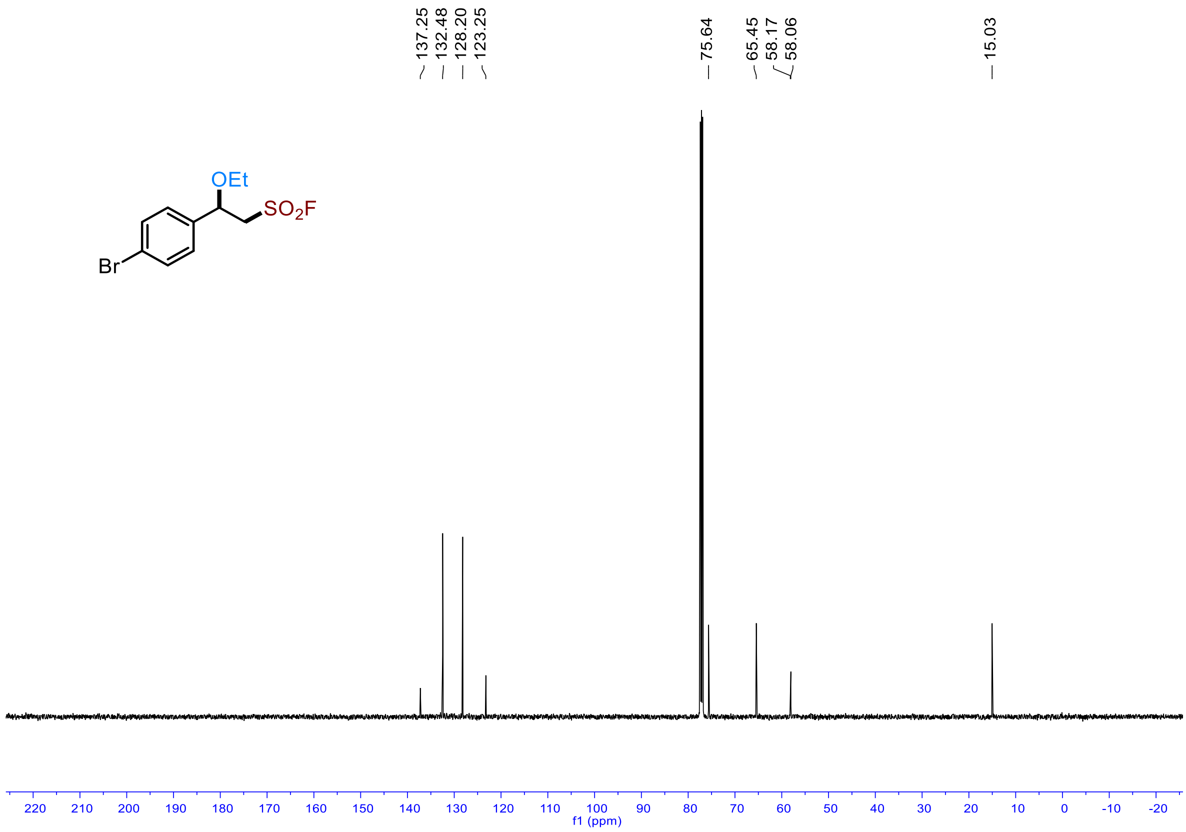

Supplementary Figure 193. ^{13}C NMR spectra of **7g**

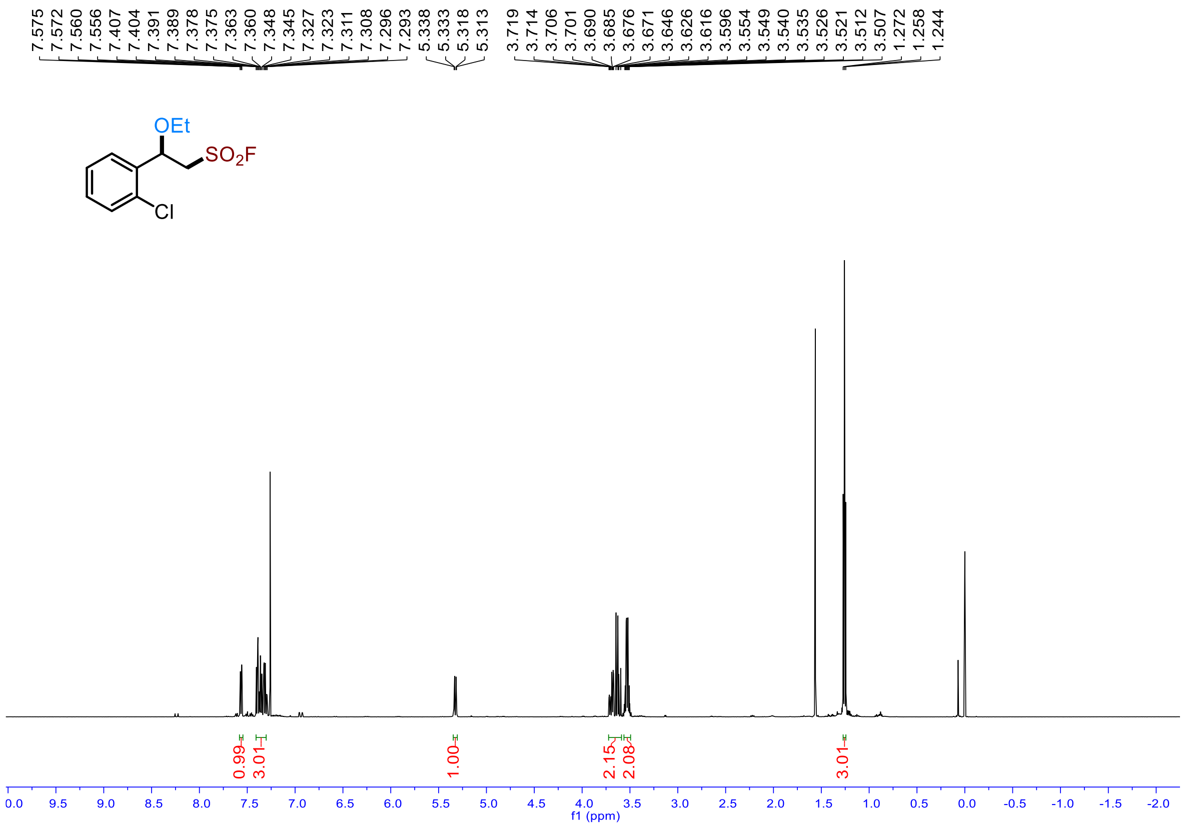

Supplementary Figure 194. ^{1}H NMR spectra of **7h**

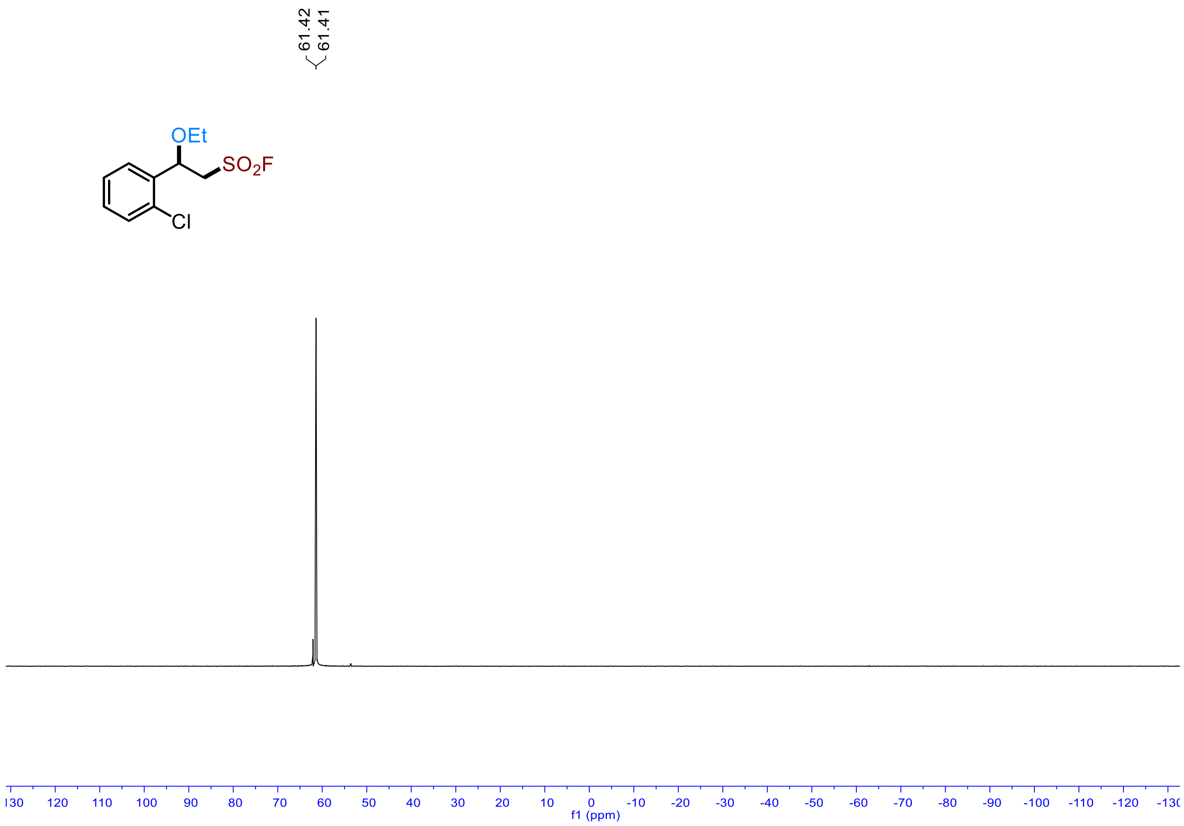

Supplementary Figure 195. ^{19}F NMR spectra of **7h**

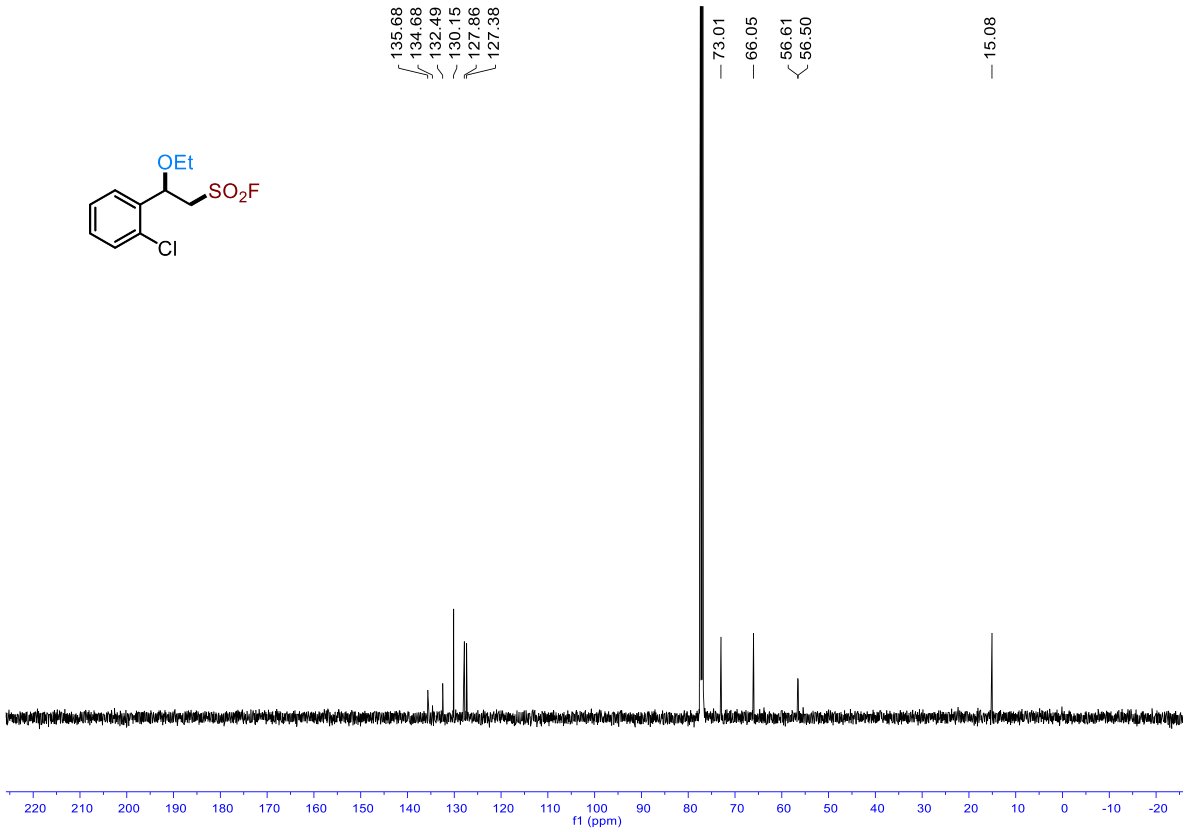

Supplementary Figure 196. ^{13}C NMR spectra of **7h**

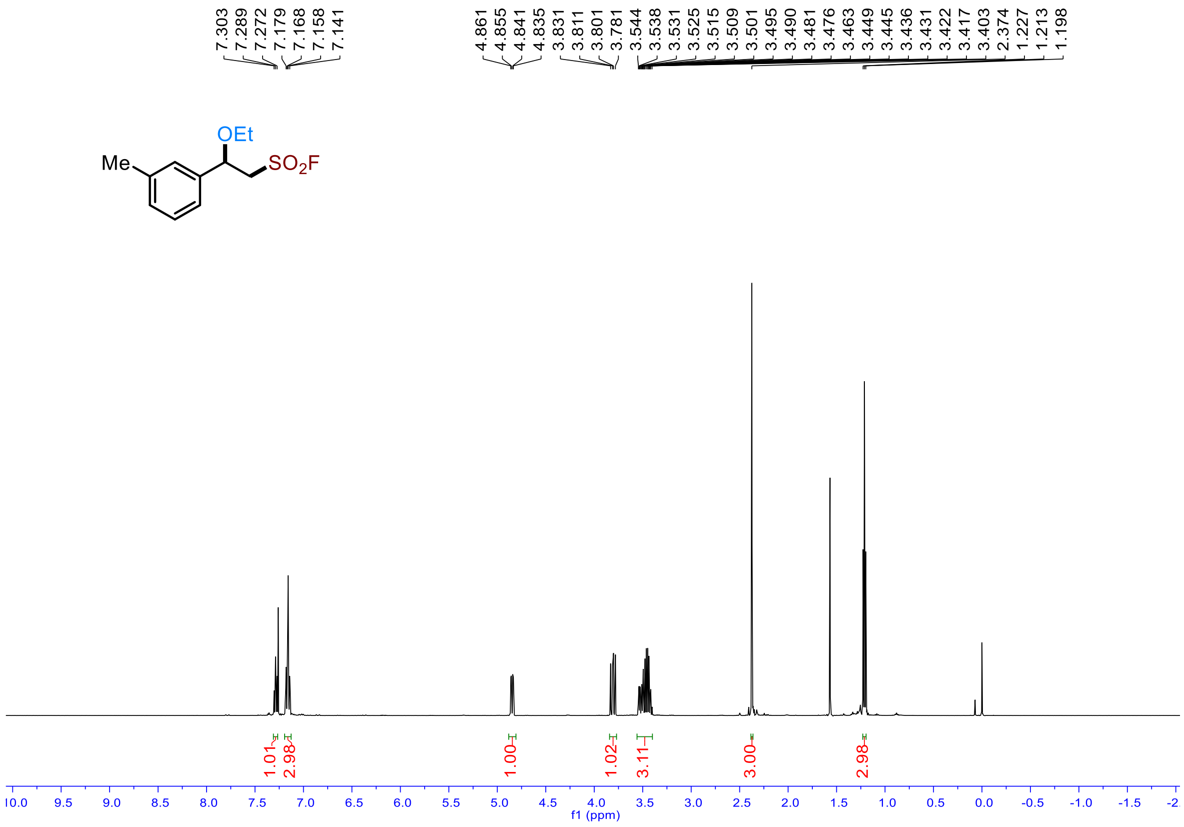

Supplementary Figure 197. ^{1}H NMR spectra of **7i**

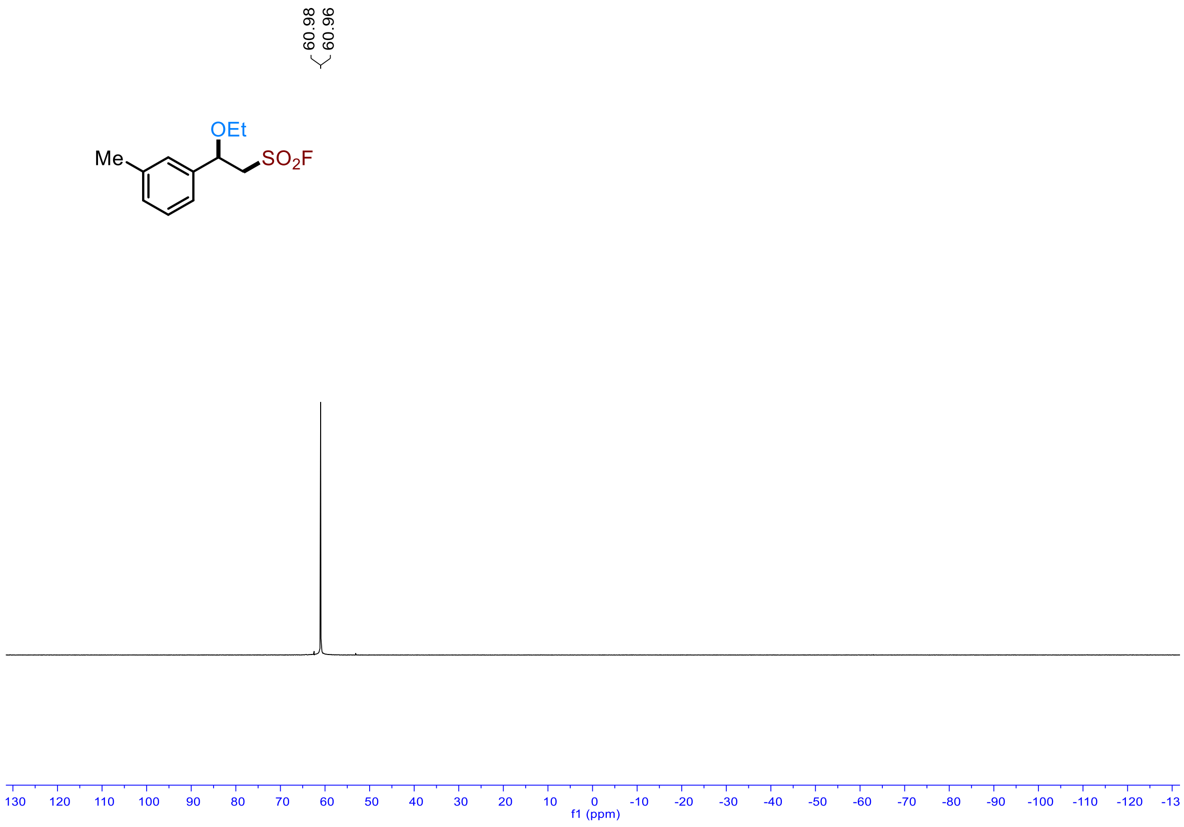

Supplementary Figure 198. ^{19}F NMR spectra of **7i**

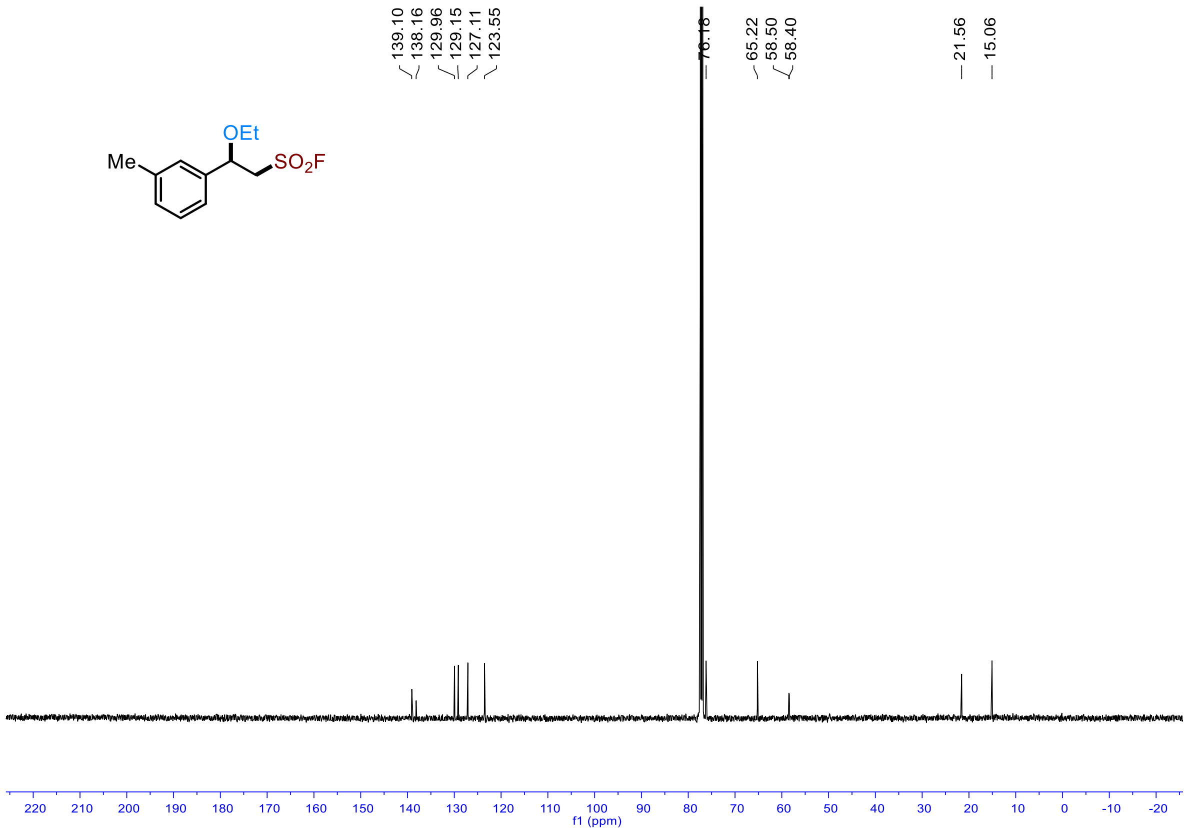

Supplementary Figure 199. ^{13}C NMR spectra of **7i**

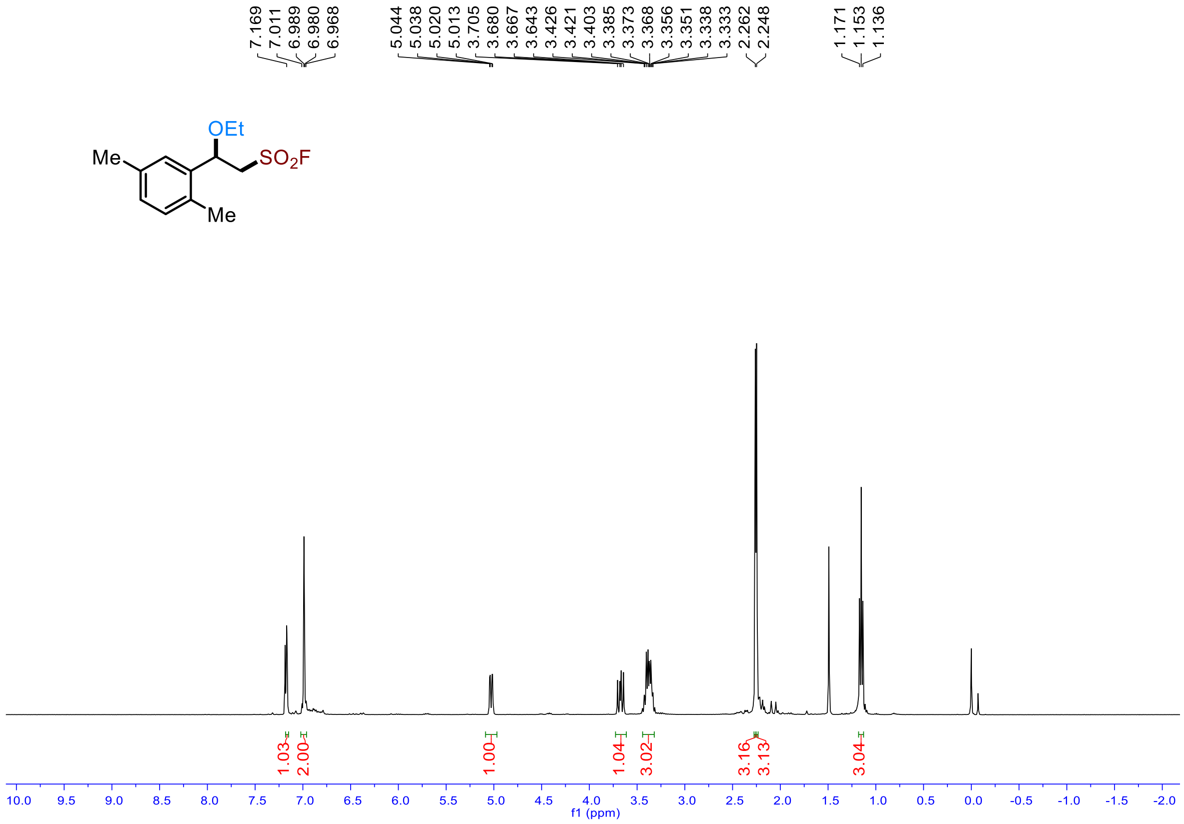

Supplementary Figure 200. ^{1}H NMR spectra of **7j**

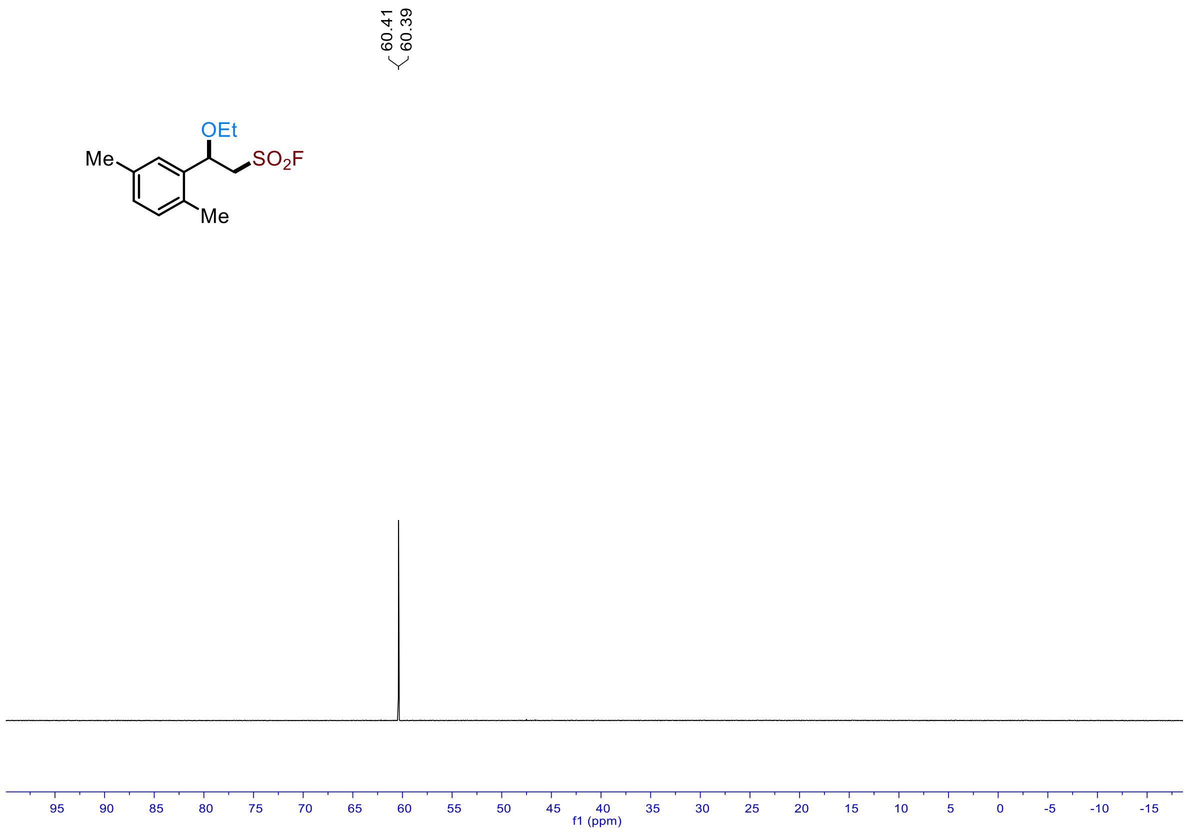

Supplementary Figure 201. ^{19}F NMR spectra of **7j**

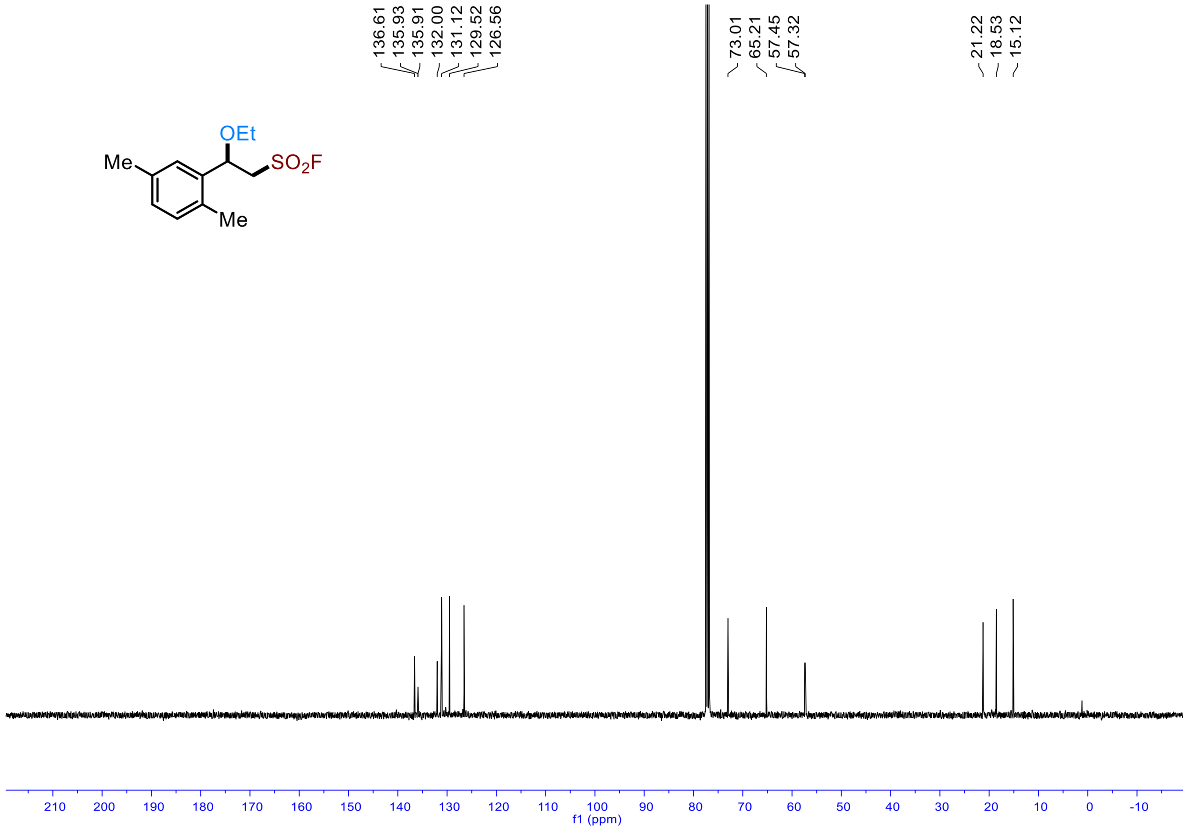

Supplementary Figure 202. ^{13}C NMR spectra of **7j**

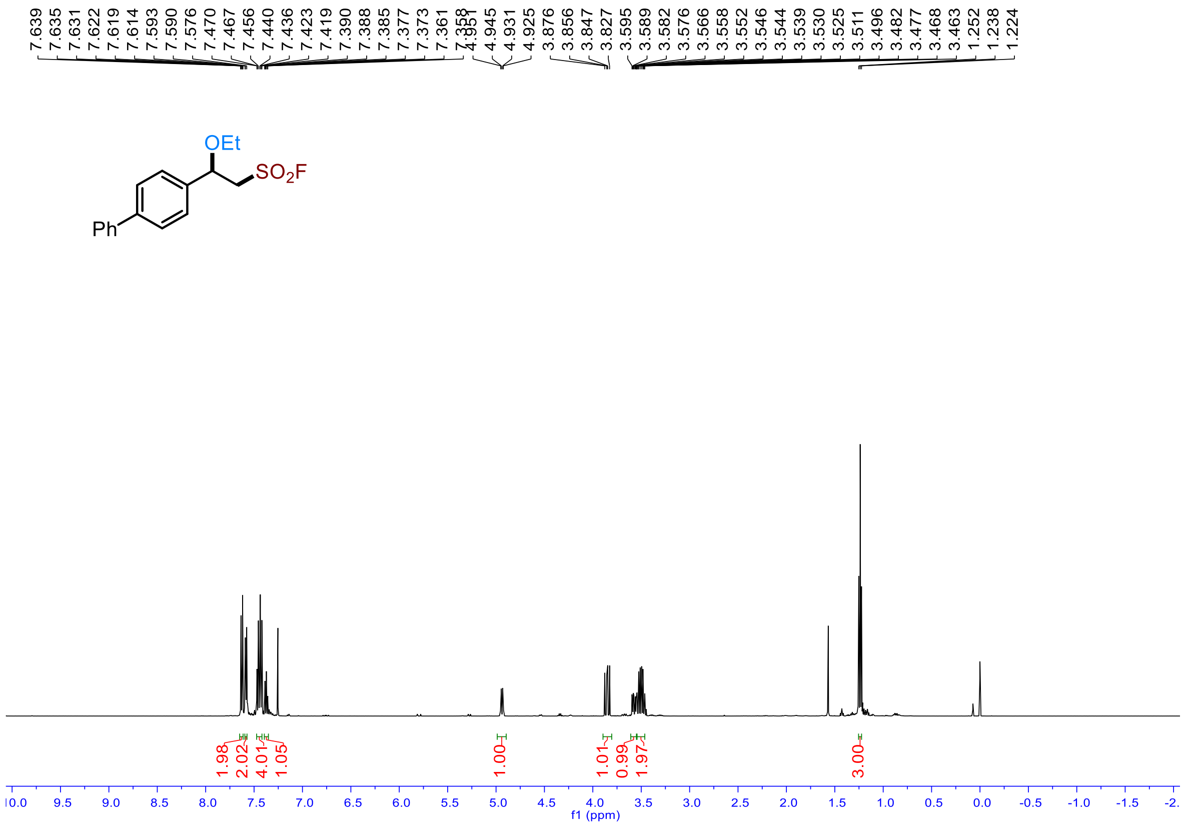

Supplementary Figure 203. ^{1}H NMR spectra of **7k**

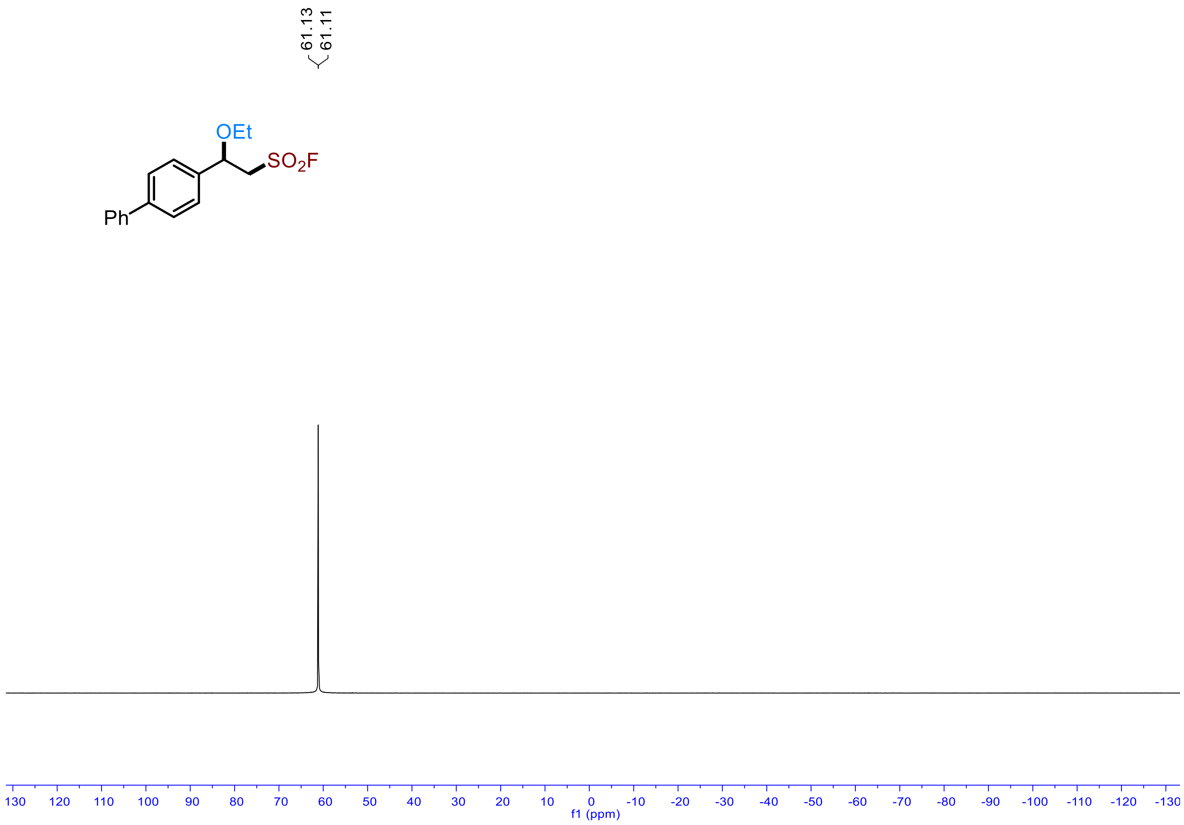

Supplementary Figure 204. ^{19}F NMR spectra of **7k**

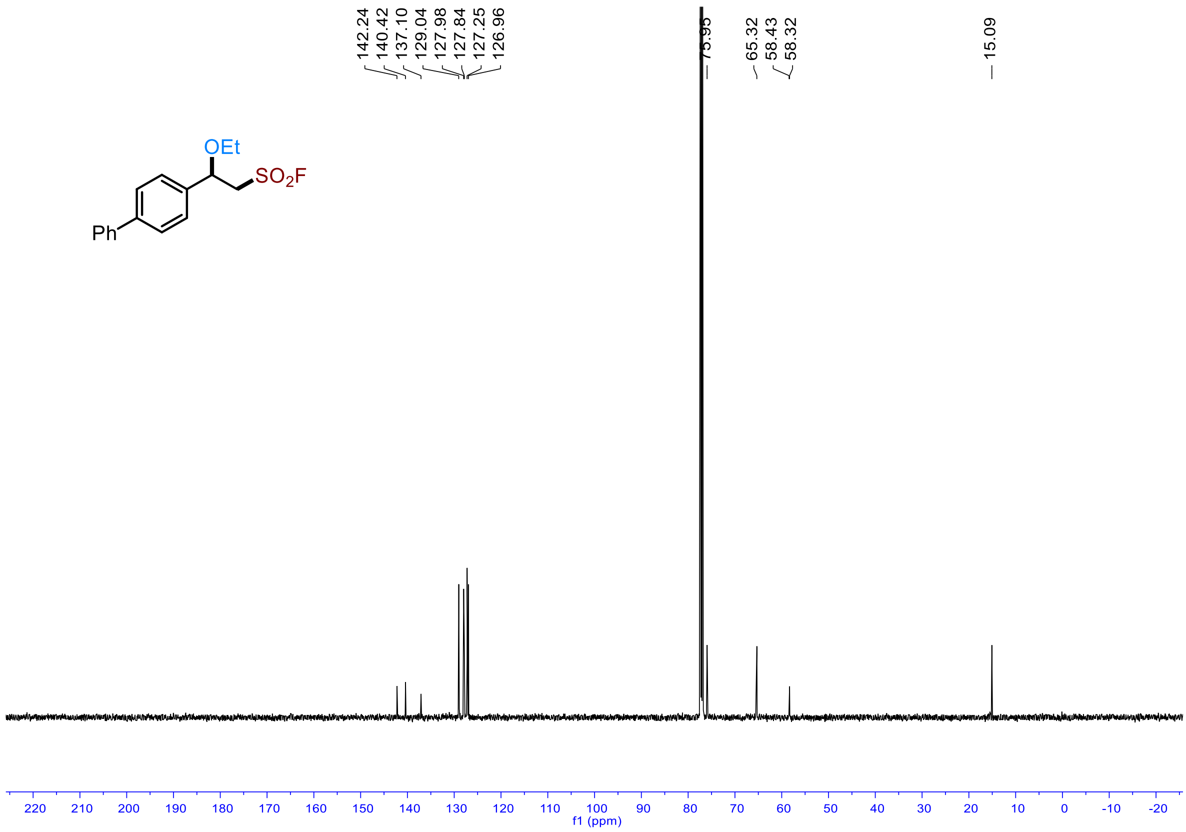

Supplementary Figure 205. ^{13}C NMR spectra of **7k**

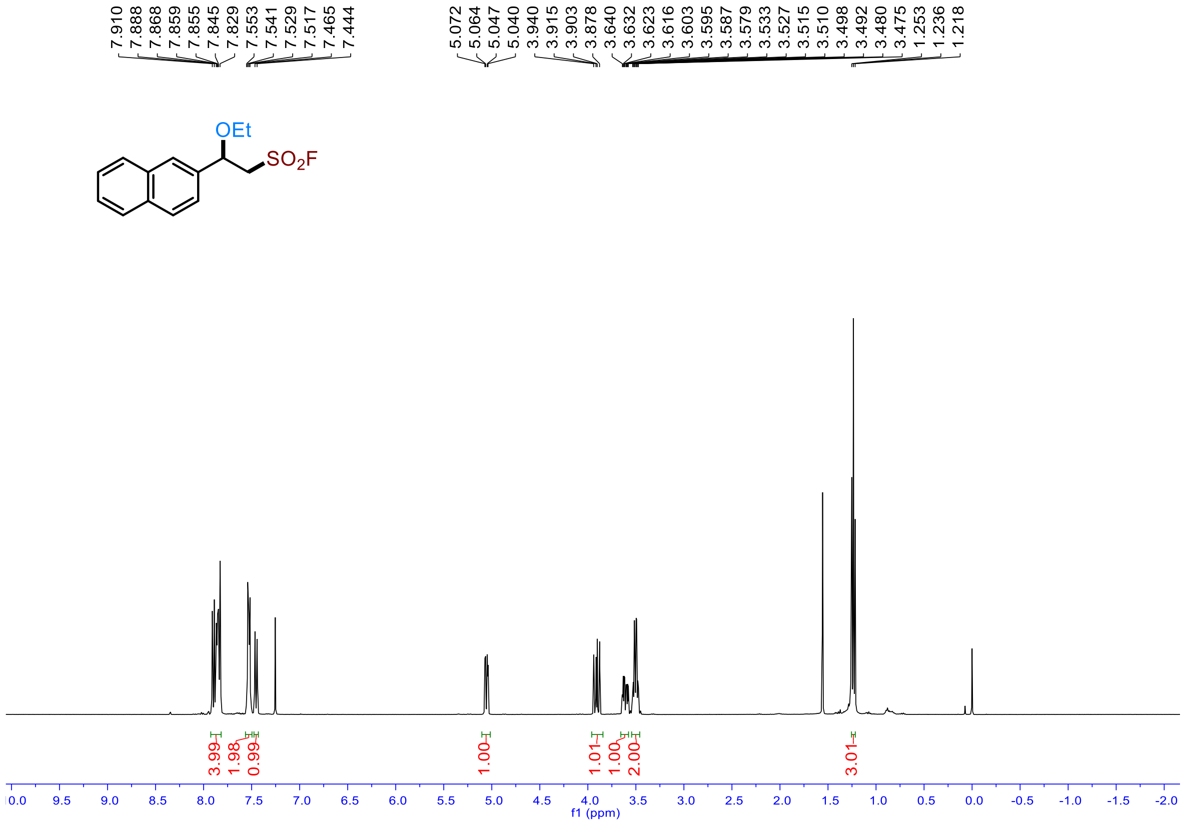

Supplementary Figure 206. ^{1}H NMR spectra of **7l**

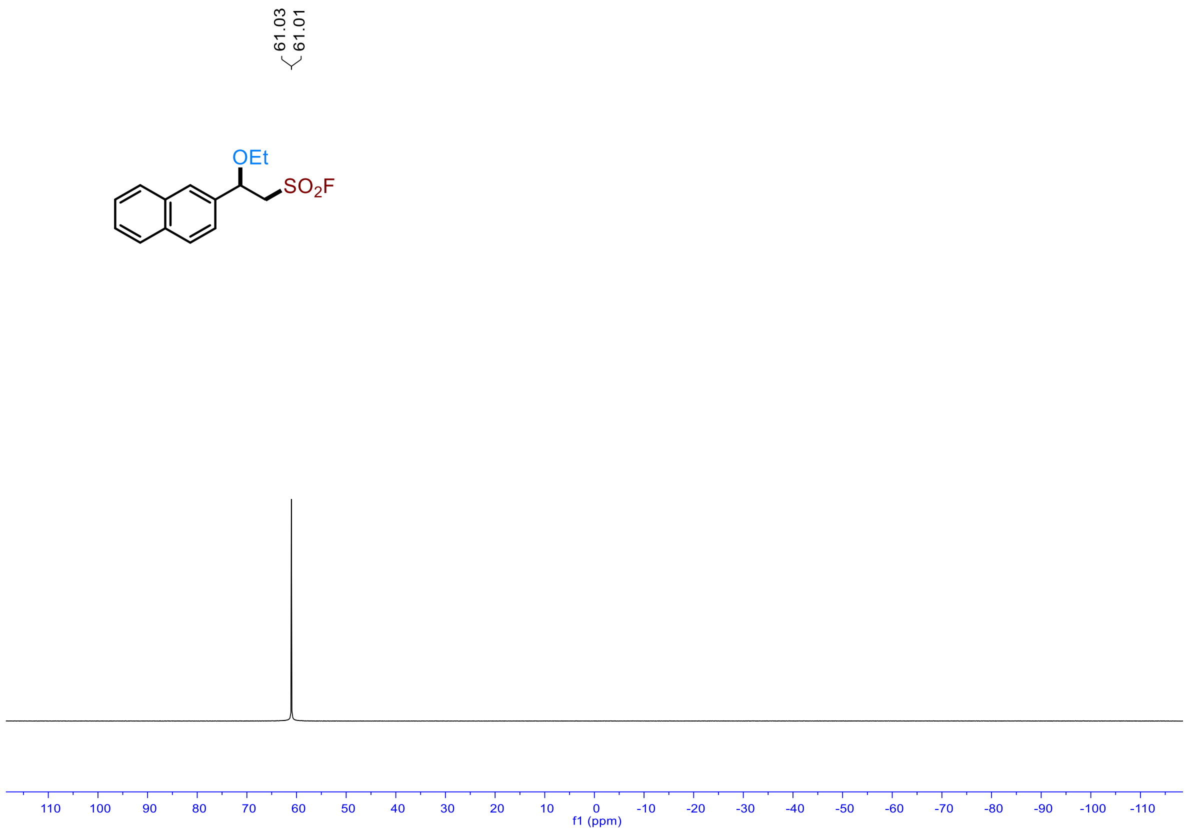

Supplementary Figure 207. ^{19}F NMR spectra of **7l**

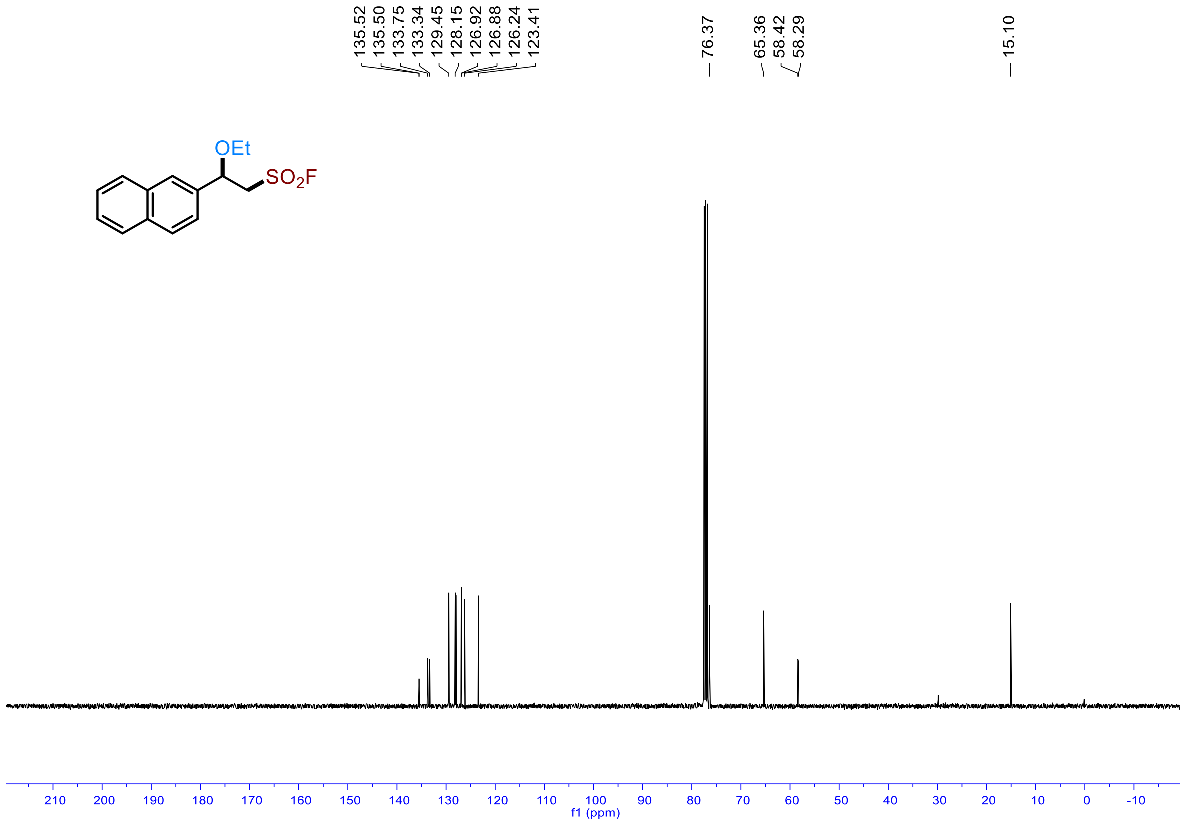

Supplementary Figure 208. ^{13}C NMR spectra of **7l**

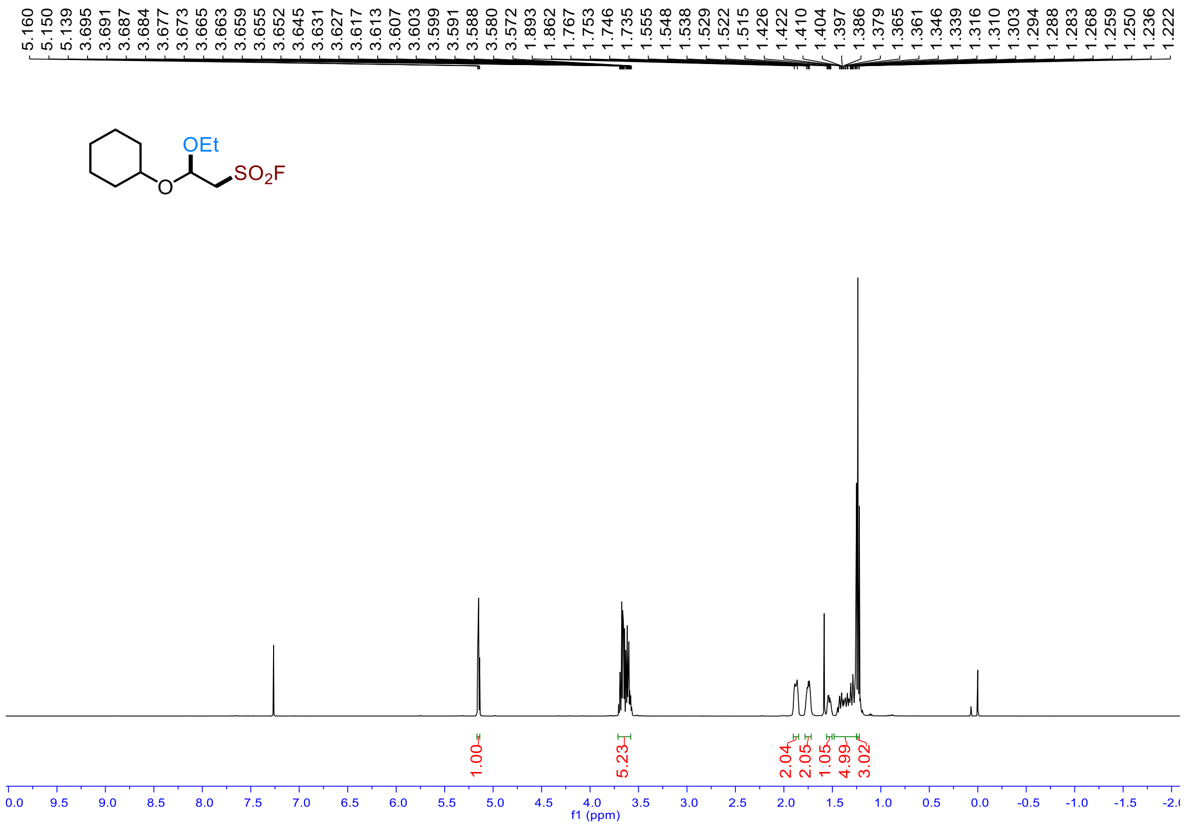

Supplementary Figure 209. ^{1}H NMR spectra of **7m**

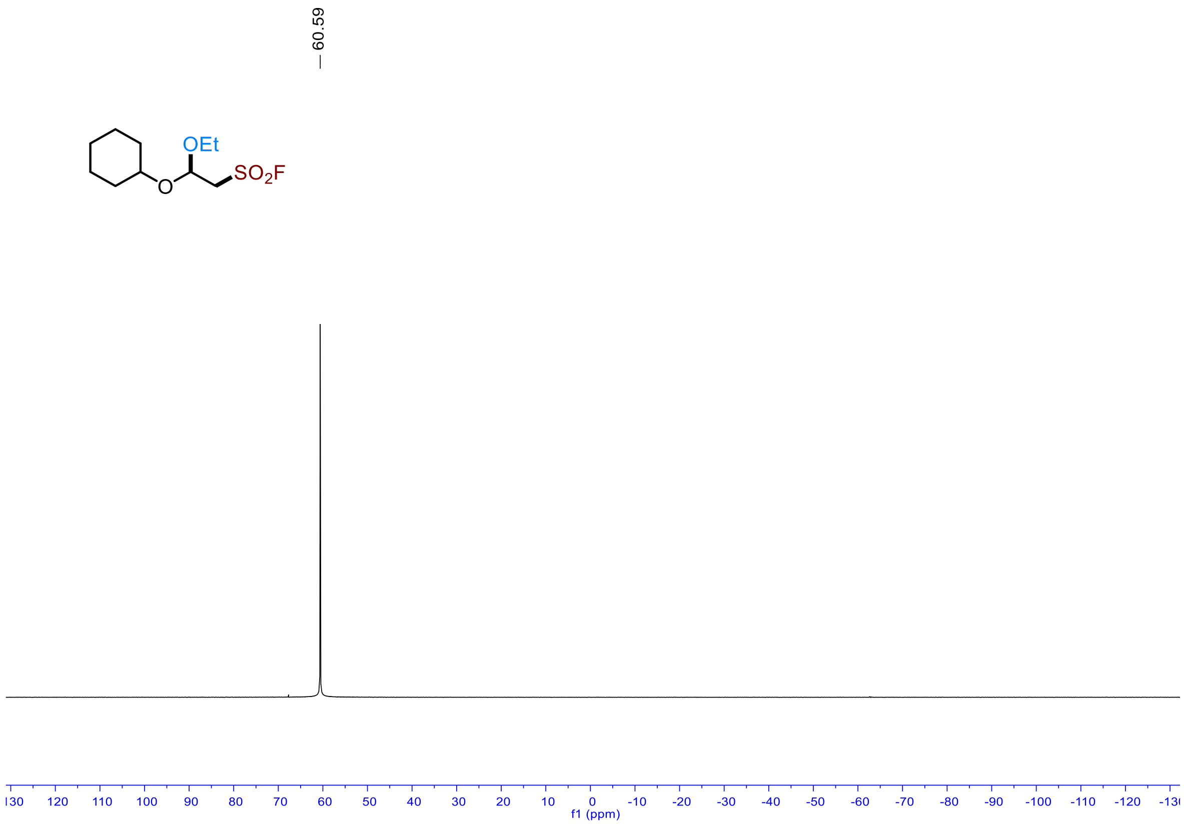

Supplementary Figure 210. ^{19}F NMR spectra of **7m**

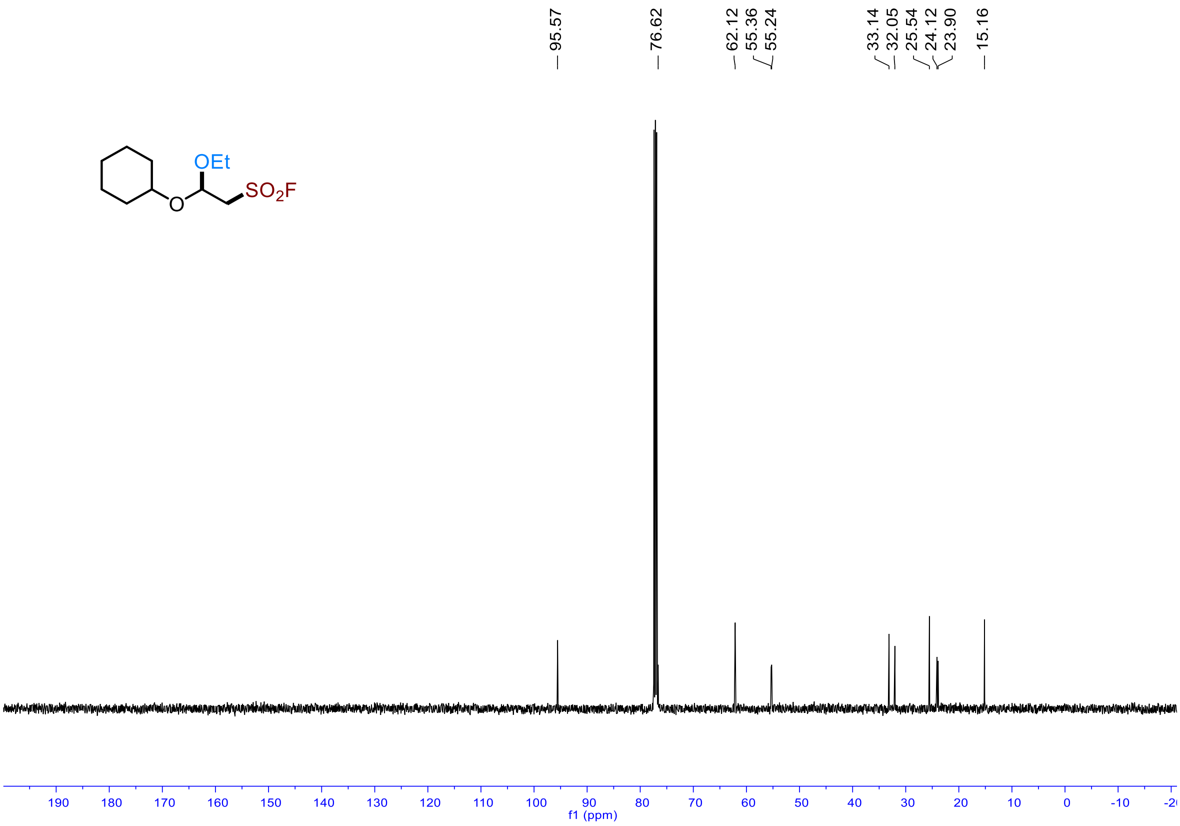

Supplementary Figure 211. ^{13}C NMR spectra of **7m**

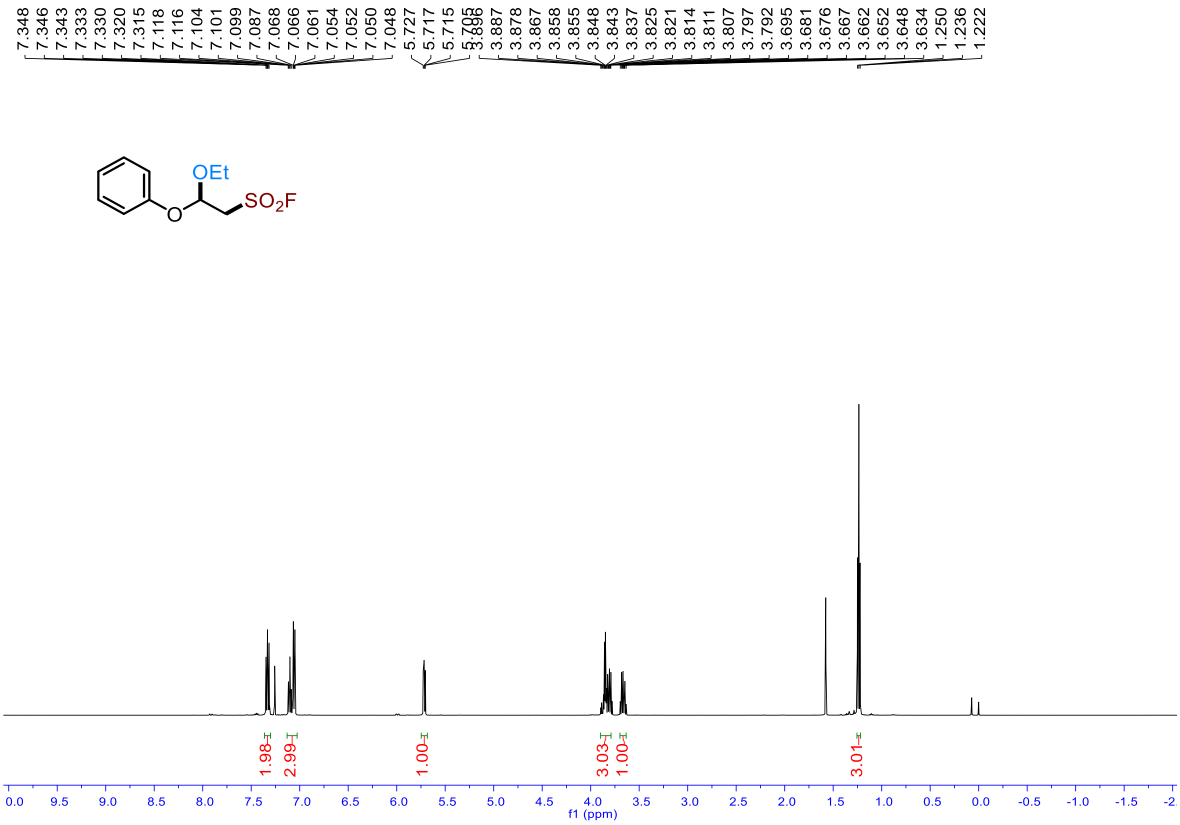

Supplementary Figure 212. ^{1}H NMR spectra of **7n**

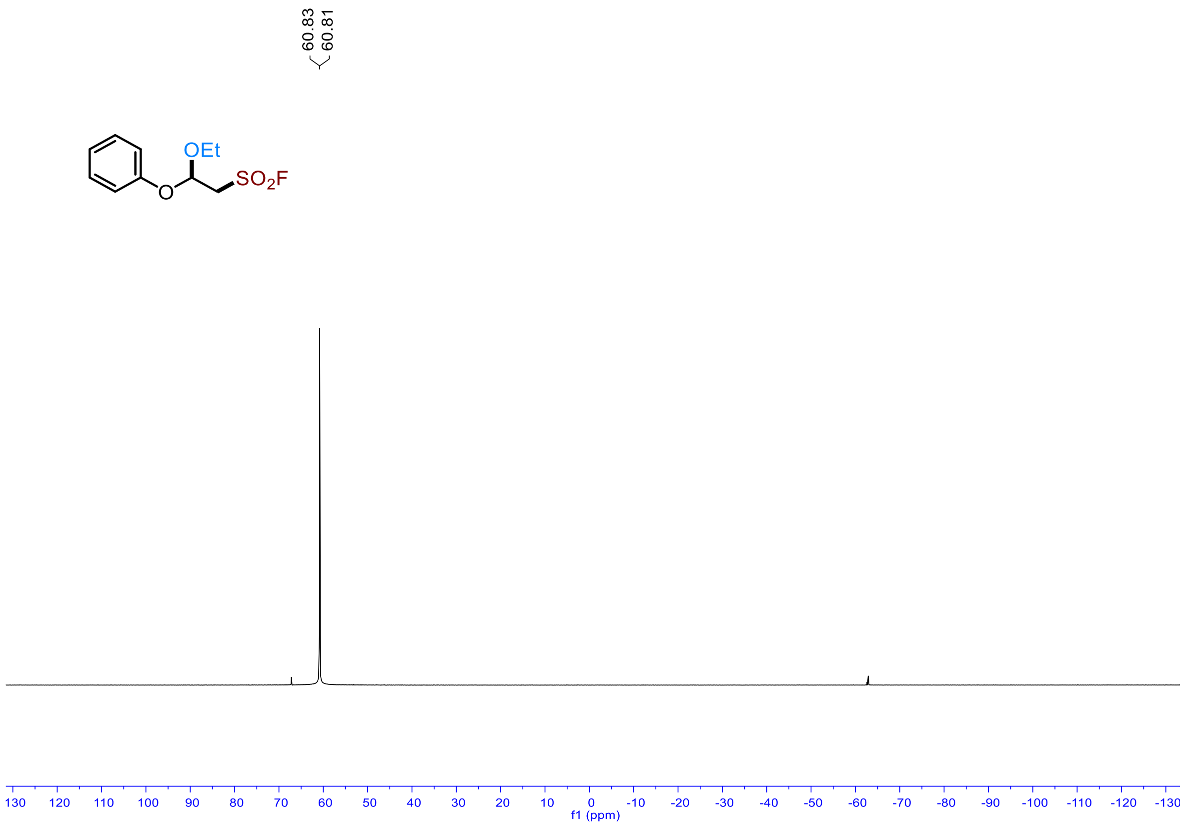

Supplementary Figure 213. ^{19}F NMR spectra of **7n**

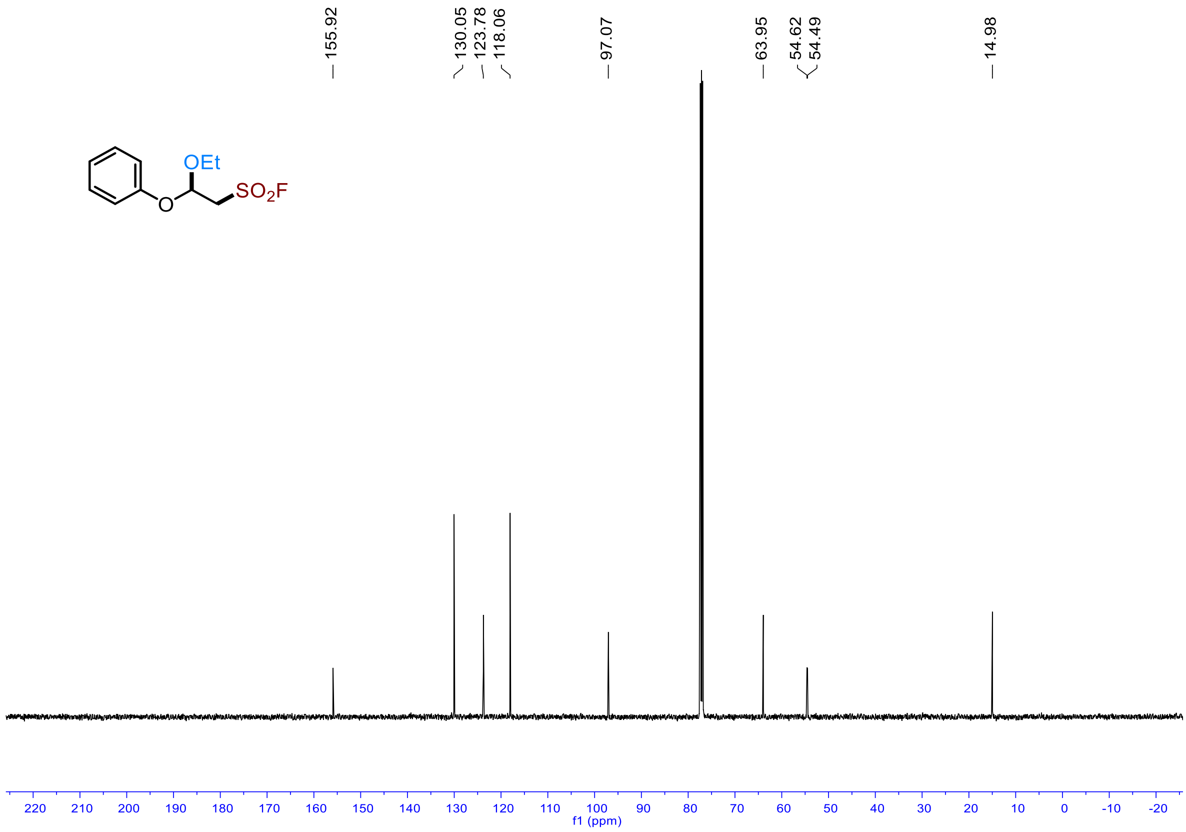

Supplementary Figure 214. ^{13}C NMR spectra of **7n**

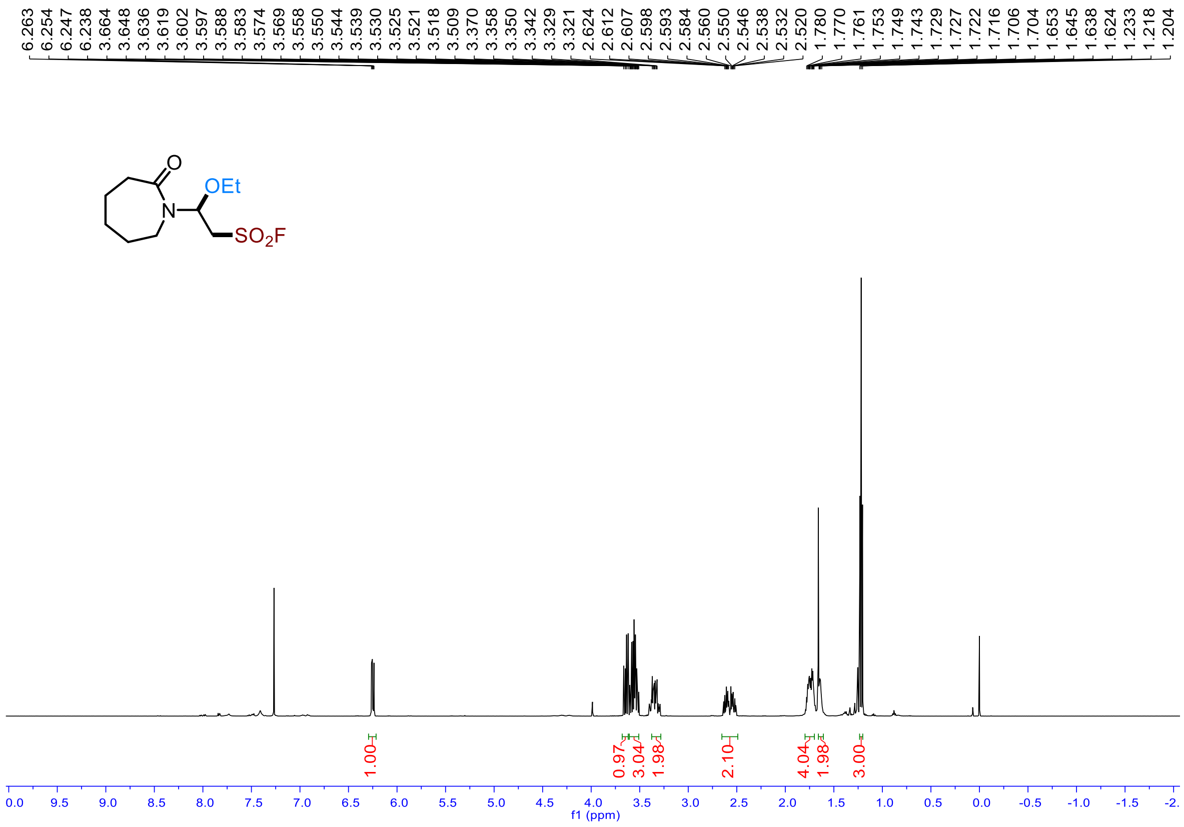

Supplementary Figure 215. ^{1}H NMR spectra of **7o**

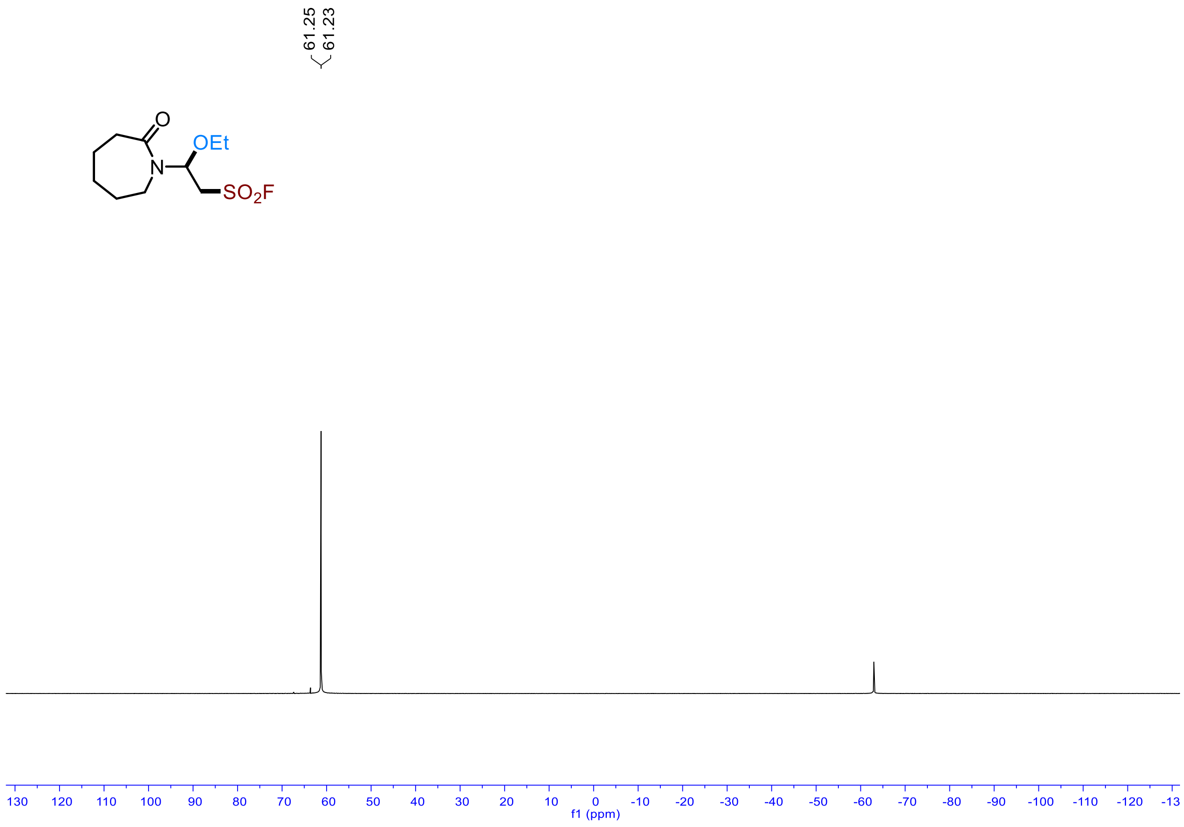

Supplementary Figure 216. ^{19}F NMR spectra of **7o**

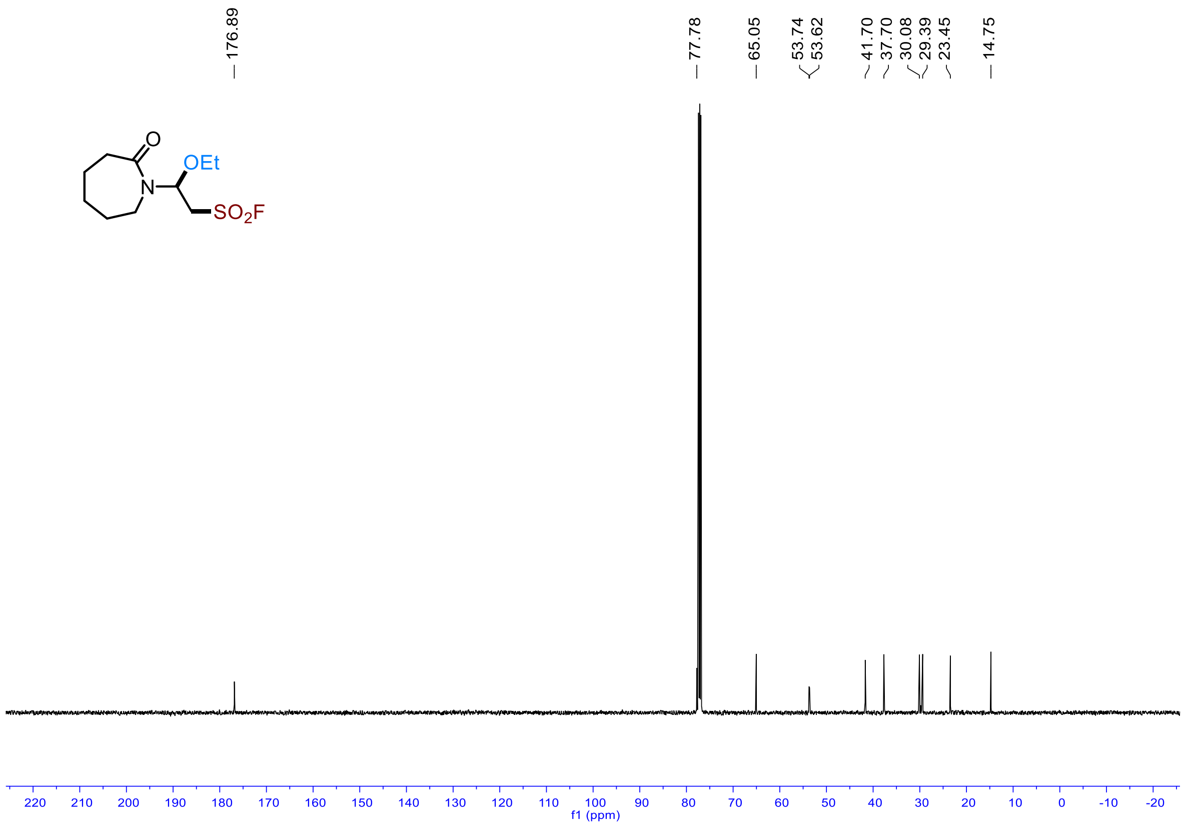

Supplementary Figure 217. ^{13}C NMR spectra of **7o**

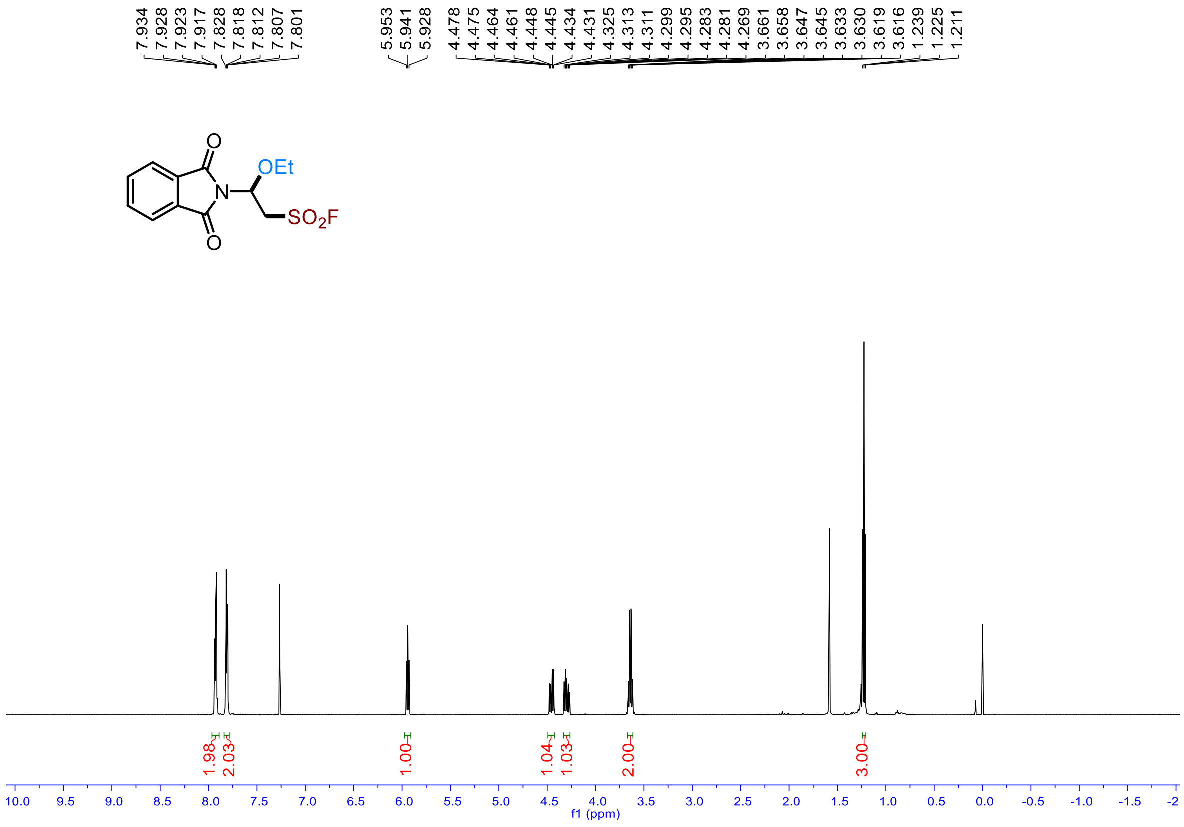

Supplementary Figure 218. ^{1}H NMR spectra of **7p**

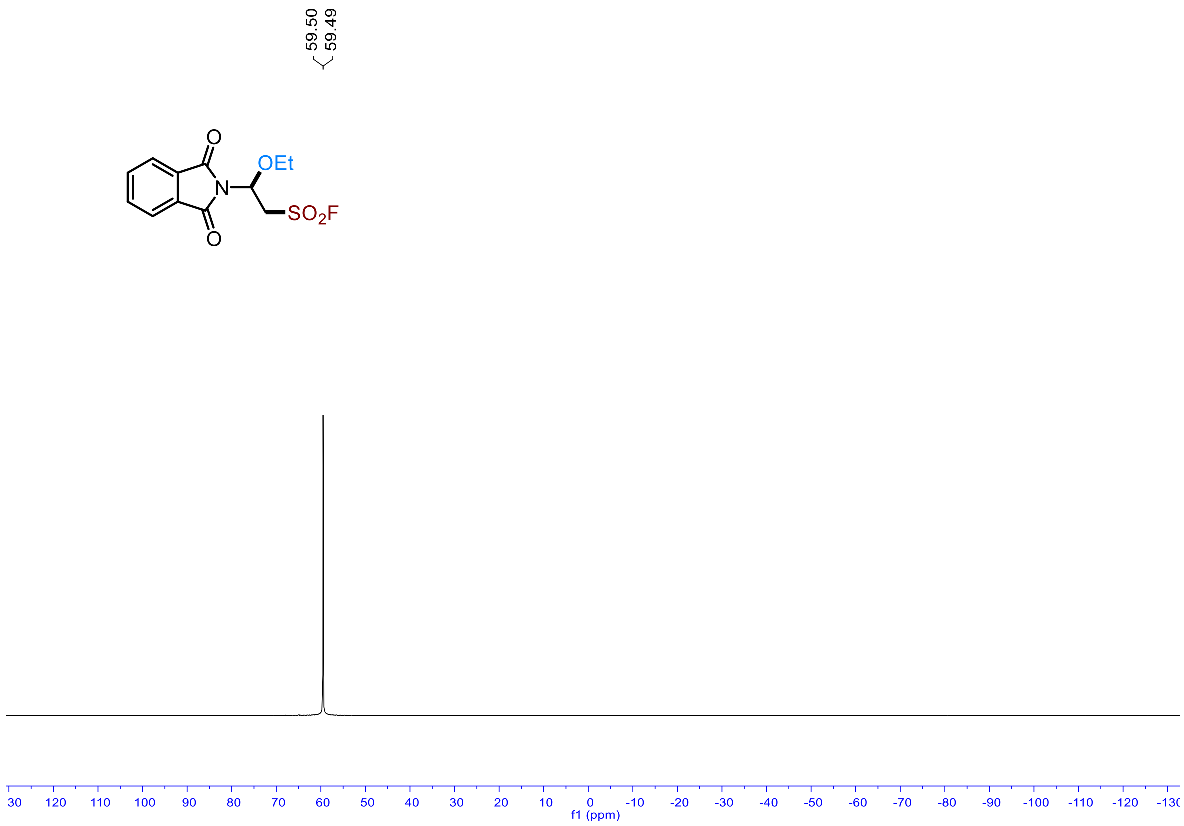

Supplementary Figure 219. ^{19}F NMR spectra of **7p**

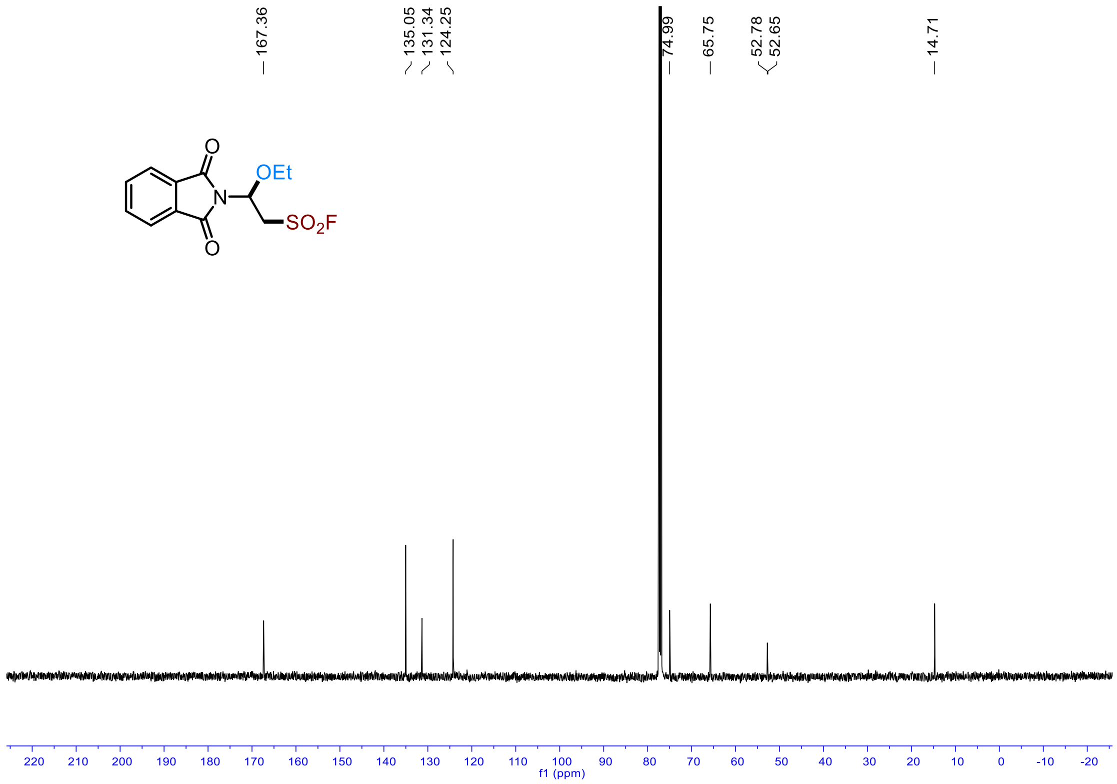

Supplementary Figure 220. ^{13}C NMR spectra of **7p**

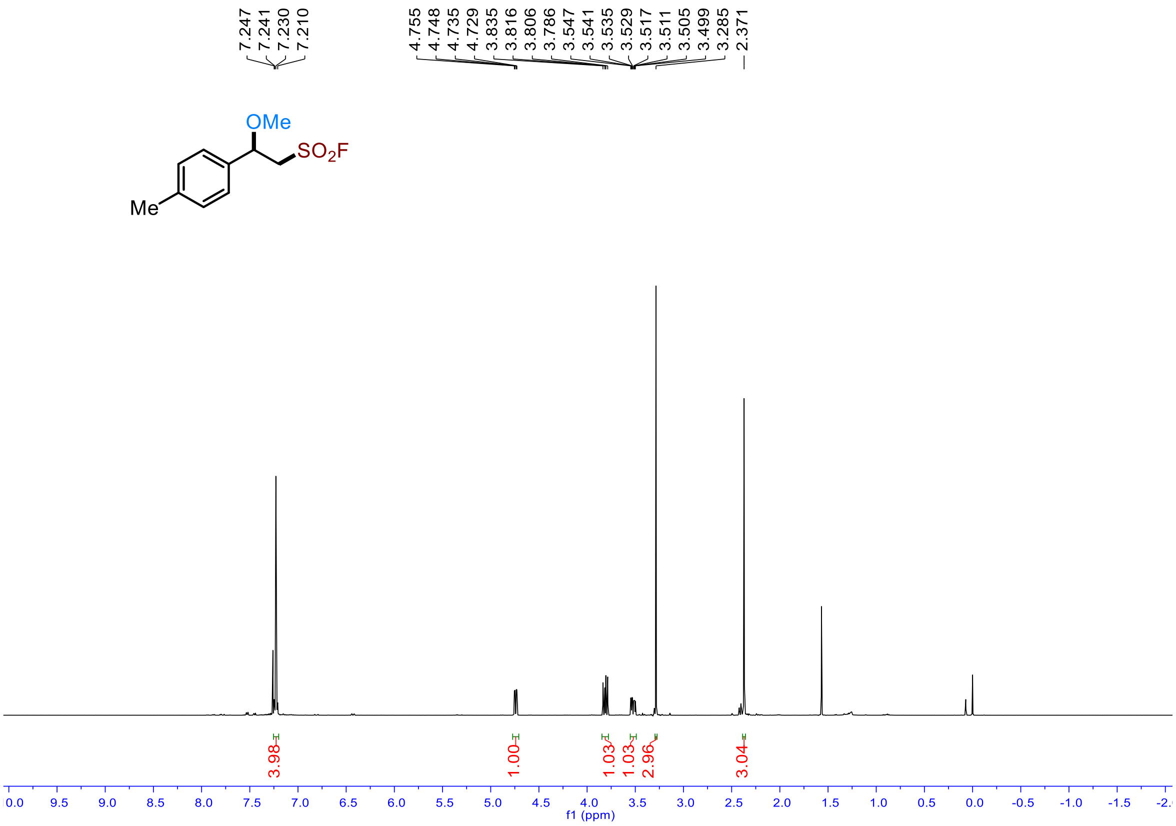

Supplementary Figure 221. ^{1}H NMR spectra of **7q**

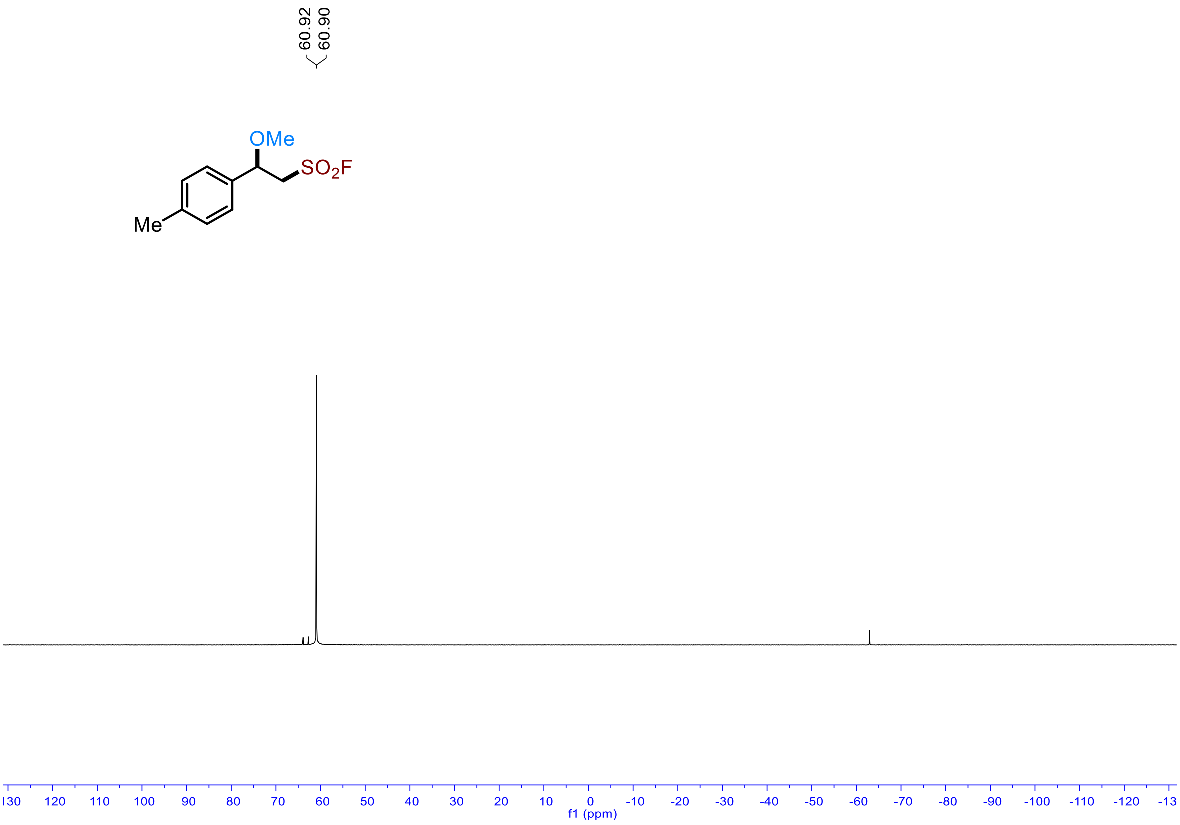

Supplementary Figure 222. ^{19}F NMR spectra of **7q**

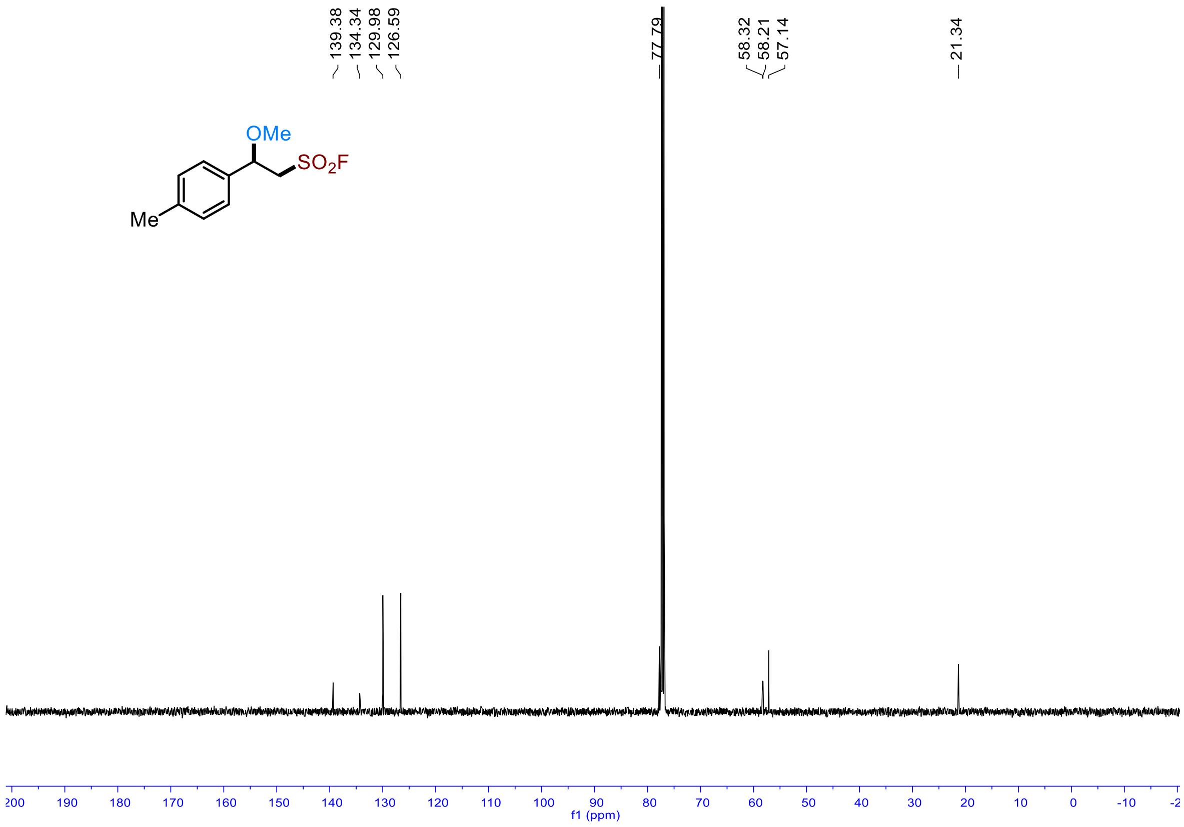

Supplementary Figure 223. ^{13}C NMR spectra of **7q**

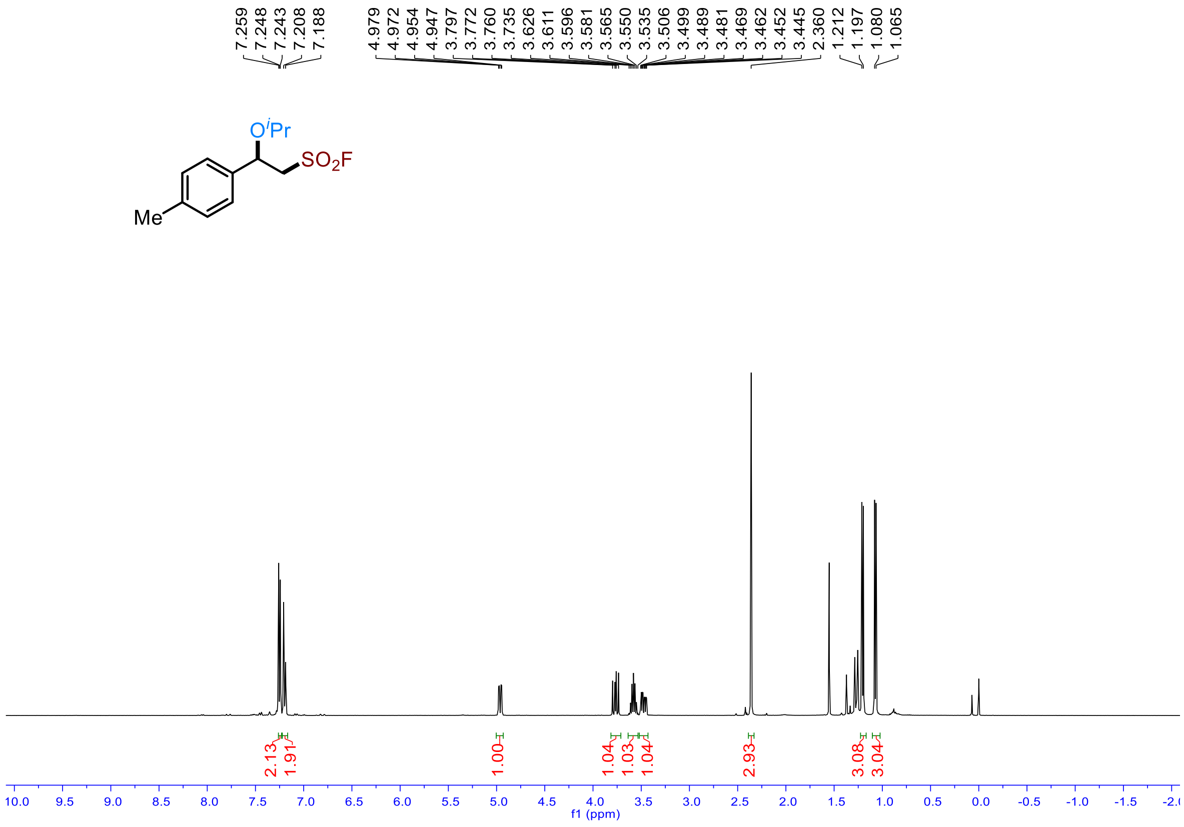

Supplementary Figure 224. ^{1}H NMR spectra of **7r**

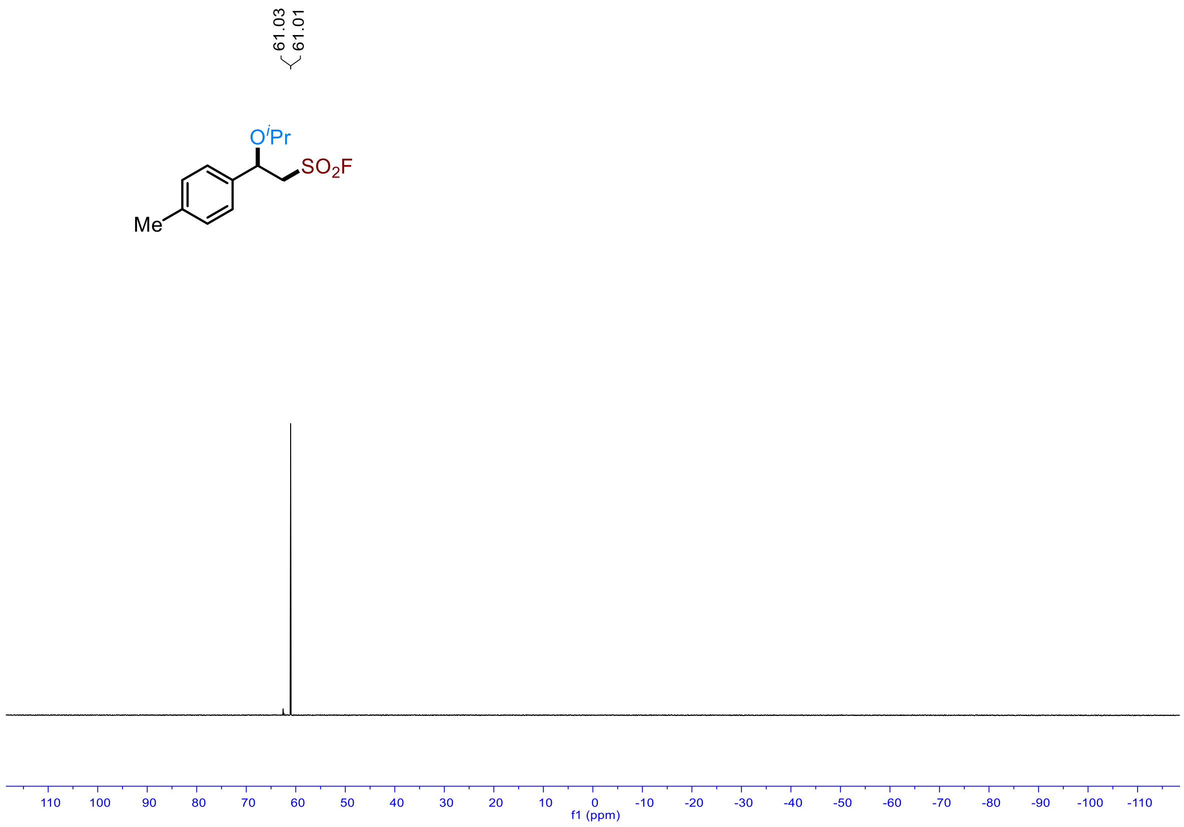

Supplementary Figure 225. ^{19}F NMR spectra of **7r**

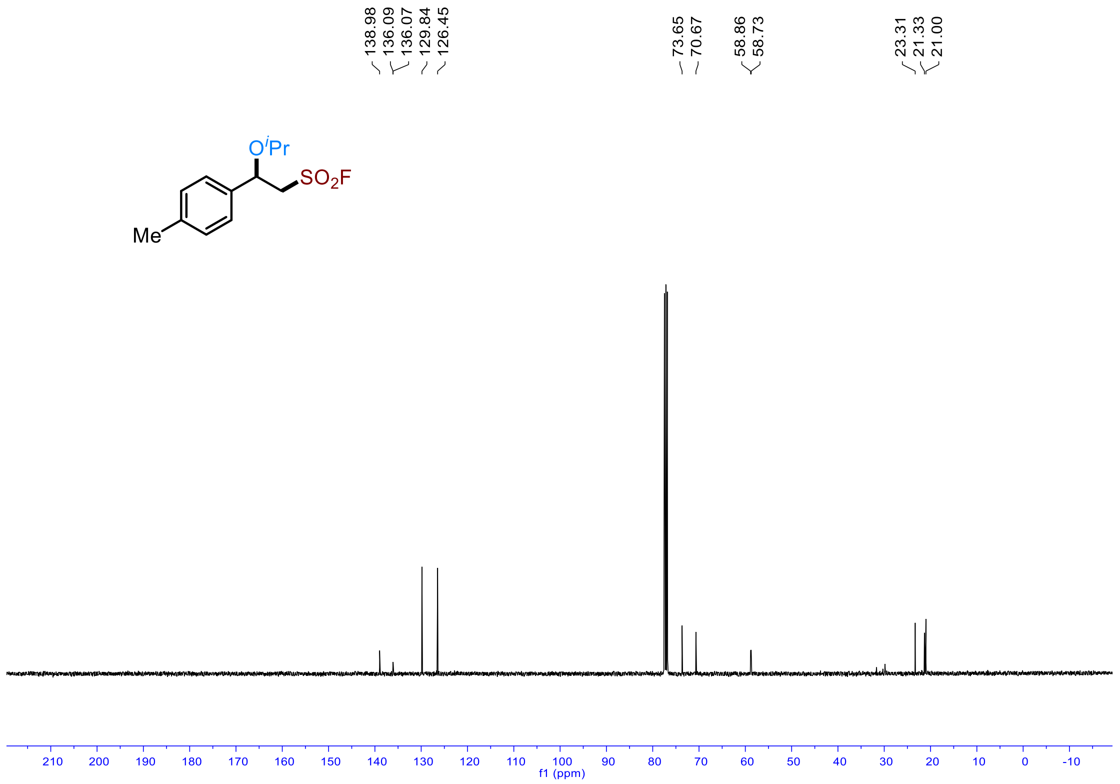

Supplementary Figure 226. ^{13}C NMR spectra of **7r**

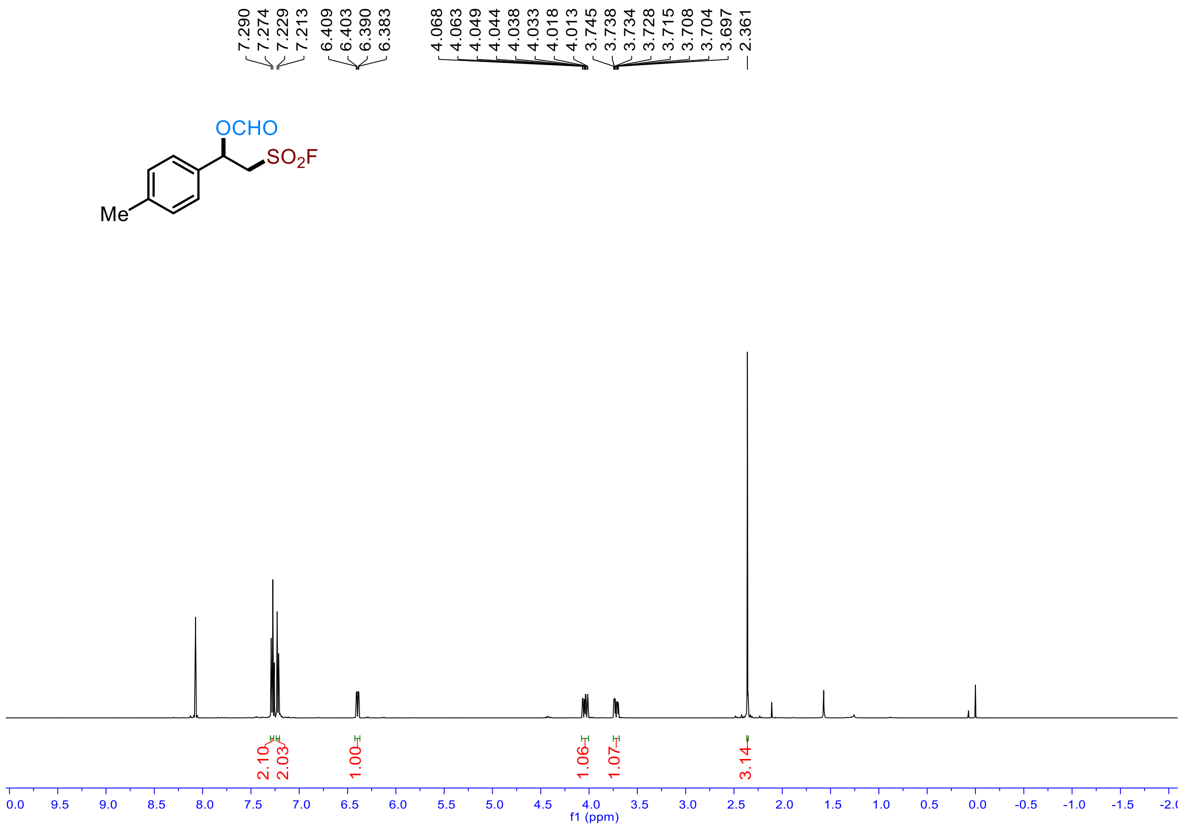

Supplementary Figure 227. ^{1}H NMR spectra of **7s**

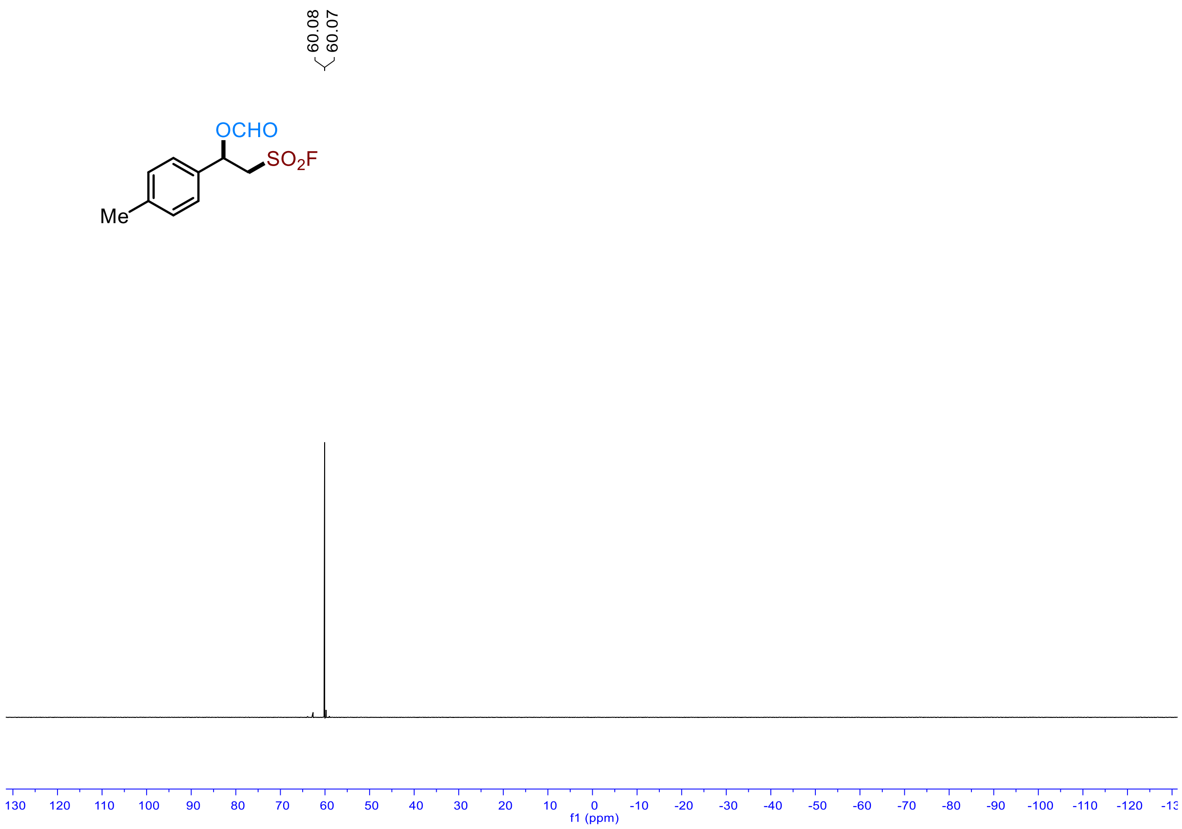

Supplementary Figure 228. ^{19}F NMR spectra of **7s**

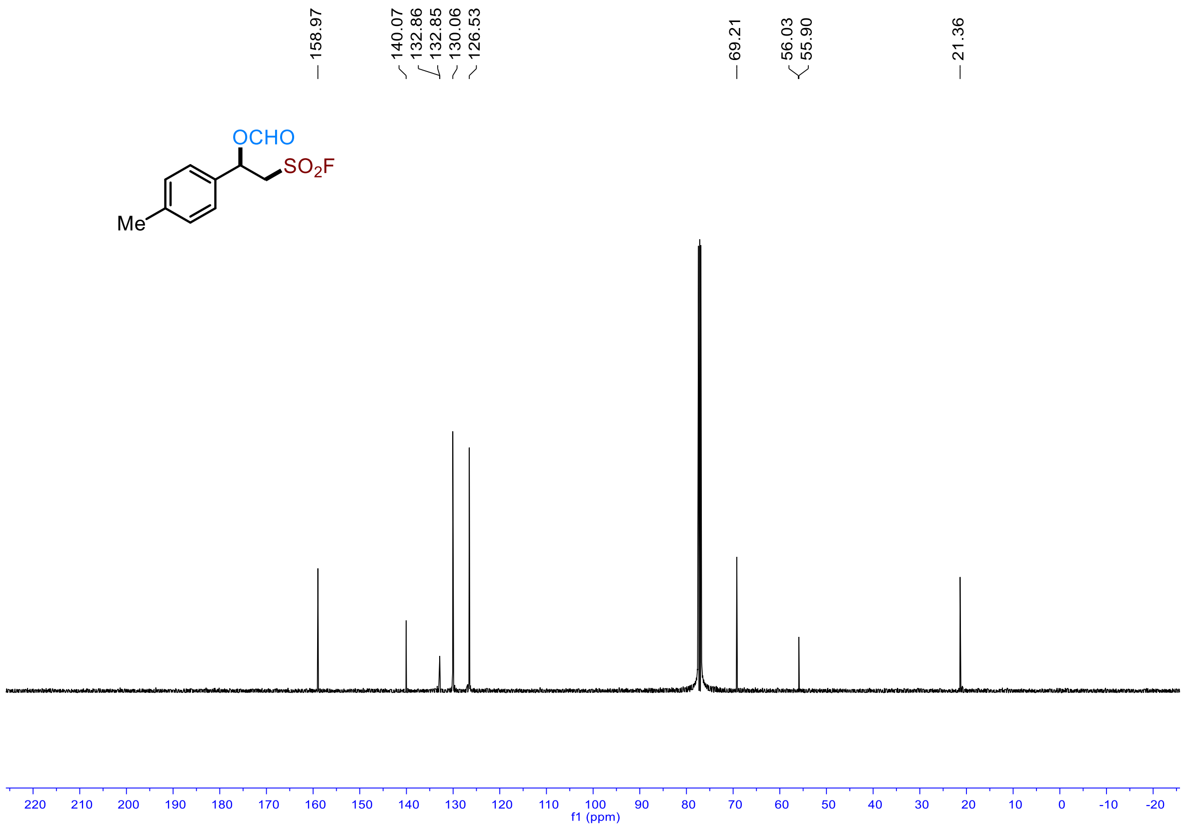

Supplementary Figure 229. ^{13}C NMR spectra of **7s**

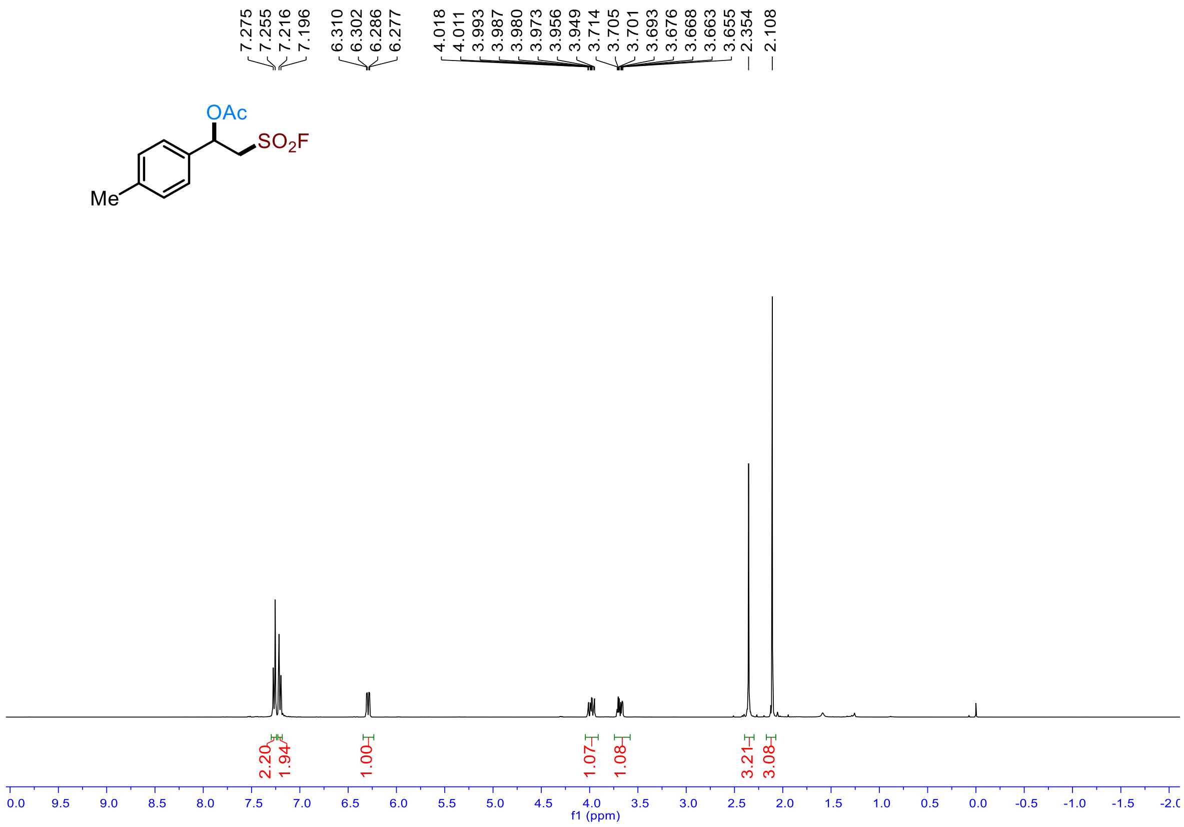

Supplementary Figure 230. ^{1}H NMR spectra of **7t**

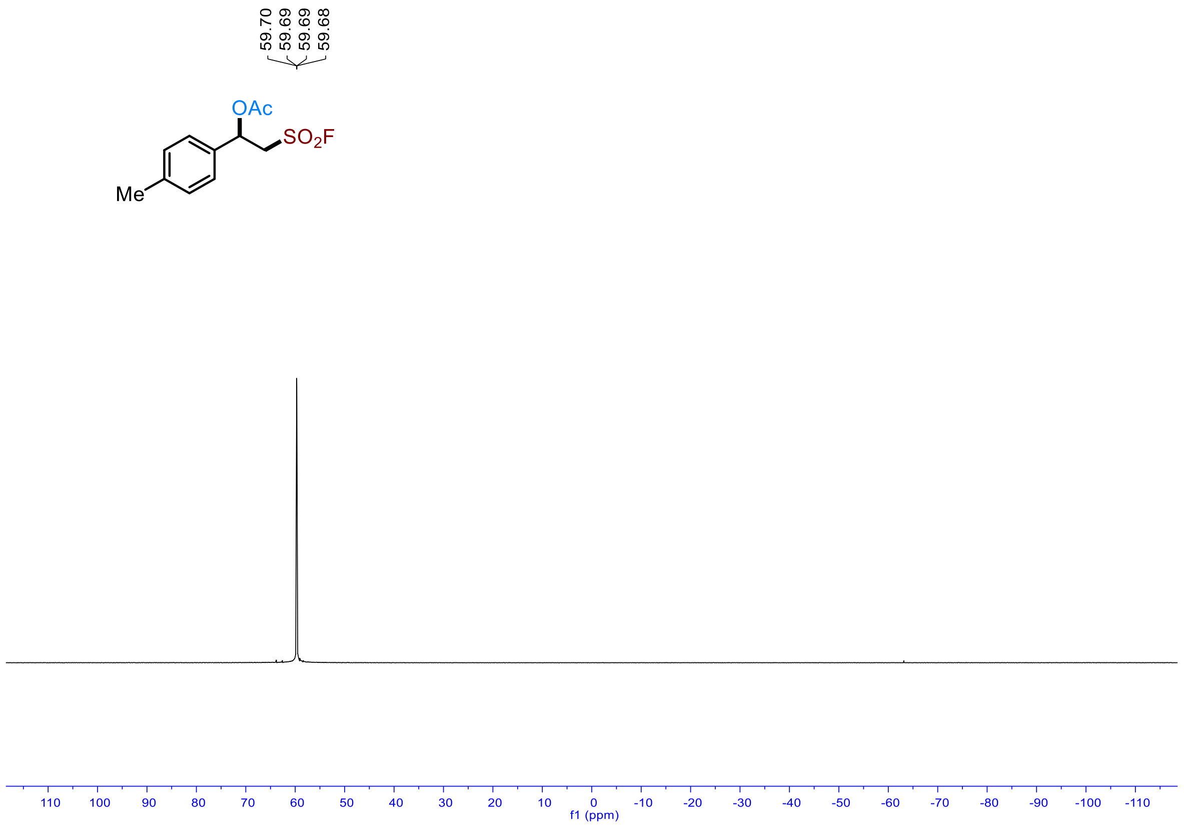

Supplementary Figure 231. ^{19}F NMR spectra of **7t**

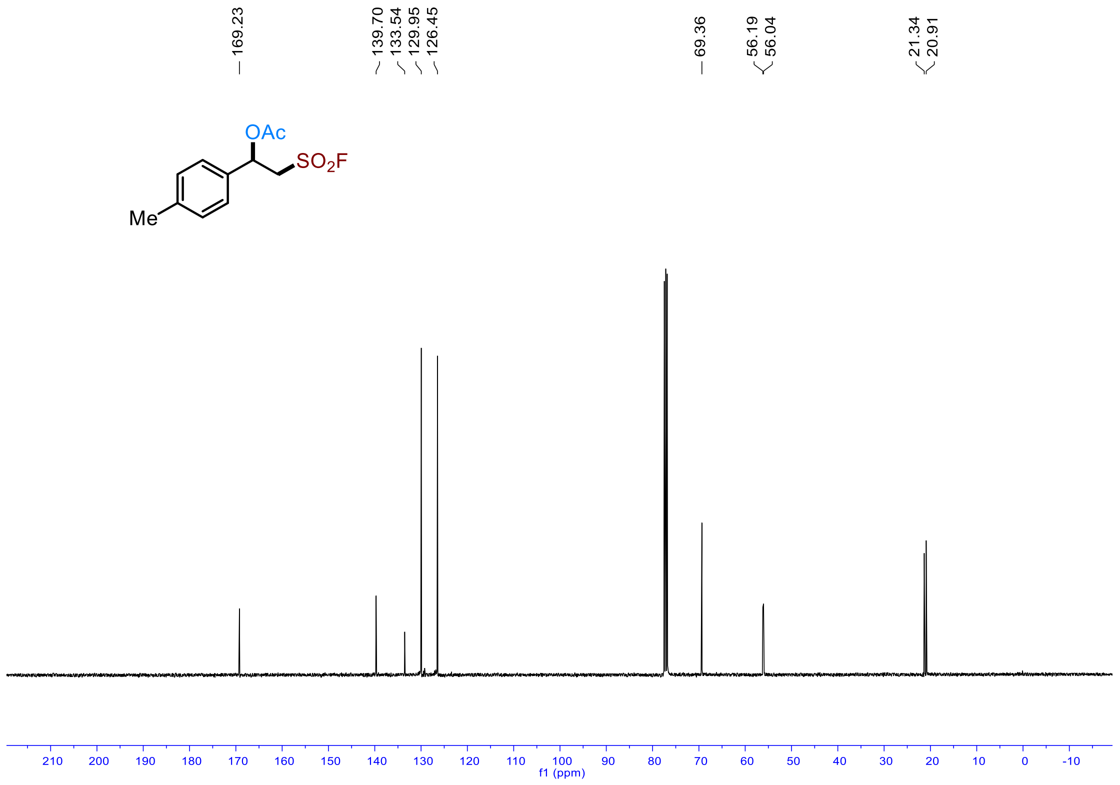

Supplementary Figure 232. ^{13}C NMR spectra of **7t**

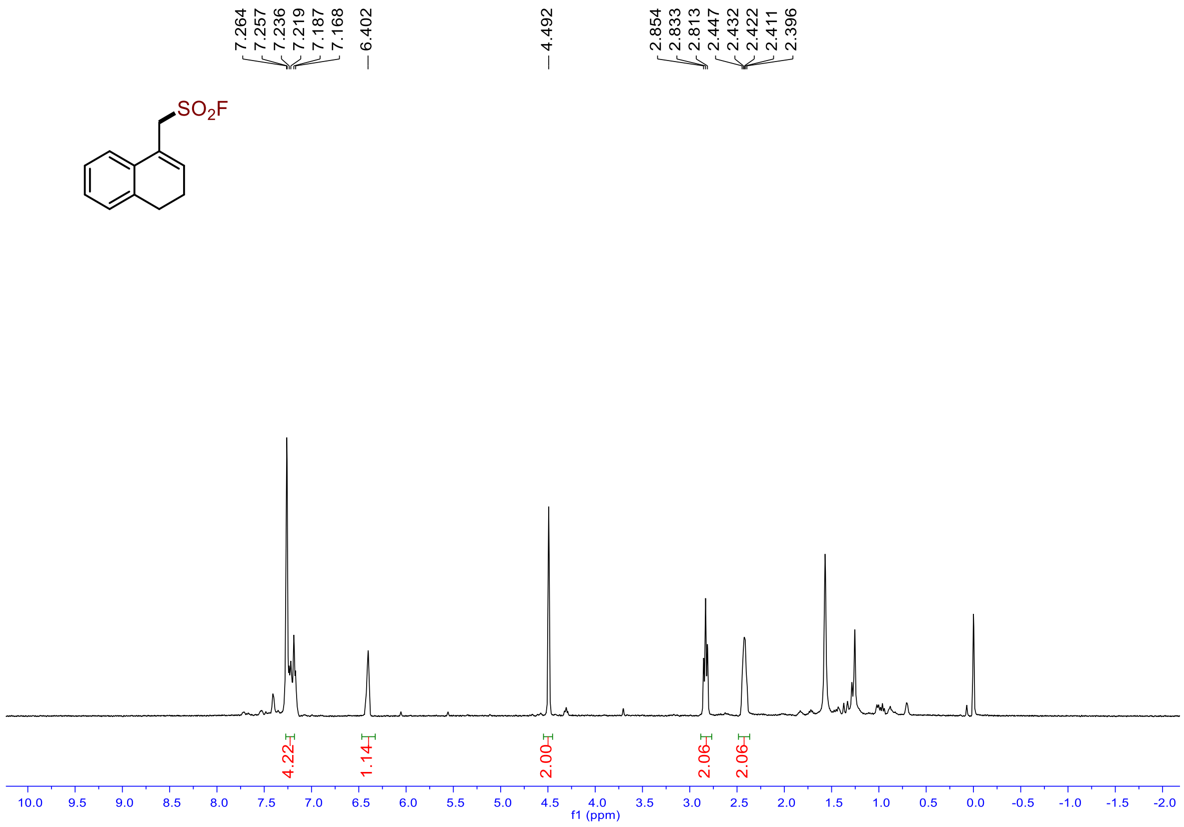

Supplementary Figure 233. ^{1}H NMR spectra of **9**

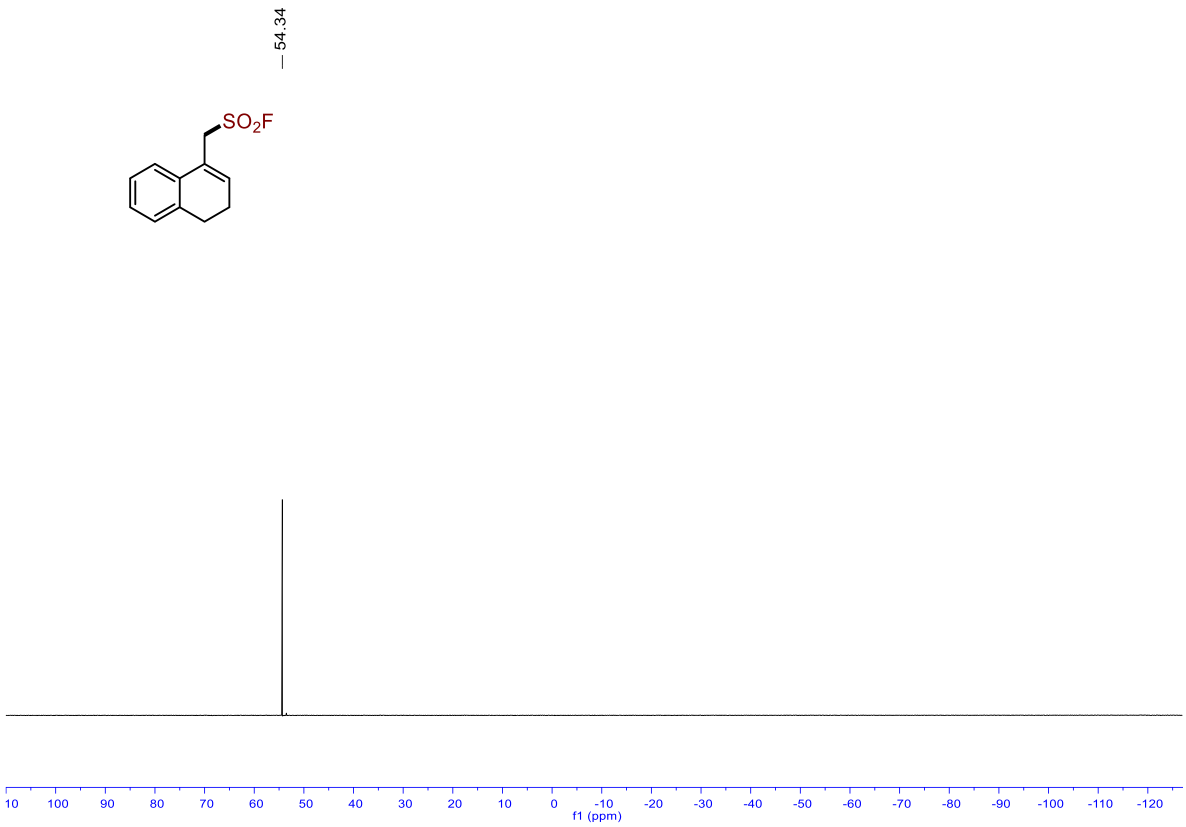

Supplementary Figure 234. ^{19}F NMR spectra of **9**

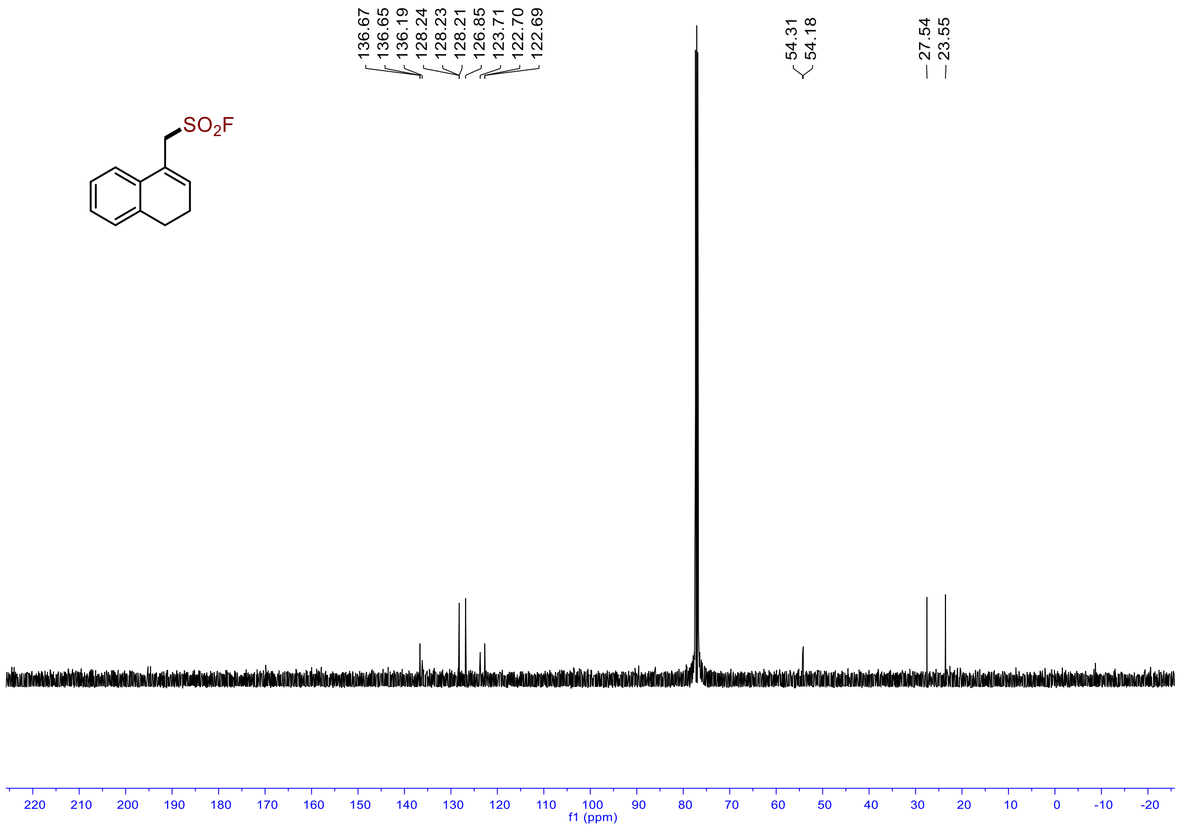

Supplementary Figure 235. ^{13}C NMR spectra of **9**

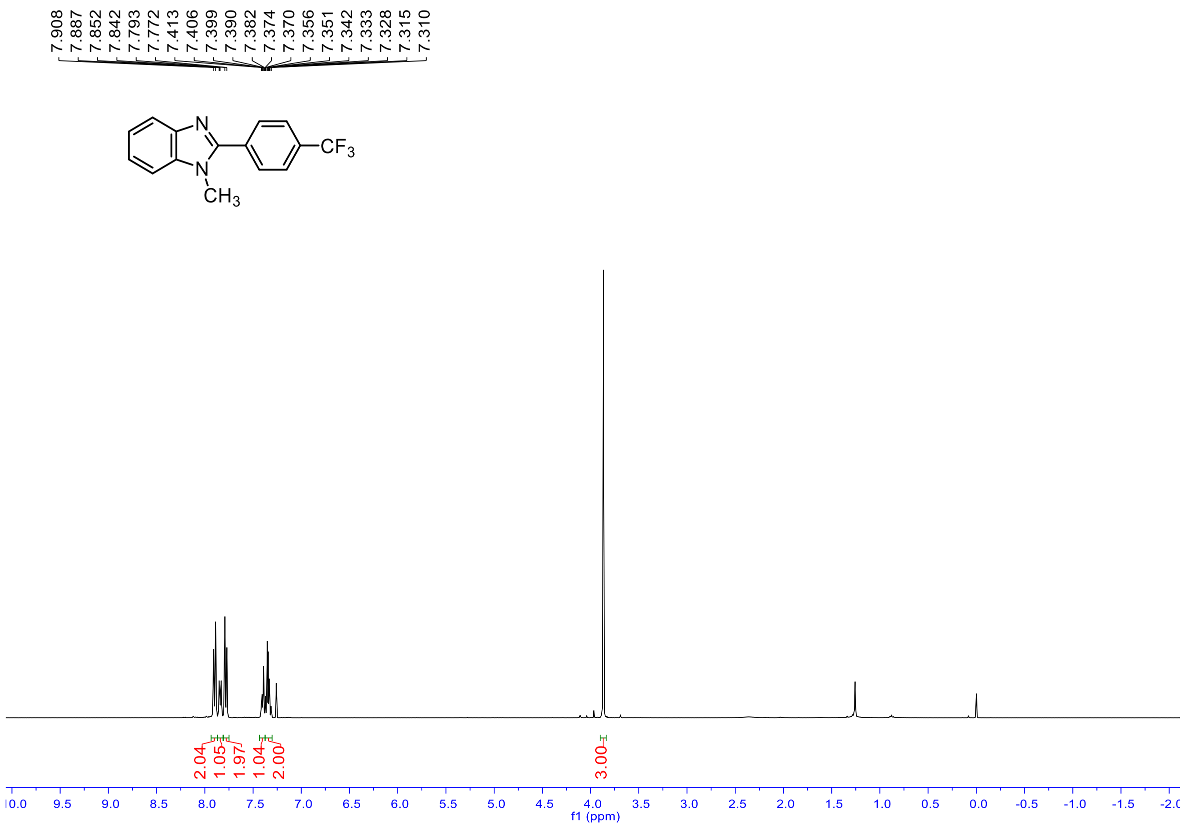

Supplementary Figure 236. ^{1}H NMR spectra of **2e''**

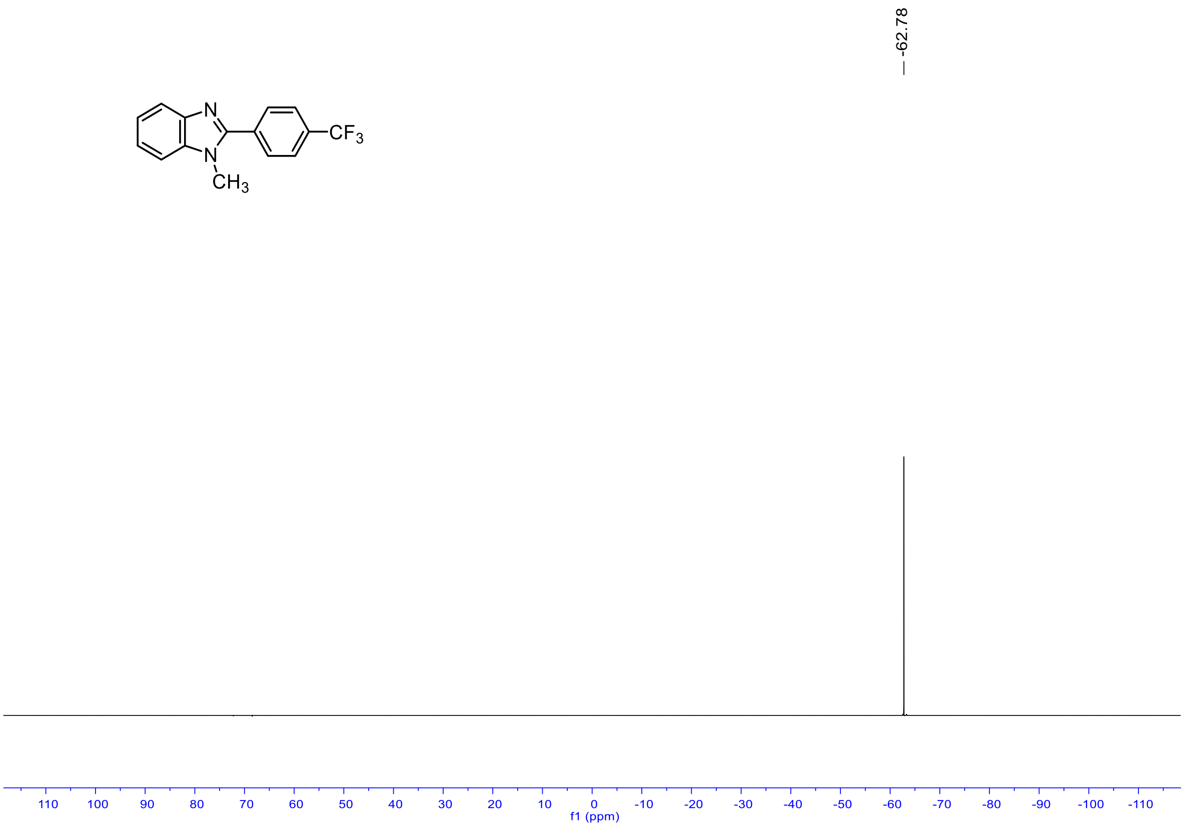

Supplementary Figure 237. ^{19}F NMR spectra of **2e''**

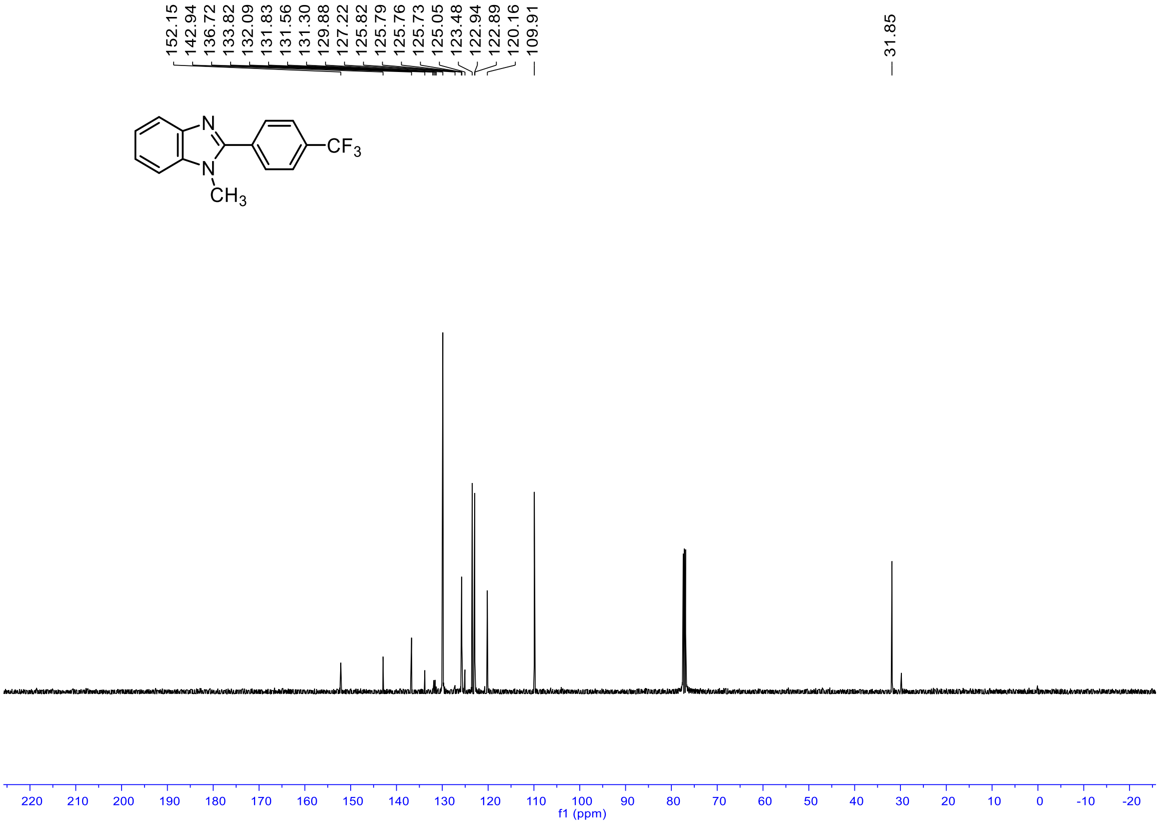

Supplementary Figure 238. ^{13}C NMR spectra of **2e''**

Supplementary References

1. Guo, T.; Meng, G.; Zhan, X.; Yang, Q.; Ma, T.; Xu, L.; Sharpless, K. B.; Dong, J., *Angew. Chem. Int. Ed.* **57**, 2605–2610 (2018).

2. Zhang, W.; Zou, Z.; Zhao, W.; Lu, S.; Wu, Z.; Huang, M.; Wang, X.; Wang, Y.; Liang, Y.; Zhu, Y.; Zheng, Y.; Pan, Y., *Nat. Commun.* **11**, 2572, (2020).

3. Yuan, Y.; Zheng, Y.; Xu, B.; Liao, J.; Bu, F.; Wang, S.; Hu, J.-G.; Lei, A., *ACS Catal.* **10**, 6676–6681 (2020).

4. Nie, X.; Xu, T.; Song, J.; Devaraj, A.; Zhang, B.; Chen, Y.; Liao, S., *Angew. Chem. Int. Ed.* **60**, 3956–3960 (2021).

5. Dong, J., Yang Q., Guo T., Zhan X., Meng G. Fluorosulfonyl-containing compound, intermediate thereof, preparation method therefor and use thereof, WO2019101132 A1 (2019).
